# Chromoselective access to *Z*- or *E*- allylated amines and heterocycles by a photocatalytic allylation reaction

**DOI:** 10.1038/s41467-019-10441-4

**Published:** 2019-06-14

**Authors:** Ana María Martínez-Gualda, Rafael Cano, Leyre Marzo, Raúl Pérez-Ruiz, Javier Luis-Barrera, Rubén Mas-Ballesté, Alberto Fraile, Víctor A. de la Peña O’Shea, José Alemán

**Affiliations:** 1 Organic Chemistry Department, Módulo 1, Universidad Autónoma de Madrid, 28049 Madrid, Spain; 2 Inorganic Chemistry Department, Módulo 7, Universidad Autónoma de Madrid, 28049 Madrid, Spain; 3 Departamento de Química, Universitat Politècnica de València, Camino de Vera s/n, 46022 Valencia, Spain; 4 Institute for Advanced Research in Chemical Sciences (IAdChem), Universidad Autónoma de Madrid, Madrid, 28049 Spain; 5 Photoactivated Processes Unit, IMDEA Energy, Av. Ramón de la Sagra 3C, 28935 Móstoles, Madrid Spain

**Keywords:** Homogeneous catalysis, Synthetic chemistry methodology, Reaction mechanisms, Photocatalysis

## Abstract

The most useful strategies for the alkylation of allylic systems are related to the Tsuji–Trost reaction or the use of different Lewis acids. Herein we report a photocatalytic approach for the allylation reaction of a variety of nucleophiles, such as heteroarenes, amines and alcohols. This method is compatible with a large variety of pyrroles and indoles, containing different substituents such as electron-withdrawing and electron-donating groups, unprotected nitrogen atoms and bromo derivatives. Moreover, this methodology enables the chromoselective synthesis of *Z*- or *E*-allylated compounds. While the use of UV-light irradiation has allowed the synthesis of the previously inaccessible *Z*-allylated products, *E*-isomers are prepared simply by changing both the light source to the visible region, and the catalytic system. Based on mechanistic and photochemical proofs, laser flash photolysis studies and DFT calculations, a rational mechanism is presented.

## Abstract (web summary)

Tsuji–Trost allylation is a traditional method for selective C-C bond formation that involves the use of palladium-based catalysts. Here, the authors report a metal-free, photocatalytic allylation of several heterocycles, amines and alcohols, which can be easily tuned towards the *Z*- or *E*- allylated product.

## Introduction

**Fig. 1:**
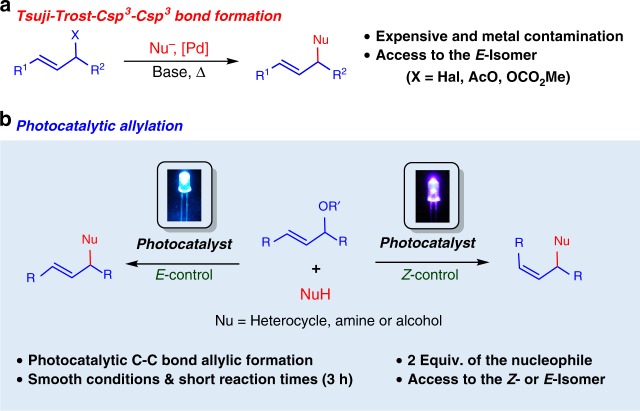
The photocatalytic allylation reaction. **a** Precedents in the Tsuji–Trost allylation and **b** this work

The preparation of allyl-substituted compounds has attracted a special interest due to their utility as building blocks in organic synthesis^1–3^. The Tsuji–Trost reaction^4^ is one of the most powerful methodologies for the alkylation of allylic systems, which is commonly catalyzed by palladium, and the allylic position is usually activated by a halide, an acetate, or a carbonate (eq. a, Fig. 1) and affords exclusively the *E-*isomer. The high selectivity and the general scope of this reaction makes it one of the most prominent Csp^3^–Csp^3^ bond formation methodologies^5^. Indoles and pyrroles are versatile and useful heterocycles for the synthesis of a large variety of biologically active compounds and natural products^6^. Different authors have reported the allylation of indoles at the C-3 position via the Tsuji–Trost reaction in a racemic manner^7–12^. However, although this methodology is very important, to the best of our knowledge, no photocatalytic approaches for the allylation of heterocycles have been reported so far.

Over the past decade, photocatalysis has emerged as a powerful tool for the construction of new bonds that are difficult to obtain using other established procedures^13–31^. A large number of photocatalytic methodologies have been described for the formation of new Csp^2^–Csp^2^ bonds. In particular, the arylation of (hetero)-aromatic rings, usually pyrroles, under different photocatalytic systems has been recently reported^32–40^. However, one of the major problems related to this photocatalytic arylation is the large excess of the heterocycle required in this reaction (24–40 equiv.). Although the photocatalytic heteroatomatic ring arylation has been extensively studied, the photoallylation of heterocycles remains an elusive process.

We hypothesize that the reduction of the allylic derivative by a photocatalyst with the adequate redox potential would result in the appropriate intermediate, which will allow the functionalization of the allylic position. There are two prerequisites to achieve this goal: (i) the development of a photocatalytic system able to activate the C–O bond; (ii) since an unsaturation is present, it is necessary to control the isomerization of the double bond (*Z* or *E*).

In this work, we present a chromoselective photocatalytic allylation of heteroaromatic rings, using smooth conditions and short reaction times to access the *Z*- or *E*-double bonds, depending on the reaction conditions (eq. b, Fig. 1). In addition, mechanistic and photochemical proofs, DFT calculations, and laser flash photolysis studies enabled us to postulate a plausible mechanistic pathway.

## Results

### Optimization of the model reaction

Based on the previous photocatalytic arylation reactions^32–40^, we started the screening of the reaction using the acetate allylic derivative **1a** and pyrrole **2a** (18 equiv.) in the presence of different photocatalysts **3** under light irradiation (Table 1). Transition-metal-based photocatalysts (**3a–3b**) failed to promote the formation of the allylated heterocycle (entries 1 and 2). Several photoorganocatalysts with different reductive power (**3c–3f**) were tested, but only the 10-phenyl-10*H*-phenothiazine (PTH) (**3e**) gave the *Z-*allylated pyrrole **4a** with low conversion under 420-nm LED irradiation (entries 3–6). Encouraged by these results, we used an irradiation source with a wavelength closer to the maximum absorption of PTH (entry 7). Pleasantly, using a 365-nm LED, **4a** was obtained with a 65% conversion. In the absence of a photocatalyst, light, and both, the allylation did not proceed (entries 8–10), confirming the photocatalytic nature of this transformation. Different solvents were then evaluated, and the best result was obtained using CH_3_CN (entries 7 and 11–14). In order to decrease the amount of heterocycle, the reaction was carried out using 10 and 2 equivalents of **2a** (entries 15 and 16) and **4a** was obtained with a good yield of 58% in only 3 h, using just two equivalents of the heterocycle. The use of inorganic bases (Na_2_CO_3_, LiOAc) afforded the final product, although with moderate yield, due to the lower solubility of such bases in acetonitrile (entries 17 and 18).

**Table 1:** Optimization of the photocatalytic allylation reaction^a^


Entry	3 (mol%)	Light (nm)	Solvent	Pyrrole (equiv.)	t (h)	4a:5a^b^
1	**3a** (5)	420	MeCN	18	1	n.r.
2	**3b** (5)	420	MeCN	18	1	n.r.
3	**3c** (5)	530	MeCN	18	1	n.r.
4	**3d** (5)	420	MeCN	18	1	n.r.
5	**3e** (5)	420	MeCN	18	1	100:0 (5%)^c^
6	**3f** (10)	455	MeCN	18	41	n.r.
7	**3e** (5)	365	MeCN	18	1	100:0 (65%)^c^
8	**3e** (5)	–	MeCN	18	1	n.r.
9	–	365	MeCN	18	1	n.r.
10	–	–	MeCN	18	1	n.r.
11	**3e** (5)	365	DMSO	18	1	100:0 (61%)^c^
12	**3e** (5)	365	DMF	18	1	100:0 (49%)^c^
13	**3e** (5)	365	Toluene	18	1	100:0 (21%)^c^
14	**3e** (5)	365	DCM	18	1	100:0 (10%)^c^
15	**3e** (5)	365	MeCN	10	3	94:6 (86%)^c^
**16**	**3e (5)**	**365**	**MeCN**	**2**	**3**	**94:6 (91%)** ^c,d^
17^e^	**3e** (5)	365	MeCN	2	3	80:20 (33%)^c^
18^f^	**3e** (5)	365	MeCN	2	3	78:22 (28%)^c^

^a^Conditions: **1a** (0.1 mmol), **2a** (see table), DIPEA (0.5 mmol), and catalyst (mol%) in the solvent indicated (1.0 mL)

^b^Measured by ^1^H-NMR

^c^Conversion in the crude mixture

^d^Optimized conditions highlighted in bold

^e^Reaction carried out under standard conditions but using Na_2_CO_3_ (0.5 mmol) instead of DIPEA

^f^Reaction carried out under standard conditions but using LiOAc (0.5 mmol) instead of DIPEA

### Substrate scope

Having established the best conditions (Table 1, entry 16), we performed the scope of the reaction (Table 2). With *N*-methyl pyrrole, the allylic derivative **4b** was obtained with a better yield than **4a** and with a similar selectivity for the *Z*-isomer. Other substituents were tolerated at the *N*-atom of the pyrrole (**4c** and **4d**) with excellent *Z*/*E* selectivity (up to 96:4) and with a slight decrease for the phenyl derivative **4c**. Indoles without protecting groups at the nitrogen were also employed, keeping the high selectivity for the *Z*-isomers, and with better yields than with the pyrroles (compare **4e** and **4f** with **4a** and **4b**). Electron-donating groups (EDGs) were well tolerated at different positions of the indole ring (**4g**, **4h,** and **4i**) as well as electron-withdrawing groups (EWG) at the aromatic ring (**4j**). A methyl substituent next to the indolinic nitrogen (**4k**) or the reactive C-3 center (**4l**) did not have a negative influence on the reactivity, obtaining both in very good yields and high selectivity. Remarkably, the presence of Br at the 5-position (**4m**) was also well tolerated under the presence of the high-reducing photocatalyst **3e**. The scope of the allylic derivative was also evaluated. Electron-rich (**4n**, **4o,** and **4p**) as well as electron-poor aromatic rings (**4q**) worked with excellent selectivities (up to > 98:2). We then studied the influence of the leaving group at the allylic position (R group in **1a**). The reaction worked with other leaving groups such as benzoate, carbamate, or carbonate, which were suitable for this process. However, the reaction with the hydroxyl group did not proceed, because of its higher reduction potential (−2.52 V vs. SCE) compared with the other activated allylic alcohols (E = −2.06 to −2.35 V vs. SCE, see Suplementary Note 4 for cyclic voltammetry).

**Table 2:** Scope of the allylation reaction for the synthesis of *Z*-isomers with pyrroles and indoles under catalyst **3e**^a,b^

^a^Conditions: **1** (0.1 mmol), **2** (0.2 mmol), DIPEA (0.5 mmol), and **3e** (5 mol%) in MeCN (1.0 mL)

^b^Isolated yields after flash chromatography

^c^Combined isolated yield along with the C2-allylated compound

**Fig. 2:**
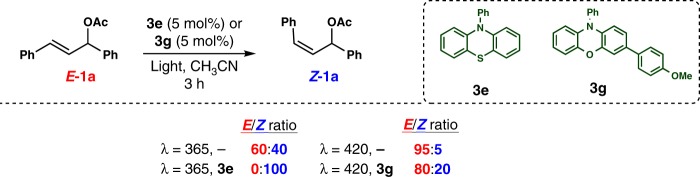
Isomerization studies. Isomerization proofs of *E*-**1a** under different catalysts (**3e** and **3g**) and different irradiation wavelengths

After obtaining these good results with the *Z*-isomer, our next objective was the development of a photocatalytic variant to obtain the corresponding *E*-isomers. To achieve this goal, we analyzed the conditions that avoided the isomerization of the reagent **1a**. A sample containing *E*-**1a** in MeCN was irradiated for 3 h under different reaction conditions (Fig. 2). Without the use of the photocatalyst under 365-nm irradiation, we found a mixture of 60/40 *E*/*Z*-**1a**, while in the presence of **3e**, this isomerization to *Z*-**1a** was complete (Fig. 2). According to theoretical calculations, photosensitization and subsequent isomerization of *E*-**1a** by the photocatalyst is feasible, while photosensitization and subsequent isomerization of *Z*-**1a** cannot take place (see Supplementary Information Fig. 25). The absorption spectra of *E*-**1a** at the reaction conditions revealed a significant absorption at 365 nm (see Supplementary Information Fig. 8), while at 420 nm, it was negligible, suggesting that the reaction must be carried out in the visible-light region to avoid isomerization. Under 420-nm irradiation, only 5% of the *E*-**1a** was isomerized to the *Z*-isomer after 3 h. Therefore, a photocatalyst with high reduction potential (≥2.35 V vs. SCE) and absorption in the visible-light region is required. The phenoxazine **3g**, that meets all these criteria^41^, resulted in only a small amount of *Z*-**1a** at 420-nm irradiation after 3 h (Fig. 2). Therefore, under these conditions (using photocatalyst **3g** and 420-nm irradiation), it should be possible to avoid the isomerization step and selectively form *E*-allylated products **5**.

To our delight, when carrying out the reaction between the allylic derivative **1a** and pyrrole (**2a**) in the presence of the photocatalyst **3g** under 420-nm irradiation, the allylated product *E*-**5a** was obtained with a good yield as the major isomer (Table 3). Other *N*-substituted pyrroles were also employed and maintained the same selectivity (**5b–5c**). Only compound **5d** was obtained as a complex mixture. The reaction with indoles afforded even better yield and selectivity than pyrroles (**5e** and **5f**). Unprotected indolinic nitrogen as well as different substituents were tolerated, from EDGs (**5g–i**) to EWGs (**5j**), methyl (**5k–l**), or bromo derivatives (**5m**), obtaining in all cases good yields (67–92%) and excellent selectivities (up to > 98:2). The isomerization of the final product **5e** under 420-nm irradiation was also studied, obtaining a *Z/E* mixture 30/70 after 3 h of irradiation, without the photocatalyst, while in the presence of the photocatalyst **3g,** a *Z/E* mixture 20/80 was obtained. The final product is present in the reaction in higher concentrations only after 2 h of reaction. Therefore, the irradiation time is not enough to produce its isomerization, which explains the obtention of the *E*-isomer as the major one.

**Table 3:** Scope of the allylation reaction for the synthesis of *E*-isomers with pyrroles and indoles under catalyst **3g**^a,b^

^a^ Conditions: **1a** (0.1 mmol), **2** (0.2 mmol), DIPA (0.5 mmol), and **3g** (5 mol%) in MeCN (1.0 mL)

^b^Isolated yields after flash chromatography

^c^Combined isolated yield along with the C2-allylated compound

### Mechanistic studies

**Fig. 3:**
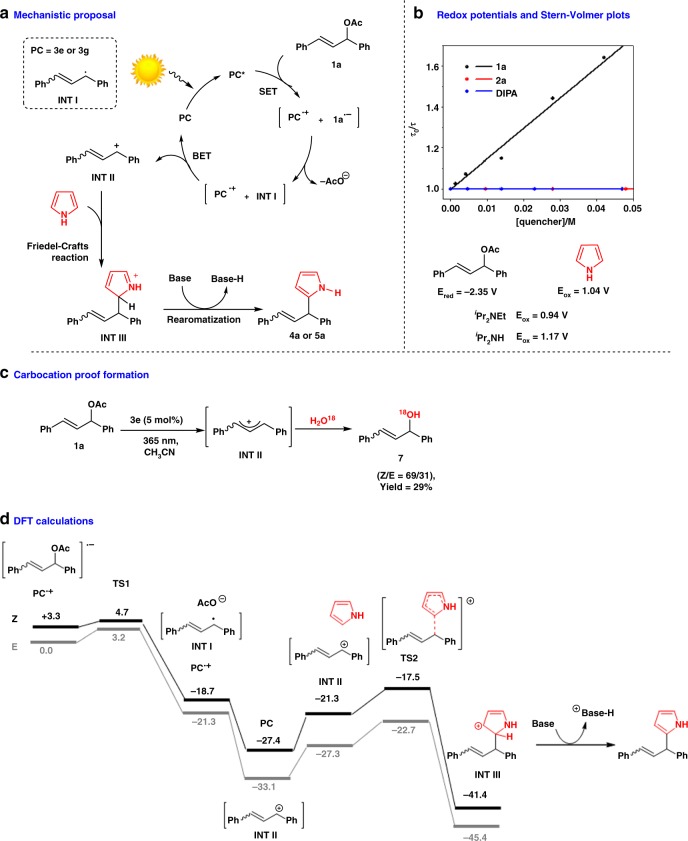
Mechanistic studies. **a** Mechanistic proposal for the photoallylation. **b** Redox potentials and Stern–Volmer plots of the time-resolved fluorescence quenching of **3g** with **1a**, pyrrole, and DIPA (DIPA = diisopropylamine). **c** Reaction of **1a** with H_2_O^18^ under standard reaction conditions. **d** For DFT calculations, geometry optimizations were performed using the M06-2X functional in combination with the 6-311G** basis set

The proposed reaction mechanism is outlined in Fig. 3a. After light absorption by the photocatalyst under LED irradiation (*λ* = 365 or 420 nm), single-electron transfer (SET) takes place from its S_1_ excited state (*E*_S1_ = 3.2 eV, see Supplementary Information Figs. 11 and 12) to **1a**. Steady-state and time-resolved fluorescence quenching studies in the presence of **1a** afforded a quenching rate constant of *k*_q_(S_1_) = 4.7 × 10^9^ M^−1^ s^−1^ (see Supplementary Information Fig. 9a), indicating that the radical ion pair (**PC**^•+^ + **1a**^•−^) formation occurs at nearly diffusion rate. In addition, SET from the excited singlet state would be an exergonic process, taking into account the free energy change (Δ*G*_ET_ = −4.0 kcal mol^−1^) associated with the electron transfer (see Supplementary Note 4 for Rehm–Weller equation).

Importantly, photooxidation of DIPEA (*E*_ox_ = 0.94 V vs. SCE)^42^, DIPA (*E*_ox_ = 1.17 V vs. SCE)^43^, or pyrrole (*E*_ox_ = 1.04 V vs. SCE)^44^ by **PC** S_1_ excited state could not occur, taking into account the oxidation power of **3e** and **3g** (*E*_(Pc*/Pc·−)_ = −0.3 V vs. SCE for **3e** and **3g**, see Supplementary Note 4), and was further confirmed by fluorescent-quenching studies (see Fig. 3b). The fate of such reduced species has been investigated by DFT calculations, considering both *Z-* and *E*-isomers (Fig. 3d). Initial single-electron transfer process from the photocatalyst (**PC**) to **1a** generates the radical cation **PC**^**·+**^ and the radical anion **1a**^**·**−^, that evolves through the C–O bond scission to afford acetate anion and the radical intermediate **I** (**INT I**). This step is a very exergonic process (−22 or −21.3 kcal mol^−1^) and proceeds through a very shallow kinetic barrier (*E*_a_ = 1.4 or 3.2 kcal mol^−1^). Then, the oxidation of **INT I** by the oxidized photocatalyst (**PC**^**·+**^), results in the regeneration of the photocatalyst (**PC**) and formation of a carbocationic intermediate **II** (**INT II**) (Fig. 3d). Such electron transfer is calculated as a thermodynamically favorable process (−8.7 or −11.8 kcal mol^−1^). The calculated energetic barriers for *E* to *Z* isomerizations for radical or carbocation intermediates **I** and **II** rule out this process from such transient species (see Supplementary Information Fig. 28). Formation of this carbocation **INT II** was experimentally confirmed, carrying out the reaction in the presence of H_2_O^18^ as the nucleophile obtaining the isotopically labeled compound **7** (Fig. 3c). In addition, when two nonsymmetric allylic derivatives bearing different aryl groups were studied, an equimolecular mixture of products was obtained (see Supplementary Fig. 30, compounds **6** and **6′**), indicating that the reaction takes places through a common intermediate. Then, a Friedel–Crafts reaction between the carbocation **INT II** and pyrrole takes place, generating the protonated intermediate **III** (**INT III**). This step is also theoretically found exergonic (−14 or −26.3 kcal mol^−1^) and kinetically favorable (*E*_a_ = 3.8 kcal mol^−1^). A final rearomatization by deprotonation of **INT III** gives the final product (Fig. 3d). For such deprotonation, both DIPEA and the anion acetate (formed during the reaction) would act as a base through very exergonic processes (see entries 16 and 17 from Table 1 for reactions in the presence of Na_2_CO_3_ and LiOAc).

**Fig. 4:**
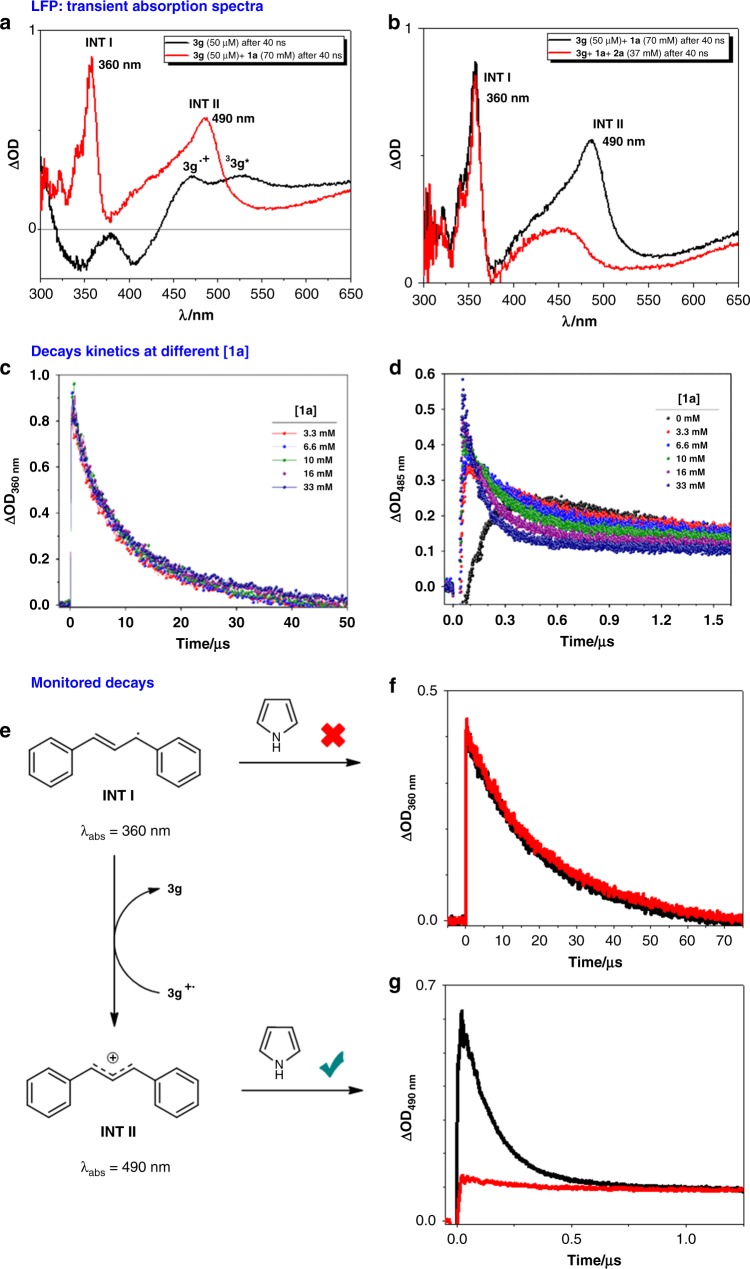
Laser flash photolysis (*λ*_exc_ = 355 nm, MeCN/Ar) experiments. **a** Transient absorption spectra recorded at 40 ns after the laser pulse of **3g** (50 mM) without **1a** (black), with 70 mM of **1a** (red). **b** Transient absorption spectra recorded at 40 ns after the laser pulse of **3g** (50 mM) with 70 mM of **1a** (black) and with 35 mM of **2a** (red). **c** Decay kinetics at 360 nm after 355-nm LFP of **3g** (50 µM) in the presence of increasing amounts of **1a**. **d** Decay kinetics at 485 nm after 355-nm LFP of **3g** (50 µM) in the presence of increasing amounts of **1a**. **e** Scheme of the formation of intermediate **II** from intermediate **I** and their reaction with **2a**. **f** Lifetime of **INT I**: decays monitored at 360 nm of **3g** (50 mM) and **1a** (70 mM) (black line) and in the presence of **2a** (37 mM) (red line). **g** Lifetime of **INT II**: decays monitored at 490 nm of **3g** (50 mM) and **1a** (70 mM) (black line) and in the presence of **2a** (37 mM) (red line)

In order to gain a better understanding of the reaction mechanism, laser flash photolysis (LFP) measurements have been carried out. Excitation of **3g** at 355 nm results in two peaks at 468 and 530 nm at 40 ns after the laser pulse, which are assigned to the **3g** radical cation (**3** **g**^•+^) and the excited triplet state of **3g** (^**3**^3g*), respectively (Fig. 4a, black line, for further details, see also Supplementary Note 2). This experiment was performed in the presence of **1a** to identify the possible transient reaction intermediates (Fig. 4a, red line). Two new absorption bands at 360 and 490 nm are clearly observed, which correspond to intermediates **I** and **II**, respectively, based on literature data^45^. The lifetime of the carbocation **INT II** also depends on the nucleophilicity of the anionic leaving group (see Supplementary Information Fig. 4). In order to check whether formation of **INT II** and **INT I** is instantaneous with the laser pulse, additional LFP experiments of **3g** in the presence of increasing amounts of **1a** were performed (Fig. 4c and d). Generation of **INT II** is practically instantaneous even at lower concentration of **1a** (Fig. 4d), whereas lifetimes of **INT I** are not affected by higher amounts of **1a** (Fig. 4c). This result suggests that SET from 3g* to **1a** at diffusion control rate (see Supplementary Information Fig. 9) gives rise to the contact radical ion pair at this singlet stage (Fig. 3a). All processes in the contact radical ion pairs undergo in the sub-nanosecond scale^46^. Fast acetate release from **1a**^**·**−^ led to **INT I**, which is still in close contact with **3g**^•+^. At this point, **INT I** undergoes an ultrafast back electron transfer with **3g**^•+^ restoring **3g** and generating **INT II,** whose amount is slightly dependent on the concentration of **1a** in the sample (Fig. 4d). In addition, **3g**^•+^ and **INT I** can split up, forming the corresponding free **3g**^•+^ and free **INT I**, that are detected in the LFP experiments with lifetimes in the microsecond scale (Fig. 4a, red line). Once the detection of both intermediates **I** and **II** by LFP has been established, the question arises whether **INT I** or **INT II** (radical or carbocation) would react with a trapping agent (Fig. 4e and transient absorption spectrum in Fig. 4b). Addition of pyrrole to a **3g**/**1a** mixture results only in a marked decrease of the **INT II** lifetime (Fig. 4g), while the band corresponding to **INT I** (360 nm) is not affected (Fig. 4f). Therefore, this experiment clearly corroborated with the previous data (Fig. 3) that the carbocation **INT II** is the reactive intermediate in our reaction. A quantum yield of 1.5% was found, suggesting a photocatalytic process without a significant radical chain propagation^47^.

### Scope with alcohols and amines

Once that we proved that the reaction takes place through a carbocation intermediate formation, we decided to study other nucleophiles to prove the generality of our protocol. Allylic amines are very useful compounds that can be employed as building blocks for the synthesis of amino acids, alkaloids, and carbohydrate derivatives^48–50^. Moreover, this structure is present in numerous natural products and drugs with antifungal, antibacterial, and anti-inflammatory action^51–53^. Different amines were tried under UV irradiation in order to obtain the *Z*-allylated amines which are not accessible by other methodologies (Table 4). Aniline gave the corresponding *Z*-allylated amine **9a** with high selectivity. Aromatic amines with EDGs were well tolerated (**9b–d**) with moderate selectivity, whereas anilines with EWGs gave significant better yield (**9e**) and *Z*/*E* ratio. In addition, the presence of Br at the aromatic ring was tolerated without detecting the corresponding reduced product (**9f**). Aliphatic primary and secondary amines were also suitable for the reaction conditions (**9g–h**). Cyclic allylated amine **9i** was obtained in good selectivity (*Z*:*E* = 91:9) and good yield. Moreover, the use of morpholine as a nucleophile can be employed for the synthesis of **9j** with excellent selectivity. The preparation of allylic ethers has a great interest, as they are also present in numerous pharmaceuticals and natural products^54–57^. For this reason, alcohols were employed as nucleophiles, obtaining *Z*-allylated ethers with good yields and good selectivities (**9k–m**)^58^.

**Table 4:** Scope of the allylation reaction with amines and alcohols for the synthesis of *Z*-isomers under photocatalyst **3e**^a,b^

^a^ Conditions: **1a** (0.1 mmol), **2** (0.2 mmol), DIPEA (0.5 mmol), and **3g** (5 mol%) in MeCN (1.0 mL)

^b^Isolated yields after flash chromatography

Using the visible-light irradiation conditions and photocatalyst **3g** with amines and alcohols is possible to obtain the corresponding *E*-isomers (Table 5). Aromatic amines gave the corresponding allylated compounds with high selectivities and good yields with EDGs (**10b–d**) and EWGs (**10e**), or *ortho*-bromo substituents (**10f**). Aliphatic primary (**10g**) and secondary amines (**10h–j**) were employed, keeping in all the cases high *Z*/*E* selectivity. Moreover, allylated ethers can also be obtained under visible-light irradiation with excellent selectivities (**10k–m**). A similar mechanistic scenario was found for amines and alcohols, using *p*-toluidine **8b** as a nucleophile in the LFP and photochemical mechanistic probes (see Supplementary Fig. 9).

**Table 5:** Scope of the allylation reaction with amines and alcohols for the synthesis of *E*-isomers under photocatalyst **3g**^a,b^

^a^ Conditions: **1a** (0.1 mmol), **8** (0.2 mmol), and **3g** (5 mol%) in MeCN (1.0 mL)

^b^Isolated yields after flash chromatography

^c^Reaction performed by adding DIPEA (0.5 mmol)

## Discussion

In summary, a chromoselective photocatalytic approach for the allylation of indoles, pyrroles, amines, and alcohols has been developed. This approach represents a photocatalytic allylation reaction for the synthesis of demand of *Z*- or *E*-isomers, with only two equivalents of the desired nucleophile. Therefore, under UV-light irradiation *Z*-allylated products are obtained, while the *E*-isomer is simply prepared by changing both the light source to the visible region, and the catalytic system. DFT calculations, photochemical proofs, and mechanistic experiments indicate that the most plausible mechanism involves a nucleophilic attack to an allyl-cation intermediate.

## Methods

### Procedure for the preparation of *Z*-allylic compounds

A vial equipped with a magnetic stir bar and fitted with a Teflon screw cap septum was charged with the corresponding allylic compound **1** (0.1 mmol), the corresponding heterocycle, amine, or alcohol (0.2 mmol), *N*-phenyl phenothiazine (1.4 mg, 5 mol%), DIPEA (86 μL, 0.5 mmol), and acetonitrile (1 mL). The reaction was degassed with three freeze–pump–thaw cycles. The vial was then backfilled with N_2_ and stirred under 365-nm LED irradiation (8.2460 W m^−2^ intensity; approximate distance was 2 cm from the vial) at 20 °C. After 3 h, the vial was opened, the solvent evaporated, and the crude product was purified by column chromatography to give the corresponding products **4** or **9**.

### Procedure for the preparation of *E*-allylic compounds

A vial equipped with a magnetic stir bar and fitted with a Teflon screw cap septum was charged with the corresponding allylic compound **1** (0.1 mmol), the corresponding heterocycle, amine, or alcohol (0.2 mmol), 3-(4-methoxyphenyl)-10-phenyl-10H-phenoxazine (1.7 mg, 5 mol%), DIPA (70 μL, 0.5 mmol, only base is needed for reactions with heterocycles as nucleophile), and acetonitrile (1 mL). The reaction was degassed with three freeze–pump–thaw cycles. The vial was then backfilled with N_2_ and stirred under 420-nm LED irradiation (18.3396 W m^−2^ intensity; approximate distance was 2 cm from the vial) at room temperature. After 3 h, the vial was opened, the solvent evaporated, and the crude product was purified by column chromatography to give the corresponding products **5** or **10**.

## Supplementary information

Supplementary Information

## Data Availability

The authors declare that all data supporting the findings of this study are available within the article and Supplementary Information files, and also are available from the corresponding author upon reasonable request.
