# Supplementary material for: Chromoselective access to Z- or E- allylated amines and heterocycles by a photocatalytic allylation reaction
Source: Nat Commun. 2019 Jun 14;10:2634. doi: 10.1038/s41467-019-10441-4 (PMC6572830; doi:10.1038/s41467-019-10441-4)
Supplement: Supplementary file 1 — Supplementary Information [file 41467_2019_10441_MOESM1_ESM.pdf]

Supplementary Information

Chromoselective Access to *Z*- or *E*- Allylated Amines and Heterocycles by a Photocatalytic Allylation Reaction

Ana María Martínez-Gualda, et al

Supplementary Methods.

General Experimental Details

The solvents employed in the reactions were used without any further purification. The reactions were carried out in vials and stirred with a magnetic bar under inert atmosphere.

NMR spectra were acquired on a Bruker 300 spectrometer, running at 300 and 75 MHz for ^{1}H and ^{13}C, respectively. Chemical shifts (δ) are reported in ppm relative to residual solvent signals ($CDCl_3$, 7.26 ppm for ^{1}H NMR and 77.00 ppm for ^{13}C NMR). ^{13}C NMR spectra were acquired on a broadband decoupled mode. The following abbreviations are used to describe peak patterns when appropriate: s (singlet), d (doublet), t (triplet), q (quartet), quint (quintet), sept (septuplet), m (multiplet), br (broad).

Analytical thin layer chromatography (TLC) was performed using pre-coated aluminium-backed plates, with fluorescence indicator to 254 nm, and visualized by ultraviolet irradiation. Purification of reaction products was carried out by flash chromatography (FC).

High Resolution Mass Spectra (HRMS) were acquired on a spectrometer Agilent Technologies 5977B MSD using electron ionization (EI) making use of the MassWorks software ver. 4.0.0.0. (Cerno Bioscience) for the formula identification. MassWorks is a MS calibration software, which calibrates for isotope profile as well as for mass accuracy allowing highly accurate comparisons between calibrated and theoretical spectra.[1],[2],[34] Obtained data are expressed in mass/charge (m/z) units.

Commercially available reagents and catalyst were used without further purification.

Supplementary Notes 1.

Synthesis of allylic derivatives (1)

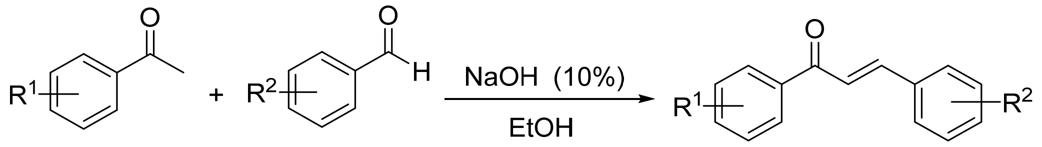

To a stirred solution of the corresponding ketone (5 mmol) in EtOH (14 mL), an aqueous solution of NaOH 10% (6 mL) was added dropwise at 0 ºC. After 5 min, the corresponding aldehyde was added dropwise and the mixture allowed to stir at room temperature until

complete conversion (4-6 h). After reaction completion, the mixture was diluted with water (10 mL). If the chalcone was precipitated at this stage, it was filtered and washed with water and with the minimum amount of EtOH, dried and used in the next step without further purification. If the chalcone was not precipitated, the reaction mixture was extracted three times with DCM (10 mL). The combined organic phases were dried over $MgSO_4$ and the solvent evaporated under reduced pressure. The crude oil was used in the next step without further purification.

(*E*)-1,3-di-*p*-tolylprop-2-en-1-one

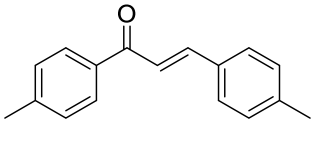

From 1-(*p*-tolyl)ethan-1-one (0.66 mL, 5 mmol) and 4-methylbenzaldehyde (0.59 mL, 5 mmol), following the general procedure, (*E*)-1,3-di-*p*-tolylprop-2-en-1-one (1.180 g, 4.99 mmol) was obtained in 99% yield as a white solid. Spectroscopic data are in agreement with the published data.[5] **^{1}H-NMR (300 MHz, $CDCl_3$)** δ 7.94 (d, *J* = 6.7 Hz, 2H), 7.80 (d, *J* = 15.5 Hz, 1H), 7.60 – 7.35 (m, 3H), 7.35 – 7.20 (m, 4H), 2.45 (s, 3H), 2.40 (s, 3H).

(*E*)-1,3-di-*m*-tolylprop-2-en-1-one

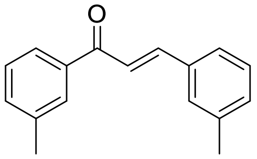

From 1-(*m*-tolyl)ethan-1-one (0.68 mL, 5 mmol) and 3-methylbenzaldehyde (0.59 mL, 5 mmol), following the general procedure, (*E*)-1,3-di-*m*-tolylprop-2-en-1-one (1.044 g, 4.42 mmol) was obtained in 88% yield as a pale yellow oil. Spectroscopic data are in agreement with the published data.[6] **^{1}H-NMR (300 MHz, $CDCl_3$)** δ 7.85 – 7.75 (m, 3H), 7.55 – 7.37 (m, 5H), 7.35 – 7.28 (m, 1H), 7.25 – 7.20 (m, 1H), 2.45 (s, 3H), 2.41 (s, 3H).

(*E*)-1,3-bis(3,5-dimethoxyphenyl)prop-2-en-1-one

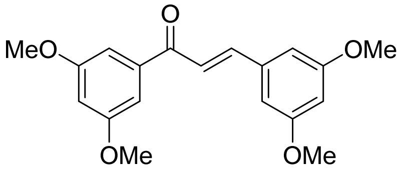

From 1-(3,5-dimethoxyphenyl)ethan-1-one (901.0 mg, 5 mmol) and 3,5-dimethoxybenzaldehyde (831.0 mg, 5 mmol), following the general procedure, (*E*)-1,3-bis(3,5-dimethoxyphenyl)prop-2-en-1-one (1.412 g, 4.30 mmol) was obtained in 86% yield as a white solid. Spectroscopic data are in agreement with the published data.[7] **^{1}H-NMR (300 MHz, $CDCl_3$)** δ 7.72 (d, *J* = 15.7 Hz, 1H), 7.41 (d, *J* = 15.6 Hz, 1H), 7.14 (d, *J* = 2.4 Hz, 2H), 6.78 (d, *J* = 2.4 Hz, 2H), 6.68 (t, *J* = 2.2 Hz, 1H), 6.53 (t, *J* = 2.2 Hz, 1H), 3.87 (s, 6H), 3.85 (s, 6H).

(*E*)-1,3-bis(4-fluorophenyl)prop-2-en-1-one

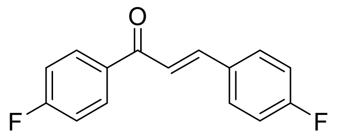

From 1-(4-fluorophenyl)ethan-1-one (0.61 mL, 5 mmol) and 4-fluorobenzaldehyde (0.54 mL, 5 mmol), following the general procedure, (*E*)-1,3-bis(4-fluorophenyl)prop-2-en-1-one (927 mg, 3.80 mmol) was obtained in 76% yield as a white solid. Spectroscopic data are in agreement with the published data.[8] **^{1}H-NMR (300 MHz, $CDCl_3$)** δ 8.10 – 8.00 (m, 2H), 7.79 (d, *J* = 15.8 Hz, 1H), 7.70 – 7.60 (m, 2H), 7.44 (d, *J* = 15.8, 1H), 7.25 – 7.08 (m, 4H).

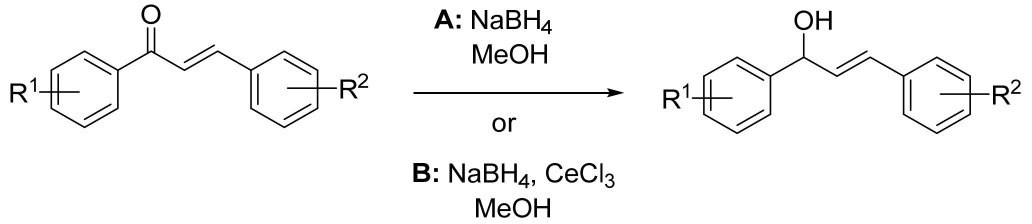

Procedure **A** for chalcone reduction: To a stirred solution of the corresponding chalcone (1.5 mmol) in MeOH (3 mL) at 0 ºC, $NaBH_4$ (114 mg, 3 mmol) was added portionwise. The mixture was then stirred at room temperature for 6 h. The reaction was quenched with water (3 mL) and extracted with DCM (3 x 10 mL). The combined organic phases were dried over $MgSO_4$ and the solvent evaporated under reduced pressure. The crude was used in the next step without further purification.

Procedure **B** for chalcone reduction: To a stirred solution of the corresponding chalcone (3.5 mmol) and $CeCl_3$ (106 mg, 4.2 mmol) in MeOH (15 mL) at 0 ºC, $NaBH_4$ (161 mg, 4.2 mmol) was added portionwise. The mixture was then stirred at room temperature for 30 min. The reaction was quenched with water (15 mL) and extracted with DCM (3 x 20 mL). The combined organic phases were dried over $MgSO_4$ and the solvent evaporated under reduced pressure. The crude was used in the next step without further purification.

(*E*)-1,3-di-*p*-tolylprop-2-en-1-ol

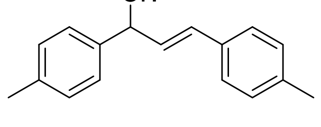

From (*E*)-1,3-di-*p*-tolylprop-2-en-1-one (1.11 g, 4.70 mmol), following the general procedure **A**, (*E*)-1,3-di-p-tolylprop-2-en-1-ol (554 mg, 2.32 mmol) was obtained in 50% yield as a colorless oil. Spectroscopic data are in agreement with the published data.[6] **^{1}H-NMR (300 MHz, $CDCl_3$)** δ 7.37 – 7.28 (m, 4H), 7.22 – 7.08 (m, 4H), 6.65 (d, *J* = 15.7 Hz, 1H),

6.33 (dd, *J* = 15.8, 6.5 Hz, 1H), 5.40 – 5.32 (m, 1H), 2.36 (s, 3H), 2.33 (s, 3H), 1.95 (d, *J* = 3.6 Hz, 1H).

(*E*)-1,3-di-*m*-tolylprop-2-en-1-ol

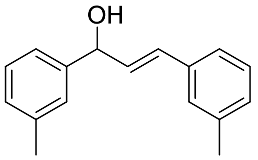

From (*E*)-1,3-di-*m*-tolylprop-2-en-1-one (1.040 g, 4.42 mmol), following the general procedure **B**, (*E*)-1,3-di-*m*-tolylprop-2-en-1-ol (823 g, 3.46 mmol) was obtained in 78% yield as a pale yellow oil. Spectroscopic data are in agreement with the published data.[9] **^{1}H-NMR (300 MHz, CDCl$_3$)** δ 7.30 – 7.05 (m, 8H), 6.67 (dd, *J* = 15.8, 1.2 Hz, 1H), 6.37 (dd, *J* = 15.8, 6.5 Hz, 1H), 5.35 (d, *J* = 6.6 Hz, 1H), 2.37 (s, 3H), 2.33 (s, 3H), 1.99 (s, 1H).

(*E*)-1,3-bis(3,5-dimethoxyphenyl)prop-2-en-1-ol

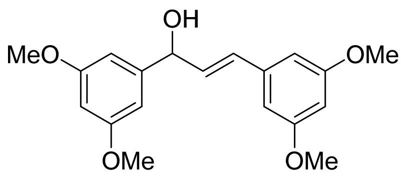

From (*E*)-1,3-bis(3,5-dimethoxyphenyl)prop-2-en-1-one (1.334 g, 4.07 mmol), following the general procedure **B**, (*E*)-1,3-bis(3,5-dimethoxyphenyl)prop-2-en-1-ol (897 mg, 2.72 mmol) was obtained in 67% yield as a white solid. **^{1}H-NMR (300 MHz, CDCl$_3$)** δ 6.62 (d, *J* = 15.8 Hz, 1H), 6.60 (d, *J* = 2.1 Hz, 2H), 6.55 (d, *J* = 2.3 Hz, 2H), 6.40 (t, *J* = 2.3 Hz, 1H), 6.37 (t, *J* = 2.1 Hz, 1H), 6.33 (dd, *J* = 15.8, 6.4 Hz, 1H), 5.33 – 5.30 (m, 1H), 3.80 (s, 6H), 3.79 (s, 6H), 2.04 – 2.00 (m, 1H). **^{13}C-NMR (75 MHz, CDCl$_3$)** δ 161.0 (2C), 160.9 (2C), 145.3, 138.5, 131.7, 130.6, 104.7 (2C), 104.2 (2C), 100.2, 99.7, 75.0, 55.4 (2C), 55.3 (2C). **HRMS (EI$^+$)** calculated for $C_{19}H_{22}O_4$ $[M-O]^+$: 314.1513, found: 314.1518.

(*E*)-1,3-bis(4-fluorophenyl)prop-2-en-1-ol

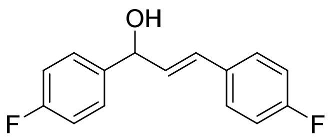

From 1-(4-fluorophenyl)ethan-1-one (927 mg, 3.8 mmol), following the general procedure **A**, (*E*)-1,3-bis(4-fluorophenyl)prop-2-en-1-ol (697 mg, 2.83 mmol) was obtained in 75% yield as a colorless oil. Spectroscopic data are in agreement with the published data.[9] **^{1}H-NMR (300 MHz, CDCl$_3$)** δ 7.42 – 7.32 (m, 4H), 7.10 – 6.95 (m, 4H), 6.64 (d, *J* = 15.8 Hz, 1H), 6.27 (dd, *J* = 15.8, 6.5 Hz, 1H), 5.40 – 5.30 (m, 1H), 1.99 (d, *J* = 3.5 Hz, 1H).

(*E*)-1-(4-fluorophenyl)-3-phenylprop-2-en-1-ol

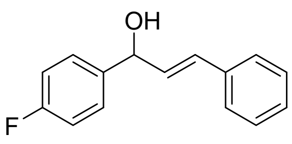

From (*E*)-1-(4-fluorophenyl)-3-phenylprop-2-en-1-one (339 mg, 1.5 mmol), following the general procedure **A**, (*E*)-1-(4-fluorophenyl)-3-phenylprop-2-en-1-ol (314.6 mg, 1.38 mmol) was obtained in 92% yield as a colorless oil. Spectroscopic data are in agreement with the published data.[10] **^{1}H-NMR (300 MHz, CDCl$_3$)** δ 7.45 – 7.23 (m, 7H), 7.10 – 7.03 (m,

2H), 6.69 (d, *J* = 15.8 Hz, 1H), 6.36 (dd, *J* = 15.9, 6.5 Hz, 1H), 5.42 – 5.36 (m, 1H), 2.02 (d, *J* = 3.2 Hz, 1H).

(*E*)-3-(4-fluorophenyl)-1-phenylprop-2-en-1-ol

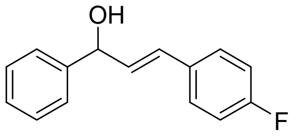

From (*E*)-3-(4-fluorophenyl)-1-phenylprop-2-en-1-one (339 mg mL, 1.5 mmol), following the general procedure **A**, (*E*)-3-(4-fluorophenyl)-1-phenylprop-2-en-1-ol (304.1 mg, 1.34 mmol) was obtained in 89% yield as a colorless oil. Spectroscopic data are in agreement with the published data.[10] **^{1}H NMR (300 MHz, $CDCl_3$)** δ 7.46 – 7.30 (m, 7H), 7.05 – 6.95 (m, 2H), 6.66 (d, *J* = 15.8 Hz, 1H), 6.31 (dd, *J* = 15.9, 6.5 Hz, 1H), 5.42 – 5.36 (m, 1H), 2.02 (d, *J* = 3.5 Hz, 1H).

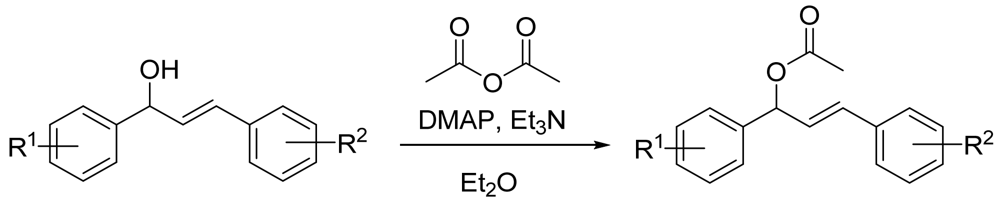

To a vigorously stirred solution of the corresponding alcohol (3 mmol), DMAP (13 mg, 0.097 mmol) and Et_3N (1.31 mL, 9.4 mmol) in Et_2O (7 mL) at 0 ºC, acetic anhydride was added dropwise (0.87 mL, 9.1 mmol). The reaction was stirred at room temperature for 2 h. The reaction mixture was quenched with sat. solution of $NaHCO_3$ (10 mL). The organic phase was separated and the aq. layer extracted with EtOAc (3 x 10 mL). The combined organic phases were dried over $MgSO_4$ and the solvent evaporated under reduced pressure. The crude was used in the next step without further purification.

(*E*)-1,3-di-*p*-tolylallyl acetate (1b)

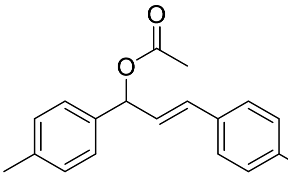

From (*E*)-1,3-di-*p*-tolylprop-2-en-1-ol (520 mg, 2.20 mmol), following the general procedure, (*E*)-1,3-di-*p*-tolylallyl acetate (**1b**) (500 mg, 1.78 mmol) was obtained in 81% yield as a pale yellow oil. Spectroscopic data are in agreement with the published data.[11] **^{1}H-NMR (300 MHz, $CDCl_3$)** δ 7.33 – 7.24 (m, 4H), 7.18 (d, *J* = 7.9 Hz, 2H), 7.10 (d, *J* = 7.7 Hz, 2H), 6.59 (d, *J* = 15.7 Hz, 1H), 6.40 (d, *J* = 6.9 Hz, 1H), 6.29 (dd, *J* = 15.7, 6.8 Hz, 1H), 2.35 (s, 3H), 2.32 (s, 3H), 2.11 (s, 3H).

(*E*)-1,3-di-*m*-tolylallyl acetate (1c)

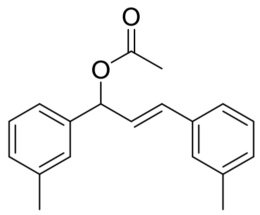

From (*E*)-1,3-di-*m*-tolylprop-2-en-1-ol (823 mg, 3.46 mmol), following the general procedure, (*E*)-1,3-di-*m*-tolylallyl acetate (**1c**) (812 mg, 2.9 mmol) was obtained in 84% yield as a pale yellow oil. Spectroscopic data are in agreement with the published data.[12] **^{1}H-NMR (300 MHz, $CDCl_3$)** δ 7.30 – 7.03 (m, 8H), 6.61 (d, *J* = 15.3 Hz, 1H), 6.41 (d, *J* = 6.9 Hz, 1H), 6.33 (dd, *J* = 15.5, 6.8 Hz, 1H), 2.37 (s, 3H), 2.33 (s, 3H), 2.14 (s, 3H).

(*E*)-1,3-bis(3,5-dimethoxyphenyl)allyl acetate (1d)

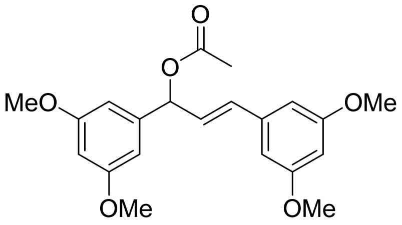

From (*E*)-1,3-bis(3,5-dimethoxyphenyl)prop-2-en-1-ol (897 mg, 2.72 mmol), following the general procedure, (*E*)-1,3-bis(3,5-dimethoxyphenyl)allyl acetate (**1d**) (925 mg, 2.49 mmol) was obtained in 91% yield as a pale yellow oil. **^{1}H-NMR (300 MHz, $CDCl_3$)** δ 6.56 (d, *J* = 15.4 Hz, 1H), 6.55 (d, *J* = 2.3 Hz, 2H), 6.53 (d, *J* = 2.3 Hz, 2H), 6.41 (t, *J* = 2.3 Hz, 1H), 6.37 (t, *J* = 2.3 Hz, 1H), 6.34 (d, *J* = 6.9 Hz, 1H), 6.28 (dd, *J* = 15.3, 6.8 Hz, 1H), 3.80 (s, 6H), 3.78 (s, 6H), 2.14 (s, 3H). **^{13}C-NMR (75 MHz, $CDCl_3$)** δ 169.9, 161.0 (2C), 160.9 (2C), 141.5, 138.2, 132.7, 127.8, 105.0 (2C), 104.8 (2C), 100.5, 99.9, 75.9, 55.39 (2C), 55.36 (2C), 21.3. **HRMS (EI$^+$)** calculated for $C_{19}H_{22}O_4$ $[M-C_2H_3O_2+H]^+$: 314.1513, found: 314.1512.

(*E*)-1,3-bis(4-fluorophenyl)allyl acetate (1e)

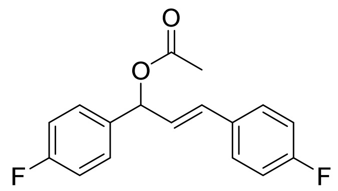

From (*E*)-1,3-bis(4-fluorophenyl)prop-2-en-1-ol (697 mg, 2.8 mmol), following the general procedure, (*E*)-1,3-bis(4-fluorophenyl)allyl acetate (**1e**) (674 mg, 2.34 mmol) was obtained in 84% yield as a pale yellow oil. **^{1}H-NMR (300 MHz, $CDCl_3$)** δ 7.45 – 7.32 (m, 4H), 7.11 – 6.97 (m, 4H), 6.58 (d, *J* = 15.8 Hz, 1H), 6.40 (d, *J* = 6.7 Hz, 1H), 6.24 (dd, *J* = 15.8, 6.7 Hz, 1H), 2.13 (s, 3H). **^{13}C-NMR (75 MHz, $CDCl_3$)** δ 169.9, 162.6 (d, $^1J_{C-F}$ = 247.7 Hz), 162.5 (d, $^1J_{C-F}$ = 247.0 Hz), 135.0 (d, $^4J_{C-F}$ = 3.1 Hz), 132.2 (d, $^4J_{C-F}$ = 3.3 Hz), 131.6, 128.9 (d, $^3J_{C-F}$ = 8.2 Hz, 2C), 128.3 (d, $^3J_{C-F}$ = 8.1 Hz, 2C), 127.1 (d, $^5J_{C-F}$ = 2.3 Hz), 115.6 (d, $^2J_{C-F}$ = 21.7 Hz, 4C), 75.4, 21.3. **^{19}F-NMR (282 MHz, $CDCl_3$)** δ -113.5, -113.7. **HRMS (ESI$^+$)** calculated for $C_{17}H_{14}F_2O_2$ $[M]^+$: 288.0956, found: 288.0950.

(*E*)-1-(4-fluorophenyl)-3-phenylallyl acetate (1j)

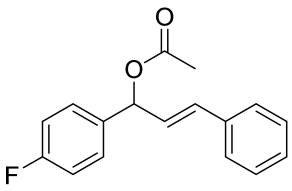

From (*E*)-1-(4-fluorophenyl)-3-phenylprop-2-en-1-ol (314.6 mg, 1.38 mmol), following the general procedure, (*E*)-1-(4-fluorophenyl)-3-phenylallyl acetate (**1j**) (309 mg, 1.14 mmol) was obtained in 83% yield as a pale yellow oil. **^{1}H-NMR (300 MHz, $CDCl_3$)** δ 7.43 – 7.25 (m, 7H), 7.43 – 7.25 (m, 2H), 6.62 (d, J = 15.7 Hz, 1H), 6.42 (d, J = 6.8 Hz, 1H), 6.32 (dd, J = 15.7, 6.6 Hz, 1H), 2.14 (s, 3H). **^{13}C-NMR (75 MHz, $CDCl_3$)** δ 169.9, 162.5 (d, $^1J_{C-F}$ = 246.9 Hz), 136.0, 135.1 (d, $^4J_{C-F}$ = 3.3 Hz), 132.7, 128.9 (d, $^3J_{C-F}$ = 8.3 Hz, 2C), 128.6 (2C), 128.4, 127.2, 126.7 (2C), 115.5 (d, $^2J_{C-F}$ = 21.6 Hz, 2C), 75.4, 21.3. **^{19}F-NMR (282 MHz, $CDCl_3$)** δ -113.8. **HRMS (EI$^+$)** calculated for $C_{17}H_{15}FO_2$ [M]$^+$: 270.1051, found: 270.1042.

(*E*)-3-(4-fluorophenyl)-1-phenylallyl acetate (1k)

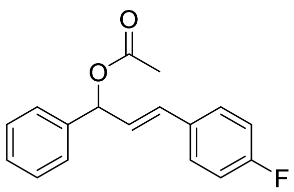

From (*E*)-3-(4-fluorophenyl)-1-phenylprop-2-en-1-ol (304.1 mg, 1.34 mmol) following the general procedure, (*E*)-3-(4-fluorophenyl)-1-phenylallyl acetate (**1k**) (288 mg, 1.07 mmol) was obtained in 80% yield as a colorless oil. **^{1}H-NMR (300 MHz, $CDCl_3$)** δ 7.44 – 7.30 (m, 7H), 7.05 – 6.95 (m, 2H), 6.60 (d, J = 15.7 Hz, 1H), 6.42 (d, J = 6.7 Hz, 1H), 6.26 (dd, J = 15.8, 6.8 Hz, 1H), 2.14 (s, 3H). **^{13}C-NMR (75 MHz, $CDCl_3$)** δ 170.0, 162.6 (d, $^1J_{C-F}$ = 247.6 Hz), 139.1, 132.3 (d, $^4J_{C-F}$ = 3.4 Hz), 131.4, 128.6 (2C), 128.3 (d, $^3J_{C-F}$ = 8.4 Hz, 2C), 128.2, 127.3 (d, $^5J_{C-F}$ = 2.3 Hz), 127.0 (2C), 115.5 (d, $^2J_{C-F}$ = 21.7 Hz, 2C), 76.1, 21.3. **^{19}F-NMR (282 MHz, $CDCl_3$)** δ -113.7. **HRMS (EI$^+$)** calculated for $C_{17}H_{15}FO_2$ [M]$^+$: 270.1051, found: 270.1056.

Synthesis of 1f:

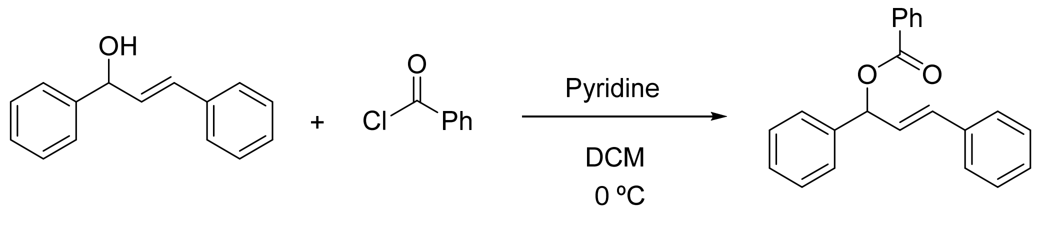

To a stirred solution of (*E*)-1,3-diphenylprop-2-en-1-ol (210 mg, 1 mmol) and pyridine (0.23 mL, 2.8 mmol) in DCM (5 mL) was added benzoyl chloride (0.33 mL, 2.8 mmol) dropwise at 0 ºC, and the mixture was stirred at room temperature for 12 h. The reaction was quenched by the addition of water (5 mL). The aqueous phase was extracted with Et_2O (4 x 8 mL). The combined organic phases were washed successively with 10% HCl (15 mL), sat. $NaHCO_3$ (15 mL), brine (15 mL) and water (15 mL), dried over $MgSO_4$ and the solvent evaporated under reduced pressure. The crude was purified by column chromatography. Eluent: Cyclohexane:AcOEt (9:1).

(*E*)-1,3-diphenylallyl benzoate (1f)

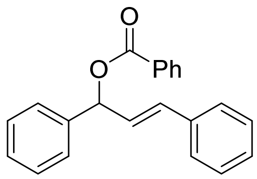

(*E*)-1,3-diphenylallyl benzoate (**1f**) (288 mg, 0.56 mmol) was obtained in 56% yield as a white solid. Spectroscopic data are in agreement with the published data.[13] **^{1}H-NMR (300 MHz, $CDCl_3$)** δ 8.19 – 8.09 (m, 2H), 7.60 – 7.20 (m, 13H), 6.73 (d, *J* = 16.0 Hz, 1H), 6.70 (d, *J* = 6.7 Hz, 1H), 6.47 (dd, *J* = 15.9, 6.7 Hz, 1H).

Synthesis of 1g:

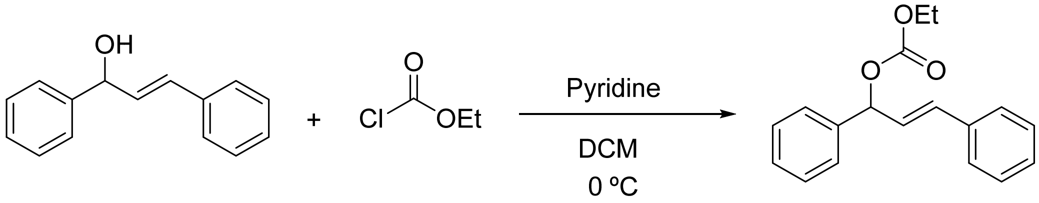

To a stirred solution of (*E*)-1,3-diphenylprop-2-en-1-ol (315 mg, 1.5 mmol) and pyridine (0.13 mL, 1.65 mmol) in DCM (6 mL) was added ethyl chloroformate (0.26 mL, 2.7 mmol) dropwise at 0 ºC. After 3 h stirring at room temperature, 1M HCl (4.5 mL) was added. The mixture was extracted with DCM (3 x 5 mL). The combined organic phases were washed with 1M HCl (8 mL), dried over $MgSO_4$ and the solvent evaporated under reduced pressure. The crude was used in the next step without further purification.

(*E*)-1,3-diphenylallyl ethyl carbonate (1g)

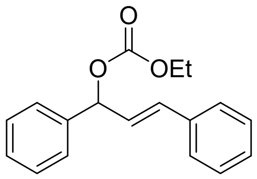

(*E*)-1,3-diphenylallyl ethyl carbonate (**1g**) (257 mg, 0.92 mmol) was obtained in 61% yield as a colorless oil. Spectroscopic data are in agreement with the published data.[14] **^{1}H-NMR (300 MHz, $CDCl_3$)** δ 7.46 – 7.26 (m, 10H), 6.69 (d, *J* = 15.7 Hz, 1H), 6.37 (dd, *J* = 15.7, 6.9 Hz, 1H), 6.26 (d, *J* = 6.9 Hz, 1H), 4.21 (q, *J* = 7.2, Hz, 1H), 4.20 (q, *J* = 7.2, Hz, 1H) 1.31 (t, *J* = 7.2 Hz, 3H).

Synthesis of 1h

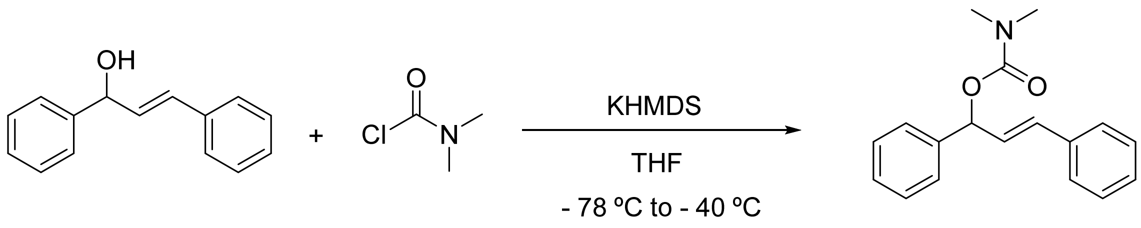

To a stirred solution of (*E*)-1,3-diphenylprop-2-en-1-ol (315 mg, 1.5 mmol) in THF (6 mL) was added dropwise a solution of potassium hexamethyldisilazide (1.83 mL, 1.65 mmol) in THF (1M) at – 78 ºC. After 10 min stirring at – 78 ºC, a solution of dimethyl carbamoyl chloride (0.15 mL, 1.65 mmol) in THF (3 mL) was added. The mixture was then stirred

at – 40 °C during 1 h. The mixture was poured into a mixture of THF (1.5 mL) and acetic acid (1.5 mL). The aqueous phase was extracted with Et_2O (3 x 5 mL). The combined organic phases were washed with water (5 mL) and brine (1.5 mL), dried over $MgSO_4$ and the solvent evaporated under reduced pressure. The crude was used in the next step without further purification.

(*E*)-1,3-diphenylallyl dimethylcarbamate (1h)

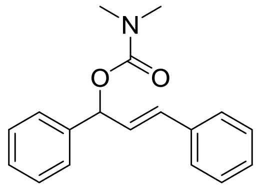

(*E*)-1,3-diphenylallyl dimethylcarbamate (**1h**) (380 mg, 0.92 mmol) was obtained in 90% yield as a yellow oil. **^{1}H-NMR (300 MHz, $CDCl_3$)** δ 7.45 – 7.20 (m, 10H), 6.67 – 6.58 (m, 1H), 6.46 – 6.30 (m, 2H), 3.02 (s, 3H), 2.94 (s, 3H). **^{13}C-NMR (75 MHz, $CDCl_3$)** δ 175.4, 155.7, 140.0, 136.4, 132.0, 128.51 (2C), 128.48 (2C), 128.45, 127.9, 126.9 (2C), 126.7 (2C), 76.96, 36.5, 35.9. **HRMS (EI$^+$)** calculated for $C_{18}H_{19}NO_2^+$ $[M]^+$: 281.1410, found: 281.1470.

General procedure for the preparation of *N*-phenyl phenothiazine catalyst (3e)

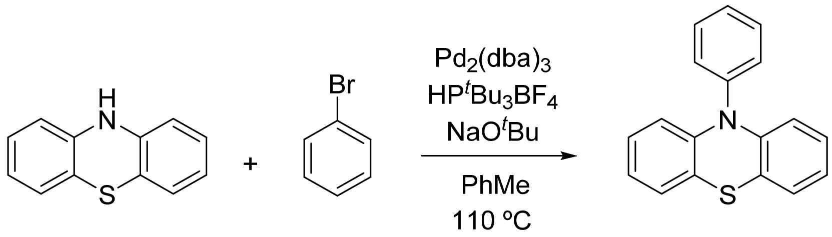

Phenothiazine (1.99 g, 10 mmol) and 1-bromobenzene (1 mL, 11 mmol) were dissolved in toluene (10 mL). $Pd_2(dba)_3$ (275 mg) and $HP^tBu_3BF_4$ (145 mg) were added and the mixture was stirred for 10 min under N_2 atmosphere. NaO^tBu (1.11 g) was added and the mixture stirred under N_2 atmosphere at 110 °C for 48 h. After cooling to room temperature, the resulting mixture was diluted with DCM (20 mL) and filtered through a pad of celite®. The celite pad was washed with DCM (3 x 10 mL). The solvent was evaporated under reduced pressure and the crude purified by column chromatography. Eluent: Cyclohexane:AcOEt (98:2). White solid (2.477 g, 9 mmol, 90% yield). Spectroscopic data are in agreement with the published data.[15] **^{1}H-NMR (300 MHz, $CDCl_3$)** δ 7.65 – 7.55 (m, 2H), 7.50 – 7.45 (m, 1H), 7.42 – 7.35 (m, 2H), 7.05 – 7.00 (m, 2H), 6.90 – 6.75 (m, 4H), 6.25 – 6.15 (m, 2H).

General procedure for the preparation of 3-(4-methoxyphenyl)-10-phenyl-10*H*-phenoxazine catalyst (3g)

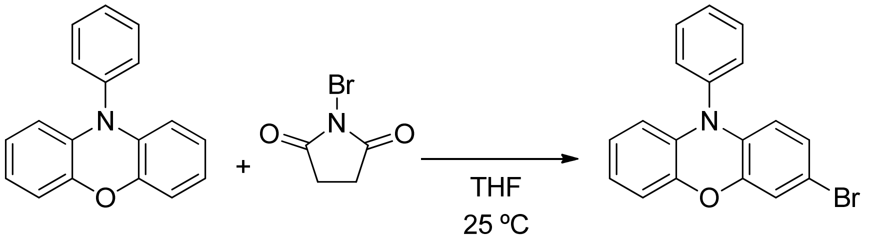

Phenyl-10-phenoxazine (1.0 g, 3.86 mmol) was dissolved in THF (168 mL) and the flask was covered in aluminum foil. *N*-bromosuccinimide (0.706 g, 3.97 mmol) was added portionwise over thirty minutes. The reaction was stirred at room temperature until disappearance of the phenyl-10-phenoxazine followed by TLC. Then, THF was removed under reduced pressure. The reaction was re-dissolved in DCM, washed with de-ionized water once and brine twice, dried over magnesium sulfate and concentrated under reduced pressure. The crude product was collected as a red oil and the crude purified by column chromatography. Eluent: Cyclohexane:DCM (15:1). White solid (2.477 g, 9 mmol, 90% yield). Spectroscopic data are in agreement with the published data.[16] **^{1}H-NMR (300 MHz, $CDCl_3$)** δ 7.69 – 7.60 (m, 2H), 7.58 – 7.48 (m, 1H), 7.40 – 7.30 (m, 2H), 6.85 (ddd, *J* = 4.7, 2.2, 0.9 Hz, 1H), 6.77 – 6.60 (m, 4H), 6.00 – 5.93 (m, 1H), 5.86 – 5.77 (m, 1H).

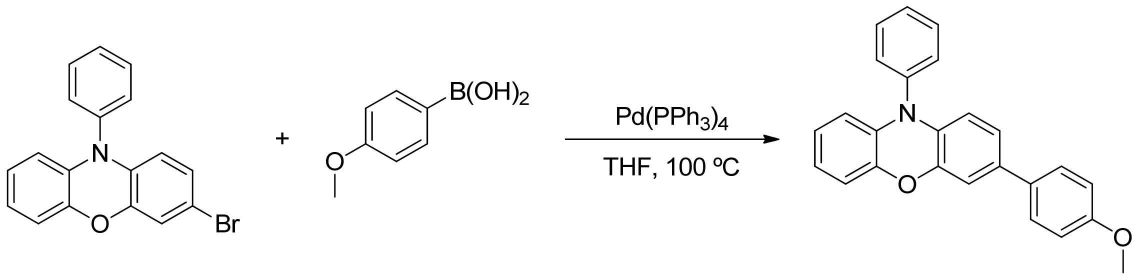

3-Bromophenyl-10-phenoxazine (0.373 g, 1.1 mmol) and 4-methoxyphenyl boronic acid (0.334 g, 2.2 mmol) were added to a storage tube and cycled between vacuum and nitrogen three times before dried and degassed THF (8.00 mL) was added. Once all reagents were dissolved, a 2.00 M aqueous solution of K_2CO_3 (8.00 mL), which had been sparged with nitrogen, was added. In a separate Schlenk flask, tetrakis(triphenylphosphine) palladium (0.102 g, 0.088 mmol) was dissolved in THF (8.00 mL) under inert atmosphere. The solution of $Pd(PPh_3)_4$ was then added to the reaction mixture and the reaction was heated at 100 °C for 48 h, before it was exposed to oxygen and allowed to cool to room temperature. The reaction mixture was concentrated under reduced pressure, diluted with DCM/hexanes, and passed through a short plug of silica. The solution was then moved to a separatory funnel, washed with de-ionized water once and brine twice. The solution was dried over magnesium sulfate, concentrated under

vacuum, and recrystallized using DCM/methanol at -25 °C. The product was collected via vacuum filtration as a white solid (0.162 g, 0.444 mmol, 40% yield). Spectroscopic data are in agreement with the published data.[16] **^{1}H-NMR (300 MHz, C_6D_6)** δ 7.48 – 7.37 (m, 2H), 7.24 – 7.18 (m, 3H), 7.17 – 6.99 (m, 3H), 6.97 – 6.77 (m, 4H), 6.57 (td, *J* = 7.6, 1.6 Hz, 2H), 6.17 – 6.02 (m, 2H), 3.39 (s, 3H).

General procedure for the preparation of *Z*-allylic compounds (4)

A vial equipped with a magnetic stir bar and fitted with a teflon screw cap septum was charged with the corresponding allylic compound **1** (0.1 mmol), the corresponding heterocycle **2** (0.2 mmol), *N*-phenyl phenothiazine (1.4 mg, 5 mol%), DIPEA (86 μL, 0.5 mmol) and acetonitrile (1 mL). The reaction was degassed with three freeze-pump-thaw cycles. The vial was then backfilled with N_2 and stirred under 365 nm LEDs irradiation (8.2460 W/m^2 intensity; approximate distance was 2 cm from the vial) at 20 ºC. After 3 h the vial was opened, the solvent evaporated and the crude purified by column chromatography.

(*Z*)-2-(1,3-diphenylallyl)-1*H*-pyrrole (4a)

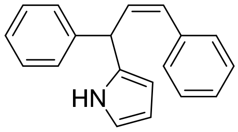

4a

From (*E*)-1,3-diphenylallyl acetate (25.2 mg, 0.1 mmol) and pyrrole (13.9 μL, 0.2 mmol), following the general procedure, compound **4a** (15.0 mg, 0.058 mmol) was obtained in 58% yield (*Z*:*E* ratio = 94:6) as a brownish oil. The crude product was purified by column chromatography. Eluent: Pentane:AcOEt (95:5). **^{1}H-NMR (300 MHz, $CDCl_3$)** δ 7.78 (br s, 1H), 7.36 – 7.21 (m, 10H), 6.71 – 6.67 (m, 1H), 6.66 (d, *J* = 11.5 Hz, 1H), 6.16 (m, 1H), 6.05 (dd, *J* = 11.4, 10.3 Hz, 1H), 6.01 – 5.98 (m, 1H), 5.18 (d, *J* = 10.3 Hz, 1H). **^{13}C-NMR (75 MHz, $CDCl_3$)** δ 142.9, 136.7, 133.6, 132.6, 129.6, 128.8 (2C), 128.7 (2C), 128.3 (2C), 128.1 (2C), 127.1, 126.8, 117.1, 108.4, 106.5, 43.3. **HRMS (EI$^+$)** calculated for $C_{19}H_{17}N$ [M]$^+$: 259.1356, found: 259.1375.

(*Z*)-2-(1,3-diphenylallyl)-1-methyl-1*H*-pyrrole (4b)

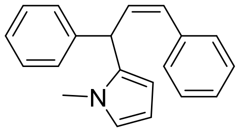

4b

From (*E*)-1,3-diphenylallyl acetate (25.2 mg, 0.1 mmol) and 1-methylpyrrole (16.2 μL, 0.2 mmol), following the general procedure, compound **4b** (19.2 mg, 0.070 mmol) was obtained in 70% yield (*Z*:*E* ratio = 92:8) as an orange oil. The crude product was purified by column chromatography. Eluent: Hexane:DCM (2:1). **^{1}H-NMR (300 MHz, $CDCl_3$)** δ 7.35 – 7.20 (m, 8H), 7.18 – 7.10 (m, 2H), 6.65 – 6.54 (m, 2H), 6.14 - 6.09 (m, 1H), 6.06 – 5.95 (m, 2H), 5.13 (d, *J* = 10.1 Hz, 1H), 3.24 (s, 3H). **^{13}C-NMR (75 MHz, $CDCl_3$)** δ 143.2, 137.0, 134.2, 133.2, 128.7 (2C), 128.6 (2C), 128.5, 128.3 (2C), 127.8 (2C), 127.0,

126.5, 122.0, 107.3, 106.6, 42.3, 33.9. **HRMS (EI$^+$)** calculated for $C_{20}H_{19}N$ $[M]^+$: 273.1512, found: 273.1505.

(*Z*)-2-(1,3-diphenylallyl)-1-phenyl-1*H*-pyrrole (4c)

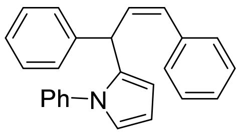

4c

From (*E*)-1,3-diphenylallyl acetate (25.2 mg, 0.1 mmol) and 1-phenylpyrrole (28.6 mg, 0.2 mmol), following the general procedure, compound **4c** (18.3 mg, 0.055 mmol) was obtained in 55% yield (*Z*:*E* ratio = 75:25) as a yellow oil. The crude product was purified by column chromatography. Eluent: Hexane:DCM (5:1). *Z*-isomer: **^{1}H-NMR (300 MHz, $CDCl_3$)** δ 7.40 – 7.05 (m, 12H), 7.03 – 6.95 (m, 3H), 6.79 – 6.74 (m, 1H), 6.50 (d, *J* = 11.3 Hz, 1H), 6.28 (m, 1H), 6.24 – 6.20 (m, 1H), 6.01 (dd, *J* = 11.3, 10.2 Hz, 1H), 5.13 (d, *J* = 10.2 Hz, 1H). **^{13}C-NMR (75 MHz, $CDCl_3$)** δ 143.9, 139.9, 136.8, 135.3, 133.6, 129.4, 128.7 (2C), 128.5 (2C), 128.3 (2C), 128.1 (2C), 127.7 (2C), 127.1, 126.8, 126.5 (2C), 126.2, 122.1, 108.2, 108.0, 41.8. **HRMS (EI$^+$)** calculated for $C_{25}H_{21}N$ $[M]^+$: 335.1669, found: 335.1642.

E-isomer: **^{1}H-NMR (300 MHz, $CDCl_3$)** δ 7.35 – 7.04 (m, 4.5H), 6.79 – 6.76 (m, 0.3H), 6.52 (dd, *J* = 15.8, 7.0 Hz, 0.3H), 6.29 – 6.23 (m, 0.3H), 6.17 – 6.09 (m, 0.6H), 4.76 (d, *J* = 7.0 Hz, 0.3H). **^{13}C-NMR (75 MHz, $CDCl_3$)** δ 142.5, 140.1, 137.3, 134.6, 132.0, 130.7, 128.8 (2C), 128.41 (2C), 128.36 (2C), 128.2 (2C), 127.4, 127.2, 126.9 (2C), 126.33, 126.29 (2C), 122.4, 108.6, 108.0, 46.3.

(*Z*)-2-(1,3-diphenylallyl)-*N*,*N*-dimethyl-1*H*-pyrrol-1-amine (4d)

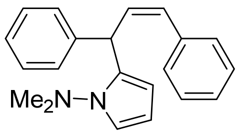

4d

From (*E*)-1,3-diphenylallyl acetate (25.2 mg, 0.1 mmol) and 1-(dimethylamino)pyrrole (24.2 μL, 0.2 mmol), following the general procedure, compound **4d** (14 mg, 0.046 mmol) was obtained in 46% yield (*Z*:*E* ratio = 96:4) as a colorless oil. The crude product was purified by column chromatography. Eluent: Hexane:DCM (5:1). **^{1}H-NMR (300 MHz, $CDCl_3$)** δ 7.35 – 7.20 (m, 7H), 7.18 – 7.10 (m, 3H), 6.88 (dd, *J* = 3.1, 1.8 Hz, 1H), 6.56 (d, *J* = 11.3 Hz, 1H), 6.15 (t, *J* = 3.4 Hz, 1H), 6.08 (t, *J* = 11.0 Hz, 1H), 5.92 (dd, *J* = 3.8, 1.8 Hz, 1H), 5.44 (d, *J* = 10.5 Hz, 1H), 2.47 (br s, 6H). **^{13}C-NMR (75 MHz, $CDCl_3$)** δ 144.3, 137.3, 134.2, 133.6, 128.8 (2C), 128.4, 128.2 (2C), 128.0 (2C), 127.8 (2C), 126.7, 125.9, 111.9, 106.4, 102.8, 47.5 (2C), 41.3. **HRMS (EI$^+$)** calculated for $C_{19}H_{16}N$ $[M-C_2H_6N]^+$: 258.1277, found: 258.1266.

(*Z*)-3-(1,3-diphenylallyl)-1*H*-indole (4e)

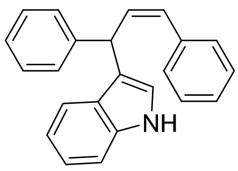

4e

From (*E*)-1,3-diphenylallyl acetate (25.2 mg, 0.1 mmol) and indole (23.4 mg, 0.2 mmol), following the general procedure, compound **4e** (20.7 mg, 0.066 mmol) was obtained in 66% yield (*Z*:*E* ratio = 91:9) as a colorless oil. The crude product was purified by column chromatography. Eluent: Hexane:DCM (1:1). **^{1}H-NMR (300 MHz, CDCl$_3$)** δ 7.95 (br s, 1H), 7.38 – 7.12 (m, 13H), 7.04 – 6.93 (m, 2H), 6.63 (d, *J* = 11.4 Hz, 1H), 6.14 (dd, *J* = 11.3, 10.3 Hz, 1H), 5.42 (d, *J* = 10.2 Hz, 1H). **^{13}C-NMR (75 MHz, CDCl$_3$)** δ 144.2, 137.1, 136.8, 134.4, 128.7 (2C), 128.5 (2C), 128.4, 128.3 (2C), 128.1 (2C), 126.9, 126.6, 126.3, 122.3, 122.1, 120.0, 119.3, 119.0, 111.1, 41.8. **HRMS (EI$^+$)** calculated for $C_{23}H_{19}N$ [M]$^+$: 309.1512, found: 309.1501.

(*Z*)-3-(1,3-diphenylallyl)-1-methyl-indole (4f)

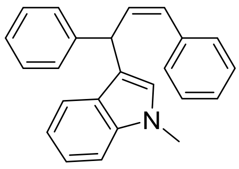

4f

From (*E*)-1,3-diphenylallyl acetate (25.2 mg, 0.1 mmol) and *N*-methylindole (26.2 μL, 0.2 mmol), following the general procedure, compound **4f** (25.3 mg, 0.077 mmol) was obtained in 77% yield (*Z*:*E* ratio = 89:11) as a colorless oil. The crude product was purified by column chromatography. Eluent: Hexane:DCM (3:1). **^{1}H-NMR (300 MHz, CDCl$_3$)** δ 7.32 – 7.15 (m, 13H), 6.99 – 6.91 (m, 1H), 6.87 (s, 1H), 6.62 (d, *J* = 11.4 Hz, 1H), 6.13 (dd, *J* = 11.4, 10.3 Hz, 1H), 5.42 (d, *J* = 10.3 Hz, 1H), 3.75 (s, 3H). **^{13}C-NMR (75 MHz, CDCl$_3$)** δ 144.4, 137.5, 137.1, 134.6, 128.8 (2C), 128.5 (2C), 128.30, 128.28 (2C), 128.1 (2C), 127.01, 126.97, 126.9, 126.2, 121.6, 120.0, 118.7, 117.4, 109.2, 41.7, 32.7. **HRMS (EI$^+$)** calculated for $C_{24}H_{21}N$ [M]$^+$: 323.1669, found: 323.1664.

(*Z*)-3-(1,3-diphenylallyl)-5-methoxy-1*H*-indole (4g)

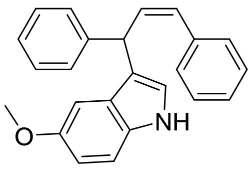

4g

From (*E*)-1,3-diphenylallyl acetate (25.2 mg, 0.1 mmol) and 5-methoxyindole (29.4 mg, 0.2 mmol), following the general procedure, compound **4g** (18.6 mg, 0.055 mmol) was obtained in 55% yield (*Z*:*E* ratio = 94:6) as a colorless oil. The crude product was purified by column chromatography. Eluent: Hexane:DCM (1:2). **^{1}H-NMR (300 MHz, CDCl$_3$)** δ 7.88 (br s, 1H), 7.35 – 7.20 (m, 11H), 6.95 (d, *J* = 2.6 Hz, 1H), 6.85 – 6.77 (m, 1H), 6.64 (d, *J* = 11.4 Hz, 1H), 6.58 (d, *J* = 2.4 Hz, 1H), 6.13 (dd, *J* = 11.1, 10.5 Hz, 1H), 5.39 (d, *J* = 10.4 Hz, 1H), 3.62 (s, 3H). **^{13}C-NMR (75 MHz, CDCl$_3$)** δ 153.7, 144.0, 137.1, 134.4, 131.8, 128.8 (2C), 128.5 (2C), 128.4, 128.3 (2C), 128.1 (2C), 127.0, 126.9, 126.3, 122.9, 118.9, 112.3, 111.7, 101.6, 55.6, 41.7. **HRMS (EI$^+$)** calculated for $C_{24}H_{21}NO$ [M]$^+$: 339.1618, found: 339.1604.

(*Z*)-3-(1,3-diphenylallyl)-6-methoxy-1*H*-indole (4h)

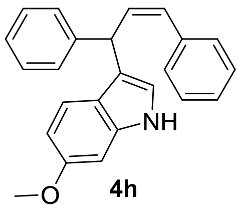

4h

From (*E*)-1,3-diphenylallyl acetate (25.2 mg, 0.1 mmol) and 6-methoxyindole (29.4 mg, 0.2 mmol), following the general procedure, compound **4h** (17.0 mg, 0.050 mmol) was obtained in 50% yield (*Z*:*E* ratio = 92:8) as a brownish oil. The crude product was purified by column chromatography. Eluent: Hexane:DCM (1:3). **^{1}H-NMR (300 MHz, CDCl$_3$)** δ 7.87 (br s, 1H), 7.36 – 7.24 (m, 10H), 7.03 (d, *J* = 8.7 Hz, 1H), 6.91 (dd, *J* = 2.4, 1.1 Hz, 1H), 6.85 (d, *J* = 2.3 Hz, 1H), 6.63 (dd, *J* = 8.7, 2.3 Hz, 1H), 6.62 (d, *J* = 11.3 Hz, 1H), 6.12 (dd, *J* = 11.4, 10.2 Hz, 1H), 5.37 (d, *J* = 10.3 Hz, 1H), 3.81 (s, 3H). **^{13}C-NMR (75 MHz, CDCl$_3$)** δ 156.5, 144.2, 137.6, 137.1, 134.5, 128.7 (2C), 128.5 (2C), 128.4, 128.3 (2C), 128.1 (2C), 126.9, 126.3, 121.1, 121.0, 120.5, 119.0, 109.2, 94.7, 55.6, 41.9. **HRMS (EI$^+$)** calculated for $C_{24}H_{21}NO$ [M]$^+$: 339.1618, found: 339.1616.

(*Z*)-3-(1,3-diphenylallyl)-5,6-dimethoxy-1*H*-indole (4i)

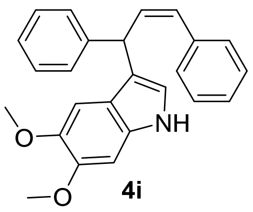

4i

From (*E*)-1,3-diphenylallyl acetate (25.2 mg, 0.1 mmol) and 5,6-dimethoxyindole (35.4 mg, 0.2 mmol), following the general procedure, compound **4i** (6.6 mg, 0.018 mmol) was obtained in 18% yield (*Z*:*E* ratio = 91:9) as a yellow oil. The crude product was purified by column chromatography. Eluent: Hexane:DCM (1:1). **^{1}H-NMR (300 MHz, CDCl$_3$)** δ 7.82 (br s, 1H), 7.36 – 7.23 (m, 10H), 6.86 (s, 1H), 6.84 (dd, *J* = 2.4, 1.1 Hz, 1H), 6.64 (d, *J* = 11.4 Hz, 1H), 6.52 (s, 1H), 6.12 (dd, *J* = 11.4, 10.3 Hz, 1H), 5.37 (d, *J* = 10.4 Hz, 1H), 3.88 (s, 3H), 3.65 (s, 3H). **^{13}C-NMR (75 MHz, CDCl$_3$)** δ 147.1, 144.7, 144.0, 137.2, 134.5, 130.9, 128.8 (2C), 128.5 (2C), 128.33, 128.29 (2C), 128.1 (2C), 126.9, 126.3, 120.7, 119.5, 119.1, 101.5, 94.5, 56.2, 56.1, 41.7. **HRMS (EI$^+$)** calculated for $C_{25}H_{23}NO_2$ [M]$^+$: 369.1723, found: 369.1707.

The C2-allylated compound was obtained as a side-product:

(*Z*)-2-(1,3-diphenylallyl)-5,6-dimethoxy-1*H*-indole (4i')

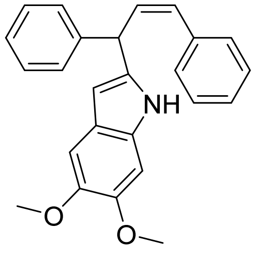

4i'

Compound **4i'** (9.3 mg, 0.027 mmol) was obtained in 25% yield as a yellow oil. The crude product was purified by column chromatography. Eluent: Hexane:DCM (1:1). **^{1}H-NMR (300 MHz, CDCl$_3$)** δ 7.59 (br s, 1H), 7.34 – 7.22 (m, 10H), 7.02 (s, 1H), 6.78 (s, 1H), 6.71 (d, *J* = 11.3 Hz, 1H), 6.29 – 6.25 (m, 1H), 6.10 (dd, *J* = 11.3, 10.1 Hz, 1H), 5.28 (d, *J* = 10.2 Hz, 1H), 3.90 (s, 3H), 3.86 (s, 3H). **^{13}C-NMR (75 MHz, CDCl$_3$)** δ 146.6, 145.1, 142.4, 139.1, 136.6, 132.0, 130.5, 130.1, 128.9 (2C), 128.7 (2C), 128.4 (2C), 128.2 (2C), 127.2, 127.0, 121.2, 102.3, 100.9,

94.5, 56.4, 56.2, 44.0. **HRMS (ESI$^+$)** calculated for $C_{25}H_{23}NO_2$ [M]$^+$: 369.1723, found: 369.1707.

(*Z*)-3-(1,3-diphenylallyl)-5-(trifluoromethyl)-1*H*-indole (4j)

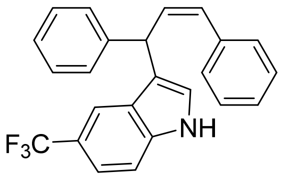

4j

From (*E*)-1,3-diphenylallyl acetate (25.2 mg, 0.1 mmol) and 5-(trifluoromethyl)indole (37.0 mg, 0.2 mmol), following the general procedure, compound **4j** (25.2 mg, 0.066 mmol) was obtained in 66% yield (*Z*:*E* ratio = 94:6) as an orange oil. The crude product was purified by column chromatography. Eluent: Hexane:DCM (3:1). **^{1}H-NMR (300 MHz, CDCl$_3$)** δ 8.15 (br s, 1H), 7.47 (s, 1H), 7.41 – 7.37 (m, 2H), 7.34 – 7.22 (m, 10H), 7.04 (dd, *J* = 2.5, 1.2 Hz, 1H), 6.67 (d, *J* = 11.2 Hz, 1H), 6.12 (dd, *J* = 11.4, 10.3 Hz, 1H), 5.41 (d, *J* = 10.4 Hz, 1H). **^{13}C-NMR (75 MHz, CDCl$_3$)** δ 143.5, 138.0, 136.9, 133.8 (2C), 129.0, 128.7 (2C), 128.4 (2C), 128.0 (2C), 127.1, 126.6, 126.3, 126.0, 125.8 (q, $^1J_{C\text{-}F}$ = 231.6 Hz), 123.8, 121.8, (q, $^2J_{C\text{-}F}$ = 31.8 Hz), 120.4, 118.9 (q, $^3J_{C\text{-}F}$ = 3.3 Hz), 117.6 (d, $^3J_{C\text{-}F}$ = 4.2 Hz), 111.3, 41.4. **^{19}F-NMR (282 MHz, CDCl$_3$)** δ -60.3. **HRMS (EI$^+$)** calculated for $C_{24}H_{18}NF_3$ [M]$^+$: 387.0617, found: 387.0618.

(*Z*)-3-(1,3-diphenylallyl)-7-methyl-1*H*-indole (4k)

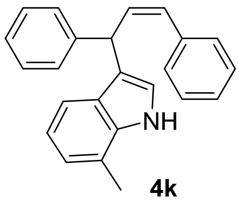

4k

From (*E*)-1,3-diphenylallyl acetate (25.2 mg, 0.1 mmol) and 7-methylindole (39.2 mg, 0.2 mmol), following the general procedure, compound **4k** (26.2 mg, 0.070 mmol) was obtained in 70% yield (*Z*:*E* ratio = 91:9) as a colorless oil. The crude product was purified by column chromatography. Eluent: Hexane:DCM (3:1). **^{1}H-NMR (300 MHz, CDCl$_3$)** δ 7.87 (br s, 1H), 7.35 – 7.20 (m, 10H), 7.08 – 7.00 (m, 2H), 6.96 (d, *J* = 6.5 Hz, 1H), 6.90 (d, *J* = 7.4 Hz, 1H), 6.62 (d, *J* = 11.4 Hz, 1H), 6.13 (dd, *J* = 11.5, 10.2 Hz, 1H), 5.42 (d, *J* = 10.1 Hz, 1H), 2.46 (s, 3H). **^{13}C-NMR (75 MHz, CDCl$_3$)** δ 144.3, 137.1, 136.4, 134.5, 128.7 (2C), 128.5 (2C), 128.4, 128.3 (2C), 128.1 (2C), 126.9, 126.2, 126.1, 122.6, 122.0, 120.2, 119.53, 119.45, 117.7, 41.9, 16.6. **HRMS (EI$^+$)** calculated for $C_{23}H_{18}N$ [M-CH$_3$]$^+$: 308.1434, found: 309.1403.

(*Z*)-3-(1,3-diphenylallyl)-4-methyl-1*H*-indole (4l)

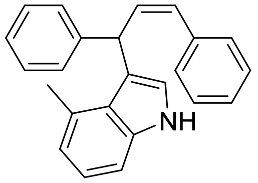

4l

From (*E*)-1,3-diphenylallyl acetate (25.2 mg, 0.1 mmol) and 4-methylindole (26.2 μL, 0.2 mmol), following the general procedure, compound **4l** (25.1 mg, 0.078 mmol) was obtained in 78% yield (*Z*:*E* ratio = 90:10) as a brownish oil. The crude product was purified by column chromatography. Eluent: Hexane:DCM (2:1). **^{1}H-NMR (300 MHz, CDCl$_3$)** δ 8.08 (br s, 1H), 7.35 – 7.20 (m, 9H), 7.15 – 7.10 (m, 3H), 7.09 – 7.03 (m, 1H), 6.76 (d, *J* = 7.1 Hz, 1H), 6.57 (d, *J* = 11.3 Hz, 1H), 6.08 (dd, *J* =

11.1, 10.3 Hz, 1H), 5.75 (d, *J* = 10.1 Hz, 1H), 2.25 (s, 3H). **^{13}C-NMR (75 MHz, CDCl$_3$)** δ 146.0, 137.1, 135.7, 131.3, 128.8 (2C), 128.4 (2C), 128.33, 128.27 (2C), 128.0 (2C), 127.6, 126.9, 126.3, 126.1, 122.8, 122.2, 121.2, 119.4, 109.0, 42.3, 20.3. **HRMS (EI$^+$)** calculated for $C_{24}H_{21}N$ [M]$^+$: 323.1669, found: 309.1675.

(*Z*)-5-bromo-3-(1,3-diphenylallyl)-1*H*-indole (4m)

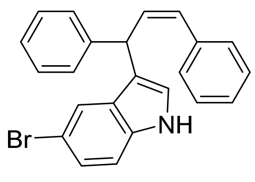

4m

From (*E*)-1,3-diphenylallyl acetate (25.2 mg, 0.1 mmol) and 5-bromoindole (39.2 mg, 0.2 mmol), following the general procedure, compound **4m** (20.3 mg, 0.053 mmol) was obtained in 53% yield (*Z*:*E* ratio = 88:12) as a brownish oil. The crude product was purified by column chromatography. Eluent: Hexane:DCM (2:1). **^{1}H-NMR (300 MHz, CDCl$_3$)** δ 8.00 (br s, 1H), 7.32 – 7.19 (m, 13H), 6.98 (d, *J* = 2.5 Hz, 1H), 6.64 (d, *J* = 11.4 Hz, 1H), 6.10 (dd, *J* = 11.4, 10.3 Hz, 1H), 5.35 (d, *J* = 10.3 Hz, 1H). **^{13}C-NMR (75 MHz, CDCl$_3$)** δ 143.7, 136.9, 135.4, 133.9, 128.8, 128.7 (2C), 128.6 (2C), 128.4 (2C), 128.3, 127.9 (2C), 127.1, 126.5, 125.0, 123.4, 122.5, 118.9, 112.7, 122.5, 41.5. **HRMS (EI$^+$)** calculated for $C_{23}H_{18}NBr$ [M]$^+$: 387.0617, found: 387.0618.

(*Z*)-2-(1,3-di-*p*-tolylallyl)-1*H*-pyrrole (4n)

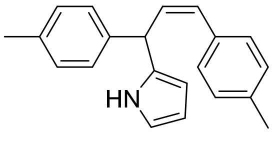

4n

From **1b** (28 mg, 0.1 mmol) and pyrrole (14 μL, 0.2 mmol), following the general procedure, compound **4n** (15 mg, 0.052 mmol) was obtained in 52% yield (*Z*:*E* ratio = 95:5) as a brownish oil. The crude product was purified by column chromatography. Eluent: Pentane:AcOEt (98:2). **^{1}H-NMR (300 MHz, CDCl$_3$)** δ 7.80 (br s, 1H), 7.24 – 7.12 (m, 8H), 6.70 – 6.67 (m, 1H), 6.62 (d, *J* = 11.3 Hz, 1H), 6.19 – 6.16 (m, 1H), 6.04 – 5.96 (m, 2H), 5.16 (d, *J* = 10.3 Hz, 1H), 2.36 (s, 3H), 2.35 (s, 3H). **^{13}C-NMR (75 MHz, CDCl$_3$)** δ 140.0, 136.8, 136.4, 133.9, 132.2, 129.5 (2C), 129.3, 129.0 (2C), 128.6 (2C), 128.0 (2C), 126.6, 117.0, 108.4, 106.3, 43.0, 21.2, 21.0. **HRMS (EI$^+$)** calculated for $C_{21}H_{21}N$ [M]$^+$: 287.1669, found: 287.1670.

(*Z*)-2-(1,3-di-*m*-tolylallyl)-1*H*-pyrrole (4o)

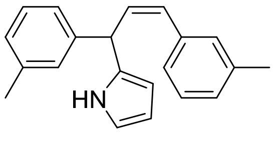

4o

From **1c** (28.0 mg, 0.1 mmol) and pyrrole (14 μL, 0.2 mmol), following the general procedure, compound **4o** (13 mg, 0.045 mmol) was obtained in 45% yield (*Z*:*E* ratio = 91:9) as a pale yellow oil. The crude product was purified by column chromatography. Eluent: Pentane:AcOEt (98:2). **^{1}H-NMR (300 MHz, CDCl$_3$)** δ 7.79 (br s, 1H), 7.25 – 7.18 (m, 2H), 7.13 – 7.01 (m, 6H), 6.69 – 6.67 (m, 1H), 6.62 (d, *J* = 11.4 Hz, 1H), 6.18 – 6.15 (m, 1H), 6.05 (dd, *J* = 11.4, 10.3 Hz, 1H), 6.00 – 5.96 (m, 1H), 5.14 (d, *J* = 10.3 Hz, 1H), 2.33 (s, 6H). **^{13}C-NMR (75 MHz, CDCl$_3$)** δ 142.9, 138.4, 137.8,

136.7, 133.8, 132.6, 129.6, 129.5, 128.8, 128.7, 128.2, 127.8, 127.6, 125.7, 125.2, 117.0, 108.4, 106.3, 43.3, 21.5, 21.4. **HRMS (EI$^+$)** calculated for $C_{21}H_{21}N$ [M]$^+$: 287.1669, found: 287.1664.

(*Z*)-2-(1,3-bis(4-methoxyphenyl)allyl)-1*H*-pyrrole (4p)

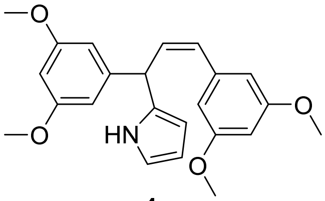

4p

From **1d** (37.2 mg, 0.1 mmol) and pyrrole (14 μL, 0.2 mmol), following the general procedure, compound **4p** (20.3 mg, 0.054 mmol) was obtained in 54% yield (*Z*:*E* ratio = 100:0) as a pale yellow oil. The crude product was purified by column chromatography. Eluent: Pentane:AcOEt (85:15). **^{1}H-NMR (300 MHz, CDCl$_3$)** δ 7.84 (br s, 1H), 6.70 – 6.65 (m, 1H), 6.60 (d, *J* = 11.3 Hz, 1H), 6.45 (d, *J* = 2.3 Hz, 2H), 6.42 (d, *J* = 2.3 Hz, 1H), 6.38 (t, *J* = 2.3 Hz, 1H), 6.36 (t, *J* = 2.3 Hz, 2H), 6.15 (q, *J* = 2.9 Hz, 1H), 6.05 (dd, *J* = 11.3, 10.1 Hz, 1H), 6.04 – 6.00 (m, 1H), 5.11 (d, *J* = 10.0 Hz, 1H), 3.76 (s, 6H), 3.72 (s, 6H). **^{13}C-NMR (75 MHz, CDCl$_3$)** δ 161.1 (2C), 160.6 (2C), 145.3, 138.6, 133.2, 132.7, 129.8, 117.1, 108.4, 106.7 (2C), 106.4, 106.2 (2C), 99.7, 98.8, 55.30 (2C), 55.25 (2C), 43.7. **HRMS (EI$^+$)** calculated for $C_{23}H_{25}NO_4$ [M]$^+$: 379.1778, found: 379.1777.

(*Z*)-2-(1,3-bis(4-fluorophenyl)allyl)-1*H*-pyrrole (4q)

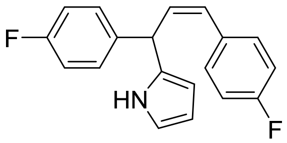

4q

From **1e** (28.8 mg, 0.1 mmol) and pyrrole (14 μL, 0.2 mmol), following the general procedure, compound **4q** (15.0 mg, 0.051 mmol) was obtained in 51% yield (*Z*:*E* ratio = 94:6) as a colorless oil. The crude product was purified by column chromatography. Eluent: Pentane:AcOEt (98:2). **^{1}H-NMR (300 MHz, CDCl$_3$)** δ 7.80 (br s, 1H), 7.26 – 7.14 (m, 4H), 7.07 – 6.98 (m, 4H), 6.74 – 6.70 (m, 1H), 6.62 (d, *J* = 11.3 Hz, 1H), 6.20 – 6.16 (m, 1H), 6.03 – 5.96 (m, 2H), 5.10 (d, *J* = 10.2 Hz, 1H). **^{13}C-NMR (75 MHz, CDCl$_3$)** δ 162.0 (d, $^1J_{C\text{-}F}$ = 246.8 Hz), 161.8 (d, $^1J_{C\text{-}F}$ = 245.5 Hz), 138.5 (d, $^4J_{C\text{-}F}$ = 3.2 Hz), 133.1, 132.6 (d, $^4J_{C\text{-}F}$ = 3.3 Hz), 132.4, 130.3 (d, $^3J_{C\text{-}F}$ = 8.0 Hz, 2C), 129.5 (d, $^3J_{C\text{-}F}$ = 7.9 Hz, 2C), 128.7, 117.4, 115.6 (d, $^2J_{C\text{-}F}$ = 21.3 Hz, 2C), 115.3 (d, $^2J_{C\text{-}F}$ = 21.4 Hz, 2C), 108.5, 106.6, 42.6. **^{19}F-NMR (282 MHz, CDCl$_3$)** δ -114.7, -115.9. **HRMS (EI$^+$)** calculated for $C_{19}H_{15}NF_2$ [M]$^+$: 295.1167, found: 295.1175.

General procedure for the preparation of *E*-allylic compounds (5)

A vial equipped with a magnetic stir bar and fitted with a teflon screw cap septum was charged with the corresponding allylic compound **1** (0.1 mmol), the corresponding heterocycle (0.2 mmol), 3-(4-methoxyphenyl)-10-phenyl-10*H*-phenoxazine (1.7 mg, 5

mol%), DIPA (70 μL, 0.5 mmol) and acetonitrile (1 mL). The reaction was degassed with three freeze-pump-thaw cycles. The vial was then backfilled with N_2 and stirred under 420 nm LEDs irradiation (18.3396 W/m^2 intensity; approximate distance was 2 cm from the vial) at room temperature. After 3 h the vial was opened, the solvent evaporated and the crude purified by column chromatography.

(*E*)-2-(1,3-diphenylallyl)-1*H*-pyrrole (5a)

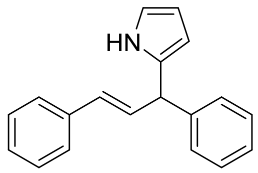

5a

From (*E*)-1,3-diphenylallyl acetate (25.2 mg, 0.1 mmol) and pyrrole (13.9 μL, 0.2 mmol), following the general procedure, compound **5a** (18.4 mg, 0.071 mmol) was obtained in 71% yield (*Z*:*E* ratio = 5:95) as a brownish oil. The crude product was purified by column chromatography. Eluent: Hexane:DCM (1:1). Spectroscopic data are in agreement with the published data.[17] **^{1}H-NMR (300 MHz, CDCl$_3$)** δ 7.88 (br s, 1H), 7.39 – 7.20 (m, 10H), 6.76 – 6.69 (m, 1H), 6.60 (dd, *J* = 15.8, 7.6 Hz, 1H), 6.43 (d, *J* = 15.8 Hz, 1H), 6.19 – 6.16 (m, 1H), 5.99 – 5.95 (m, 1H), 4.87 (d, *J* = 7.6 Hz, 1H).

(*E*)-2-(1,3-diphenylallyl)-1-methyl-1*H*-pyrrole (5b)

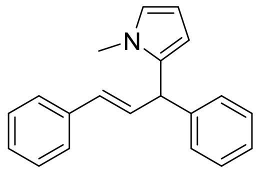

5b

From (*E*)-1,3-diphenylallyl acetate (25.2 mg, 0.1 mmol) and 1-methylpyrrole (16.2 μL, 0.2 mmol), following the general procedure, compound **5b** (10.8 mg, 0.040 mmol) was obtained in 40% yield (*Z*:*E* ratio = 10:90) as a yellow oil. The crude product was purified by column chromatography. Eluent: Hexane:DCM (1:3). **^{1}H-NMR (300 MHz, CDCl$_3$)** δ 7.40 – 7.15 (m, 10H), 6.63 – 6.56 (m, 2H), 6.25 (d, *J* = 15.7 Hz, 1H), 6.12 – 6.07 (m, 1H), 5.97 – 5.93 (m, 1H), 4.85 (d, *J* = 7.0 Hz, 1H), 3.40 (s, 3H). **^{13}C-NMR (75 MHz, CDCl$_3$)** δ 137.3, 133.7, 131.5, 130.9, 128.6, 128.54 (2C), 128.48 (2C), 128.4 (2C), 127.3, 126.6, 126.3 (2C), 122.2, 107.9, 106.5, 46.7, 34.0. **HRMS (EI$^+$)** calculated for $C_{20}H_{19}N$ [M]$^+$: 273.1512, found: 273.1516.

(*E*)-2-(1,3-diphenylallyl)-1-phenyl-1*H*-pyrrole (5c)

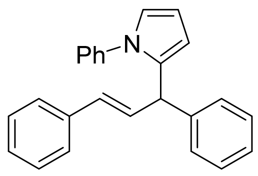

5c

From (*E*)-1,3-diphenylallyl acetate (25.2 mg, 0.1 mmol) and 1-phenylpyrrole (28.6 mg, 0.2 mmol), following the general procedure, compound **5c** (19.9 mg, 0.059 mmol) was obtained in 59% yield (*Z*:*E* ratio = 8:92) as an orange oil. The crude product was purified by column chromatography. Eluent: Hexane:DCM (4:1). **^{1}H-NMR (300 MHz, CDCl$_3$)** δ 7.35 – 7.04 (m, 15H), 6.79 – 6.76 (m, 1H), 6.52 (dd, *J* = 15.8, 7.0 Hz, 1H), 6.29 – 6.23 (m, 1H), 6.17 – 6.09 (m, 2H), 4.76 (d, *J* = 7.0 Hz, 1H). **^{13}C-NMR (75 MHz, CDCl$_3$)** δ 142.5, 140.1, 137.3, 134.6, 132.0, 130.7, 128.8 (2C), 128.41 (2C), 128.36 (2C), 128.3 (2C), 127.4, 127.2, 126.9 (2C), 126.33, 126.28 (2C),

122.4, 108.6, 108.0, 46.3. **HRMS (EI$^+$)** calculated for $C_{25}H_{21}N$ [M]$^+$: 335.1669, found: 335.1282.

(*E*)-3-(1,3-diphenylallyl)-1*H*-indole (5e)

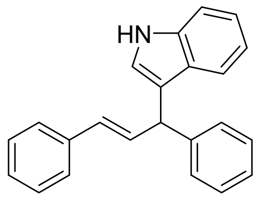

5e

From (*E*)-1,3-diphenylallyl acetate (25.2 mg, 0.1 mmol) and indole (23.4 mg, 0.2 mmol), following the general procedure, compound **5e** (23.7 mg, 0.078 mmol) was obtained in 78% yield (*Z*:*E* ratio = 4:96) as a brownish oil. The crude product was purified by column chromatography. Eluent: Hexane:DCM (6:1). Spectroscopic data are in agreement with the published data.[18] **^{1}H-NMR (300 MHz, CDCl$_3$)** δ 7.97 (br s, 1H), 7.42 (d, *J* = 8.0 Hz, 1H), 7.38 – 7.14 (m, 12H), 7.02 (t, *J* = 7.5 Hz, 1H), 6.92 – 6.87 (m, 1H), 6.73 (dd, *J* = 15.8, 7.3 Hz, 1H), 6.43 (d, *J* = 15.8 Hz, 1H), 5.12 (d, *J* = 7.4 Hz, 1H).

(*E*)-3-(1,3-diphenylallyl)-1-methyl-indole (5f)

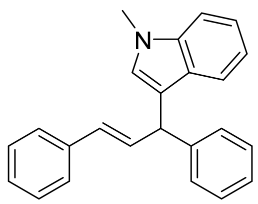

5f

From (*E*)-1,3-diphenylallyl acetate (25.2 mg, 0.1 mmol) and *N*-methylindole (26.2 μL, 0.2 mmol), following the general procedure, compound **5f** (30.4 mg, 0.094 mmol) was obtained in 94% yield (*Z*:*E* ratio = 7:93) as a yellow oil. The crude product was purified by column chromatography. Eluent: Hexane:DCM (6:1). Spectroscopic data are in agreement with the published data.[19] **^{1}H-NMR (300 MHz, CDCl$_3$)** δ 7.42 (d, *J* = 8.0 Hz, 1H), 7.35 – 7.18 (m, 12H), 7.01 (m, 1H), 6.76 – 6.66 (m, 2H), 6.43 (d, *J* = 15.8 Hz, 1H), 5.11 (d, *J* = 7.3 Hz, 1H), 3.72 (s, 3H).

(*E*)-3-(1,3-diphenylallyl)-5-methoxy-1*H*-indole (5g)

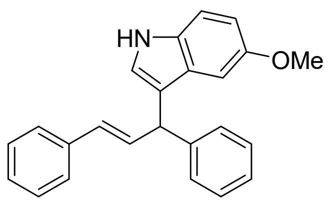

5g

From (*E*)-1,3-diphenylallyl acetate (25.2 mg, 0.1 mmol) and 5-methoxyindole (29.4 mg, 0.2 mmol), following the general procedure, compound **5g** (24.8 mg, 0.073 mmol) was obtained in 73% yield (*Z*:*E* ratio = 6:94) as a yellow oil. The crude product was purified by column chromatography. Eluent: Hexane:DCM (1:1). Spectroscopic data are in agreement with the published data.[17] **^{1}H-NMR (300 MHz, CDCl$_3$)** δ 7.90 (br s, 1H), 7.39 – 7.19 (m, 11H), 6.89 (dd, *J* = 2.5, 1.0 Hz, 1H), 6.85 – 6.81 (m, 2H), 6.71 (dd, *J* = 15.8, 7.3 Hz, 1H), 6.44 (d, *J* = 15.7 Hz, 1H), 5.07 (d, *J* = 7.3 Hz, 1H), 3.71 (s, 3H).

(*E*)-3-(1,3-diphenylallyl)-6-methoxy-1*H*-indole (5h)

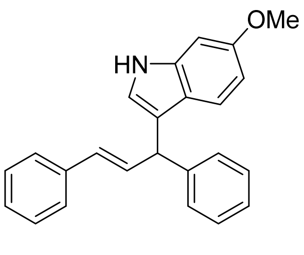

5h

From (*E*)-1,3-diphenylallyl acetate (25.2 mg, 0.1 mmol) and 6-methoxyindole (29.4 mg, 0.2 mmol), following the general procedure, compound **5h** (7.5 mg, 0.021 mmol) was obtained in 22% yield (*Z*:*E* ratio = 6:94) as a yellow oil. The crude product was purified by column chromatography. Eluent: Hexane:DCM (3:1). Spectroscopic data are in agreement with the published data.[20] **^{1}H-NMR (300 MHz, $CDCl_3$)** δ 7.88 (br s, 1H), 7.39 – 7.19 (m, 11H), 6.86 (d, *J* = 2.3 Hz, 1H), 6.81 (dd, *J* = 2.4, 1.1 Hz, 1H), 6.74 – 6.65 (m, 2H), 6.44 (d, *J* = 15.8 Hz, 1H), 5.07 (d, *J* = 7.5 Hz, 1H), 3.83 (s, 3H).

The C2-allylated compound was obtained as a side-product:

(*E*)-2-(1,3-diphenylallyl)-6-methoxy-1*H*-indole (5h')

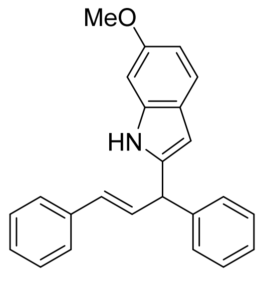

5h'

From (*E*)-1,3-diphenylallyl acetate (25.2 mg, 0.1 mmol) and 6-methoxyindole (29.4 mg, 0.2 mmol), following the general procedure, compound **5h'** (17.3 mg, 0.051 mmol) was obtained in 51% yield as a yellow oil. The crude product was purified by column chromatography. Eluent: Hexane:DCM (3:1). **^{1}H-NMR (300 MHz, $CDCl_3$)** δ 7.72 (br s, 1H), 7.43 – 7.20 (m, 11H), 6.79 – 6.77 (m, 1H), 6.75 (dd, *J* = 8.5, 2.3 Hz, 1H), 6.65 (dd, *J* = 15.8, 7.4 Hz, 1H), 6.48 (d, *J* = 15.9 Hz, 1H), 6.24 – 6.21 (m, 1H), 4.99 (d, *J* = 7.4 Hz, 1H), 3.81 (s, 3H). **^{13}C-NMR (75 MHz, $CDCl_3$)** δ 156.1, 141.5, 138.9, 137.0, 136.9, 131.8, 130.4, 128.8 (2C), 128.6 (2C), 128.5 (2C), 127.6, 127.1, 126.4 (2C), 122.7, 120.8, 109.5, 101.2, 94.5, 55.7, 48.5. **HRMS (EI$^+$)** calculated for $C_{24}H_{21}NO_3$ $[M]^+$: 339.1618, found: 339.1627.

(*E*)-3-(1,3-diphenylallyl)-5,6-dimethoxy-1*H*-indole (5i)

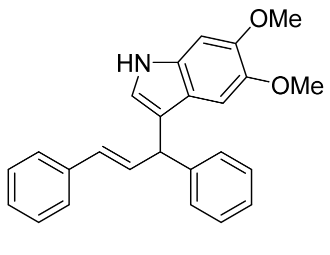

5i

From (*E*)-1,3-diphenylallyl acetate (25.2 mg, 0.1 mmol) and 5,6-dimethoxyindole (35.4 mg, 0.2 mmol), following the general procedure, compound **5i** (12.2 mg, 0.033 mmol) was obtained in 33% yield (*Z*:*E* ratio = 6:94) as a yellow oil. The crude product was purified by column chromatography. Eluent: Hexane:DCM (2:1). Spectroscopic data are in agreement with the published data.[21] **^{1}H-NMR (300 MHz, $CDCl_3$)** δ 7.83 (s, 1H), 7.39 – 7.27 (m, 8H), 7.23 – 7.18 (m, 2H), 6.88 – 6.86 (m, 1H), 6.79 – 6.76 (m, 2H), 6.70 (dd, *J* = 15.8, 7.3 Hz, 1H), 6.45 (d, *J* = 15.9 Hz, 1H), 5.05 (d, *J* = 7.3 Hz, 1H), 3.90 (s, 3H), 3.74 (s, 3H).

The C2-allylated compound was obtained as a side-product:

(*E*)-2-(1,3-diphenylallyl)-5,6-dimethoxy-1*H*-indole (5i')

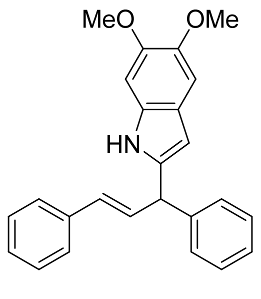

5i'

Compound **5i'** (18.5 mg, 0.050 mmol) was obtained in 50% yield as a yellow oil. The crude product was purified by column chromatography. Eluent: Hexane:DCM (2:1). **^{1}H-NMR (300 MHz, $CDCl_3$)** δ 7.68 (br s, 1H), 7.41 – 7.29 (m, 10H), 7.01 (s, 1H), 6.81 (s, 1H), 6.65 (dd, *J* = 15.8, 7.5 Hz, 1H), 6.48 (d, *J* = 15.8 Hz, 1H), 6.20 – 6.18 (m, 1H), 4.99 (d, *J* = 7.4 Hz, 1H), 3.90 (s, 3H), 3.87 (s, 3H). **^{13}C-NMR (75 MHz, $CDCl_3$)** δ 146.6, 145.1, 141.6, 138.7, 136.9, 131.7, 130.5, 128.8 (2C), 128.6 (2C), 128.5 (2C), 128.2, 127.6, 127.1, 126.4 (2C), 121.2, 102.3, 101.1, 94.5, 56.4, 56.2, 48.5. **HRMS (EI$^+$)** calculated for $C_{10}H_{10}NO_2$ [M – $C_{15}H_{13}$]$^+$: 176.0706, found: 176.0702.

(*E*)-3-(1,3-diphenylallyl)-5-(trifluoromethyl)-1*H*-indole (5j)

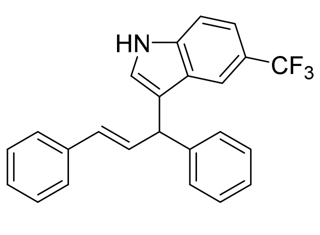

5j

From (*E*)-1,3-diphenylallyl acetate (25.2 mg, 0.1 mmol) and 5-(trifluoromethyl)indole (37.0 mg, 0.2 mmol), following the general procedure, compound **5j** (25.9 mg, 0.067 mmol) was obtained in 67% yield (*Z*:*E* ratio = 13:87) as an brownish oil. The crude product was purified by column chromatography. Eluent: Hexane:DCM (1:1). **^{1}H-NMR (300 MHz, $CDCl_3$)** δ 8.15 (br s, 1H), 7.70 (s, 1H), 7.41 – 7.39 (m, 2H), 7.38 – 7.19 (m, 10H), 7.00 (dd, *J* = 2.3, 0.7 Hz, 1H), 6.69 (dd, *J* = 15.8, 7.3 Hz, 1H), 6.42 (d, *J* = 15.9 Hz, 1H), 5.13 (d, *J* = 7.2 Hz, 1H). **^{13}C-NMR (75 MHz, $CDCl_3$)** 142.8, 137.9, 137.3, 132.0, 131.0, 128.6 (2C), 128.5 (2C), 128.4 (2C), 127.4 (q, $^1J_{C\text{-}F}$ = 244.5 Hz), 127.3, 126.7, 126.3 (2C), 126.2, 124.2, 122.0 (q, $^2J_{C\text{-}F}$ = 31.8 Hz), 119.8, 119.0 (q, $^3J_{C\text{-}F}$ = 3.5 Hz), 117.5 (q, $^3J_{C\text{-}F}$ = 4.4 Hz), 111.4, 45.9. **^{19}F-NMR (282 MHz, $CDCl_3$)** δ -60.3. **HRMS (ESI$^+$)** calculated for $C_{24}H_{18}NF_3$ [M]$^+$: 377.1386, found: 377.1387.

(*E*)-3-(1,3-diphenylallyl)-7-methyl-1*H*-indole (5k)

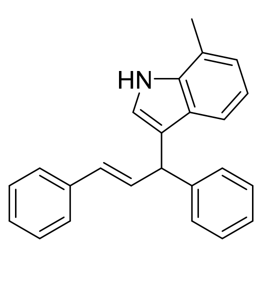

5k

From (*E*)-1,3-diphenylallyl acetate (25.2 mg, 0.1 mmol) and 7-methylindole (26.2 mg, 0.2 mmol), following the general procedure, compound **5k** (29.8 mg, 0.092 mmol) was obtained in 92% yield (*Z*:*E* ratio = 4:96) as an orange oil. The crude product was purified by column chromatography. Eluent: Hexane:DCM (2:1). Spectroscopic data are in agreement with the published data.[22] **^{1}H-NMR (300 MHz, $CDCl_3$)** δ 7.89 (br s, 1H), 7.38 – 7.18 (m, 11H),

7.00 – 6.94 (m, 2H), 6.92 – 6.88 (m, 1H), 6.72 (dd, *J* = 15.8, 7.4 Hz, 1H), 6.43 (d, *J* = 15.8 Hz, 1H), 5.11 (d, *J* = 7.3 Hz, 1H), 2.47 (s, 3H).

(*E*)-3-(1,3-diphenylallyl)-4-methyl-1*H*-indole (5l)

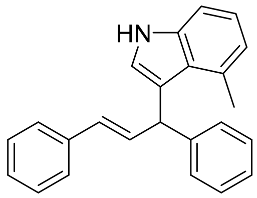

5l

From (*E*)-1,3-diphenylallyl acetate (25.2 mg, 0.1 mmol) and 4-methylindole (26.2 mg, 0.2 mmol), following the general procedure, compound **5l** (22.3 mg, 0.069 mmol) was obtained in 69% yield (*Z*:*E* ratio = 0:100) as a brownish oil. The crude product was purified by column chromatography. Eluent: *c*-Hexane:DCM (9:1). Spectroscopic data are in agreement with the published data.[23] **^{1}H-NMR (300 MHz, $CDCl_3$)** δ 8.04 (br s, 1H), 7.38 – 7.27 (m, 8H), 7.25 – 7.18 (m, 3H), 7.07 (t, *J* = 7.7 Hz, 1H), 6.87 (d, *J* = 2.6 Hz, 1H), 6.80 (d, *J* = 7.2 Hz, 1H), 6.75 (dd, *J* = 15.9, 6.5 Hz, 1H), 6.24 (d, *J* = 15.9 Hz, 1H), 5.46 (d, *J* = 6.5 Hz, 1H), 2.53 (s, 3H).

(*E*)-5-bromo-3-(1,3-diphenylallyl)-1*H*-indole (5m)

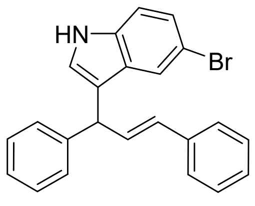

5m

From (*E*)-1,3-diphenylallyl acetate (25.2 mg, 0.1 mmol) and 5-bromoindole (39.2 mg, 0.2 mmol), following the general procedure, compound **5m** (29.9 mg, 0.076 mmol) was obtained in 76% yield (*Z*:*E* ratio = 17:83) as a brownish oil. The crude product was purified by column chromatography. Eluent: Hexane:DCM (2:1). Spectroscopic data are in agreement with the published data.[22] **^{1}H-NMR (300 MHz, $CDCl_3$)** δ 8.05 (br s, 1H), 7.55 (s, 1H), 7.40 – 7.20 (m, 12H), 6.93 (dd, *J* = 2.4, 0.9 Hz, 1H), 6.69 (dd, *J* = 15.8, 7.2 Hz, 1H), 6.41 (d, *J* = 15.9 Hz, 1H), 5.07 (d, *J* = 7.1 Hz, 1H).

General procedure for the preparation of *Z*-allylic compounds (9)

A vial equipped with a magnetic stir bar and fitted with a teflon screw cap septum was charged with the corresponding allylic compound **1** (0.1 mmol), the corresponding amine or alcohol **8** (0.2 mmol), *N*-phenyl phenothiazine (1.4 mg, 5 mol%), DIPEA (86 μL, 0.5 mmol) and acetonitrile (1 mL). The reaction was degassed with three freeze-pump-thaw cycles. The vial was then backfilled with N_2 and stirred under 365 nm LEDs irradiation (8.2460 W/m^2 intensity; approximate distance was 2 cm from the vial) at 20 ºC. After 3 h the vial was opened, the solvent evaporated and the crude purified by column chromatography.

(*Z*)-*N*-(1,3-diphenylallyl)aniline (9a)

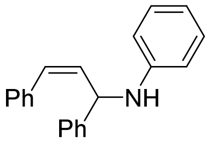

9a

From (*E*)-1,3-diphenylallyl acetate (25.2 mg, 0.1 mmol) and aniline (19 μL, 0.2 mmol), following the general procedure, compound **9a** (17.4 mg, 0.061 mmol) was obtained in 61% yield (*Z*:*E* ratio = 87:13) as a colorless oil. The crude product was purified by column chromatography. Eluent: Hexane:DCM (2:1). **^{1}H-NMR (300 MHz, $CDCl_3$)** δ 7.47 – 7.42 (m, 2H), 7.39 – 7.26 (m, 8H), 7.11 – 7.04 (m, 2H), 6.72 – 6.64 (m, 2H), 6.49 – 6.42 (m, 2H), 5.82 (dd, *J* = 11.4, 9.4 Hz, 1H), 5.40 (d, *J* = 9.3 Hz, 1H), 4.13 (br s, 1H). **^{13}C-NMR (75 MHz, $CDCl_3$)** δ 146.8, 142.7, 136.5, 133.2, 131.2, 129.1 (2C), 128.9 (2C), 128.8 (2C), 128.5 (2C), 127.5 (2C), 127.0 (2C), 117.6, 113.5 (2C), 55.5. **HRMS (EI$^+$)** calculated for $C_{21}H_{19}N$ [M]$^+$: 285.1517, found: 285.1520.

(*Z*)-*N*-(1,3-diphenylallyl)-4-methylaniline (9b)

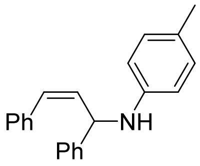

9b

From (*E*)-1,3-diphenylallyl acetate (25.2 mg, 0.1 mmol) and *p*-toluidine (21.4 mg, 0.2 mmol), following the general procedure, compound **9b** (14.3 mg, 0.048 mmol) was obtained in 48% yield (*Z*:*E* ratio = 76:24) as a light yellow oil. The crude product was purified by column chromatography. Eluent: DCM. **^{1}H-NMR (300 MHz, $CDCl_3$)** δ 7.46 – 7.41 (m, 2H), 7.39 – 7.26 (m, 8H), 6.89 (d, *J* = 8.0 Hz, 2H), 6.68 (d, *J* = 11.4 Hz, 1H), 6.38 (d, *J* = 8.4 Hz, 2H), 5.81 (dd, *J* = 11.4, 9.4 Hz, 1H), 5.37 (d, *J* = 9.4 Hz, 1H), 4.08 (br s, 1H), 2.20 (s, 3H). **^{13}C-NMR (75 MHz, $CDCl_3$)** δ 144.6, 142.9, 136.6, 133.4, 131.0, 129.6 (2C), 128.81 (2C), 128.75 (2C), 128.4 (2C), 127.4 (2C), 127.0 (2C), 126.8, 113.7 (2C), 55.7, 20.4. **HRMS (EI$^+$)** calculated for $C_{22}H_{21}N$ [M]$^+$: 299.1669, found: 299.1674.

(*Z*)-*N*-(1,3-diphenylallyl)-4-methoxyaniline (9c)

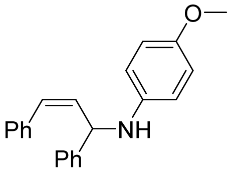

9c

From (*E*)-1,3-diphenylallyl acetate (25.2 mg, 0.1 mmol) and 4-methoxyaniline (24.6 mg, 0.2 mmol), following the general procedure, compound **9c** (24.2 mg, 0.077 mmol) was obtained in 77% yield (*Z*:*E* ratio = 36:64) as a yellow oil. The crude product was purified by column chromatography. Eluent: DCM. **^{1}H-NMR (300 MHz, $CDCl_3$)** δ 7.43 – 7.40 (m, 2H), 7.37 – 7.20 (m, 8H), 6.75 – 6.70 (m, 2H), 6.69 – 6.56 (m, 3H), 5.80 (dd, *J* = 11.3, 9.6 Hz, 1H), 5.31 (d, *J* = 9.4 Hz, 1H), 3.88 (br s, 1H), 3.69 (s, 3H). **^{13}C-NMR (75 MHz, $CDCl_3$)** δ 152.2, 142.9, 141.1, 136.6, 133.6, 131.0, 128.81, 128.76 (2C), 128.65, 128.4 (2C), 127.4 (2C), 127.0 (2C), 114.9 (2C), 114.8 (2C), 56.3, 55.7. **HRMS (EI$^+$)** calculated for $C_{22}H_{21}NO$ [M]$^+$: 315.1623, found: 315.1623.

(*Z*)-4-(*tert*-butoxy)-*N*-(1,3-diphenylallyl)aniline (9d)

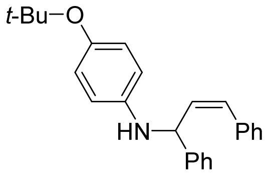

9d

From (*E*)-1,3-diphenylallyl acetate (25.2 mg, 0.1 mmol) and 4-*tert*-butoxyaniline (33 mg, 0.2 mmol), following the general procedure, compound **9d** (18.8 mg, 0.053 mmol) was obtained in 53% yield (*Z*:*E* ratio = 65:35) as a yellow oil. The crude product was purified by column chromatography. Eluent: DCM. **^{1}H-NMR (300 MHz, CDCl$_3$)** δ 7.50 – 7.46 (m, 2H), 7.42 – 7.23 (m, 8H), 6.73 and 6.35 (AA'BB' system, 4H), 6.70 (d, *J* = 11.4 Hz, 1H), 5.83 (dd, *J* = 11.5, 9.4 Hz, 1H), 5.33 (d, *J* = 9.3 Hz, 1H), 3.98 (br s, 1H), 1.28 (s, 9H). **^{13}C-NMR (75 MHz, CDCl$_3$)** δ 146.6, 143.2, 142.9, 136.5, 133.6, 131.1, 128.8 (2C), 128.7 (2C), 128.4 (2C), 127.1 (2C), 126.6, 126.4, 125.2 (2C), 113.8 (2C), 77.7, 56.2, 28.7 (3C). **HRMS (EI$^+$)** calculated for $C_{25}H_{27}NO$ [M]$^+$: 357.2093, found: 357.2034.

(*Z*)-*N*-(1,3-diphenylallyl)-4-(trifluoromethyl)aniline (9e)

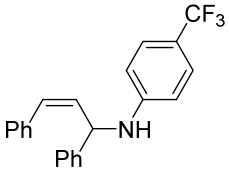

9e

From (*E*)-1,3-diphenylallyl acetate (25.2 mg, 0.1 mmol) and 4-(trifluoromethyl)aniline (25.1 μL, 0.2 mmol), following the general procedure, compound **9e** (27.2 mg, 0.076 mmol) was obtained in 76% yield (*Z*:*E* ratio = 88:12) as a colorless oil. The crude product was purified by column chromatography. Eluent: Hexane:DCM (3:1). **^{1}H-NMR (300 MHz, CDCl$_3$)** δ 7.49 – 7.28 (m, 12H), 6.75 (d, *J* = 11.4 Hz, 1H), 6.44 (d, *J* = 8.7 Hz, 2H), 5.81 (dd, *J* = 11.4, 9.3 Hz, 1H), 5.42 (d, *J* = 9.3 Hz, 1H), 4.46 (br s, 1H). **^{13}C-NMR (75 MHz, CDCl$_3$)** δ 149.2, 141.8, 136.2, 132.1, 131.9, 129.0 (2C), 128.7 (2C), 128.5 (2C), 127.8, 127.7, 126.89 (q, $^2J_{C\text{-}F}$ = 35.1 Hz), 126.89 (2C), 126.5 (q, $^3J_{C\text{-}F}$ = 4.3, 3.8 Hz, 2C), 121.3 (q, $^1J_{C\text{-}F}$ = 282.1 Hz), 112.6 (2C), 55.2. **^{19}F-NMR (282 MHz, CDCl$_3$)** δ -61.1. . **HRMS (EI$^+$)** calculated for $C_{22}H_{18}F_3N$ [M]$^+$: 353.1391, found: 353.1373.

(*Z*)-2-bromo-*N*-(1,3-diphenylallyl)aniline (9f)

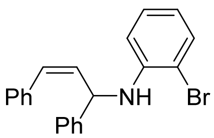

9f

From (*E*)-1,3-diphenylallyl acetate (25.2 mg, 0.1 mmol) and 2-bromoaniline (21.8 μL, 0.2 mmol), following the general procedure, compound **9f** (24.4 mg, 0.067 mmol) was obtained in 67% yield (*Z*:*E* ratio = 84:16) as a colorless oil. The crude product was purified by column chromatography. Eluent: Hexane:DCM (3:1). **^{1}H-NMR (300 MHz, CDCl$_3$)** δ 7.45 – 7.26 (m, 11H), 6.95 (ddd, *J* = 8.5, 7.4, 1.5 Hz, 1H), 6.72 (d, *J* = 11.4 Hz, 1H), 6.53 (td, *J* = 7.6, 1.5 Hz, 1H), 6.27 (dd, *J* = 8.2, 1.5 Hz, 1H), 5.82 (dd, *J* = 11.4, 9.4 Hz, 1H), 5.42 (dd, *J* = 9.4, 5.7 Hz, 1H), 4.82 (d, *J* = 5.9 Hz, 1H). **^{13}C-NMR (75 MHz, CDCl$_3$)** δ 143.6, 142.0, 136.4, 132.5, 132.3, 131.6, 129.0 (2C), 128.7 (2C), 128.5 (2C), 128.3, 127.62,

127.56, 126.9 (2C), 118.1, 112.8, 109.9, 55.5. **HRMS (EI+)** calculated for $C_{21}H_{18}BrN$ $[M]^+$: 363.0623, found: 363.0623.

(*Z*)-*N*-(1,3-diphenylallyl)butan-2-amine (9g)

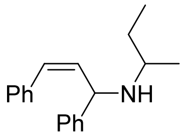

9g

From (*E*)-1,3-diphenylallyl acetate (25.2 mg, 0.1 mmol) and *sec*-butylamine (20.2 μL, 0.2 mmol), following the general procedure, compound **9g** (16 mg, 0.061 mmol) was obtained in 61% yield (*Z*:*E* ratio = 93:7) as an orange oil. The crude product was purified by column chromatography. Eluent: DCM: MeOH (4:1). **^{1}H-NMR (300 MHz, CDCl$_3$)** δ 7.46 – 7.26 (m, 10H), 6.63, 6.57 (2 x d, *J* = 11.7 Hz, 1H), 5.85, 5.80 (2 x dd, *J* = 11.6, 9.8 Hz, 1H), 4.82 (d, *J* = 9.8 Hz, 1H), 2.58, 2.50 (2 x q, *J* = 6.2 Hz, 1H), 2.11 (br s, 1H), 1.37 – 1.14 (m, 2H), 1.01, 0.86 (2 x d, *J* = 6.3 Hz, 2H), 0.85, 0.76 (2 x t, *J* = 7.4 Hz, 3H). **^{13}C-NMR (75 MHz, CDCl$_3$)** δ 137.1, 130.1, 129.8, 128.7 (2C), 128.6 (2C), 128.2 (2C), 127.4, 127.3 (2C), 127.2, 127.02, 127.00, 56.9, 56.6, 51.6, 51.4, 29.6, 29.4, 19.9, 19.8, 10.3, 10.1. **HRMS (EI+)** calculated for $C_{19}H_{23}N$ $[M]^+$: 265.1830, found: 265.1830.

(*Z*)-*N*,*N*-dibenzyl-1,3-diphenylprop-2-en-1-amine (9h)

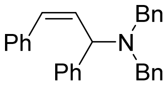

9h

From (*E*)-1,3-diphenylallyl acetate (25.2 mg, 0.1 mmol) and dibenzylamine (38.5 μL, 0.2 mmol), following the general procedure, compound **9h** (30.1 mg, 0.077 mmol) was obtained in 77% yield (*Z*:*E* ratio = 85:15) as a colorless oil. The crude product was purified by column chromatography. Eluent: Hexane:DCM (2:1). **^{1}H-NMR (300 MHz, CDCl$_3$)** δ 7.67 – 7.59 (m, 2H), 7.45 – 7.28 (m, 4H), 7.17 – 7.11 (m, 12H), 7.07 – 6.99 (m with d at 7.03, *J* = 11.8 Hz, 3H), 6.09 (dd, *J* = 11.8, 10.4 Hz, 1H), 4.85 (d, *J* = 10.5 Hz, 1H), 3.72 and 3.42 (AB System, *J* = 13.4 Hz, 4H). **^{13}C-NMR (75 MHz, CDCl$_3$)** δ 142.3, 139.8 (2C), 136.8, 133.9, 128.8 (4C), 128.5 (2C), 128.3 (2C), 128.23 (2C), 128.18 (2C), 127.9 (4C), 127.8, 127.03, 126.97, 126.6 (2C), 58.7, 53.6 (2C). **HRMS (EI+)** calculated for $C_{15}H_{13}$ $[M]^+$ $[M - C_{14}H_{14}N]^+$: 193.1012, found: 193.1019.

(*Z*)-1-(1,3-diphenylallyl)piperidine (9i)

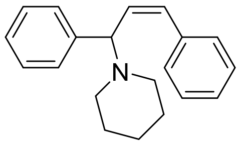

9i

From (*E*)-1,3-diphenylallyl acetate (25.2 mg, 0.1 mmol) and piperidine (20.0 μL, 0.2 mmol), following the general procedure, compound **9i** (18.7 mg, 0.068 mmol) was obtained in 68% yield (*Z*:*E* ratio = 91:9) as a pale yellow oil. The crude product was purified by column chromatography. Eluent: Pentane:AcOEt (98:2). Spectroscopic data are in agreement with the published data.[24] **^{1}H-NMR (300 MHz, CDCl$_3$)** δ 7.40 – 7.20 (m, 10H), 6.62 (d, *J* = 11.7 Hz, 1H), 5.95 (dd, *J* = 11.7, 10.1 Hz, 1H), 4.24 (d, *J* = 10.1 Hz, 1H),

2.54 – 2.40 (m, 2H), 2.25 (dt, *J* = 11.0, 5.3 Hz, 2H), 1.56 – 1.47 (m, 4H), 1.43 – 1.35 (m, 2H).

(*Z*)-4-(1,3-diphenylallyl)morpholine (9j)

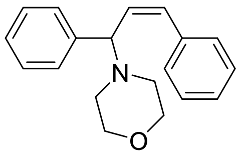

9j

From (*E*)-1,3-diphenylallyl acetate (25.2 mg, 0.1 mmol) and morpholine (17.0 μL, 0.2 mmol), following the general procedure, compound **9j** (20.0 mg, 0.076 mmol) was obtained in 76% yield (*Z*:*E* ratio = 94:6) as a white solid. The crude product was purified by column chromatography. Eluent: Cyclohexane:AcOEt (95:5). Spectroscopic data are in agreement with the published data.[24] **^{1}H-NMR (300 MHz, $CDCl_3$)** δ 7.40 – 7.20 (m, 10H), 6.66 (d, *J* = 11.7 Hz, 1H), 5.91 (dd, *J* = 11.7, 10.1 Hz, 1H), 4.23 (d, *J* = 10.1 Hz, 1H), 3.65 (t, *J* = 4.7 Hz, 4H), 2.52 (dt, *J* = 11.5, 4.7 Hz, 2H), 2.28 (dt, *J* = 11.5, 4.7 Hz, 2H).

(*Z*)-(3-methoxyprop-1-ene-1,3-diyl)dibenzene (9k)

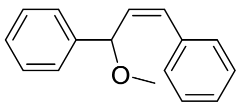

9k

From (*E*)-1,3-diphenylallyl acetate (25.2 mg, 0.1 mmol) and methanol (8.0 μL, 0.2 mmol), following the general procedure, compound **9k** (17.0 mg, 0.076 mmol) was obtained in 76% yield (*Z*:*E* ratio = 88:12) as a yellow oil. The crude product was purified by column chromatography. Eluent: Cyclohexane: DCM (95:5). **^{1}H-NMR (300 MHz, $CDCl_3$)** δ 7.44 – 7.23 (m, 10H), 6.75 (d, *J* = 11.6 Hz, 1H), 5.87 (dd, *J* = 11.6, 9.3 Hz, 1H), 5.07 (d, *J* = 9.3 Hz, 1H), 3.27 (s, 3H). **^{13}C-NMR (75 MHz, $CDCl_3$):** δ 141.2, 136.7, 132.3, 132.0, 128.8 (2C), 128.6 (2C), 128.3 (2C), 127.8, 127.3, 127.0 (2C), 78.9, 55.9. **HRMS (EI$^+$)** calculated for $C_{16}H_{16}O$ $[M]^+$: 224.1196, found: 224.1188.

(*Z*)-(3-ethoxyprop-1-ene-1,3-diyl)dibenzene (9l)

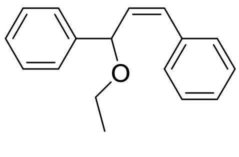

9l

From (*E*)-1,3-diphenylallyl acetate (25.2 mg, 0.1 mmol) and ethanol (12.0 μL, 0.2 mmol), following the general procedure, compound **9l** (16.4 mg, 0.069 mmol) was obtained in 69% yield (*Z*:*E* ratio = 86:14) as a yellow oil. The crude product was purified by column chromatography. Eluent: Cyclohexane:DCM (95:5). **^{1}H-NMR (300 MHz, $CDCl_3$)** δ 7.43 – 7.23 (m, 10H), 6.71 (d, *J* = 11.6 Hz, 1H), 5.88 (dd, *J* = 11.6, 9.4 Hz, 1H), 5.19 (d, *J* = 9.4 Hz, 1H), 3.50 (dq, *J* = 9.1, 7.0 Hz, 1H), 3.34 (dq, *J* = 9.1, 7.0 Hz, 1H), 1.19 (t, *J* = 7.0 Hz, 3H). **^{13}C-NMR (75 MHz, $CDCl_3$):** δ 141.7, 136.7, 132.7, 132.0, 128.8 (2C), 128.6 (2C), 128.2 (2C), 127.6, 127.2, 127.0 (2C), 77.2, 63.5, 15.4. **HRMS (EI$^+$)** calculated for $C_{17}H_{18}O$ $[M]^+$: 238.1352, found: 238.1336.

(*Z*)-(3-isobutoxyprop-1-ene-1,3-diyl)dibenzene (9m)

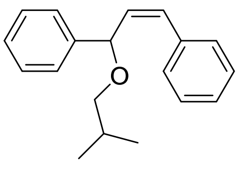

9m

From (*E*)-1,3-diphenylallyl acetate (25.2 mg, 0.1 mmol) and 2-methylpropan-1-ol (18.0 μL, 0.2 mmol), following the general procedure, compound **9m** (17.6 mg, 0.066 mmol) was obtained in 66% yield (*Z*:*E* ratio = 86:14) as a yellow oil. The crude product was purified by column chromatography. Eluent: Cyclohexane: DCM (95:5). **^{1}H-NMR (300 MHz, $CDCl_3$)** δ 7.44 – 7.24 (m, 10H), 6.72 (d, *J* = 11.6 Hz, 1H), 5.85 (dd, *J* = 11.6, 9.4 Hz, 1H), 5.16 (d, *J* = 9.4 Hz, 1H), 3.21 (dd, *J* = 9.0, 6.5 Hz, 1H), 3.05 (dd, *J* = 8.9, 6.7 Hz, 1H), 1.92 – 1.79 (m, 1H), 0.91 (d, *J* = 6.6 Hz, 3H), 0.89 (d, *J* = 6.6 Hz, 3H). **^{13}C-NMR (75 MHz, $CDCl_3$):** δ 141.8, 136.7, 132.7, 131.6, 128.8 (2C), 128.5 (2C), 128.2 (2C), 127.6, 127.2, 126.9 (2C), 77.3, 75.1, 28.6, 19.6 (2C). **HRMS (EI$^+$)** calculated for $C_{19}H_{22}O$ [M]$^+$: 266.1665, found: 266.1669.

General procedure for the preparation of *E*-allylic compounds (10)

A vial equipped with a magnetic stir bar and fitted with a teflon screw cap septum was charged with the corresponding allylic compound **1** (0.1 mmol), the corresponding amine or alcohol (0.2 mmol), 3-(4-methoxyphenyl)-10-phenyl-10*H*-phenoxazine (1.7 mg, 5 mol%) and acetonitrile (1 mL). The reaction was degassed with three freeze-pump-thaw cycles. The vial was then backfilled with N_2 and stirred under 420 nm LEDs irradiation (18.3396 W/m^2 intensity; approximate distance was 2 cm from the vial) at room temperature. After 3 h the vial was opened, the solvent evaporated and the crude purified by column chromatography.

(*E*)-*N*-(1,3-diphenylallyl)aniline (10a)

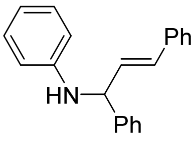

10a

From (*E*)-1,3-diphenylallyl acetate (25.2 mg, 0.1 mmol) and aniline (19 μL, 0.2 mmol), following the general procedure, compound **10a** (26.4 mg, 0.092 mmol) was obtained in 92% yield (*Z*:*E* ratio = 9:91) as a colorless oil. The crude product was purified by column chromatography. Eluent: Hexane:DCM (2:1). Spectroscopic data are in agreement with the published data.[25] **^{1}H-NMR (300 MHz, $CDCl_3$)** δ 7.48 – 7.25 (m, 10H), 7.20 – 7.12 (m, 2H), 6.75 – 6.61 (m, 4H), 6.41 (dd, *J* = 15.8, 6.1 Hz, 1H), 5.10 (d, *J* = 5.7 Hz, 1H), 4.16 (br s, 1H). **HRMS (EI$^+$)** calculated for $C_{21}H_{19}N$ [M]$^+$: 285.1517, found: 285.1520.

(*E*)-*N*-(1,3-diphenylallyl)-4-methylaniline (10b)

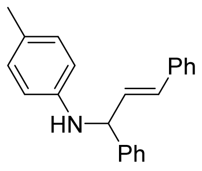

10b

From (*E*)-1,3-diphenylallyl acetate (25.2 mg, 0.1 mmol) and *p*-toluidine (21.4 mg, 0.2 mmol), following the general procedure, compound **10b** (25.5 mg, 0.084 mmol) was obtained in 84% yield (*Z*:*E* ratio = 8:92) as a colorless oil. The crude product was purified by column chromatography. Eluent: Hexane:DCM (3:1). Spectroscopic data are in agreement with the published data.[26] **^{1}H-NMR (300 MHz, $CDCl_3$)** δ 7.47 – 7.41 (m, 2H), 7.39 – 7.25 (m, 8H), 6.96 and 6.56 (AA'BB' system, 4H), 6.63 (d, *J* = 15.7 Hz, 1H), 6.39 (dd, *J* = 15.8, 6.1 Hz, 1H), 5.06 (d, *J* = 6.2 Hz, 1H), 4.18 (br s, 1H), 2.22 (s, 3H).

(*E*)-*N*-(1,3-diphenylallyl)-4-methoxyaniline (10c)

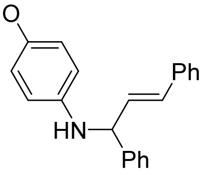

10c

From (*E*)-1,3-diphenylallyl acetate (25.2 mg, 0.1 mmol) and 4-methoxyaniline (24.6 mg, 0.2 mmol), following the general procedure and adding DIPEA (86 μL, 0.5 mmol), compound **10c** (27.8 mg, 0.087 mmol) was obtained in 87% yield (*Z*:*E* ratio = 4:96) as a yellow oil. The crude product was purified by column chromatography. Eluent: DCM. Spectroscopic data are in agreement with the published data.[26] **^{1}H-NMR (300 MHz, $CDCl_3$)** δ 7.45 – 7.39 (m, 2H), 7.37 – 7.19 (m, 8H), 6.73 and 6.59 (AA'BB' system, 4H), 6.61 (d, *J* = 16.1 Hz, 1H), 6.38 (dd, *J* = 15.8, 6.2 Hz, 1H), 5.00 (d, *J* = 6.2 Hz, 1H), 3.86 (br s, 1H), 3.71 (s, 3H).

(*E*)-4-(*tert*-butoxy)-*N*-(1,3-diphenylallyl)aniline (10d)

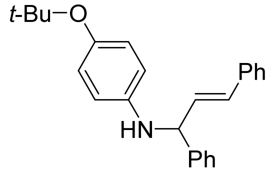

10d

From (*E*)-1,3-diphenylallyl acetate (25.2 mg, 0.1 mmol) and 4-*tert*-butoxyaniline (33 mg, 0.2 mmol), following the general procedure and adding DIPEA (86 μL, 0.5 mmol), compound **10d** (28.8 mg, 0.079 mmol) was obtained in 79% yield (*Z*:*E* ratio = 3:97) as a yellow oil. The crude product was purified by column chromatography. Eluent: DCM. **^{1}H-NMR (300 MHz, $CDCl_3$)** δ 7.45 – 7.40 (m, 2H), 7.38 – 7.17 (m, 8H), 6.78 and 6.53 (AA'BB' system, 4H), 6.61 (d, *J* = 15.9 Hz, 1H), 6.38 (dd, *J* = 15.8, 6.3 Hz, 1H), 5.01 (d, *J* = 6.2 Hz, 1H), 3.95 (br s, 1H), 1.26 (s, 9H). **^{13}C-NMR (75 MHz, $CDCl_3$)** δ 146.7, 143.6, 142.4, 136.8, 131.2, 131.0, 128.8 (2C), 128.6 (2C), 127.7, 127.5, 127.2 (2C), 126.5 (2C), 125.3 (2C), 113.9 (2C), 77.7, 61.4, 28.7 (3C). **HRMS (EI$^+$)** calculated for $C_{25}H_{27}NO$ [M]$^+$: 357.2093, found: 357.2087.

(*E*)-*N*-(1,3-diphenylallyl)-4-(trifluoromethyl)aniline (10e)

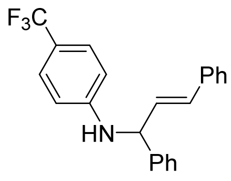

10e

From (*E*)-1,3-diphenylallyl acetate (25.2 mg, 0.1 mmol) and 4-(trifluoromethyl)aniline (25.1 μL, 0.2 mmol), following the general procedure, compound **10e** (30.1 mg, 0.084 mmol) was obtained in 84% yield (*Z*:*E* ratio = 7:93) as a yellow solid. The crude product was purified by column chromatography. Eluent: Hexane:DCM (3:1). Spectroscopic data are in agreement with the published data.[27] **^{1}H-NMR (300 MHz, $CDCl_3$)** δ 7.44 – 7.21 (m, 12H), 6.67 – 6.55 (m, 3H), 6.37 (dd, *J* = 15.8, 6.1 Hz, 1H), 5.12 (m, 1H), 4.42 (d, *J* = 5.1 Hz, 1H).

(*E*)-2-bromo-*N*-(1,3-diphenylallyl)aniline (10f)

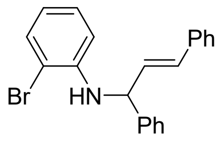

10f

From (*E*)-1,3-diphenylallyl acetate (25.2 mg, 0.1 mmol) and 2-bromoaniline (21.8 μL, 0.2 mmol), following the general procedure, compound **10f** (30.2 mg, 0.082 mmol) was obtained in 82% yield (*Z*:*E* ratio = 7:93) as a light yellow oil. The crude product was purified by column chromatography. Eluent: Hexane:DCM (3:1). **^{1}H-NMR (300 MHz, $CDCl_3$)** δ 7.45 – 7.40 (m, 3H), 7.39 – 7.33 (m, 4H), 7.32 – 7.26 (m, 3H), 7.25 – 7.21 (m, 1H), 7.06 (ddd, *J* = 8.5, 7.3, 1.5 Hz, 1H), 6.63 – 6.52 (m, 3H), 6.40 (dd, *J* = 15.9, 6.1 Hz, 1H), 5.13 (t, *J* = 5.5 Hz, 1H), 4.81 (d, *J* = 5.0 Hz, 1H). **^{13}C-NMR (75 MHz, $CDCl_3$)** δ 144.0, 141.5, 136.5, 132.4, 131.5, 130.2, 129.0 (2C), 128.6 (2C), 128.4, 127.8, 127.7, 127.1 (2C), 126.6 (2C), 118.2, 112.9, 110.0, 60.5. **HRMS (EI^+)** calculated for $C_{21}H_{18}BrN$ $[M]^+$: 363.0623, found: 363.0623.

(*E*)-*N*-(1,3-diphenylallyl)butan-2-amine (10g)

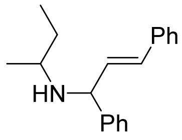

10g

From (*E*)-1,3-diphenylallyl acetate (25.2 mg, 0.1 mmol) and *sec*-butylamine (20.2 μL, 0.2 mmol), following the general procedure, compound **10g** (10.3 mg, 0.039 mmol) was obtained in 39% yield (*Z*:*E* ratio = 7:93) as an orange oil. The crude product was purified by column chromatography. Eluent: DCM: MeOH (6:1). **^{1}H-NMR (300 MHz, $CDCl_3$)** δ 7.40 – 7.24 (m, 10H), 6.55, 6.50 (2 x d, *J* = 15.7 Hz, 1H), 6.32, 6.27 (2 x dd, *J* = 15.7, 6.7 Hz, 1H), 4.51, 4.48 (2 x d, *J* = 6.6 Hz, 1H), 2.64, 2.56 (2 x q, *J* = 6.3 Hz, 1H), 1.85 (br s, 1H), 1.54 – 1.32 (m, 2H), 1.07, 1.04 (2 x d, *J* = 6.3 Hz, 3H), 0.89, 0.86 (2 x t, *J* = 7.4 Hz, 3H). **^{13}C-NMR (75 MHz, $CDCl_3$)** δ 137.1, 130.0, 129.9, 128.63 (2C), 128.55 (2C), 128.48, 128.47, 127.39, 127.35, 127.29 (2C), 127.13, 127.12, 126.4 (2C), 62.43, 62.43, 51.4, 51.2, 29.8, 29.5, 20.1, 19.9, 10.17, 10.15. **HRMS (EI^+)** calculated for $C_{19}H_{23}N$ $[M]^+$: 265.1830, found: 265.1830.

(*E*)-*N,N*-dibenzyl-1,3-diphenylprop-2-en-1-amine (10h)

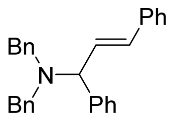

10h

From (*E*)-1,3-diphenylallyl acetate (25.2 mg, 0.1 mmol) and dibenzylamine (38.5 μL, 0.2 mmol), following the general procedure, compound **10h** (23.4 mg, 0.060 mmol) was obtained in 60% yield (*Z*:*E* ratio = 5:95) as a colorless oil. The crude product was purified by column chromatography. Spectroscopic data are in agreement with the published data.[28] Eluent: Hexane:DCM (3:1). **^{1}H-NMR (300 MHz, $CDCl_3$)** δ 7.57 – 7.52 (m, 2H), 7.45 – 7.40 (m, 6H), 7.38 – 7.30 (m, 7H), 7.29 – 7.21 (m, 5H), 6.57 – 6.41 (m, 2H), 4.44 (d, *J* = 6.7 Hz, 1H), 3.73 and 3.61 (AB system, *J* = 13.8 Hz, 4H).

(*E*)-1-(1,3-diphenylallyl)piperidine (10i)

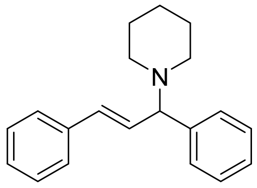

10i

From (*E*)-1,3-diphenylallyl acetate (25.2 mg, 0.1 mmol) and piperidine (20.0 μL, 0.2 mmol), following the general procedure, compound **10i** (18.8 mg, 0.068 mmol) was obtained in 68% yield (*Z*:*E* ratio = 12:88) as a pale yellow solid. The crude product was purified by column chromatography. Eluent: Pentane:AcOEt (98:2). Spectroscopic data are in agreement with the published data.[29] **^{1}H-NMR (300 MHz, $CDCl_3$)** δ 7.42 – 7.15 (m, 10H), 6.52 (d, *J* = 15.8 Hz, 1H), 6.34 (dd, *J* = 15.8, 8.6 Hz, 1H), 3.81 (d, *J* = 8.6 Hz, 1H), 2.55 – 2.45 (m, 2H), 2.40 – 2.28 (m, 2H), 1.62 – 1.51 (m, 4H), 1.47 – 1.39 (m, 2H).

(*E*)-4-(1,3-diphenylallyl)morpholine (10j)

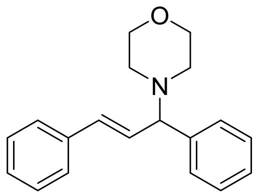

10j

From (*E*)-1,3-diphenylallyl acetate (25.2 mg, 0.1 mmol) and morpholine (17.0 μL, 0.2 mmol), following the general procedure, compound **10j** (18.4 mg, 0.066 mmol) was obtained in 66% yield (*Z*:*E* ratio = 5:95) as a yellow oil. The crude product was purified by column chromatography. Eluent: Cyclohexane:AcOEt (98:2). Spectroscopic data are in agreement with the published data.[29] **^{1}H-NMR (300 MHz, $CDCl_3$)** δ 7.44 – 7.16 (m, 10H), 6.56 (d, *J* = 15.8 Hz, 1H), 6.28 (dd, *J* = 15.8, 8.9 Hz, 1H), 3.78 (d, *J* = 8.9 Hz, 1H), 3.70 (t, *J* = 4.6 Hz, 4H), 2.62 – 2.50 (m, 2H), 2.38 (dt, *J* = 11.6, 4.6 Hz, 2H).

(*E*)-(3-methoxyprop-1-ene-1,3-diyl)dibenzene (10k)

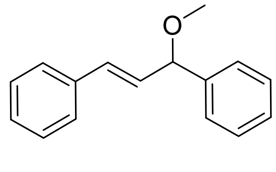

10k

From (*E*)-1,3-diphenylallyl acetate (25.2 mg, 0.1 mmol) and methanol (8.0 μL, 0.2 mmol), following the general procedure, compound **10k** (10.8 mg, 0.048 mmol) was obtained in 48% yield (*Z*:*E* ratio = 5:95) as a yellow oil. The crude product was purified

by column chromatography. Eluent: Cyclohexane:DCM (95:5). Spectroscopic data are in agreement with the published data.[30] **^{1}H-NMR (300 MHz, $CDCl_3$)** δ 7.42 – 7.35 (m, 6H), 7.35 – 7.19 (m, 4H), 6.63 (d, *J* = 15.9 Hz, 1H), 6.28 (dd, *J* = 15.9, 7.0 Hz, 1H), 4.80 (d, *J* = 7.0, 1H), 3.38 (s, 3H).

(*E*)-(3-ethoxyprop-1-ene-1,3-diyl)dibenzene (10l)

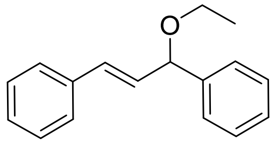

10l

From (*E*)-1,3-diphenylallyl acetate (25.2 mg, 0.1 mmol) and ethanol (12.0 μL, 0.2 mmol), following the general procedure, compound **10l** (8.8 mg, 0.037 mmol) was obtained in 37% yield (*Z*:*E* ratio = 6:94) as a yellow oil. The crude product was purified by column chromatography. Eluent: Cyclohexane: DCM (95:5). Spectroscopic data are in agreement with the published data.[30] **^{1}H-NMR (300 MHz, $CDCl_3$)** δ 7.44 – 7.18 (m, 10H), 6.60 (d, *J* = 15.8 Hz, 1H), 6.31 (dd, *J* = 15.8, 7.0 Hz, 1H), 4.92 (d, *J* = 7.0 Hz, 1H), 3.59 (dq, *J* = 9.1, 7.0 Hz, 1H), 3.48 (dq, *J* = 9.1, 7.0 Hz, 1H), 1.26 (t, *J* = 7.0 Hz, 3H).

(*E*)-(3-isobutoxyprop-1-ene-1,3-diyl)dibenzene (10m)

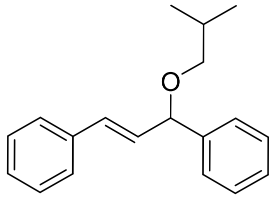

10m

From (*E*)-1,3-diphenylallyl acetate (25.2 mg, 0.1 mmol) and 2-methylpropan-1-ol (18.0 μL, 0.2 mmol), following the general procedure, compound **10m** (9.1 mg, 0.034 mmol) was obtained in 34% yield (*Z*:*E* ratio = 13:87) as a yellow oil. The crude product was purified by column chromatography. Eluent: Cyclohexane: DCM (95:5). Spectroscopic data are in agreement with the published data.[31] **^{1}H-NMR (300 MHz, $CDCl_3$)** δ 7.45 – 7.20 (m, 10H), 6.61 (d, *J* = 15.9 Hz, 1H), 6.29 (dd, *J* = 15.9, 6.9 Hz, 1H), 4.89 (d, *J* = 6.9 Hz, 1H), 3.30 (dd, *J* = 8.8, 6.7 Hz, 1H), 3.18 (dd, *J* = 8.8, 6.7 Hz, 1H), 2.02 – 1.87 (m, 1H), 0.94 (d, *J* = 6.7 Hz, 6H).

Supplementary Notes 2.

Laser flash photolysis studies of the reaction with photocatalysts 3e and 3g:

The employed laser flash photolysis equipment (λ_{exc} = 355 nm, power = 300-500 μJ/pulse) is based on a pump-probe setup purchased from Edinburgh Co (LP980-K). The pump source is an optical parametric oscillator (OPO) pumped by the third harmonic of a Nd:YAG laser (EKSPLA). The wavelength can be set from 210 nm to about 2600 nm, with a pulse width of about 5 nm using an OPO mod. NT342A-10 with an UV extension NT242 with typical pulse duration of 5 ns. A pulsed xenon flash lamp (150 W) is employed as detecting light source. A monochormator (TMS302-A, grating 150

lines/mm) disperses the probe light after it has passed the sample. The probe light is then passed on to a PMT detector (Hamamatsu Photonics) to obtain the temporal resolved picture. The time resolution in each window is about 10 % of the temporal window width. All components are controlled by the software L900 provided by Edinburgh.

Laser flash photolysis of a solution 50 µM of **3g** in CH_3CN under inert atmosphere resulted in the formation of two new peaks at 468 nm and 530 nm (Supplementary Figure 1a) that were assigned to the radical cation **[3g]**$^{\cdot+}$ and the triplet excited state **^{3}[3g]*** respectively (Supplementary Figure 1a). The latest was confirmed by disappearance of this band under aerobic conditions, due to triplet quenching by oxygen (Supplementary Figure 1b).

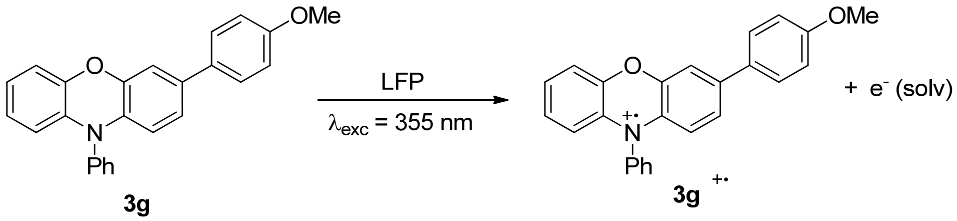

a) b)

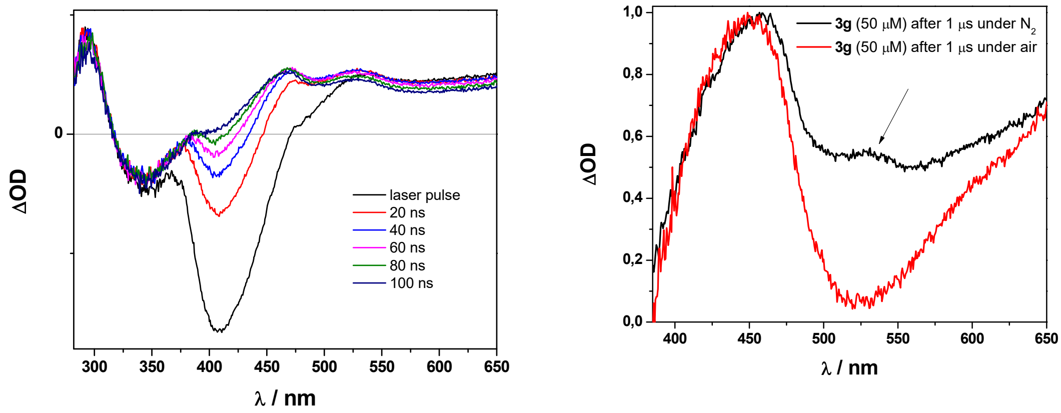

Supplementary Figure 1: **Reaction with photocatalyst 3g:** Laser flash photolysis (λ_{exc} = 355 nm, MeCN/Ar) experiments. a) Transient absorption spectra recorded at different times after the laser pulse of **3g** (50 µM); b) Transient absorption spectra recorded at 1 µs after the laser pulse of **3g** (50 mM) under N_2 (black) or under air (red).

Laser flash photolysis (λ_{exc} = 355 nm) of a solution containing **3g** (50 µM) and **1a** (70 mM) gave rise to the formation of two new intense peaks at 360 nm and 490 nm (see Supplementary Figure 2a), that were assigned to the radical intermediate **I** (**INT I**) and

the carbocation **II** (**INT II**) on the basis of the reported data (For the transient absorption spectrum of **I** and **II** see [32]). Thus, after single electron transfer (SET) from the singlet excited state (^{1}**3g***) to **1a** at diffusion-controlled rate ($k_q(S_1)$ = 4.9 x 10^9 $M^{-1}s^{-1}$), the corresponding radical ion pair (**3g**$^{\cdot+}$**------1a**$^{\cdot-}$) is generated. Fast acetate release from the **1a**$^{\cdot-}$ led to the formation of **INT I** which is still in contact with the radical cation of **3g** (**3g**$^{\cdot+}$**-----INT I)** At this point, two pathways could take place. On one hand, ultra-fast back electron transfer (BET) occurs between the **3g** radical cation (**3g**$^{\cdot+}$) and **INT I**, restoring **3g** to its ground state and generating free **INT II** which has been successfully observed by LFP (λ_{abs} = 490 nm, Supplementary Figure 2b). In order to whether **INT II** comes from **INT I** we have performed additional LFP experiments of **3g** in the presence of increasing amounts of **1a** (Supplementary Figure 2 c and d). Generation of **INT II** is practically instantaneous even at lower concentrations of **1a** Supplementary Figure 2d) whereas lifetimes of **INT I** is not affected by higher amounts of **1a** (Supplementary Figure 2c), what is indicating that the BET process undergoes in the sub-nanosecond scale (as well as the SET and the C-O bond scission).[33] The other possible evolution pathway of (**3g**$^{\cdot+}$**-----INT I)** is that they diffuse apart (escape process), forming their corresponding free radical ion and free radical, respectively. These two stable species are actually detected by the LPF experiments with lifetimes in the microsecond scale (see Supplementary Figure 2b and c) and it seems that they do not interact (see Supplementary Figure 2b and c).

a)

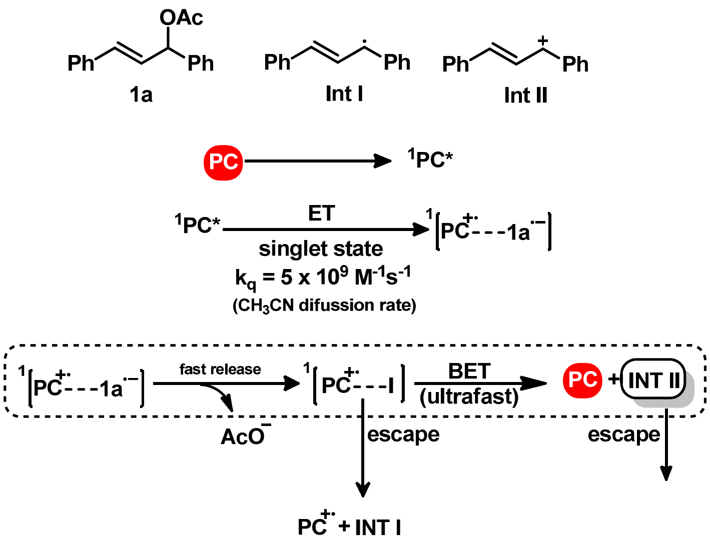

b)

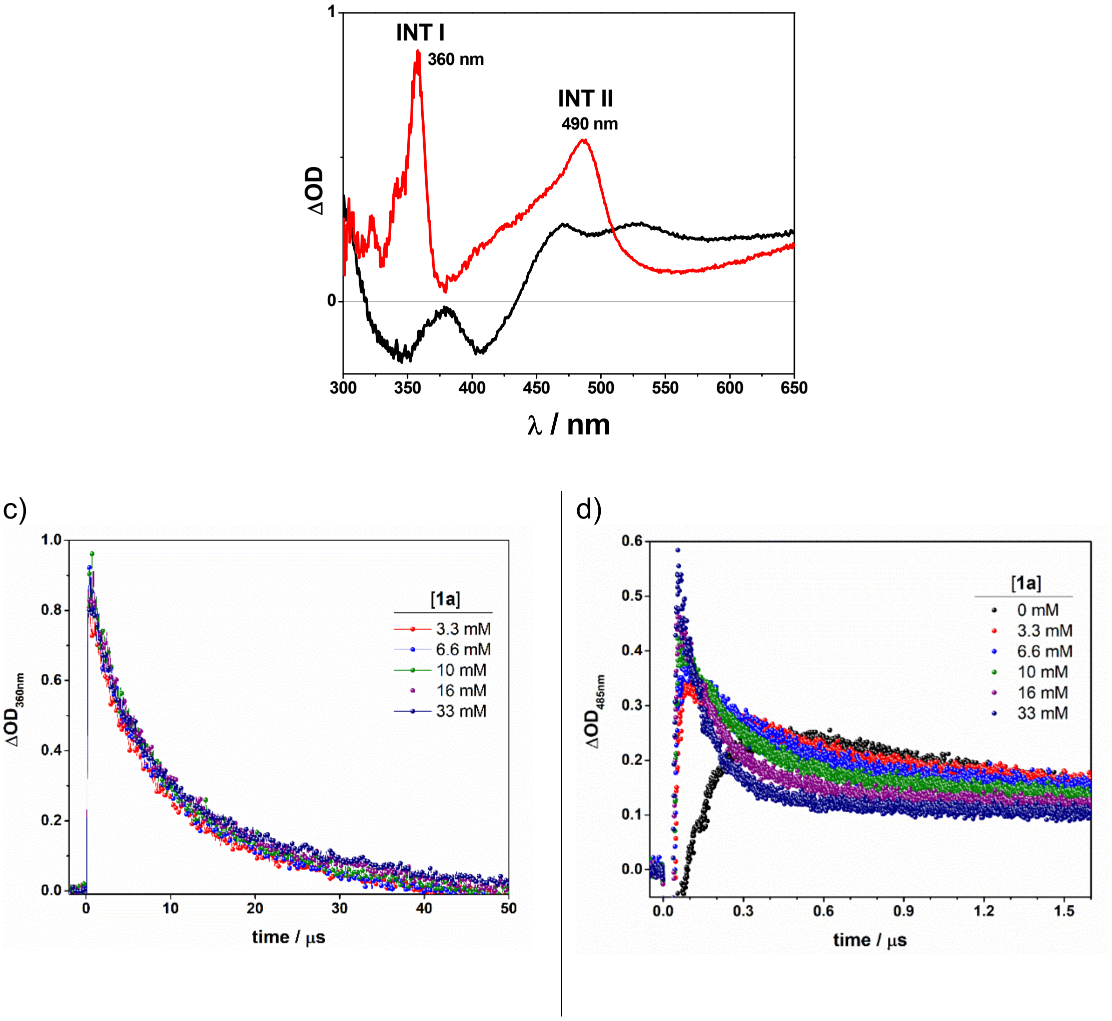

Supplementary Figure 2. a) Proposed reaction mechanism; b) Transient absorption spectra recorded 40 ns after the laser pulse (λ_{exc} = 355 nm, MeCN/Ar) of **3g** (50 μM) without **1a** (black), with 70 mM of **1a** (red); c) Normalized decays monitored at 357 nm of **3g** (50 mM), with increasing concetrations of 1a; d) Normalized decays monitored at 485 nm of **3g** (50 mM), with increasing concetrations of **1a.**

Having established the formation of **INT I** and **INT II**, the question arises whether nucleophiles such as pyrrole (**2a**) or *p*-toluidine (**8b**) are capable of reacting with these intermediates, which would require direct quenching of **INT I** or **INT II** by **2a** (or **8b**). In fact, addition of **2a** (37 mM) or **8b** (40 mM) to the **3g**/**1a** mixture resulted in a totally disappearance of the band at 490 nm, while the band at 360 nm is not affected (Supplementary Figure 3), clearly confirming that **INT II** is the reactive species.

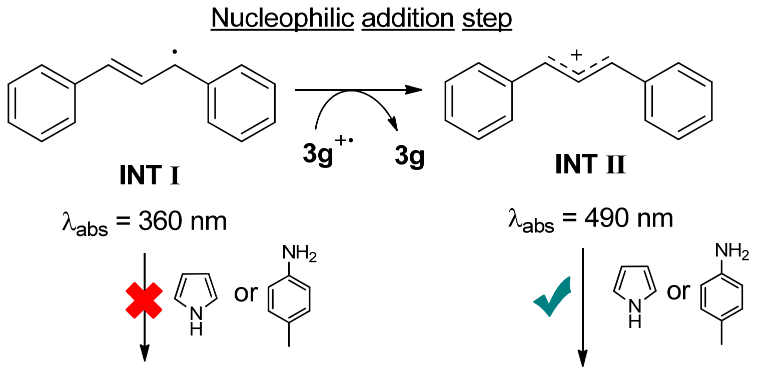

a) Reaction with pyrrole **2a**

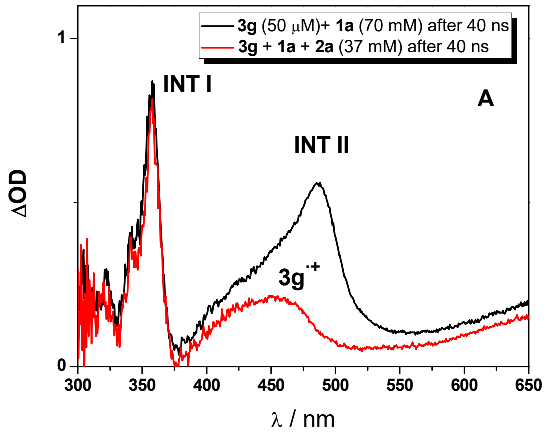

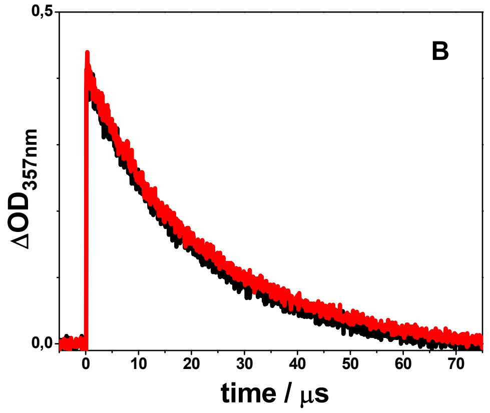

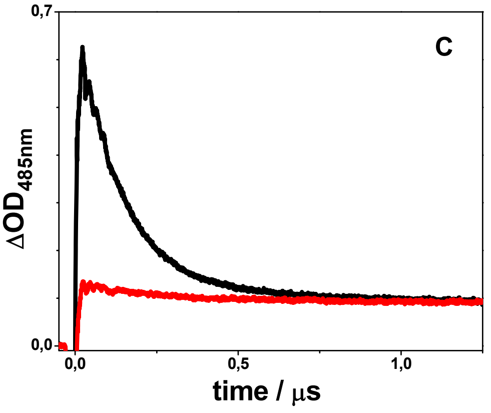

b) Reaction with *p*-toluidine **8b**

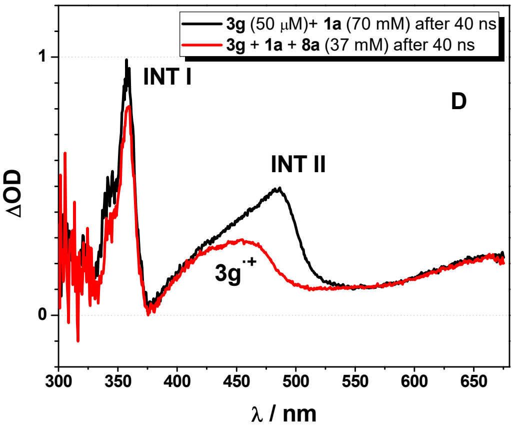

E

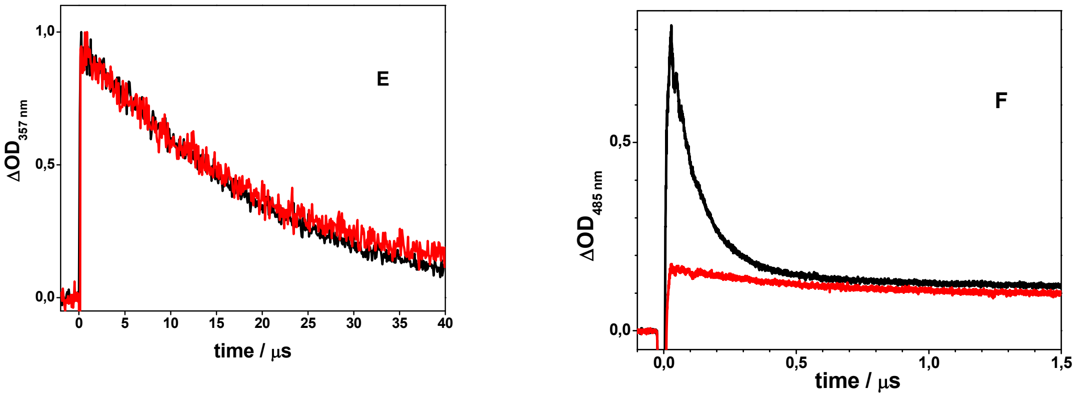

Supplementary Figure 3. a) LFP of the quenching with pyrrole **2a**: A: Transient absorption spectra recorded 40 ns after the laser pulse (λ_{exc} = 355 nm, MeCN/Ar) of **3g** (50 µM) with 70 mM of **1a** (black), and in the presence of **2a** (40 m M) (red). B: Decay at 357 nm of **3g** (50 mM) and **1a** (70 mM) (black) and in the presence of **2a** (37 mM) (red). C: Decay at 490 nm of **3g** (50 mM) and **1a** (70 mM) (black) and in the presence of **2a** (37 mM) (red); b) LFP of the quenching with *p*-toluidine **8b**: D: Transient absorption spectra recorded 40 ns after the laser pulse (λ_{exc} = 355 nm, MeCN/Ar) of **3g** (50 µM) with 70 mM of **1a** (black), and in the presence of **8b** (37 mM) (red). E: Decay at 357 nm of **3g** (50 mM) and **1a** (70 mM) (black) and in the presence of **8b** (37 mM) (red). F: Decay at 490 nm of **3g** (50 mM) and **1a** (70 mM) (black) and in the presence of **8b** (37 mM) (red).

Laser flash photolysis (λ_{exc} = 355 nm) of a solution containing **3g** (50 µM) and the **allylic benzoate 1** (70 mM) was also performed in order to compare the lifetime of the carbocation compared to the one obtained with the allylic acetate (Supplementary Figure 4). The experiment gave rise to the formation of the same two intense peaks at 360 nm and 490 nm (see Supplementary Figure 4b), corresponding again to the radical

intermediate **I** (**INT I**) and the carbocation **II** (**INT II**). Supplementary Figure 4b shows a comparison of the life time of **INT II** formed from the allylic acetate **1a** and the carbocation formed from the **allylic benzoate 1**. As it can be seen, the life time of the carbocation is dependent on the nucleophilic character of the anion released after the SET step. In the presence of a more nucleophilic anion (AcO^-) the life time of **INT II** is shorter than in the presence of the less nucleophilic benzoate ($PhOCO^-$).

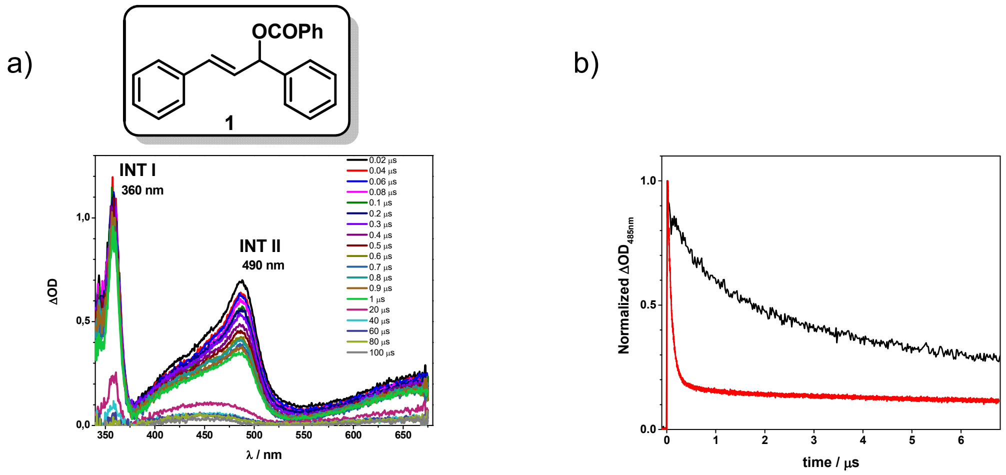

Supplementary Figure 4. a) Transient absorption spectra of **3g** (50 mM) and 70 mM of **"allylic benzoate 1"** recorded at different times after the laser pulse; b) Normalized decays monitored at 485 nm of **3g** (50 mM), with 70 mM of "**allylic benzoate 1**" (black), or with 70 mM of **1a** (red).

<u>Reaction with photocatalyst 3e</u>:

Laser flash photolysis (λ_{exc} = 355 nm) of a solution 50 µM of **3e** in CH_3CN under inert atmosphere resulted in the formation of two a new peak at 460 nm corresponding to the triplet excited state **^{3}[3g]**, confirmed by disappearance of the signal when carrying out the experiment under air (Supplementary Figure 5).

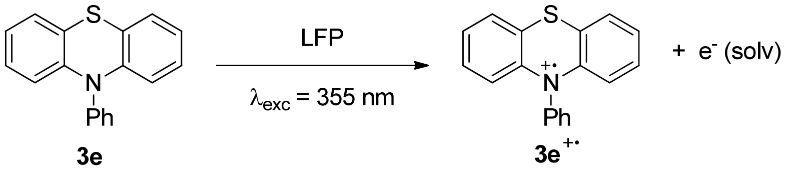

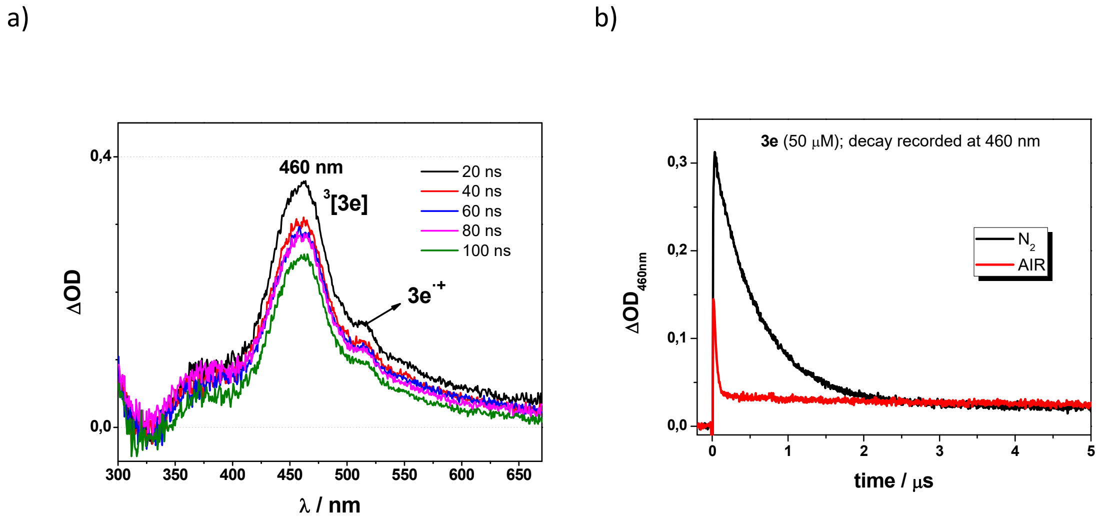

Supplementary Figure 5: a) Transient absorption spectra recorded at different times after the laser pulse of **3e** (50 μM) in CH_3CN; b) Decay at 460 nm of **3e** (50 mM) under N_2 (black) and under air (red).

Laser flash photolysis (λ_{exc} = 355 nm) of a solution containing **3e** (50 μM) and **1a** (70 mM) revealed the formation of two new absorption bands at 360 nm and 490 nm corresponding, as in the previous case, to the formation of the radical intermediate **I** (**INT I**) and the carbocation **II** (**INT II**) (see Supplementary Figure 6a).[32]

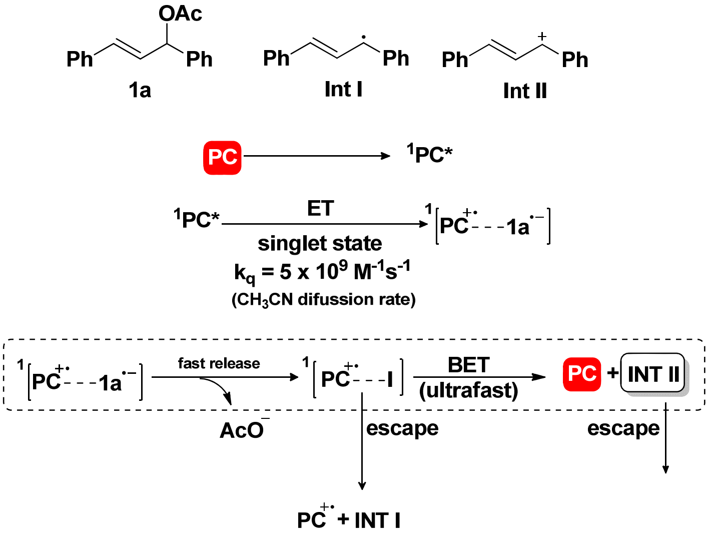

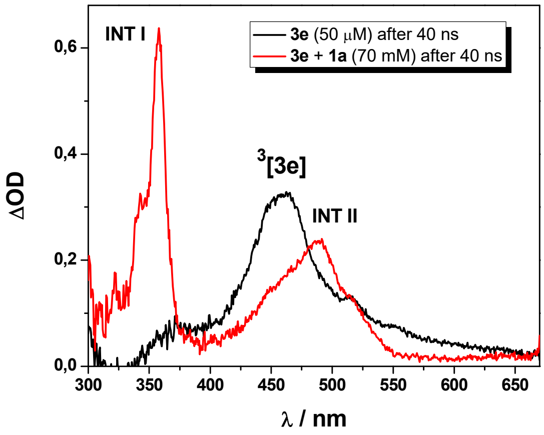

Supplementary Figure 6. Transient absorption spectra recorded 40 ns after the laser pulse (λ_{exc} = 355 nm, MeCN/Ar) of **3e** (50 µM) without **1a** (black) and with **1a** (70 mM, red).

As in the reaction with photocatalyst **3g** (Supplementary Figure 7), in the presence of pyrrole **2a** (37 mM) or *p*-toluidine **8b** (40 mM), the transient absorption spectrum shows disappearance of the peak at 490 nm while the band at 360 nm is not affected, confirming that the reactive intermediate is the carbocation intermediate **II** (**INT II**).

3rd step of the mechanism

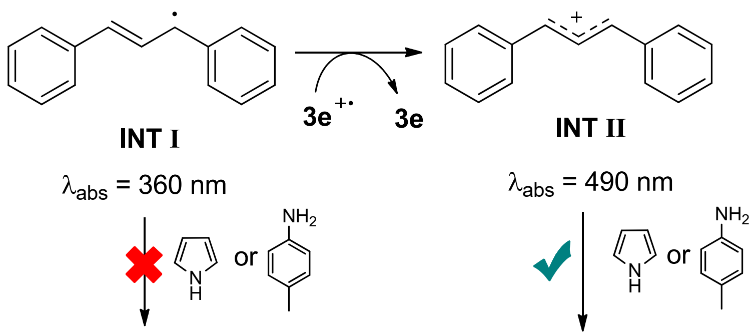

a) Reaction with pyrrole **2a**

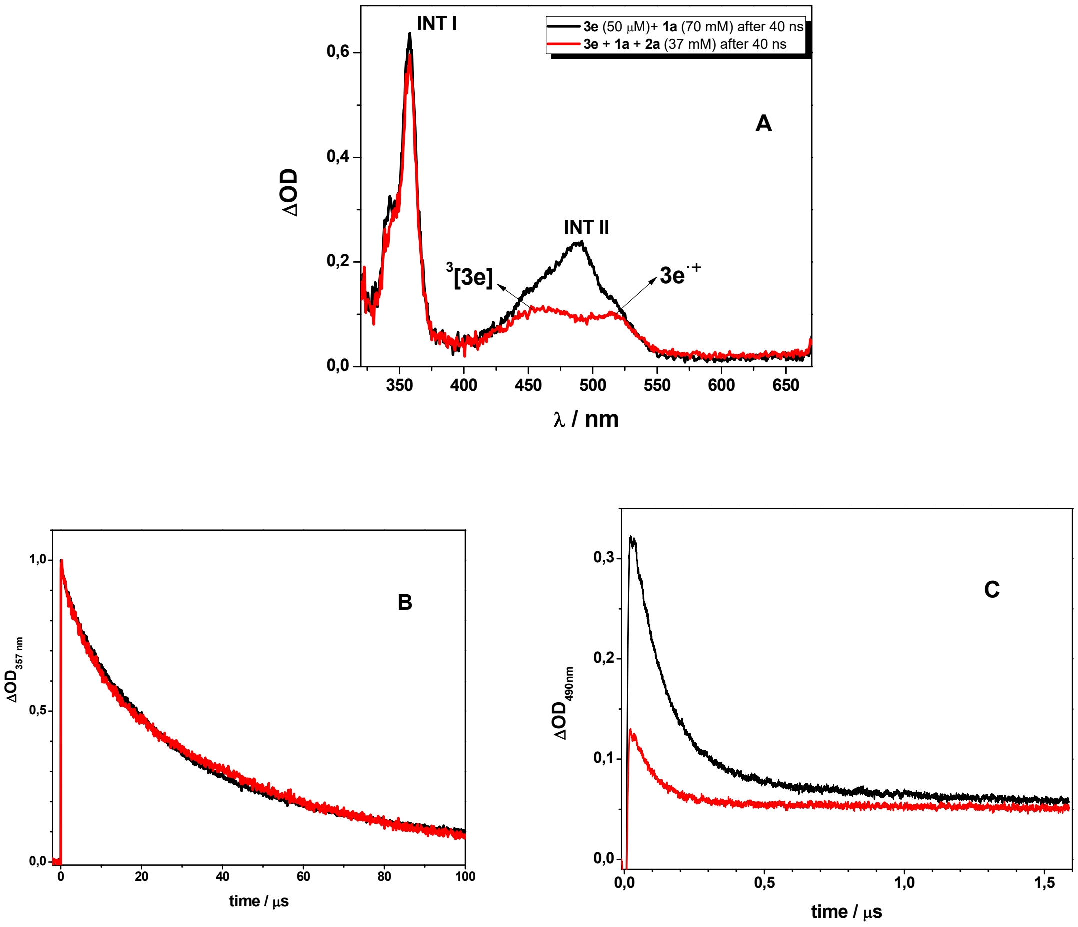

b) Reaction with *p*-toluidine **8b**

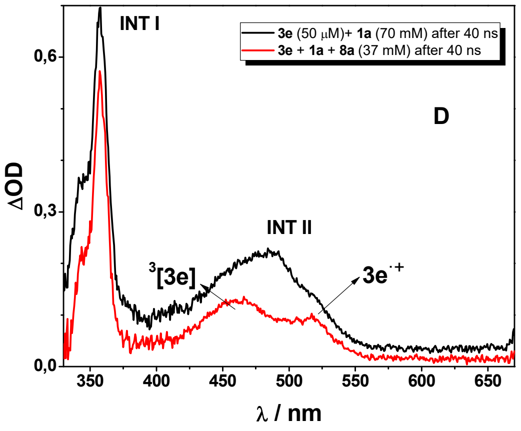

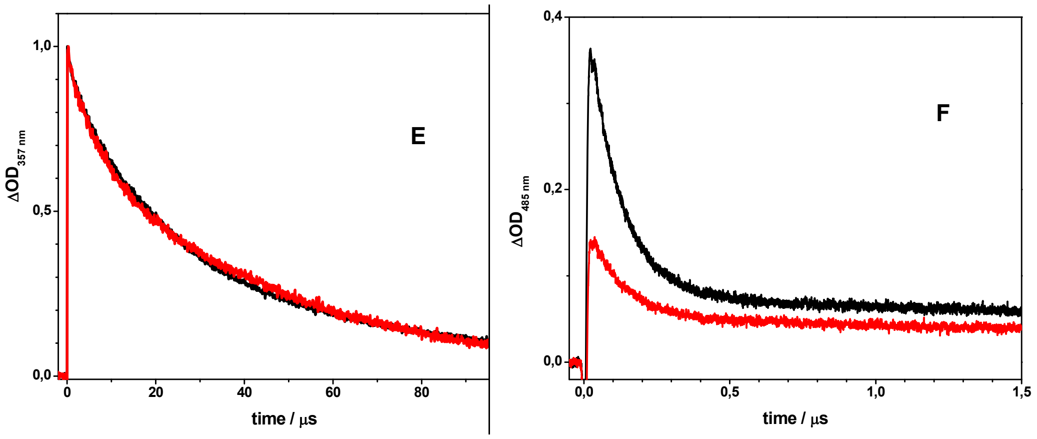

Supplementary Figure 7. a) LFP of the quenching with pyrrole **2a**: A: Transient absorption spectra recorded 40 ns after the laser pulse (λ_{exc} = 355 nm, MeCN/Ar) of **3e** (50 μM) with 70 mM of **1a** (black), and in the presence of **2a** (40 m M) (red). B: Decay at 357 nm of **3e** (50 mM) and **1a** (70 mM) (black) and in the presence of **2a** (37 mM) (red). C: Decay at 490 nm of **3e** (50 mM) and **1a** (70 mM) (black) and in the presence of **2a** (37 mM) (red); b) LFP of the quenching with *p*-toluidine **8b**: D: Transient absorption spectra recorded 40 ns after the laser pulse (λ_{exc} = 355 nm, MeCN/Ar) of **3e** (50 μM) with 70 mM of **1a** (black), and in the presence of **8b** (37 mM) (red). E: Decay at 357 nm of **3e** (50 mM) and **1a** (70 mM) (black) and in the presence of **8b** (37 mM) (red). F: Decay at 490 nm of **3e** (50 mM) and **1a** (70 mM) (black) and in the presence of **8b** (37 mM) (red).

Supplementary Notes 3:

Absorption spectrum of 1a, 3e and 3g:

The absorption spectrum of a solution of the different compounds in CH_3CN was measured using a quartz cuvette with 1 cm of optical pathway.

a)

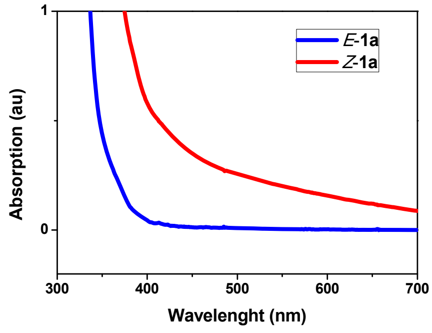

b)

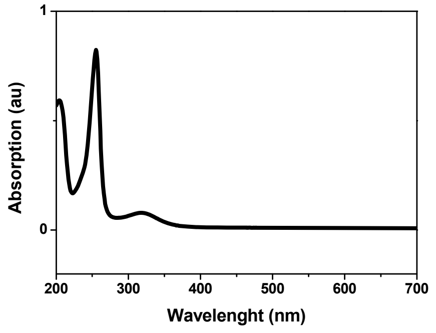

c)

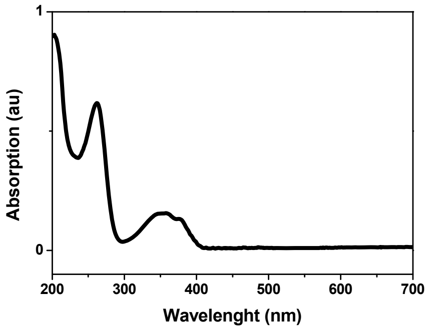

d)

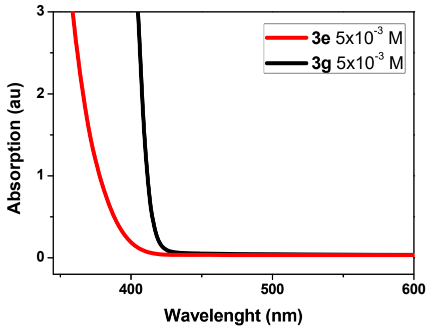

Supplementary Figure 8. a) Absorption spectrum of a 0.1 M solution of ***E*-1a** (blue line) and ***Z*-1a** (red line) in acetonitrile; b) Absorption spectrum of a 0.17 mM solution of **3e** in acetonitrile; c) Absorption spectrum of 0.1 mM solution of **3g** in acetonitrile; d) Absorption spectrum of **3e** and **3g** at the concentration of the reaction (5 mM) in acetonitrile.

Fluorescence quenching studies:

For the steady-state and time resolved fluorescence quenching studies with photocatalyst **3g**, increasing concentrations of quencher were added to a solution 100 μM of **3g** in CH_3CN under N_2 atmosphere (λ_{exc} = 372 nm).

a)

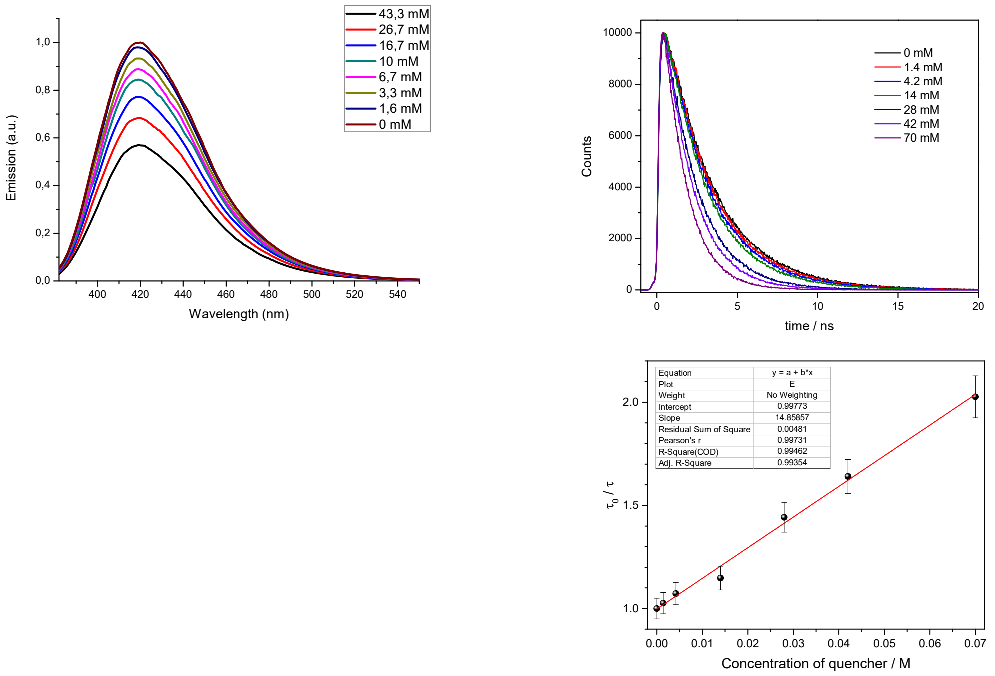

k_q (S_1) = 4.7 x 10^9 $M^{-1}s^{-1}$

b)

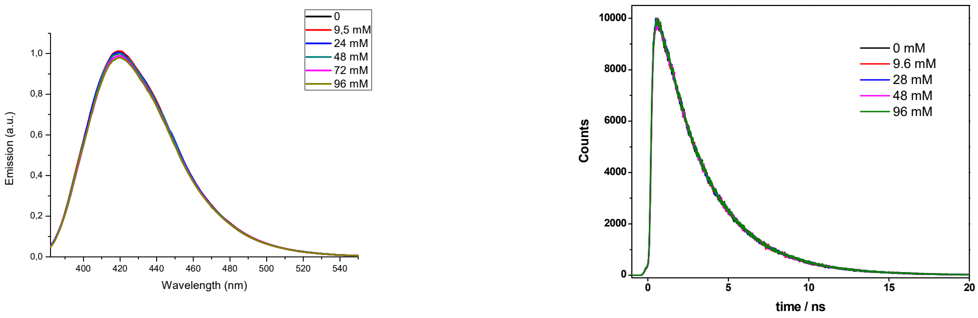

c)

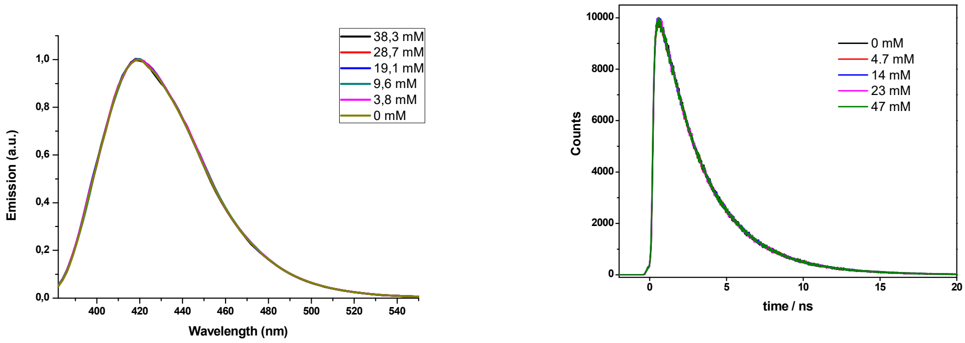

d)

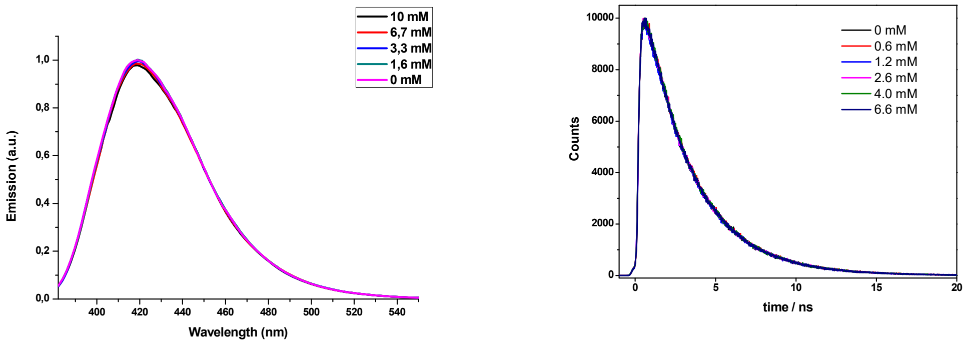

Supplementary Figure 9. Steady-state and time resolved fluorescence quenching of **3g** with increasing concentrations of a) ***E*-1a**, Stern-Volmer plot to obtain $k_q(S_1)$; b) **2a**; c) DIPA, d) *p*-Toluidine

For the steady-state fluorescence quenching studies with photocatalyst **3e** increasing concentrations of quencher were added (up to 5 x 10^{-4} M) to a solution of **3e** in CH_3CN with absorbance 0.1 at the excitation wavelength (λ_{exc} = 350 nm) under N_2 atmosphere.

a)

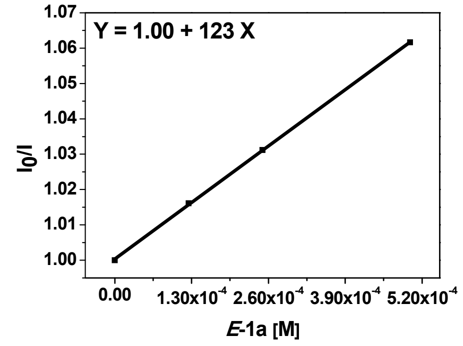

b)

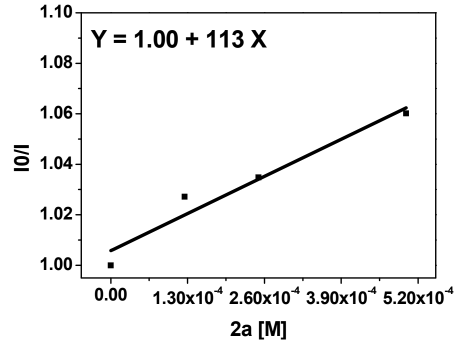

c)

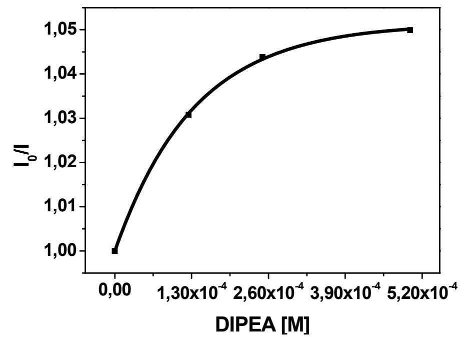

d)

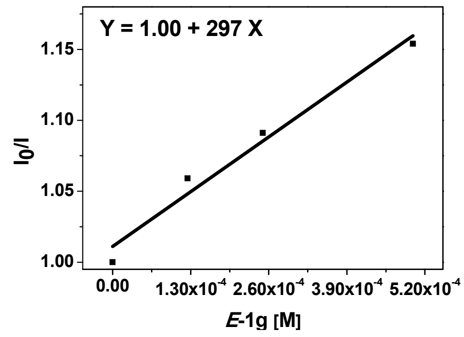

e)

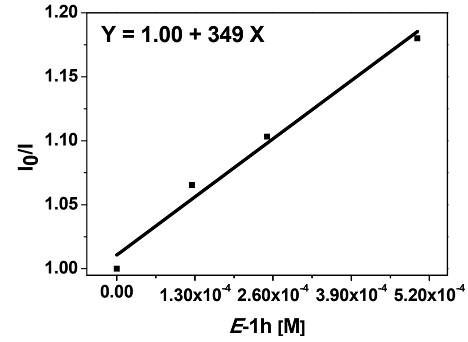

f)

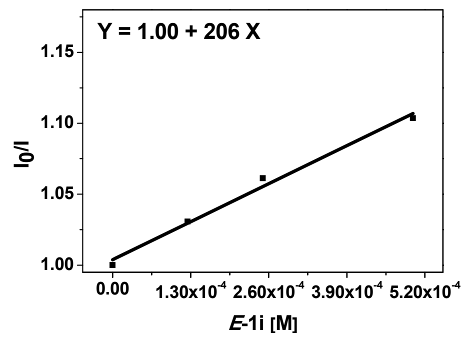

g)

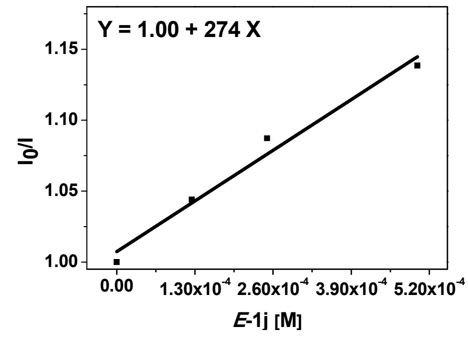

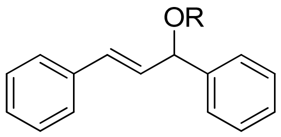

1f, R= COPh
1g, R= CO_2Et
1h, R= $CONMe_2$
1i, R= OH

Supplementary Figure 10. Stern-Volmer equations of the steady-state fluorescence quenching of **3e** with increasing concentrations of a) ***E*-1a**; b) **2a**; c) DIPEA; d) ***E*-1g**; e) ***E*-1h**; f) ***E*-1i**; g) ***E*-1j**.

Excited state energy of 3e and 3g:

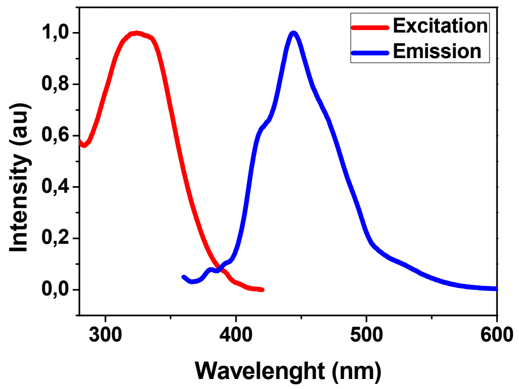

Supplementary Figure 11. Normalized emission and excitation spectrum of **3e**.

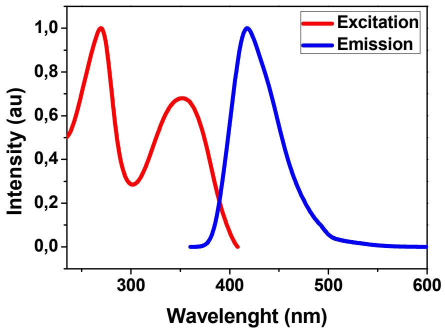

Supplementary Figure 12. Normalized emission and excitation spectrum of **3g**.

The singlet excited state energy of the photocatalysts [E_{0-0}(**3*/3**)] were calculated from the intersection between the emission and excitation spectrum applying the Supplementary Equation 1:

$$E = N * \frac{h\,c}{\lambda} = 6.022 * 10^{23} mol^{-1} \frac{6.63 \cdot 10^{-34} J \cdot s * 3 \cdot 10^{8} (m/s)}{\lambda\,(m)}$$

Supplementary Equation 1

E_{0-0} S_1(**3e*/3e**) = 73.7 Kcal/mol = 3.19 eV

E_{0-0} S_1(**3g*/3g**) = 73.5 Kcal/mol = 3.18 eV

The triplet excited state energy of the photocatalysts [E_{0-0}(**3*/3**)] were obtained by DFT calculations (see section 13.3)

E_{0-0} T_3(**3***/**3**) = -2.8 eV

Supplementary Notes 4.

Cyclic Voltametry

CV measurements were carried out under argon atmosphere. The measurement were performed in MeCN containing 0.1 M tetra *n*-butylammonium tetrafluoroborate. A glassy carbon electrode (working electrode), platinum wire counter electrode, and Ag/AgCl reference electrode was employed for the CV measurement. The scan rate was 50 mV/s, a step potential of 5.0 mV was applied.

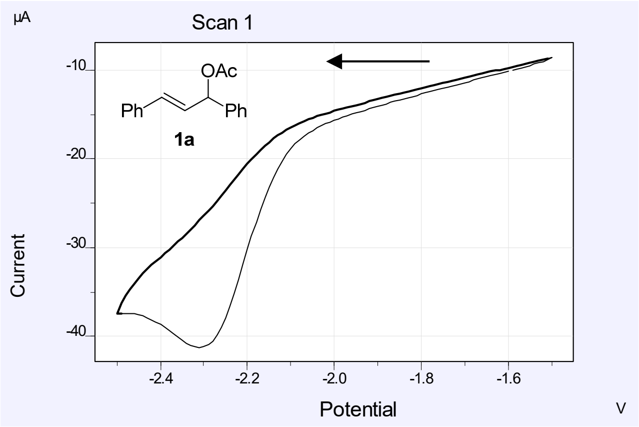

Supplementary Figure 13. Cyclic voltammetry of **1a**, E (**1a**/**1a**$^{\bullet-}$) = - 2.35 eV vs SCE.

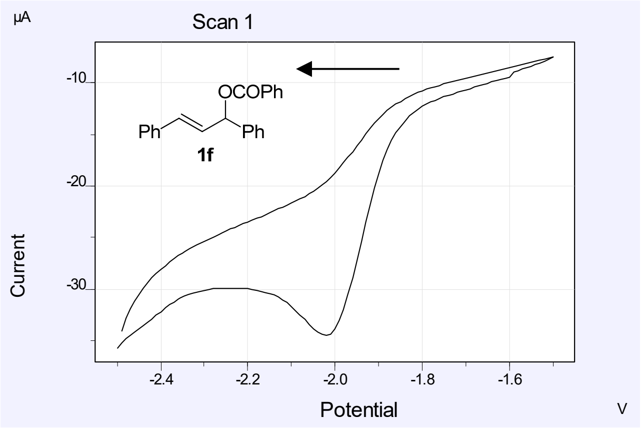

Supplementary Figure 14. Cyclic voltammetry of **1f**, E (**1f**/**1f**$^{\bullet-}$) = - 2.06 eV vs SCE.

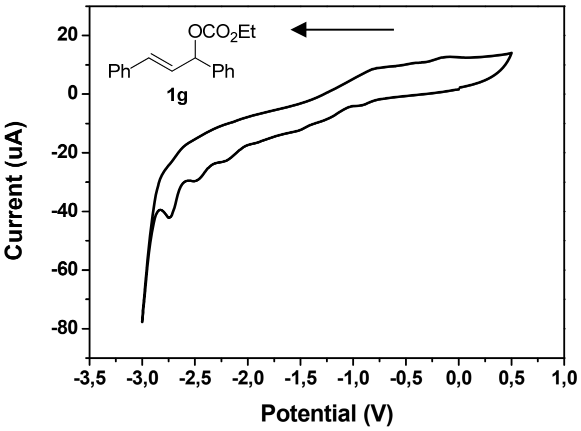

Supplementary Figure 15. Cyclic voltammetry of **1g**, E (**1g**/**1g**$^{\bullet-}$) = - 2.27 eV vs SCE.

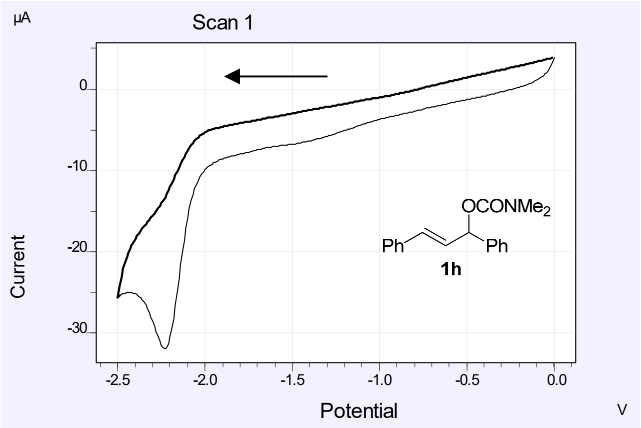

Supplementary Figure 16. Cyclic voltammetry of **1h**, E (**1h**/**1h**$^{\bullet-}$) = - 2.27 eV vs SCE.

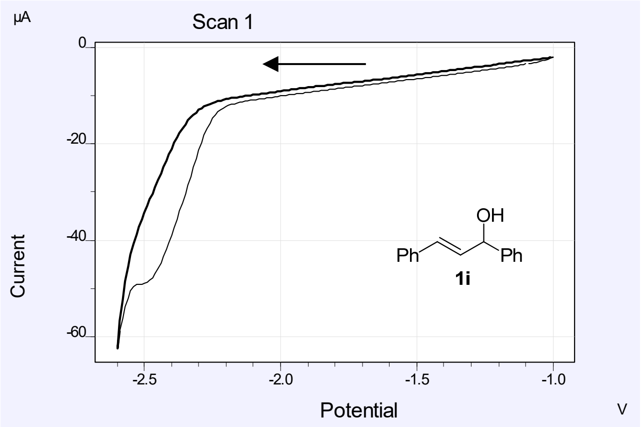

Supplementary Figure 17. Cyclic voltammetry of **1i**, E (**1i**/**1i**$^{\bullet-}$) = - 2.52 eV vs SCE.

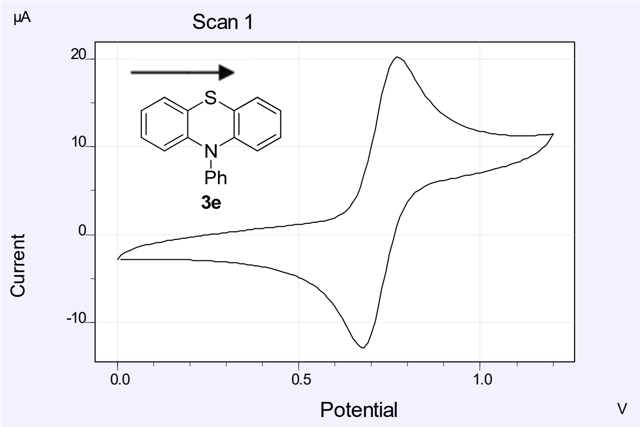

Supplementary Figure 18. Cyclic voltammetry of **3e**, E ($\mathbf{3e}^{\bullet+}$/**3e**) = 0.68 eV vs SCE.

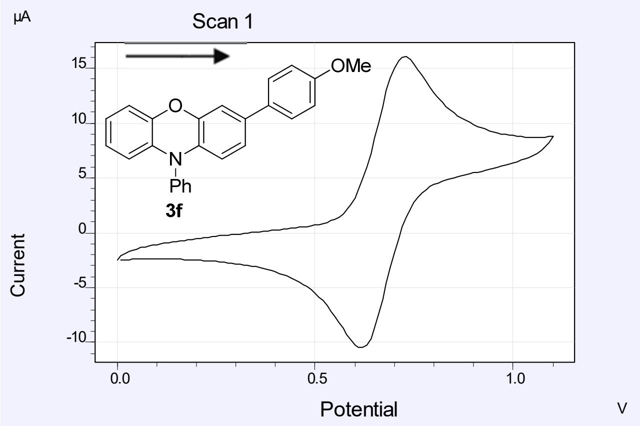

Supplementary Figure 19. Cyclic voltammetry of **3g**, E ($\mathbf{3g}^{\bullet+}$/**3g**) = 0.63 eV vs SCE.

Determination of the reduction redox potential of the excited photocatalyst (E*red) and Rhem-Weller equation:

The reduction redox potential of the excited photocatalyst (**3e** and **3g**) was subsequently determined. Indeed, knowing the electrochemical oxidation peak potential and estimating spectroscopically the excited state energy of them [$E_{0\text{-}0}$(**3***/**3**)] from the intersection between the emission and excitation spectrum, the value of E^{*}red(**3**) = E($\mathbf{3}^{\bullet+}$/**3***) can be obtained from the Supplementary Equation 2.[34]

$$E(\mathbf{3}^{\bullet+}/\mathbf{3}^{*}) = E(\mathbf{3}^{\bullet+}/\mathbf{3}) - E_{0\text{-}0}\,S_1(\mathbf{3}^{*}/\mathbf{3})$$

Supplementary Equation 2

E*red($\mathbf{3e}^{\bullet+}$/**3e***) = 0.68 – 3.19 = -2.51 V (vs SCE)

E*red($\mathbf{3g}^{\bullet+}$/**3g***) = 0.63 – 3.18 = -2.55 V (vs SCE)

To calculate the free Gibbs energy of the reaction the Rhem-Weller equation was used:

$$\Delta G^{o}_{et}(eV) = (E^{o}_{red}(PC^{*}/PC^{\cdot -}) - E^{o}_{red}(D^{\cdot +}/D))$$

Supplementary Equation 3

$\Delta G_{\mathbf{1a/3e}}$ = -2.51 – (-2.35) = -0.16 eV = -3.6 Kcal/mol

$\Delta G_{\mathbf{1a/3g}}$ = -2.55 – (-2.35) = -0.2 eV = -4.6 Kcal/mol

The free Gibbs energy for the SET form the triplet excited state of **3** was also calculated:

$E_{0\text{-}0}$ T_3(**3***/**3**) = -2.8 eV

E*red T_3 (**3e**$^{\cdot +}$/**3e***) = 0.68 – 2.8 = -2.12 V (vs SCE)

$\Delta G_{\mathbf{1a/3}}$ = -2.12 – (-2.35) = 0.23 eV = 3.6 Kcal/mol

According to the Rhem-Weller equation, the SET from the triplet excited state is an unfavourable process.

Supplementary Notes 5.

Determination of the Quantum Yield

A solution of ferrioxalate was chosen as actinometer following the procedure described by the IUPAC (subcommittee on photochemistry).[35] The procedure is based on the decomposition under irradiation of ferric ions to ferrous ions which are complexed by 1,10-phenanthroline. This photochemical transformation has a known quantum yield and the complexation of Fe^{2+} with 1,10-phenanthroline can be monitored by UV-Visible absorption since its extinction coefficient at 510 nm is known (=11100 M^{-1} cm^{-1}). Therefore, the moles transformed can be related with the moles of photons absorbed by the Supplementary Equation 4.

$$\Phi = \frac{mol\ transformed}{photons\ absorbed}$$

Supplementary Equation 4

The complete procedure should be done under a red safe-light environment. At 420 nm ferroxilate has a Φ = 1.05.[36] 0.006, 0.012, or 0.15 M solutions of $K_3[Fe(C_2O_4)_3]$ $3H_2O$ can be used for actinometry. In this case, we chose a concentration of 0.15 M. The solutions were prepared and stored in a dark laboratory:

1. Potassium ferrioxalate solution (0.15 M): 368.4 mg of $K_3[Fe(C_2O_4)_3]$ $3H_2O$ (commercially available) and 26.6 μL of H_2SO_4 were added into a 5 mL volumetric flask and filled to the mark with Milli-Q water.

2. Phenanthroline solution (0.15 M): 1.35 g of 1,10-phenantroline monohydrate were added to 50 mL volumetric flask and filled to the mark with MilliQ water.

3. Buffer solution: 4.94 g of NaOAc and 1 mL of H_2SO_4 were added to 100 mL volumetric flask and filled to the mark with MilliQ water.

4. Model reaction solution: A vial equipped with a magnetic stir bar was charged with the corresponding allylic compound **1** (0.1 mmol), pyrrole (0.2 mmol), **3g** (1.7 mg, 5 mol%), DIPA (86 μL, 0.5 mmol) and acetronitrile (1 mL) and fitted with a teflon screw cap septum. The reaction was degassed with three freeze-pump-thaw cycles. The vial was then backfilled with N_2 and stirred under 420 nm LED irradiation (18.3396 W/m^2 intensity; approximate distance was 2 cm from the vial) at 20ºC.

Actinometry procedure: Due to the reactor setup (Supplementary Figure 20), the simultaneous irradiation of both the actinometer solution and model reaction is not feasible. However, the stability of the irradiation light was checked through radiometer measurements (from spectro-radiometer equipment Stellarnet model Blue-Wave UV-NB50). Therefore, we assumed that consecutive measurements of both actinometer and model reaction are comparable. In addition, using the same spectrometer, the LED source spectrum was measured, detecting a maximum wavelength of emission of 418 nm (Supplementary Figure 21).

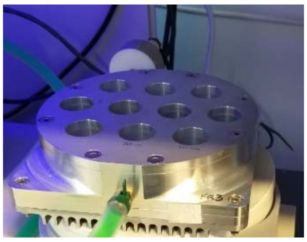

Supplementary Figure 20. LED setup of the reaction

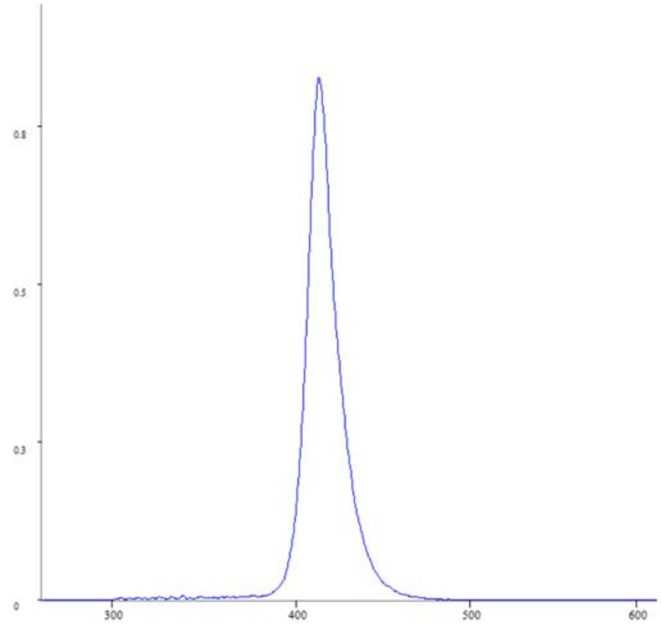

Supplementary Figure 21. Emission spectrum of the blue LED of the photochemical reactor (λ_{max}= 415 nm).

2 mL of potassium ferrioxalate solution (0.15 M) were introduced into the photoreactor under dark conditions while being stirred. Then, the LED was switched on. Every 10 s the light was switched off and a 0.1 mL aliquot was taken. To each aliquot, 2 mL of buffer solution and 0.5 mL of 1,10-phenanthroline 0.15 M were added and the final volume was raised to 10 mL with MilliQ water. Then 83 μL of this solution were diluted to 5 mL with MilliQ water. As a blank sample, a solution was prepared with 0.1 mL of potassium ferrioxalate solution (0.15 M) before irradiation, 2 mL of buffer solution and 0.5 mL of 1,10-phenanthroline 0.15 M in a 10 mL of volumetric flask filled with water until the mark, and 83 μL of this solution were diluted to 5 mL with MilliQ water. The absorbance spectrum of each sample was monitored at 510 nm. The absorbance to each time was related with the photochemically produced Fe^{2+} ions across the Lambert-Beer Law (Supplementary Equation 5), where V_1 is the irradiated volume (noting that the initial volume is 2 mL but it changes as the aliquots are taken); V_2 is the aliquot volume (0.1 mL), V_3 is the final volume after addition of 1,10-phenanthroline and buffer (10 mL). b is referred to the optical pathway (1 cm), ΔA (510 nm) is the difference in absorbance between the irradiated solution and the blank sample, ε (510 nm) is the extinction coefficient of the complex formed by Fe(II) and 1,10-phenanthroline (ca. 11100 M^{-1} cm^{-1}).

$$moles\ of\ Fe^{2+} = \frac{V_1 \cdot V_3 \cdot \Delta A_{(510\ nm)}}{10^3 \cdot V_2 \cdot b \cdot \varepsilon_{(510\ nm)}}$$

Supplementary Equation 5

The moles of Fe^{2+} formed (x) are plotted as a function of time (t) (Supplementary Figure 21).

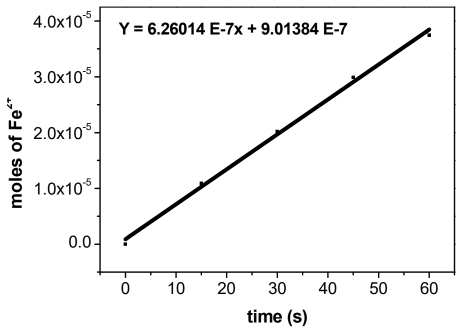

Supplementary Figure 22. Actinometer.

The slope of this line (*dx*/*dt*) was correlated to the moles of incident photons by unit of time ($q^0_{n,p}$) using the Supplementary Equation 6:

$$q^0_{n,p} = \frac{dx/dt}{\Phi(\lambda)\cdot[1-10^{-A(\lambda)}]}$$

Supplementary Equation 6

Where $\Phi_{(\lambda)}$ is the quantum yield of the actinometer reaction at the irradiated wavelength, in this case being 1.05 at 420 nm for 0.15 M dilution[35] and $A_{(\lambda)}$ is the absorbance of the actinometer solution (ferrioxalate) at the irradiated wavelength (415 nm). The absorbance at 415 nm was measured with an Agilent 8453 UV-visible Spectroscopy System using a quartz cuvette with 1 cm of optical pathway.

Therefore, the moles of incident photons by unit of time ($q^0_{n,p}$) was determined as 5.96 10^{-7} einstein s^{-1}.

The kinetics of the reaction under study were done as follows: the photoreactor (blue LEDs) was switched on and the reaction mixture was stirred. At 60, 90, 120, 150, and 180 minutes an aliquot of 0.1 mL was taken from the reaction mixture under a positive flow of nitrogen, and diluted with 0.6 mL of $CDCl_3$. Thus, the conversion of the reaction at the different indicated time was determined by ^1H NMR. Knowing the initial molar concentration, the determination of the moles of photo-converted product is possible.

Plotting the moles of product versus the irradiation time, the slope *dx*/*dt* can be related with the quantum yield across the equation [4] being equal to time ($q^0_{n,p}$) $\Phi_{(\lambda)}\cdot$[1-10-$A_{(\lambda)}$]. Therefore, the quantum yield at the wavelength of irradiation Φ (420 nm) can be calculated once A (420 nm) is determined. To measure A (420 nm), a model reaction

solution was added to a 1 cm optical pathway cuvette and the UV-Visible spectrum was recorded obtaining an absorbance of 0.19288.

Therefore the quantum yield for the reaction is: Φ = 0.015 = 1.5 %.

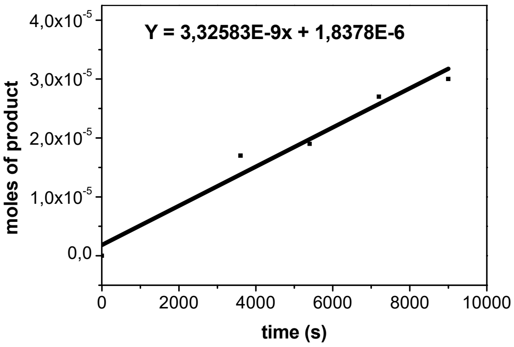

Supplementary Figure 23. Kinetic of the reaction.

Supplementary Notes 6.

General methods for DFT calculations:

Quantum chemistry calculations were carried out using the density functional theory (DFT). In particular, geometry optimizations were performed using the M06-2X functional[37] in combination with the 6-311G**[38] basis set including acetonitrile (ε = 37.5) solvent effects with the solvation model density (SMD).[39] All optimizations were performed without any geometrical constraint and harmonic vibrational frequencies have been also evaluated at the same level of theory to characterize minima and transition states in the potential energy surface. Transition states have been connected to products by optimization of geometries slightly modified from the transition states. All the calculations were performed using the Gaussian09 program.[40]

Calculation of redox potentials $E_{(Pc*/Pc-)}$.:

Redox potentials from excited state are calculated considering the potential value in the ground state, measured by electrochemical methods, and the energy of the S^1 excited state experimentally determined as the crossing point between the emission and the excitation spectrum. However, the reduction of photocatalysts in the ground state occur out of the practical range measurable with our experimental system. Therefore, we

theoretically estimated the energetics of reduction of **3e** and **3g** in acetonitrile by considering the energy differences between the photocatalyst in the ground state and its reduced form. The absolute potentials, expressed in eV, are shown bellow.

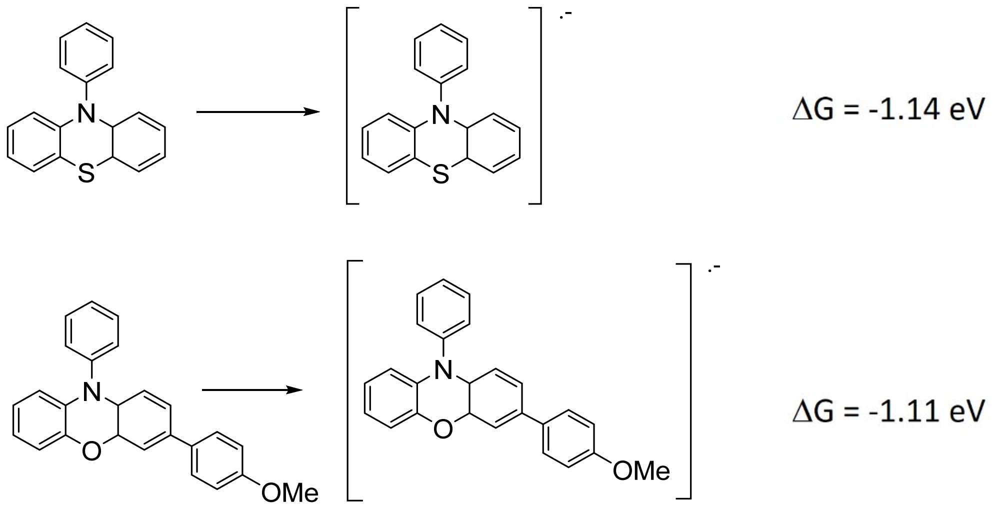

Supplementary Figure 24. Calculated redox potentials of **3e** and **3g**.

In order to convert the absolute potentials into values comparable to experimental results, we have to compare them with the known absolute potential of a common reference electrode. The IUPAC recommended absolute potential of NHE $E^{\ominus}(H^+/H_2)_{abs}$ value is 4.42 V.[41] Thus, redox potential for the reduction of **3e** and **3g** NHE are -3.28 V and -3.31V respectively. Considering that Saturated Calomel Electrode has a potential of +0.244 V vs NHE the values of reduction potentials of our photocatalysts vs SCE are: -3.52 V for **3e** and -3.55 V for **3g**. Effectively, such values are too negative and definitely should fall out of the measurable range in acetonitrile solution. Estimation of redox potential from excited state arise from consideration of the energy found for S_1. Such value is estimated to be 3.2 eV (see above), and therefore, the $E_{(Pc*/Pc-)}$ are found to be approximately -0.3 V vs SCE for both **3e** and **3g**.

Theoretical evaluation of the triplet excited state of 3e, 3g and 1a, and SOMO orbital of 1a.

The energy of the triplet excited state of **3e**, **3g** (Supplementary Figure 25A), *E*-**1a** and *Z*-**1a** (Supplementary Figure 25B) were calculated. According to the energies obtained, photosensitization of *E*-**1a** by both photocatalyst is feasible, while photosensitization of *Z*-**1a** cannot take place. Attending to the geometries of the excited states, the triplet excited state of *E*-**1a** presents an intermediate conformation between the *E*- and the *Z*-

isomer, and after relaxation its affords either the *E*- or the *Z*-**1a** in the ground state. However, the conformation of the *Z*-**1a** triplet excited state a does not change significantly compared to the ground state, therefore after relaxation its affords *Z*-**1a** exclusively. Therefore, under UV-light irradiation, there is an accumulation of the Z-**1a** isomer that cannot isomerize to the *E* isomer, and can only undergo the photoredox reaction.

Moreover, theoretical calculations show that the SOMO orbital either in the *E* or in the *Z* allylic acetate is centered in the double bond and the phenyl ring (Supplementary Figure 25C), confirming that the injection takes place in this part of the molecule. Here we attach the images to clear out the explanation.

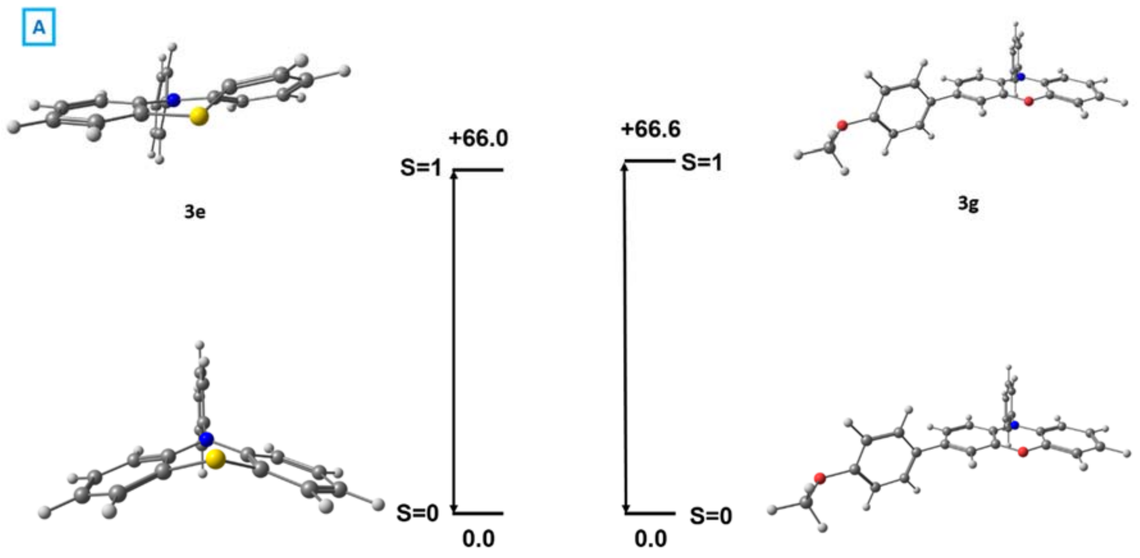

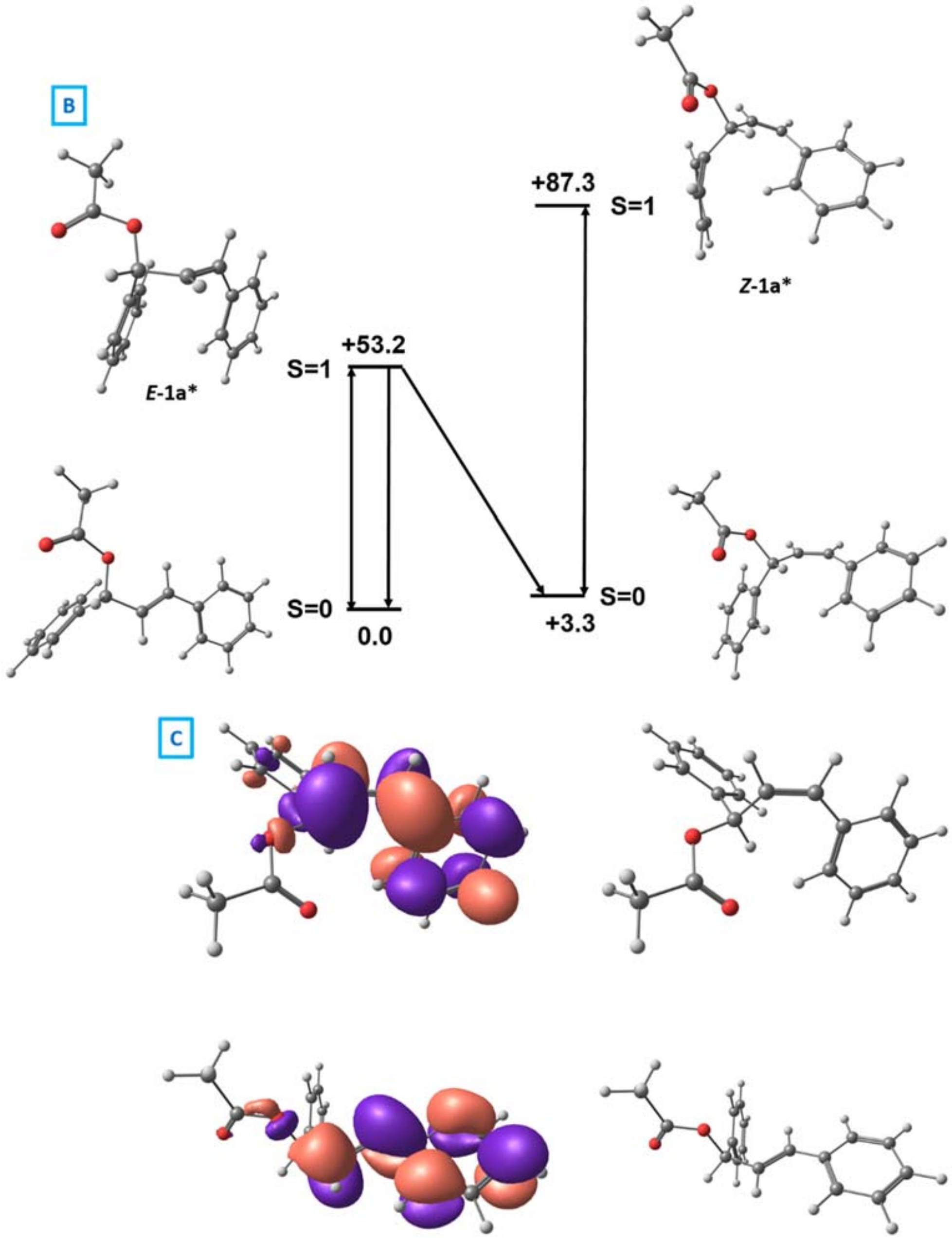

Supplementary Figure 25. A) Thermodynamic assesment of singlet-triplet gaps for both photocatalysts; B) Thermodynamic assesment of singlet-triplet gaps for Z and E substrates (energies in Kcal/mol); C) SOMO orbital in *E*-**1a** and *Z*-**1a.**

Theoretical evaluation of scission of acetate from anion radical generated from reduction of olefin 1a:

According experimental evidences, the photocatalyst undergoes a single electron transfer to **1a**, to afford the corresponding anion radical intermediate. According to theoretical results, such species evolves very fast through C-O bond scission to from the allyl radical and acetate anion. The very shallow kinetic barrier found ($\Delta G^{‡}$ = 1.4 kcal/mol)

and the considerable thermodynamic driving force observed (ΔG = -22 kcal/mol), suggest that such process is, in practice, concerted to electron transfer process and a very important driving force that trigger the whole photocatalytic transformation.

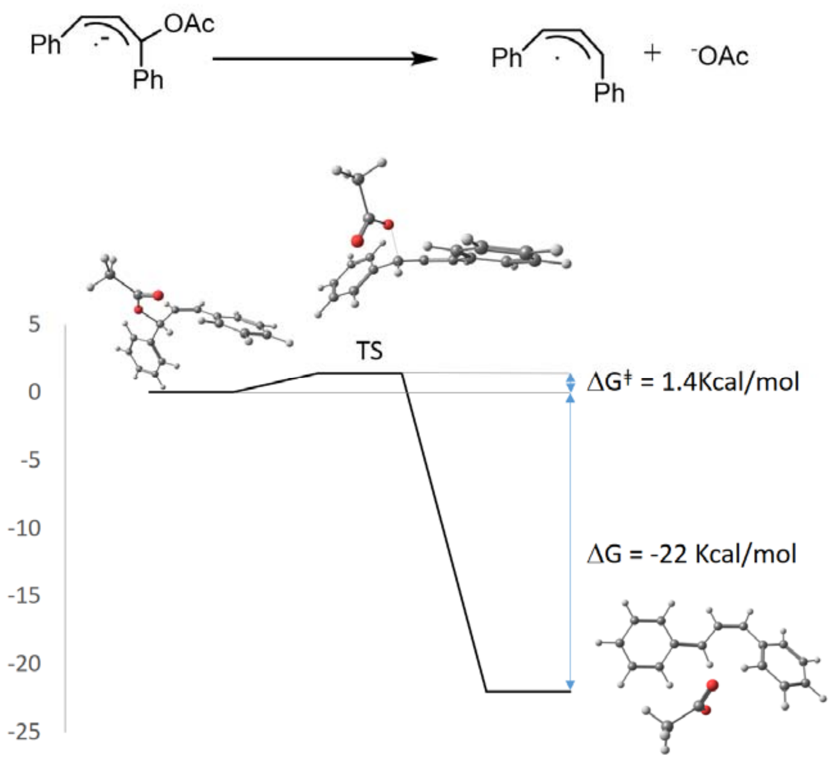

Supplementary Figure 26. Energetic profile for the scission of acetate from anion radical generated from **1a** reduction.

Calculation of energetics of redox reaction between allyl radical and oxidized photocatalysts:

Once allyl radical is formed by the scission of acetate, carbocation formation is the key step of the proposed mechanism. In accordance with experimental evidences for carbocation generation shown in the manuscript, calculations support the formation of such intermediate. Indeed, according to theoretical results the oxidation of allyl radical (**I**) by the oxidized photocatalyst (**PC**$^{+\cdot}$), results in the regeneration of the catalyst (**PC**) and formation of carbocationic intermediate (**II**). Such electron transfer has been evaluated by calculation of the different species involved separately. The thermodynamic balance shows that such process is favorable by -8.7 kcal/mol, for **3e** and -6.7 kcal/mol for **3g**.

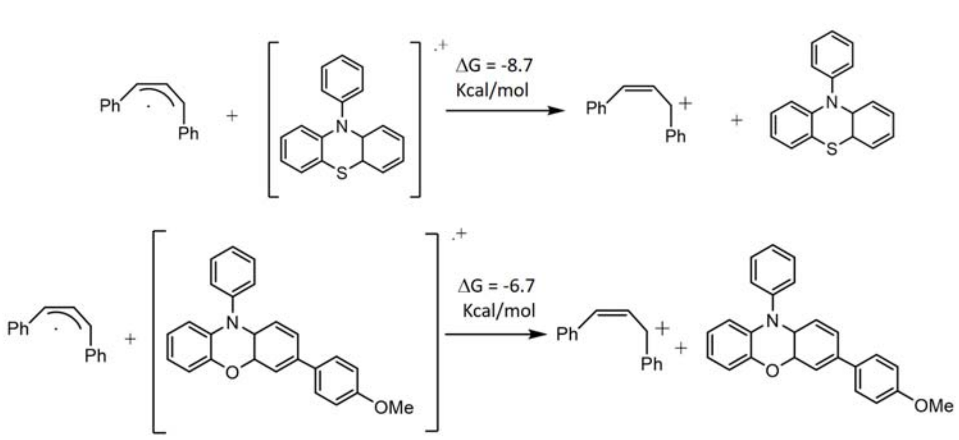

Supplementary Figure 27. Energetic profile for redox reaction between allyl radical and oxidized photocatalysts.

Theoretical evaluation of the isomerization of INT I and INT II:

In order to explain that the intermediate radical **I** or carbocation **II** can maintain its stereochemical information we have carried out additional calculations (Supplementary Figure 28). Although the energetic barriers are not very high (10.2-15.5 kcal/mol), they are significantly higher than the activation energy of the nucleophilic attack to the carbocation (4-5 kcal/mol). In addition, the ultrafast conversion of INT I at the sub-nanosecond scale to the carbocation via back electron transfer discards the isomerization of the radical intermediate. Therefore, the isomerization of these intermediates can be discarded.

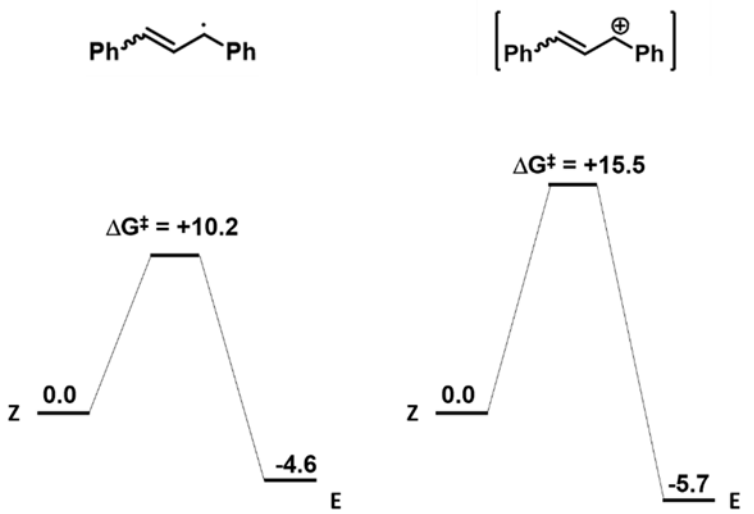

Supplementary Figure 28. Energetic profile for the isomerization of **INT I** and **INT II**.

Theoretical evaluation of reaction between carbocation and pyrrole:

Once the formation of a common carbocation intermediate is achieved, a Friedel-Crafts reaction between the carbocation and pyrrole takes place. Theoretical calculations

allowed to evaluate the thermodynamics and kinetics of such process. The first required step is the approaching of the reactants to generate an initial Van der Waals complex. The energetic difference between the initial complex and reactants calculated separately allows an estimation of the entropic cost of such approaching. In that case value of this cost is around 6 kcal/mol. From this initial complex, the system only has to overcome a barrier of 3.8 kcal/mol to produce the protonated intermediate **III**, that lays 14 kcal/mol below the reactants forming the initial Van der Waals complex. Thus, reaction between carbocation and pyrrole is a very favourable process from both thermodynamic and kinetic points of view.

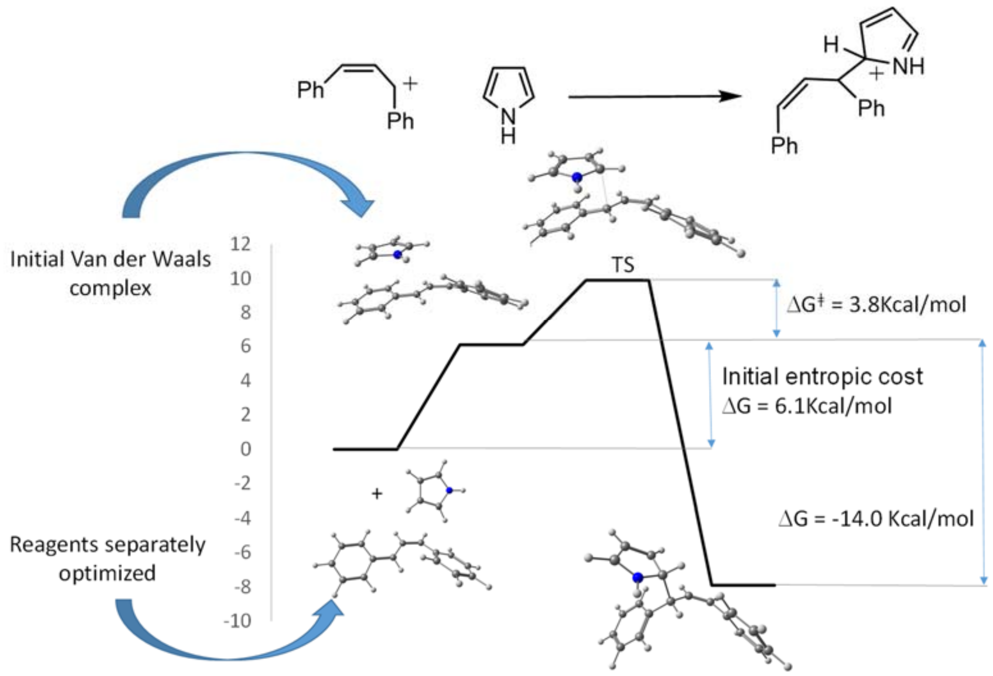

Supplementary Figure 29. Energetic profile for reaction between carbocation and pyrrole.

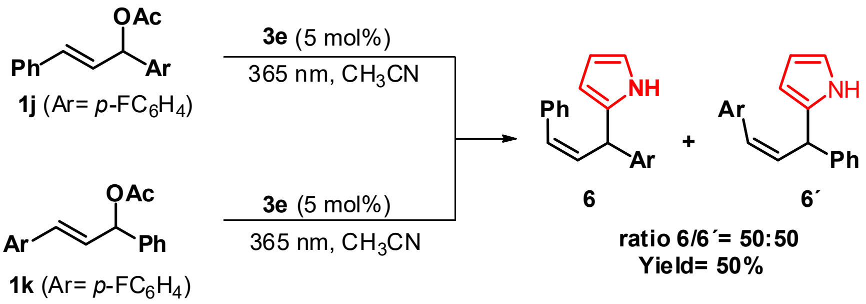

Supplementary Figure 30. The reaction of the two non-symmetric allylic derivatives bearing different aryl groups (**1j, 1k**) afforded the same equimolecular mixture of

products **6** and **6'** revealing that the reaction takes place through a common intermediate.,

Supplementary Tables 1.

Optimized geometries:

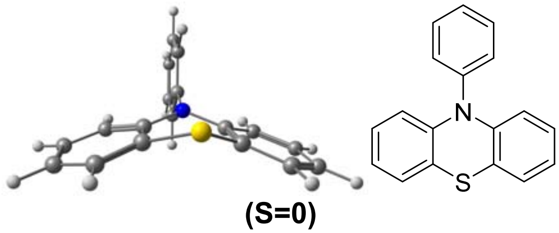

(S=0)

Sum of electronic and thermal Free Energies= **-1146.355886**

6	-0.406151000	3.553305000	-0.739516000
6	-1.777366000	3.643586000	-0.537917000
6	0.270175000	2.358501000	-0.513934000
1	1.336640000	2.315460000	-0.687349000
6	-2.468555000	2.526336000	-0.081423000
6	-0.412730000	1.227771000	-0.052799000
1	-3.537270000	2.575418000	0.095470000
6	-1.791220000	1.341904000	0.184271000
1	0.153061000	4.415022000	-1.084907000
6	-0.412148000	-1.227825000	-0.052569000
6	-1.790395000	-1.342701000	0.184359000
6	0.271408000	-2.358279000	-0.513972000
1	1.337831000	-2.314496000	-0.687492000
6	-2.467317000	-2.527553000	-0.081437000
6	-0.404293000	-3.553217000	-0.739643000
1	-3.536027000	-2.576768000	0.095452000
1	0.155030000	-4.414797000	-1.085191000
6	-1.775620000	-3.644274000	-0.537907000
7	0.246929000	0.000132000	0.191732000
6	1.684924000	0.000438000	0.189541000
6	2.401760000	0.000551000	-1.006201000
6	2.349297000	0.000479000	1.410046000
6	3.791466000	0.000659000	-0.974333000
6	3.741795000	0.000704000	1.437563000
6	4.461774000	0.000743000	0.247435000
1	1.866127000	0.000463000	-1.949840000

1	1.770409000	0.000444000	2.326809000
1	4.351610000	0.000696000	-1.902028000
1	4.261478000	0.000761000	2.388494000
1	5.545332000	0.000865000	0.269580000
16	-2.651517000	-0.000603000	0.962594000
1	-2.305903000	4.569995000	-0.725934000
1	-2.303370000	-4.571104000	-0.726066000

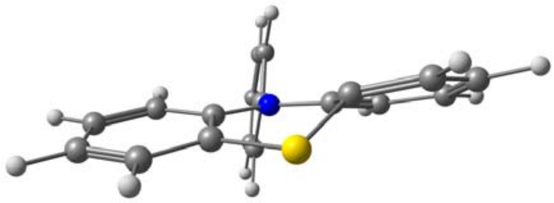

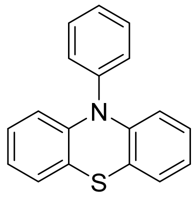

(S=1)

Sum of electronic and thermal Free Energies= **-1146.250717**

6	0.408669000	-3.637988000	0.236115000
6	1.799968000	-3.686616000	-0.160319000
6	-0.251907000	-2.419996000	0.282806000
1	2.283420000	-4.651090000	-0.274766000
1	-1.312741000	-2.399916000	0.501925000
6	2.498084000	-2.553125000	-0.406772000
6	0.417290000	-1.226252000	-0.000081000
1	3.543172000	-2.593909000	-0.698371000
6	1.865711000	-1.252869000	-0.288217000
1	-0.123470000	-4.553458000	0.456234000
6	0.391587000	1.229933000	-0.147086000
6	1.774530000	1.391692000	0.106011000
6	-0.370957000	2.396077000	-0.399427000
1	-1.425058000	2.302918000	-0.623669000
6	2.338889000	2.675444000	0.156709000
6	0.206175000	3.644680000	-0.361666000
1	3.400278000	2.770154000	0.359933000
1	-0.406431000	4.515392000	-0.563060000
6	1.570909000	3.796738000	-0.068554000
1	2.021727000	4.780645000	-0.036926000
7	-0.237741000	-0.009667000	-0.091679000
6	-1.678636000	-0.030060000	-0.023272000
6	-2.407782000	-0.368889000	-1.155965000
6	-2.304167000	0.270155000	1.179904000
6	-3.796417000	-0.402863000	-1.080005000
6	-3.693221000	0.231229000	1.247596000
6	-4.437688000	-0.104448000	0.119669000
1	-1.886664000	-0.600560000	-2.078190000
1	-1.704801000	0.528471000	2.046038000
1	-4.375983000	-0.662143000	-1.958170000
1	-4.192747000	0.459719000	2.181605000
1	-5.519566000	-0.133908000	0.175658000
16	2.836052000	0.036769000	0.358053000

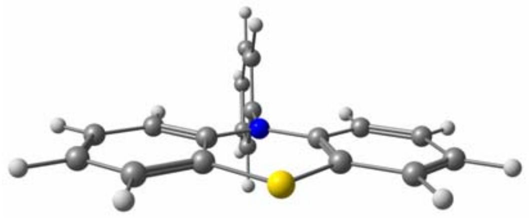

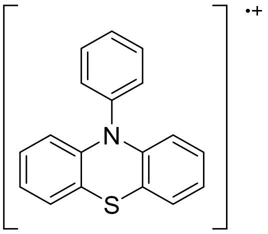

Sum of electronic and thermal Free Energies= **-1146.169476**

6	0.258551000	-3.649322000	0.000098000
6	1.658000000	-3.757789000	-0.000028000
6	-0.355204000	-2.419743000	0.000158000
1	-1.433974000	-2.362286000	0.000272000
6	2.428270000	-2.619059000	-0.000053000
6	0.412165000	-1.232585000	0.000067000
1	3.510275000	-2.686038000	-0.000117000
6	1.820443000	-1.354186000	-0.000010000
1	-0.352757000	-4.543183000	0.000154000
6	0.412285000	1.232568000	0.000059000
6	1.820576000	1.354026000	0.000004000
6	-0.354959000	2.419810000	0.000122000
1	-1.433734000	2.362470000	0.000206000
6	2.428534000	2.618838000	-0.000022000
6	0.258925000	3.649325000	0.000071000
1	3.510545000	2.685705000	-0.000062000
1	-0.352290000	4.543250000	0.000106000
6	1.658383000	3.757648000	-0.000015000
7	-0.222947000	0.000020000	0.000079000
6	-1.674180000	0.000073000	0.000021000
6	-2.343206000	0.000083000	-1.215243000
6	-2.343337000	0.000103000	1.215208000
6	-3.734113000	0.000139000	-1.208171000
6	-3.734242000	0.000157000	1.207985000
6	-4.426195000	0.000177000	-0.000130000
1	-1.780505000	0.000056000	-2.141917000
1	-1.780704000	0.000087000	2.141925000
1	-4.275074000	0.000153000	-2.146627000
1	-4.275304000	0.000183000	2.146383000
1	-5.509706000	0.000219000	-0.000187000
16	2.895356000	-0.000133000	-0.000079000
1	2.131749000	-4.731134000	-0.000088000
1	2.132232000	4.730943000	-0.000060000

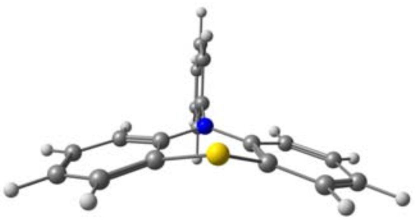

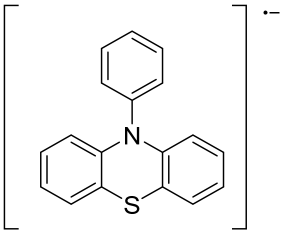

Sum of electronic and thermal Free Energies= -**1146.397071**

6	0.347758000	-3.620195000	-0.597422000
6	1.736690000	-3.721563000	-0.417181000
6	-0.295663000	-2.396297000	-0.449329000
1	-1.365412000	-2.333222000	-0.604008000
6	2.451701000	-2.589480000	-0.062559000
6	0.415501000	-1.235944000	-0.106103000
1	3.523055000	-2.647618000	0.102260000
6	1.810646000	-1.357820000	0.111074000
1	-0.235283000	-4.493919000	-0.864502000
6	0.415518000	1.235942000	-0.106094000
6	1.810665000	1.357797000	0.111082000
6	-0.295626000	2.396307000	-0.449324000
1	-1.365376000	2.333249000	-0.604008000
6	2.451740000	2.589448000	-0.062550000
6	0.347814000	3.620195000	-0.597418000
1	3.523095000	2.647570000	0.102270000
1	-0.235213000	4.493927000	-0.864503000
6	1.736747000	3.721542000	-0.417172000
7	-0.229254000	0.000003000	0.060003000
6	-1.660614000	0.000009000	0.133020000
6	-2.418316000	0.000027000	-1.062647000
6	-2.278740000	0.000001000	1.371849000
6	-3.826354000	0.000035000	-0.957721000
6	-3.675915000	0.000008000	1.469531000
6	-4.446456000	0.000026000	0.276956000
1	-1.914949000	0.000033000	-2.022034000
1	-1.657274000	-0.000013000	2.262796000
1	-4.429035000	0.000050000	-1.860768000
1	-4.157955000	0.000001000	2.439254000
1	-5.529812000	0.000033000	0.335269000
16	2.699648000	-0.000020000	0.820834000
1	2.245954000	-4.668378000	-0.551677000
1	2.246026000	4.668348000	-0.551669000

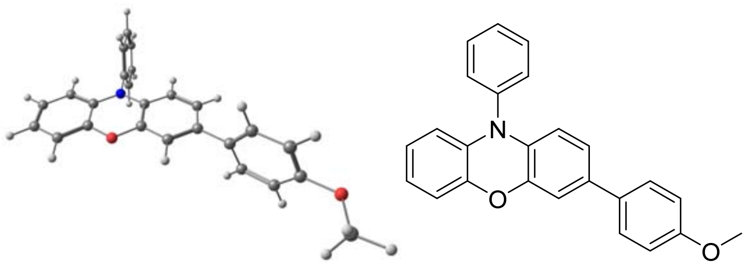

(S=0)

Sum of electronic and thermal Free Energies= **-1168.800542**

6	-3.519574000	2.893287000	0.000000000
6	-2.272797000	2.298907000	0.000000000
6	-1.100823000	3.071383000	0.000000000
6	0.189570000	1.026896000	0.000000000
6	-1.010502000	0.300094000	0.000000000
6	-3.634391000	4.283831000	0.000000000
6	-1.226527000	4.459695000	0.000000000
6	1.386268000	0.313332000	0.000000000
6	-1.010961000	-1.081154000	0.000000000
6	0.192891000	-1.794204000	0.000000000
6	1.385993000	-1.081102000	0.000000000
1	2.325865000	0.851296000	0.000000000
1	-1.965602000	-1.595613000	0.000000000
1	2.331400000	-1.611494000	0.000000000
6	-2.485593000	5.060138000	0.000000000
1	-4.393172000	2.251674000	0.000000000
1	-4.615047000	4.743001000	0.000000000
1	-0.333782000	5.072763000	0.000000000
1	-2.553964000	6.141353000	0.000000000
6	0.185234000	-3.286419000	0.000000000
6	0.185587000	-3.996206000	1.203129000
6	0.185587000	-3.996206000	-1.203129000
6	0.185587000	-5.386983000	1.207129000
6	0.185587000	-5.386983000	-1.207129000
6	0.191636000	-6.079374000	0.000000000
1	0.188686000	-3.454101000	2.142637000
1	0.188686000	-3.454101000	-2.142637000
1	0.193249000	-5.944583000	2.137184000
1	0.193249000	-5.944583000	-2.137184000
8	0.229396000	-7.451426000	0.000000000
6	-1.066562000	-8.047749000	0.000000000
1	-1.628734000	-7.756672000	0.893084000
1	-1.628734000	-7.756672000	-0.893084000
1	-0.917732000	-9.126674000	0.000000000
8	-2.237103000	0.922166000	0.000000000
7	0.140653000	2.425508000	0.000000000
6	1.353299000	3.184305000	0.000000000
6	1.939872000	3.543666000	1.209583000
6	1.939872000	3.543666000	-1.209583000
6	3.127932000	4.268055000	1.206855000
6	3.127932000	4.268055000	-1.206855000
6	3.721501000	4.628578000	0.000000000
1	1.463257000	3.251416000	2.138995000

1	1.463257000	3.251416000	-2.138995000
1	3.589855000	4.549537000	2.145943000
1	3.589855000	4.549537000	-2.145943000
1	4.646774000	5.192970000	0.000000000

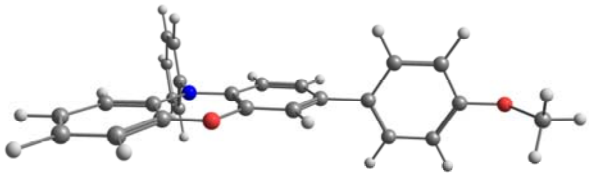 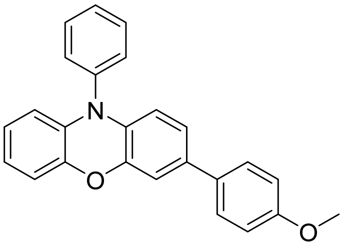

(S=1)

Sum of electronic and thermal Free Energies= **-1168.694354**

6	-3.452135000	3.016561000	0.000000000
6	-2.218557000	2.364411000	0.000000000
6	-1.005844000	3.099528000	0.000000000
6	0.232354000	1.042091000	0.000000000
6	-1.060855000	0.328366000	0.000000000
6	-3.503072000	4.394476000	0.000000000
6	-1.082005000	4.509625000	0.000000000
6	1.408886000	0.273785000	0.000000000
6	-1.100472000	-1.071288000	0.000000000
6	0.059032000	-1.802824000	0.000000000
6	1.358603000	-1.098838000	0.000000000
1	2.366595000	0.781555000	0.000000000
1	-2.071409000	-1.556534000	0.000000000
1	2.270673000	-1.681421000	0.000000000
6	-2.304576000	5.140989000	0.000000000
1	-4.350204000	2.409603000	0.000000000
1	-4.460336000	4.900195000	0.000000000
1	-0.167875000	5.089842000	0.000000000
1	-2.342137000	6.223326000	0.000000000
6	0.050512000	-3.289670000	0.000000000
6	0.061542000	-4.003272000	1.202570000
6	0.061542000	-4.003272000	-1.202570000
6	0.061542000	-5.394509000	1.206988000
6	0.061542000	-5.394509000	-1.206988000
6	0.066754000	-6.086907000	0.000000000
1	0.065133000	-3.461595000	2.142490000
1	0.065133000	-3.461595000	-2.142490000
1	0.067300000	-5.952119000	2.137170000
1	0.067300000	-5.952119000	-2.137170000
8	0.102606000	-7.459703000	0.000000000
6	-1.193866000	-8.053886000	0.000000000
1	-1.755898000	-7.762086000	0.893013000
1	-1.755898000	-7.762086000	-0.893013000
1	-1.047053000	-9.133158000	0.000000000
8	-2.225146000	1.015850000	0.000000000
7	0.192540000	2.413072000	0.000000000
6	1.432736000	3.144892000	0.000000000
6	2.018140000	3.479631000	1.213439000
6	2.018140000	3.479631000	-1.213439000
6	3.224518000	4.173373000	1.207875000
6	3.224518000	4.173373000	-1.207875000
6	3.825513000	4.517750000	0.000000000
1	1.530910000	3.198399000	2.140370000

1	1.530910000	3.198399000	-2.140370000
1	3.693884000	4.442625000	2.146522000
1	3.693884000	4.442625000	-2.146522000
1	4.764681000	5.058421000	0.000000000

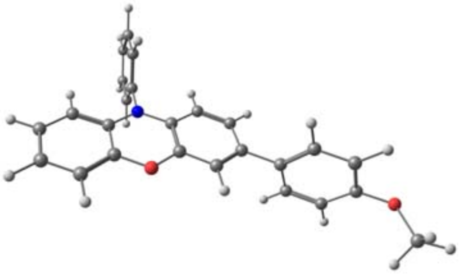

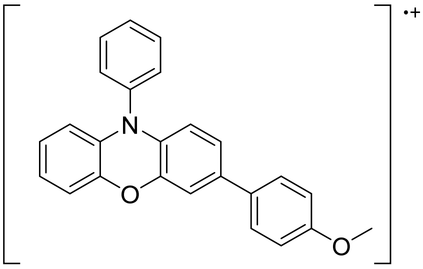

Sum of electronic and thermal Free Energies= **-1168.617325**

6	-3.481679000	2.943577000	0.000000000
6	-2.237099000	2.324008000	0.000000000
6	-1.044092000	3.076110000	0.000000000
6	0.190505000	1.025639000	0.000000000
6	-1.030573000	0.321290000	0.000000000
6	-3.540778000	4.321945000	0.000000000
6	-1.125881000	4.480781000	0.000000000
6	1.391708000	0.292783000	0.000000000
6	-1.065976000	-1.068154000	0.000000000
6	0.121509000	-1.778767000	0.000000000
6	1.348692000	-1.081224000	0.000000000
1	2.338093000	0.816644000	0.000000000
1	-2.028822000	-1.564942000	0.000000000
1	2.274444000	-1.643862000	0.000000000
6	-2.361250000	5.086858000	0.000000000
1	-4.370806000	2.325604000	0.000000000
1	-4.503803000	4.816658000	0.000000000
1	-0.218138000	5.069387000	0.000000000
1	-2.422385000	6.167649000	0.000000000
6	0.109414000	-3.268780000	0.000000000
6	0.111352000	-3.971438000	1.205979000
6	0.111352000	-3.971438000	-1.205979000
6	0.111352000	-5.361630000	1.207873000
6	0.111352000	-5.361630000	-1.207873000
6	0.117672000	-6.053152000	0.000000000
1	0.114433000	-3.427967000	2.144400000
1	0.114433000	-3.427967000	-2.144400000
1	0.118569000	-5.919392000	2.137613000
1	0.118569000	-5.919392000	-2.137613000
8	0.156921000	-7.423592000	0.000000000
6	-1.138551000	-8.022624000	0.000000000
1	-1.700638000	-7.732380000	0.893262000
1	-1.700638000	-7.732380000	-0.893262000
1	-0.986956000	-9.101018000	0.000000000
8	-2.213792000	0.972311000	0.000000000
7	0.160633000	2.403745000	0.000000000
6	1.402761000	3.146228000	0.000000000
6	1.978934000	3.483018000	1.216741000
6	1.978934000	3.483018000	-1.216741000
6	3.180225000	4.183780000	1.208359000
6	3.180225000	4.183780000	-1.208359000
6	3.777935000	4.531150000	0.000000000
1	1.493269000	3.198647000	2.143257000
1	1.493269000	3.198647000	-2.143257000
1	3.647854000	4.455968000	2.146761000

1	3.647854000	4.455968000	-2.146761000
1	4.713665000	5.077376000	0.000000000

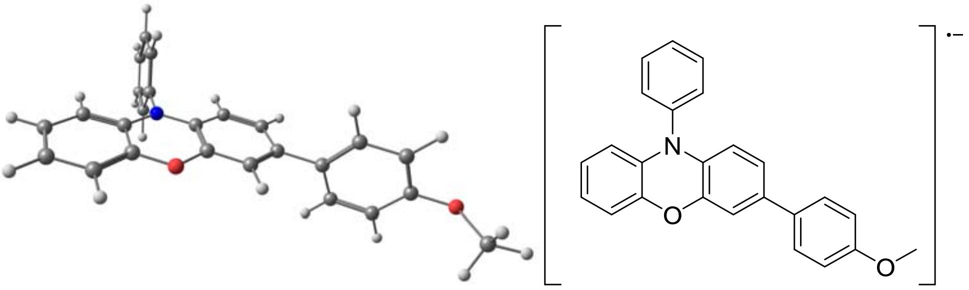

Sum of electronic and thermal Free Energies= **-1168.841207**

6	-3.544923000	2.913724000	0.000000000
6	-2.296987000	2.304997000	0.000000000
6	-1.114005000	3.085088000	0.000000000
6	0.183677000	1.041959000	0.000000000
6	-1.026594000	0.309061000	0.000000000
6	-3.660389000	4.300547000	0.000000000
6	-1.245484000	4.476111000	0.000000000
6	1.384319000	0.340432000	0.000000000
6	-1.013853000	-1.091391000	0.000000000
6	0.187132000	-1.786484000	0.000000000
6	1.400422000	-1.065671000	0.000000000
1	2.315855000	0.893117000	0.000000000
1	-1.963610000	-1.616394000	0.000000000
1	2.345979000	-1.594334000	0.000000000
6	-2.500897000	5.082877000	0.000000000
1	-4.420973000	2.273978000	0.000000000
1	-4.640965000	4.760667000	0.000000000
1	-0.349062000	5.085215000	0.000000000
1	-2.566552000	6.164353000	0.000000000
6	0.199782000	-3.277183000	0.000000000
6	0.208832000	-3.990602000	1.201957000
6	0.208832000	-3.990602000	-1.201957000
6	0.208832000	-5.383160000	1.207186000
6	0.208832000	-5.383160000	-1.207186000
6	0.213360000	-6.075243000	0.000000000
1	0.210987000	-3.448128000	2.141473000
1	0.210987000	-3.448128000	-2.141473000
1	0.214589000	-5.941356000	2.137071000
1	0.214589000	-5.941356000	-2.137071000
8	0.246283000	-7.449278000	0.000000000
6	-1.051339000	-8.039678000	0.000000000
1	-1.613031000	-7.746461000	0.892880000
1	-1.613031000	-7.746461000	-0.892880000
1	-0.908197000	-9.119560000	0.000000000
8	-2.255139000	0.938999000	0.000000000
7	0.120655000	2.438336000	0.000000000
6	1.335695000	3.185934000	0.000000000
6	1.920872000	3.516551000	1.216080000
6	1.920872000	3.516551000	-1.216080000
6	3.148241000	4.219206000	1.214701000
6	3.148241000	4.219206000	-1.214701000
6	3.742864000	4.555350000	0.000000000
1	1.427041000	3.232556000	2.138511000

1	1.427041000	3.232556000	-2.138511000
1	3.624992000	4.487629000	2.149910000
1	3.624992000	4.487629000	-2.149910000
1	4.686624000	5.091836000	0.000000000

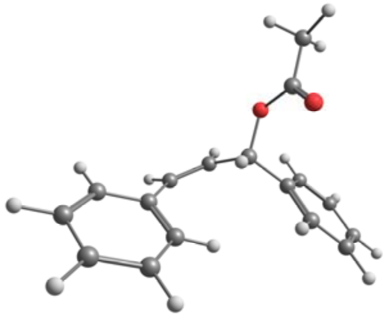

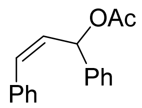

(S=0)

Sum of electronic and thermal Free Energies= **-807.536344**

6	-0.233430000	0.450798000	1.651093000
6	-1.531037000	0.193139000	1.826378000
1	0.372854000	0.669964000	2.526675000
1	-1.916381000	0.250424000	2.841732000
6	-2.538547000	-0.121225000	0.792040000
6	-2.286443000	-1.008599000	-0.259792000
6	-3.805929000	0.468200000	0.883555000
6	-3.263602000	-1.269139000	-1.216010000
6	-4.778758000	0.213659000	-0.074827000
6	-4.508250000	-0.652725000	-1.131957000
1	-1.332893000	-1.521616000	-0.311954000
1	-4.020328000	1.138462000	1.709654000
1	-3.053624000	-1.962215000	-2.022741000
1	-5.750709000	0.687089000	0.005643000
1	-5.268139000	-0.854275000	-1.878205000
6	0.509522000	0.534154000	0.348439000
1	-0.169112000	0.535836000	-0.504832000
6	1.529144000	-0.576701000	0.188938000
6	1.439423000	-1.476828000	-0.870397000
6	2.575020000	-0.699887000	1.106306000
6	2.371554000	-2.502681000	-1.003316000
6	3.510264000	-1.719837000	0.970240000
6	3.407491000	-2.626437000	-0.082651000
1	0.642011000	-1.366927000	-1.598014000
1	2.661456000	0.007868000	1.924393000
1	2.293338000	-3.198449000	-1.830875000
1	4.319385000	-1.808539000	1.686214000
1	4.136199000	-3.422024000	-0.187735000
8	1.188573000	1.812465000	0.397522000
6	1.628434000	2.303542000	-0.775168000
8	1.443866000	1.755019000	-1.829916000
6	2.368654000	3.591959000	-0.577116000
1	3.308071000	3.381793000	-0.060576000
1	1.783542000	4.266821000	0.048294000
1	2.573744000	4.047561000	-1.543035000

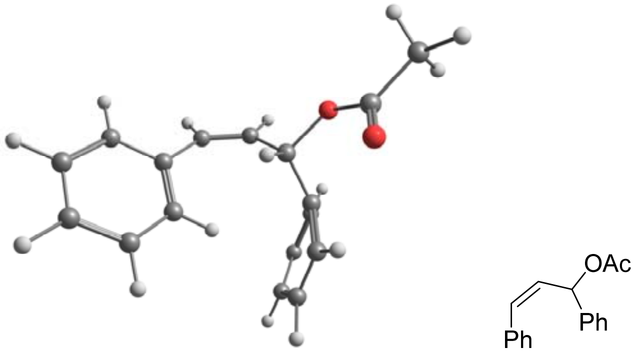

(S=1)

Sum of electronic and thermal Free Energies= **-807.402483**

6	0.203269000	0.731617000	1.638662000
6	-1.118789000	0.844264000	1.793602000
1	0.838938000	0.837863000	2.513491000
1	-1.489804000	1.080040000	2.788077000
6	-2.148389000	0.703381000	0.746943000
6	-2.054401000	-0.280400000	-0.243136000
6	-3.267571000	1.546257000	0.751581000
6	-3.032124000	-0.396474000	-1.226010000
6	-4.241996000	1.433625000	-0.232744000
6	-4.124500000	0.465067000	-1.228490000
1	-1.230758000	-0.983677000	-0.221803000
1	-3.364784000	2.297513000	1.528847000
1	-2.941817000	-1.167707000	-1.982969000
1	-5.097855000	2.098957000	-0.221830000
1	-4.888238000	0.374527000	-1.992397000
6	0.947589000	0.521436000	0.349017000
1	0.366414000	0.866097000	-0.509566000
6	1.418924000	-0.890067000	0.112712000
6	1.179822000	-1.530923000	-1.206287000
6	1.600277000	-1.809686000	1.268288000
6	0.575914000	-2.736102000	-1.225571000
6	0.973084000	-3.004212000	1.226544000
6	0.327768000	-3.443734000	0.012659000
1	1.448766000	-0.996565000	-2.110477000
1	2.182837000	-1.498560000	2.129597000
1	0.318529000	-3.216285000	-2.164086000
1	1.007513000	-3.680325000	2.074734000
1	-0.194361000	-4.391013000	-0.006032000
8	2.115117000	1.394991000	0.479549000
6	2.773920000	1.695541000	-0.649504000
8	2.439393000	1.301016000	-1.737564000
6	3.962006000	2.566964000	-0.369679000
1	4.700575000	1.986952000	0.188285000
1	3.665932000	3.416534000	0.246549000
1	4.393227000	2.908259000	-1.307842000

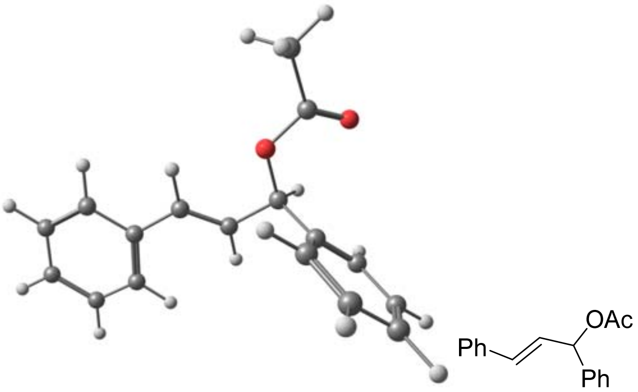

(S=0)

Sum of electronic and thermal Free Energies= **-807.541654**

6	-0.436072000	-0.064981000	-0.727533000
6	-1.428413000	0.550014000	-0.084540000
1	-0.604650000	-0.984426000	-1.282334000
1	-1.218198000	1.480050000	0.435654000
6	-2.830458000	0.095183000	-0.028572000
6	-3.233301000	-1.187187000	-0.425888000
6	-3.804144000	0.980195000	0.448646000
6	-4.568889000	-1.561148000	-0.360641000
6	-5.142523000	0.606287000	0.513214000
6	-5.530592000	-0.666100000	0.106403000
1	-2.498075000	-1.902281000	-0.777897000
1	-3.502769000	1.972401000	0.768814000
1	-4.862016000	-2.558101000	-0.669763000
1	-5.880399000	1.308562000	0.884431000
1	-6.571790000	-0.963031000	0.157780000
6	0.989414000	0.406271000	-0.767314000
1	1.273737000	0.628482000	-1.799905000
6	1.921009000	-0.664328000	-0.226372000
6	2.671892000	-1.448420000	-1.097440000
6	1.992631000	-0.895000000	1.147547000
6	3.488770000	-2.461051000	-0.600743000
6	2.816671000	-1.898774000	1.644392000
6	3.563729000	-2.686113000	0.770319000
1	2.621970000	-1.259904000	-2.165074000
1	1.402643000	-0.285313000	1.824447000
1	4.073395000	-3.067037000	-1.283568000
1	2.872871000	-2.071207000	2.713172000
1	4.204738000	-3.469475000	1.158054000
8	1.091109000	1.605280000	0.009714000
6	2.188447000	2.363642000	-0.189880000
8	3.032701000	2.092804000	-1.000983000
6	2.195843000	3.548262000	0.727008000
1	2.274193000	3.197963000	1.758523000
1	1.259165000	4.098691000	0.628798000
1	3.040610000	4.189447000	0.487384000

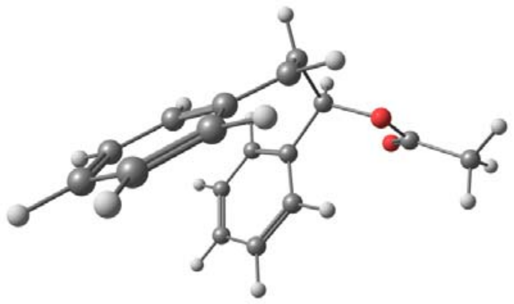

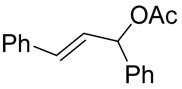

(S=1)

Sum of electronic and thermal Free Energies= **-807.456773**

6	0.380189000	1.173821000	-1.635028000
6	-0.596091000	1.824662000	-0.764743000
1	0.373433000	1.344346000	-2.706650000
1	-0.351054000	2.808406000	-0.367236000
6	-1.848569000	1.268778000	-0.385743000
6	-2.292311000	0.004240000	-0.849435000
6	-2.694781000	1.980032000	0.501554000
6	-3.506434000	-0.515199000	-0.433621000
6	-3.906239000	1.451441000	0.909241000
6	-4.321054000	0.199024000	0.448604000
1	-1.667017000	-0.559884000	-1.533328000
1	-2.371780000	2.950526000	0.864072000
1	-3.825653000	-1.486212000	-0.795985000
1	-4.535225000	2.012625000	1.591248000
1	-5.268861000	-0.214470000	0.772684000
6	1.500752000	0.361603000	-1.053103000
1	2.193009000	0.052581000	-1.837141000
6	0.956347000	-0.863345000	-0.343903000
6	0.778323000	-2.044530000	-1.062381000
6	0.530198000	-0.793678000	0.982147000
6	0.167471000	-3.143384000	-0.466712000
6	-0.079732000	-1.893750000	1.578161000
6	-0.268435000	-3.067853000	0.854335000
1	1.113924000	-2.098334000	-2.093645000
1	0.665769000	0.124279000	1.544148000
1	0.033655000	-4.059024000	-1.031471000
1	-0.413869000	-1.831479000	2.607660000
1	-0.746795000	-3.922429000	1.318877000
8	2.200289000	1.218507000	-0.121636000
6	3.419232000	0.804028000	0.273181000
8	3.948005000	-0.185849000	-0.158831000
6	3.991343000	1.720752000	1.311904000
1	3.457516000	1.554660000	2.251221000
1	3.847888000	2.761306000	1.021006000
1	5.047455000	1.501980000	1.451527000

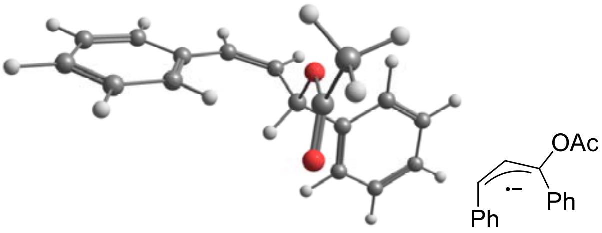

Sum of electronic and thermal Free Energies= **-807.607337**

6	0.026399000	-1.284151000	-0.914474000
6	1.388305000	-1.592444000	-0.941024000
1	-0.632740000	-1.880225000	-1.537718000
1	1.637095000	-2.476109000	-1.529379000
6	2.522890000	-0.976614000	-0.349782000
6	2.534824000	0.265143000	0.379431000
6	3.808194000	-1.608663000	-0.485366000
6	3.701843000	0.779442000	0.919002000
6	4.957730000	-1.080651000	0.057093000
6	4.934843000	0.128920000	0.782628000
1	1.623121000	0.832980000	0.506357000
1	3.860914000	-2.543434000	-1.037839000
1	3.652330000	1.721412000	1.458847000
1	5.897438000	-1.608401000	-0.078855000
1	5.838712000	0.542170000	1.212888000
6	-0.624869000	-0.221767000	-0.118266000
1	-0.157140000	-0.090800000	0.858130000
6	-2.097248000	-0.483017000	0.081934000
6	-2.574406000	-0.948785000	1.307329000
6	-2.995909000	-0.302637000	-0.972680000
6	-3.924189000	-1.242679000	1.475439000
6	-4.346482000	-0.595666000	-0.805720000
6	-4.814651000	-1.067994000	0.418116000
1	-1.880346000	-1.079966000	2.131549000
1	-2.631484000	0.074366000	-1.922576000
1	-4.282809000	-1.602822000	2.433363000
1	-5.035877000	-0.452755000	-1.630497000
1	-5.866927000	-1.293208000	0.549078000
8	-0.477655000	1.096692000	-0.811655000
6	-0.579309000	2.193512000	-0.061955000
8	-0.776948000	2.183285000	1.130434000
6	-0.402123000	3.439794000	-0.884773000
1	-1.201821000	3.500928000	-1.625895000
1	0.547676000	3.390501000	-1.419985000
1	-0.426367000	4.315093000	-0.239273000

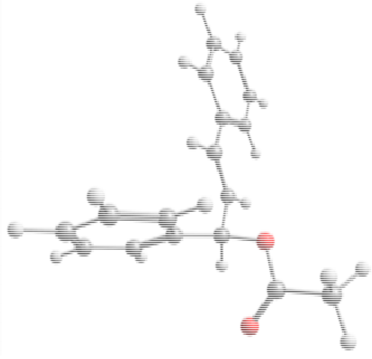

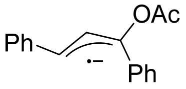

Sum of electronic and thermal Free Energies= **-807.609451**

6	0.403578000	-0.714658000	-0.850015000
6	1.245352000	0.026165000	-0.021369000
1	0.812443000	-1.454057000	-1.531208000
1	0.779963000	0.742708000	0.655329000
6	2.660470000	-0.024326000	0.033991000
6	3.456137000	-0.909601000	-0.775334000
6	3.397198000	0.826726000	0.922947000
6	4.836692000	-0.925520000	-0.683732000
6	4.772212000	0.794957000	1.000771000
6	5.530143000	-0.084288000	0.197128000
1	2.967568000	-1.583158000	-1.471095000
1	2.840551000	1.515533000	1.553868000
1	5.395866000	-1.612139000	-1.314111000
1	5.278373000	1.460891000	1.693951000
1	6.611212000	-0.106756000	0.258821000
6	-1.076566000	-0.687395000	-0.758730000
1	-1.524160000	-0.992214000	-1.706836000
6	-1.656188000	0.657822000	-0.369350000
6	-1.799871000	1.639614000	-1.352319000
6	-2.003422000	0.965099000	0.945349000
6	-2.281692000	2.902540000	-1.029504000
6	-2.482081000	2.232917000	1.272480000
6	-2.623749000	3.204971000	0.287995000
1	-1.527129000	1.406171000	-2.377363000
1	-1.895529000	0.211195000	1.717236000
1	-2.393975000	3.652013000	-1.805205000
1	-2.744115000	2.458927000	2.300373000
1	-2.998379000	4.189887000	0.542224000
8	-1.533475000	-1.700674000	0.232174000
6	-2.790197000	-2.134250000	0.126998000
8	-3.562552000	-1.771580000	-0.727707000
6	-3.125322000	-3.116869000	1.215122000
1	-3.216190000	-2.574646000	2.159837000
1	-2.325858000	-3.849861000	1.324651000
1	-4.068580000	-3.609886000	0.989213000

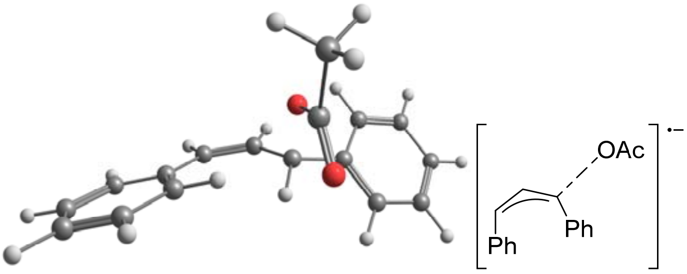

TS

Sum of electronic and thermal Free Energies= **-807.605148**

6	0.056543000	-1.461874000	-0.755856000
6	1.430825000	-1.728756000	-0.783056000
1	-0.569835000	-2.125097000	-1.345645000
1	1.706868000	-2.649526000	-1.294425000
6	2.545779000	-1.018331000	-0.246448000
6	2.503774000	0.283587000	0.342200000
6	3.839641000	-1.623722000	-0.306823000
6	3.648457000	0.890280000	0.838878000
6	4.969338000	-1.006750000	0.190828000
6	4.895790000	0.264684000	0.782014000
1	1.572552000	0.830326000	0.394145000
1	3.925355000	-2.608985000	-0.756945000
1	3.564358000	1.881232000	1.275693000
1	5.925900000	-1.515791000	0.121671000
1	5.781663000	0.748005000	1.175635000
6	-0.621697000	-0.411035000	-0.056557000
1	-0.142259000	-0.053821000	0.848591000
6	-2.088972000	-0.576466000	0.138773000
6	-2.658201000	-0.386053000	1.401881000
6	-2.931085000	-0.897208000	-0.934495000
6	-4.026745000	-0.547969000	1.597601000
6	-4.297349000	-1.056643000	-0.739857000
6	-4.852220000	-0.886702000	0.528706000
1	-2.018884000	-0.109401000	2.233552000
1	-2.508399000	-1.009573000	-1.927242000
1	-4.449921000	-0.404118000	2.585848000
1	-4.934805000	-1.307184000	-1.580771000
1	-5.918726000	-1.009211000	0.679610000
8	-0.531459000	1.028354000	-0.984321000
6	-0.536550000	2.151337000	-0.322077000
8	-0.548104000	2.253283000	0.894944000
6	-0.509175000	3.365560000	-1.228526000
1	-1.381869000	3.350688000	-1.884413000
1	0.381355000	3.327808000	-1.859092000
1	-0.505770000	4.277903000	-0.634284000

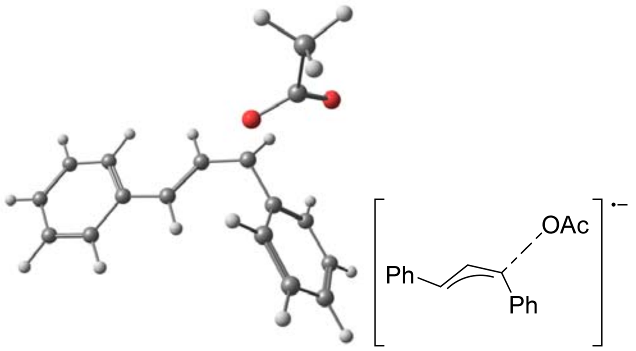

TS

Sum of electronic and thermal Free Energies= **-807.605140**

6	0.468399000	-0.644301000	-0.785911000
6	1.342312000	0.158660000	-0.044507000
1	0.883612000	-1.442965000	-1.393824000
1	0.922732000	0.969246000	0.546057000
6	2.763304000	0.053559000	-0.009691000
6	3.504279000	-0.948912000	-0.705137000
6	3.533073000	0.980592000	0.753038000
6	4.887013000	-1.002638000	-0.637054000
6	4.911849000	0.913861000	0.813592000
6	5.617092000	-0.079224000	0.117751000
1	2.980198000	-1.690306000	-1.297488000
1	3.009356000	1.760978000	1.298367000
1	5.410819000	-1.782890000	-1.181433000
1	5.453513000	1.643277000	1.407895000
1	6.698185000	-0.130105000	0.164644000
6	-0.955428000	-0.577643000	-0.777378000
1	-1.432264000	-1.039521000	-1.635902000
6	-1.642652000	0.688064000	-0.380389000
6	-2.295457000	1.444762000	-1.356376000
6	-1.644683000	1.151099000	0.939222000
6	-2.916125000	2.646829000	-1.030780000
6	-2.263352000	2.353662000	1.266685000
6	-2.898705000	3.108383000	0.283047000
1	-2.314760000	1.084096000	-2.379979000
1	-1.172842000	0.551081000	1.708553000
1	-3.418946000	3.219503000	-1.802262000
1	-2.255670000	2.699568000	2.294548000
1	-3.384359000	4.042847000	0.540086000
8	-1.565314000	-1.707581000	0.436041000
6	-2.745656000	-2.198022000	0.204306000
8	-3.449222000	-1.935416000	-0.760641000
6	-3.213203000	-3.152818000	1.288644000
1	-3.485747000	-2.570392000	2.172727000
1	-2.407739000	-3.831138000	1.571582000
1	-4.082168000	-3.714779000	0.948847000

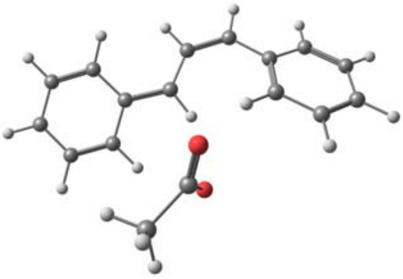

AcO$^-$

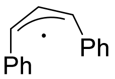

Sum of electronic and thermal Free Energies= **-807.642302**

6	0.221770000	-2.041479000	-0.299765000
6	1.604101000	-2.159449000	-0.340101000
1	-0.311310000	-2.953159000	-0.557214000
1	1.987408000	-3.158618000	-0.528228000
6	2.632890000	-1.154866000	-0.123418000
6	2.426759000	0.230664000	-0.266407000
6	3.925488000	-1.601869000	0.218007000
6	3.471051000	1.119400000	-0.035924000
6	4.959971000	-0.707545000	0.448455000
6	4.735830000	0.663873000	0.328688000
1	1.472075000	0.638824000	-0.587093000
1	4.104268000	-2.668672000	0.310078000
1	3.290688000	2.183109000	-0.152456000
1	5.942486000	-1.077332000	0.719598000
1	5.541542000	1.367248000	0.505688000
6	-0.545483000	-0.949698000	0.094855000
1	-0.077034000	-0.038944000	0.457895000
6	-1.996338000	-0.939948000	0.125846000
6	-2.645285000	0.138799000	0.762088000
6	-2.800302000	-1.949900000	-0.441264000
6	-4.030323000	0.195678000	0.839472000
6	-4.183520000	-1.886449000	-0.360034000
6	-4.808904000	-0.816827000	0.281941000
1	-2.033257000	0.930313000	1.185184000
1	-2.342716000	-2.784170000	-0.960042000
1	-4.506307000	1.034398000	1.335974000
1	-4.781574000	-2.673615000	-0.805761000
1	-5.890318000	-0.772451000	0.340637000
8	-0.037191000	2.037455000	-1.331453000
6	-0.493941000	2.502069000	-0.264032000
8	-0.130056000	2.215594000	0.902093000
6	-1.665837000	3.491707000	-0.393502000
1	-2.596798000	2.915216000	-0.425046000
1	-1.597038000	4.065415000	-1.319045000
1	-1.711705000	4.163077000	0.465276000

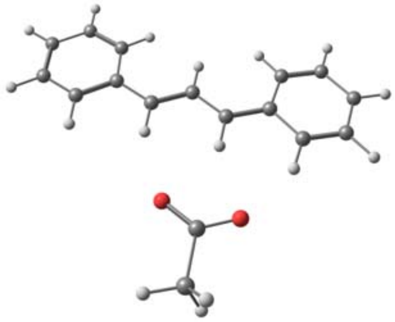

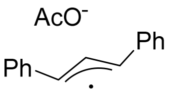

Sum of electronic and thermal Free Energies= **-807.646427**

6	-0.369757000	-0.820973000	-0.000101000
6	-1.450047000	0.051356000	-0.000146000
1	-0.550032000	-1.892599000	0.000034000
1	-1.217018000	1.115722000	-0.000181000
6	-2.848361000	-0.319163000	-0.000091000
6	-3.822013000	0.699736000	0.000205000
6	-3.298695000	-1.655677000	-0.000312000
6	-5.177164000	0.401001000	0.000331000
6	-4.653810000	-1.949226000	-0.000187000
6	-5.602802000	-0.926127000	0.000146000
1	-3.493887000	1.734401000	0.000342000
1	-2.583598000	-2.469968000	-0.000621000
1	-5.904495000	1.205191000	0.000572000
1	-4.976450000	-2.984572000	-0.000358000
1	-6.660667000	-1.161837000	0.000251000
6	0.934114000	-0.347887000	-0.000159000
1	1.082650000	0.732304000	-0.000151000
6	2.140405000	-1.147547000	-0.000077000
6	3.377765000	-0.470517000	-0.000081000
6	2.153868000	-2.557136000	0.000031000
6	4.574259000	-1.174221000	0.000043000
6	3.354020000	-3.252352000	0.000184000
6	4.570730000	-2.568496000	0.000183000
1	3.360318000	0.616556000	-0.000155000
1	1.222342000	-3.111670000	-0.000023000
1	5.515429000	-0.635341000	-0.000011000
1	3.344598000	-4.336709000	0.000304000
1	5.504406000	-3.118995000	0.000320000
8	0.128066000	2.987783000	0.000055000
6	1.318115000	3.372550000	-0.000039000
8	2.346825000	2.656673000	0.000012000
6	1.567314000	4.892587000	-0.000059000
1	2.157216000	5.163623000	0.879531000
1	2.157877000	5.163675000	-0.879191000
1	0.634542000	5.457128000	-0.000421000

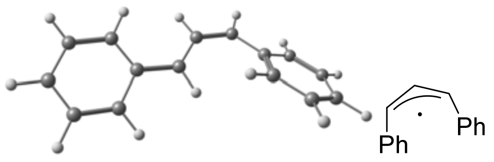

Sum of electronic and thermal Free Energies= **-579.092619**

6	0.059556000	-1.375429000	0.045390000
6	-1.309123000	-1.591103000	0.030517000
1	0.665731000	-2.273859000	0.125577000
1	-1.634805000	-2.625769000	-0.024309000
6	-2.386749000	-0.612087000	0.049519000
6	-2.271245000	0.663486000	0.632255000
6	-3.631375000	-0.976581000	-0.497197000
6	-3.344833000	1.545140000	0.628494000
6	-4.698593000	-0.090788000	-0.506202000
6	-4.558670000	1.179435000	0.051366000
1	-1.351717000	0.948749000	1.127961000
1	-3.745622000	-1.966733000	-0.926668000
1	-3.236112000	2.520267000	1.090050000
1	-5.643821000	-0.391584000	-0.943786000
1	-5.393030000	1.871067000	0.049245000
6	0.729448000	-0.165799000	-0.112294000
1	0.161219000	0.726888000	-0.351119000
6	2.165813000	0.004975000	-0.070912000
6	2.712023000	1.246880000	-0.451011000
6	3.056686000	-1.007957000	0.339439000
6	4.082204000	1.463052000	-0.439077000
6	4.425389000	-0.786708000	0.349363000
6	4.948399000	0.446551000	-0.040949000
1	2.040806000	2.040416000	-0.763194000
1	2.675807000	-1.968897000	0.664882000
1	4.476892000	2.426427000	-0.741679000
1	5.092082000	-1.579190000	0.670278000
1	6.019000000	0.613814000	-0.029436000

TS rotation (from radical):

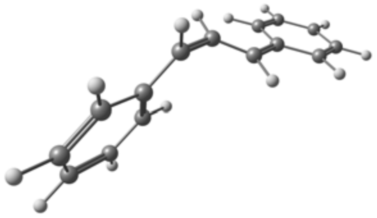

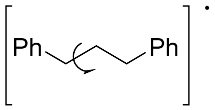

TS

Sum of electronic and thermal Free Energies= **-579.076304**

6	0.021821000	-0.738304000	0.794819000
6	-1.347786000	-1.263188000	0.648727000
1	0.289010000	-0.327424000	1.769872000
1	-1.529984000	-2.302939000	0.906916000
6	-2.462242000	-0.473704000	0.262047000
6	-2.340503000	0.901836000	-0.058310000
6	-3.753198000	-1.056911000	0.185522000
6	-3.449906000	1.643338000	-0.429078000
6	-4.852786000	-0.306551000	-0.188740000
6	-4.711816000	1.049359000	-0.497947000
1	-1.364753000	1.372317000	-0.009833000
1	-3.865785000	-2.109090000	0.426043000
1	-3.335137000	2.694929000	-0.667472000
1	-5.829744000	-0.773934000	-0.242597000
1	-5.575675000	1.635174000	-0.789320000
6	0.928108000	-0.767730000	-0.186763000
1	0.644366000	-1.208273000	-1.140979000
6	2.318089000	-0.279550000	-0.111075000
6	3.210014000	-0.642131000	-1.126863000
6	2.786805000	0.534714000	0.928909000
6	4.535963000	-0.222502000	-1.099350000
6	4.110392000	0.954234000	0.956096000
6	4.991903000	0.575868000	-0.055685000
1	2.856151000	-1.264189000	-1.942793000
1	2.113150000	0.855131000	1.715681000
1	5.210689000	-0.518394000	-1.894518000
1	4.455178000	1.586023000	1.766870000
1	6.023396000	0.907994000	-0.031740000

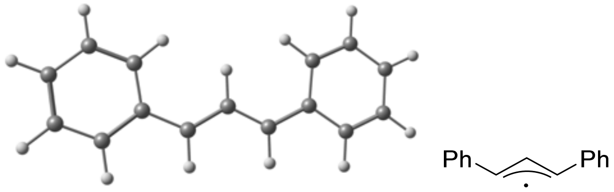

Sum of electronic and thermal Free Energies= **-579.099985**

6	0.000000000	0.312101000	-0.000003000
6	1.216301000	0.976777000	-0.000144000
1	0.000001000	-0.773560000	0.000191000
1	1.198857000	2.064096000	-0.000360000
6	2.527275000	0.364347000	-0.000076000
6	3.665361000	1.194746000	-0.000422000
6	2.730028000	-1.030707000	0.000316000
6	4.945150000	0.658828000	-0.000403000
6	4.010756000	-1.561119000	0.000329000
6	5.126412000	-0.722774000	-0.000034000
1	3.527315000	2.271152000	-0.000710000
1	1.881488000	-1.704504000	0.000622000
1	5.805000000	1.319202000	-0.000671000
1	4.143878000	-2.637094000	0.000627000
1	6.125301000	-1.142963000	-0.000022000
6	-1.216300000	0.976776000	-0.000079000
1	-1.198857000	2.064095000	-0.000154000
6	-2.527276000	0.364347000	-0.000014000
6	-3.665360000	1.194746000	0.000394000
6	-2.730028000	-1.030707000	-0.000365000
6	-4.945150000	0.658829000	0.000513000
6	-4.010756000	-1.561119000	-0.000232000
6	-5.126412000	-0.722774000	0.000217000
1	-3.527315000	2.271152000	0.000626000
1	-1.881488000	-1.704504000	-0.000783000
1	-5.805000000	1.319203000	0.000828000
1	-4.143879000	-2.637094000	-0.000499000
1	-6.125301000	-1.142963000	0.000319000

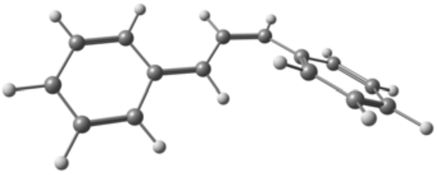

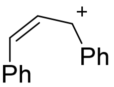

Sum of electronic and thermal Free Energies= **-578.920114**

6	0.052429000	-1.488720000	0.055688000
6	-1.322400000	-1.642206000	-0.012160000
1	0.641355000	-2.389717000	0.182199000
1	-1.686878000	-2.660318000	-0.131542000
6	-2.338905000	-0.633703000	0.059639000
6	-2.154938000	0.604341000	0.710783000
6	-3.588921000	-0.927751000	-0.524895000
6	-3.175549000	1.539246000	0.716519000
6	-4.594354000	0.021172000	-0.533637000
6	-4.385775000	1.255257000	0.083984000
1	-1.240961000	0.800191000	1.258544000
1	-3.737893000	-1.896658000	-0.989143000
1	-3.040356000	2.483076000	1.230144000
1	-5.544771000	-0.197069000	-1.004819000
1	-5.182211000	1.990673000	0.095762000
6	0.693187000	-0.281038000	-0.176533000
1	0.095519000	0.563940000	-0.508710000
6	2.100710000	-0.047394000	-0.113988000
6	2.575372000	1.213937000	-0.529717000
6	3.016386000	-1.012712000	0.359440000
6	3.927807000	1.502441000	-0.480675000
6	4.363611000	-0.715128000	0.408704000
6	4.818918000	0.538412000	-0.012176000
1	1.864874000	1.950390000	-0.889511000
1	2.666755000	-1.982803000	0.691284000
1	4.291486000	2.469952000	-0.803328000
1	5.068776000	-1.450984000	0.774937000
1	5.878825000	0.762403000	0.028324000

TS rotation (from Carbocation):

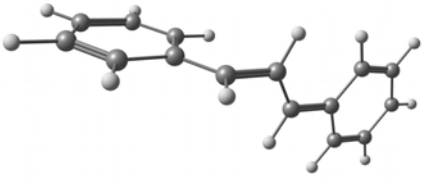

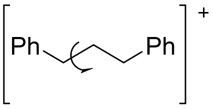

Sum of electronic and thermal Free Energies= **-578.895332**

6	0.007326000	-0.916691000	0.498816000
6	-1.359398000	-1.337703000	0.195461000
1	0.224413000	-0.841051000	1.565024000
1	-1.541388000	-2.404951000	0.058502000
6	-2.451202000	-0.497209000	0.101373000
6	-2.323691000	0.916794000	0.258977000
6	-3.730665000	-1.072698000	-0.165546000
6	-3.438231000	1.710017000	0.152936000
6	-4.836332000	-0.263127000	-0.267843000
6	-4.684753000	1.119285000	-0.107389000
1	-1.347670000	1.344127000	0.457759000
1	-3.803866000	-2.148245000	-0.283498000
1	-3.365387000	2.783468000	0.269449000
1	-5.813660000	-0.682294000	-0.468225000
1	-5.559057000	1.756336000	-0.186225000
6	0.920142000	-0.665017000	-0.441156000
1	0.633262000	-0.757012000	-1.485948000
6	2.312266000	-0.247691000	-0.203802000
6	3.111974000	0.043305000	-1.313679000
6	2.867277000	-0.132973000	1.077346000
6	4.434866000	0.441850000	-1.152466000
6	4.187717000	0.262753000	1.237048000
6	4.976165000	0.552282000	0.123613000
1	2.688308000	-0.046352000	-2.308601000
1	2.271518000	-0.357707000	1.955222000
1	5.040254000	0.666255000	-2.023076000
1	4.605889000	0.346500000	2.233558000
1	6.006615000	0.861873000	0.253922000

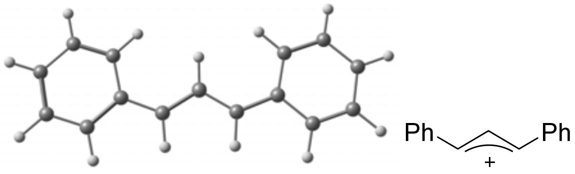

Sum of electronic and thermal Free Energies= **-578.929260**

6	-0.000076000	-0.096584000	0.000052000
6	1.182930000	-0.815095000	-0.000507000
1	-0.000161000	0.986151000	-0.000557000
1	1.098158000	-1.900322000	-0.000864000
6	2.515468000	-0.298198000	-0.000727000
6	3.575297000	-1.227866000	-0.000617000
6	2.805857000	1.083310000	-0.000411000
6	4.888876000	-0.793102000	-0.000055000
6	4.119833000	1.509090000	0.000198000
6	5.159064000	0.574216000	0.000380000
1	3.344549000	-2.287611000	-0.000716000
1	2.005540000	1.813077000	-0.000600000
1	5.701345000	-1.508994000	0.000035000
1	4.345904000	2.568229000	0.000580000
1	6.186984000	0.918361000	0.000819000
6	-1.182959000	-0.815231000	0.001225000
1	-1.098199000	-1.900445000	0.002149000
6	-2.515490000	-0.298240000	0.001011000
6	-3.575337000	-1.227873000	0.000479000
6	-2.805793000	1.083293000	0.000776000
6	-4.888919000	-0.793041000	-0.000598000
6	-4.119734000	1.509115000	-0.000202000
6	-5.159017000	0.574272000	-0.000956000
1	-3.344592000	-2.287616000	0.000779000
1	-2.005375000	1.812950000	0.001463000
1	-5.701437000	-1.508876000	-0.001139000
1	-4.345781000	2.568260000	-0.000427000
1	-6.186930000	0.918448000	-0.001807000

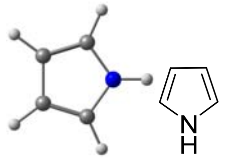

Sum of electronic and thermal Free Energies=**-210.087629**

6	0.000000000	0.712215000	-0.982027000
6	0.000000000	1.119523000	0.332855000
7	0.000000000	0.000000000	1.115698000
6	0.000000000	-1.119523000	0.332855000
6	0.000000000	-0.712215000	-0.982027000
1	0.000000000	1.361588000	-1.844462000
1	0.000000000	2.104471000	0.771895000
1	0.000000000	-2.104471000	0.771895000
1	0.000000000	-1.361588000	-1.844462000
1	0.000000000	0.000000000	2.125318000

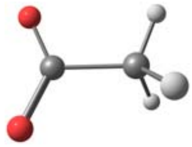

CH_3COO^-

Sum of electronic and thermal Free Energies=**-228.551332**

8	1.151635000	0.691688000	0.000000000
8	-1.091321000	0.810425000	0.000000000
6	0.000000000	0.203469000	0.000000000
6	-0.056509000	-1.343830000	0.000000000
1	0.468938000	-1.728304000	0.878985000
1	-1.081329000	-1.718132000	0.000000000
1	0.468938000	-1.728304000	-0.878985000

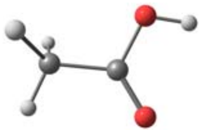

CH_3COOH

Sum of electronic and thermal Free Energies=**-229.031532**

1	1.869351000	0.365180000	0.000000000
8	1.237425000	-0.371014000	0.000000000
8	-0.194660000	1.339146000	0.000000000
6	0.000000000	0.152210000	0.000000000
6	-1.052373000	-0.913117000	0.000000000
1	-0.929331000	-1.544346000	0.882028000
1	-2.038575000	-0.456099000	0.000000000
1	-0.929331000	-1.544346000	-0.882028000

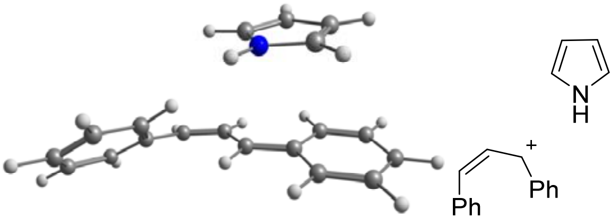

Sum of electronic and thermal Free Energies= **-788.998001**

6	0.530555000	-1.138067000	-1.226827000
6	1.903497000	-1.093081000	-1.341560000
1	-0.018211000	-1.546716000	-2.066922000
1	2.322342000	-1.601727000	-2.207544000
6	2.881259000	-0.482768000	-0.474825000
6	2.608756000	0.614371000	0.365765000
6	4.188315000	-1.007250000	-0.511444000
6	3.603546000	1.129388000	1.180234000
6	5.171327000	-0.502085000	0.322224000
6	4.878035000	0.564058000	1.171529000
1	1.638418000	1.092700000	0.340120000
1	4.408116000	-1.827409000	-1.185964000
1	3.391945000	1.983065000	1.812598000
1	6.168312000	-0.924887000	0.303114000
1	5.651414000	0.971355000	1.812447000
6	-0.153234000	-0.827331000	-0.052015000
1	0.432654000	-0.573720000	0.826885000
6	-1.555550000	-0.931612000	0.164718000
6	-2.029426000	-0.701944000	1.474129000
6	-2.476424000	-1.261900000	-0.855608000
6	-3.377412000	-0.824592000	1.764938000
6	-3.818866000	-1.381061000	-0.557460000
6	-4.267647000	-1.168049000	0.750357000
1	-1.319623000	-0.436541000	2.250417000
1	-2.134038000	-1.425338000	-1.870315000
1	-3.736678000	-0.654764000	2.772238000
1	-4.526168000	-1.638343000	-1.336089000
1	-5.323800000	-1.267660000	0.974177000
6	-1.730445000	1.998595000	-1.743995000
6	-0.546177000	2.166024000	-1.062008000
7	-0.850219000	2.392088000	0.253699000
6	-2.203423000	2.376775000	0.428840000
6	-2.783935000	2.130183000	-0.797098000
1	-1.826921000	1.800356000	-2.800889000
1	0.477078000	2.156946000	-1.403905000
1	-2.641168000	2.537369000	1.401382000
1	-3.843468000	2.051860000	-0.988838000
1	-0.174797000	2.586952000	0.979705000

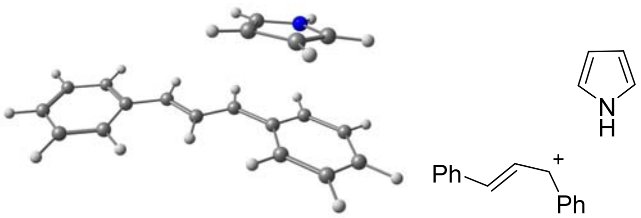

Sum of electronic and thermal Free Energies= **-789.007486**

6	-0.735266000	-0.584050000	-0.278573000
6	-1.866088000	-0.054678000	-0.854767000
1	-0.786762000	-1.146130000	0.645700000
1	-1.739001000	0.502078000	-1.781736000
6	-3.213601000	-0.151819000	-0.365177000
6	-4.230026000	0.486951000	-1.100064000
6	-3.548593000	-0.851734000	0.811404000
6	-5.545955000	0.432809000	-0.670347000
6	-4.863999000	-0.903383000	1.233476000
6	-5.861742000	-0.261464000	0.495512000
1	-3.968055000	1.024702000	-2.004901000
1	-2.779907000	-1.352608000	1.387026000
1	-6.324884000	0.928074000	-1.236951000
1	-5.121624000	-1.442853000	2.136687000
1	-6.890701000	-0.305360000	0.833657000
6	0.483575000	-0.389072000	-0.924419000
1	0.462170000	0.168743000	-1.858308000
6	1.754144000	-0.907698000	-0.534671000
6	2.839340000	-0.719887000	-1.414598000
6	1.960850000	-1.597142000	0.679754000
6	4.087655000	-1.235641000	-1.109183000
6	3.211551000	-2.100169000	0.982384000
6	4.270740000	-1.926373000	0.087002000
1	2.676239000	-0.177273000	-2.339707000
1	1.145476000	-1.728850000	1.380579000
1	4.915502000	-1.100332000	-1.794019000
1	3.372590000	-2.627744000	1.914372000
1	5.247546000	-2.329246000	0.329968000
6	1.095501000	2.040743000	1.162246000
6	1.296733000	2.435810000	-0.145451000
7	2.627131000	2.305277000	-0.418031000
6	3.285268000	1.825976000	0.677437000
6	2.358181000	1.654152000	1.684384000
1	0.146219000	2.026280000	1.676383000
1	0.608582000	2.809265000	-0.887636000
1	4.348801000	1.648009000	0.653879000
1	2.566774000	1.284969000	2.677074000
1	3.061517000	2.521333000	-1.303955000

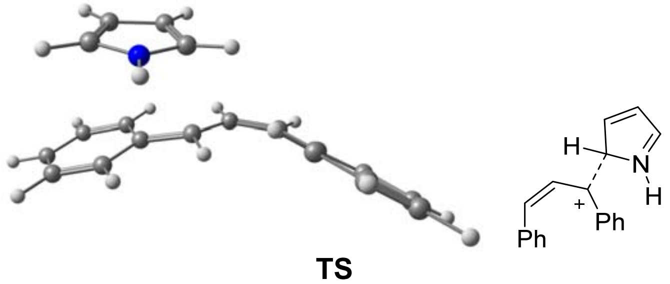

TS

Sum of electronic and thermal Free Energies= **-788.992009**

6	0.494244000	-0.910468000	-1.231057000
6	1.848210000	-0.932725000	-1.352261000
1	-0.069373000	-1.339732000	-2.051213000
1	2.215835000	-1.432190000	-2.245595000
6	2.922286000	-0.472170000	-0.474426000
6	2.810757000	0.540556000	0.493354000
6	4.171291000	-1.094462000	-0.646947000
6	3.902698000	0.888264000	1.276667000
6	5.255819000	-0.756549000	0.149265000
6	5.122908000	0.235572000	1.116882000
1	1.888926000	1.089036000	0.625493000
1	4.277668000	-1.858077000	-1.410234000
1	3.801257000	1.677442000	2.012289000
1	6.205979000	-1.258101000	0.008868000
1	5.970072000	0.509381000	1.735035000
6	-0.234643000	-0.466740000	-0.089645000
1	0.331699000	-0.112198000	0.764434000
6	-1.589117000	-0.831564000	0.208046000
6	-2.073354000	-0.554287000	1.500506000
6	-2.443636000	-1.466983000	-0.719169000
6	-3.357710000	-0.931716000	1.872634000
6	-3.719610000	-1.837884000	-0.343526000
6	-4.176557000	-1.574532000	0.952716000
1	-1.418901000	-0.061302000	2.211555000
1	-2.101334000	-1.672294000	-1.726092000
1	-3.715420000	-0.724619000	2.873717000
1	-4.369621000	-2.332750000	-1.054778000
1	-5.179257000	-1.871672000	1.238038000
6	-1.460167000	1.627465000	-1.677425000
6	-0.543742000	1.894888000	-0.655326000
7	-1.267077000	2.262479000	0.449771000
6	-2.588759000	2.155267000	0.191774000
6	-2.743403000	1.771034000	-1.140128000
1	-1.197406000	1.322537000	-2.679374000
1	0.523490000	2.040955000	-0.699300000
1	-3.325348000	2.359841000	0.953177000
1	-3.685930000	1.603628000	-1.637790000
1	-0.867883000	2.524959000	1.341762000

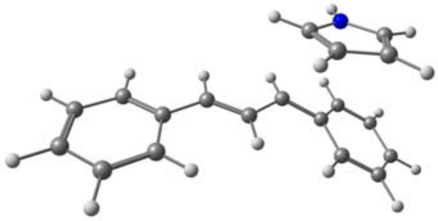

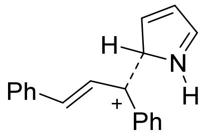

TS

Sum of electronic and thermal Free Energies= **-789.000972**

6	-0.686246000	-0.264096000	-0.162239000
6	-1.813560000	0.184345000	-0.762858000
1	-0.728809000	-0.855630000	0.744936000
1	-1.703607000	0.773108000	-1.672319000
6	-3.185959000	-0.049483000	-0.334043000
6	-4.224357000	0.418897000	-1.152065000
6	-3.509365000	-0.718791000	0.857714000
6	-5.551727000	0.220090000	-0.795920000
6	-4.834998000	-0.912199000	1.212213000
6	-5.859008000	-0.445732000	0.386923000
1	-3.977959000	0.938878000	-2.071860000
1	-2.724666000	-1.083902000	1.509639000
1	-6.344602000	0.584512000	-1.438227000
1	-5.075499000	-1.428144000	2.134270000
1	-6.893974000	-0.601299000	0.668819000
6	0.585103000	0.026284000	-0.749226000
1	0.555589000	0.554059000	-1.699045000
6	1.802991000	-0.698291000	-0.485802000
6	2.882462000	-0.525049000	-1.369268000
6	1.949805000	-1.577567000	0.605780000
6	4.063485000	-1.235841000	-1.193422000
6	3.127646000	-2.280688000	0.777518000
6	4.183872000	-2.114539000	-0.123666000
1	2.774094000	0.158171000	-2.204960000
1	1.141304000	-1.708454000	1.315034000
1	4.883186000	-1.103695000	-1.888926000
1	3.232218000	-2.962172000	1.613059000
1	5.103448000	-2.670779000	0.017915000
6	1.245232000	1.649115000	1.430242000
6	1.079568000	2.132931000	0.123180000
7	2.335931000	2.341884000	-0.386396000
6	3.266570000	1.919432000	0.494226000
6	2.614511000	1.492315000	1.651850000
1	0.436880000	1.397643000	2.100569000
1	0.208836000	2.541724000	-0.363844000
1	4.318156000	1.950045000	0.253124000
1	3.095377000	1.094480000	2.531537000
1	2.532363000	2.701457000	-1.311764000

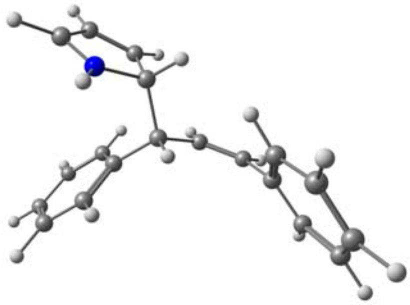

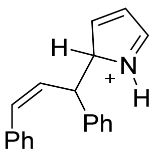

Sum of electronic and thermal Free Energies= **-789.020343**

6	0.382681000	-0.110279000	1.512330000
6	1.693308000	0.037468000	1.723116000
1	-0.270270000	-0.078730000	2.380631000
1	2.009029000	0.215130000	2.748715000
6	2.788435000	0.054173000	0.729811000
6	2.864255000	-0.844759000	-0.340046000
6	3.817065000	0.990950000	0.890856000
6	3.922383000	-0.784725000	-1.241879000
6	4.869407000	1.056301000	-0.013680000
6	4.923218000	0.169902000	-1.086693000
1	2.108729000	-1.614233000	-0.452458000
1	3.781281000	1.676122000	1.731508000
1	3.969318000	-1.492122000	-2.062001000
1	5.651092000	1.795115000	0.121522000
1	5.746803000	0.214864000	-1.789874000
6	-0.312934000	-0.244341000	0.182264000
1	0.408588000	-0.111493000	-0.626109000
6	-1.407693000	0.795830000	0.002158000
6	-1.421771000	1.574664000	-1.156253000
6	-2.406648000	0.993458000	0.959565000
6	-2.410651000	2.533812000	-1.356797000
6	-3.396815000	1.950295000	0.759241000
6	-3.401562000	2.723125000	-0.398807000
1	-0.648536000	1.430094000	-1.904260000
1	-2.417247000	0.406432000	1.872126000
1	-2.404193000	3.133055000	-2.260136000
1	-4.163747000	2.093702000	1.511810000
1	-4.171457000	3.470864000	-0.550813000
6	-1.885027000	-2.144163000	1.005082000
6	-0.865896000	-1.689692000	0.019890000
7	-1.584187000	-1.852093000	-1.235969000
6	-2.779139000	-2.310947000	-1.043712000
6	-3.010405000	-2.516614000	0.364982000
1	-1.696617000	-2.168988000	2.069814000
1	-0.016322000	-2.385232000	0.023894000
1	-3.455130000	-2.497433000	-1.868599000
1	-3.927773000	-2.900405000	0.782989000
1	-1.187497000	-1.628839000	-2.145552000

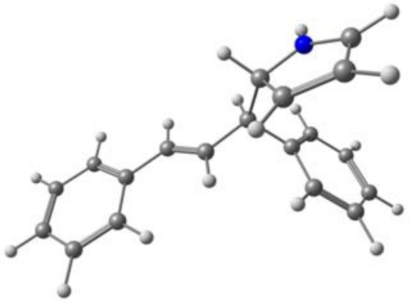

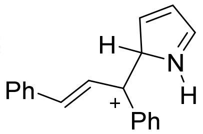

Sum of electronic and thermal Free Energies= **-789.026656**

6	0.618745000	-0.218507000	-0.092446000
6	1.729553000	-0.158477000	-0.828681000
1	0.662074000	-0.152353000	0.991766000
1	1.640046000	-0.245253000	-1.910514000
6	3.106273000	0.011715000	-0.329569000
6	4.164655000	-0.108332000	-1.237122000
6	3.404162000	0.293747000	1.010956000
6	5.484330000	0.037138000	-0.821573000
6	4.721026000	0.440317000	1.425321000
6	5.767136000	0.311085000	0.512272000
1	3.944891000	-0.319359000	-2.278814000
1	2.605021000	0.409757000	1.734375000
1	6.289349000	-0.062397000	-1.540711000
1	4.934280000	0.661157000	2.465153000
1	6.793363000	0.428046000	0.840677000
6	-0.746598000	-0.377444000	-0.705518000
1	-0.640696000	-0.382771000	-1.793182000
6	-1.708436000	0.735829000	-0.325721000
6	-2.473905000	1.349157000	-1.318666000
6	-1.861118000	1.152947000	0.998849000
6	-3.373485000	2.361597000	-0.998059000
6	-2.760240000	2.164586000	1.320829000
6	-3.518697000	2.771457000	0.323485000
1	-2.359889000	1.031578000	-2.350499000
1	-1.274348000	0.691361000	1.786247000
1	-3.957200000	2.831030000	-1.781659000
1	-2.866056000	2.480843000	2.352166000
1	-4.215883000	3.562270000	0.575048000
6	-1.587737000	-2.032212000	1.107261000
6	-1.329345000	-1.775983000	-0.335700000
7	-2.655218000	-1.966633000	-0.905685000
6	-3.525508000	-2.264763000	0.004852000
6	-2.889209000	-2.323289000	1.298036000
1	-0.809403000	-1.985491000	1.857137000
1	-0.659313000	-2.542766000	-0.743658000
1	-4.565905000	-2.438810000	-0.239469000
1	-3.399139000	-2.560053000	2.218614000
1	-2.864266000	-1.863772000	-1.895724000

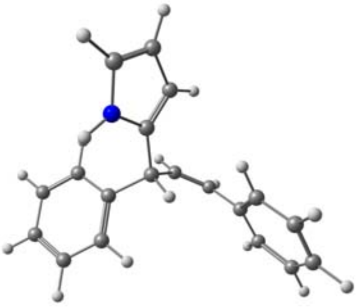

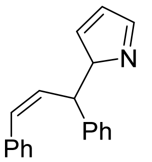

Sum of electronic and thermal Free Energies= **-788.609420**

6	0.168951000	0.046468000	1.461149000
6	1.467306000	0.252408000	1.693725000
1	-0.513756000	0.077320000	2.307899000
1	1.762311000	0.479827000	2.715703000
6	2.571613000	0.258572000	0.709864000
6	2.676574000	-0.687425000	-0.317103000
6	3.573952000	1.228911000	0.830976000
6	3.737293000	-0.639815000	-1.216691000
6	4.630422000	1.279430000	-0.070294000
6	4.713102000	0.346585000	-1.101075000
1	1.936922000	-1.477859000	-0.396090000
1	3.513659000	1.952925000	1.637172000
1	3.807122000	-1.381858000	-2.004110000
1	5.392151000	2.043785000	0.034646000
1	5.539018000	0.380747000	-1.802204000
6	-0.500996000	-0.162415000	0.123186000
1	0.231250000	-0.002460000	-0.673335000
6	-1.583023000	0.898242000	-0.048332000
6	-1.316834000	2.036446000	-0.808920000
6	-2.823332000	0.781652000	0.584216000
6	-2.271932000	3.041021000	-0.941850000
6	-3.777710000	1.785622000	0.455144000
6	-3.505202000	2.917867000	-0.309484000
1	-0.353070000	2.134937000	-1.299108000
1	-3.043934000	-0.100971000	1.177096000
1	-2.050977000	3.917893000	-1.540026000
1	-4.737168000	1.682472000	0.949531000
1	-4.251541000	3.697092000	-0.413910000
6	-0.749637000	-2.700649000	0.746763000
6	-1.037161000	-1.566021000	0.016831000
7	-1.944866000	-1.920103000	-0.944556000
6	-2.244578000	-3.251257000	-0.847321000
6	-1.515758000	-3.769874000	0.196570000
1	-0.065389000	-2.748707000	1.580985000
1	-2.949960000	-3.710959000	-1.521384000
1	-1.533046000	-4.795591000	0.532657000
1	-2.347836000	-1.282468000	-1.617756000

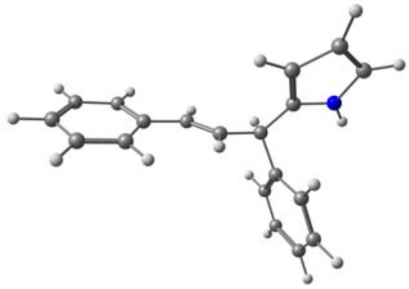

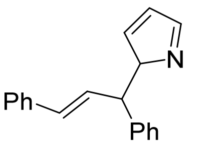

Sum of electronic and thermal Free Energies= **-788.613642**

6	0.412058000	0.180111000	0.060478000
6	1.523851000	0.101572000	0.793359000
1	0.449156000	0.158462000	-1.027116000
1	1.433079000	0.145852000	1.877517000
6	2.903000000	-0.026840000	0.286888000
6	3.962673000	0.080065000	1.194951000
6	3.203601000	-0.253903000	-1.063512000
6	5.283723000	-0.024424000	0.771251000
6	4.521948000	-0.358323000	-1.486603000
6	5.568669000	-0.242257000	-0.572533000
1	3.742865000	0.249553000	2.244170000
1	2.403953000	-0.359245000	-1.788023000
1	6.088765000	0.064518000	1.491928000
1	4.735574000	-0.536208000	-2.534573000
1	6.596062000	-0.325862000	-0.907502000
6	-0.967953000	0.285511000	0.660422000
1	-0.863282000	0.273660000	1.750905000
6	-1.779128000	-0.946165000	0.267223000
6	-1.810245000	-2.049910000	1.120063000
6	-2.447959000	-1.017758000	-0.956594000
6	-2.502144000	-3.203503000	0.762917000
6	-3.137294000	-2.171787000	-1.317084000
6	-3.168045000	-3.267131000	-0.457820000
1	-1.287695000	-2.001514000	2.070631000
1	-2.433625000	-0.164131000	-1.627264000
1	-2.522862000	-4.051170000	1.438520000
1	-3.654702000	-2.214307000	-2.268929000
1	-3.710671000	-4.163071000	-0.736879000
6	-1.128453000	2.691415000	-0.382815000
6	-1.630515000	1.572907000	0.247845000
7	-2.952428000	1.812658000	0.510855000
6	-3.307508000	3.055626000	0.064016000
6	-2.193558000	3.632576000	-0.498258000
1	-0.109321000	2.816991000	-0.717093000
1	-4.315494000	3.420762000	0.181218000
1	-2.145359000	4.615172000	-0.942593000
1	-3.579783000	1.155240000	0.953937000

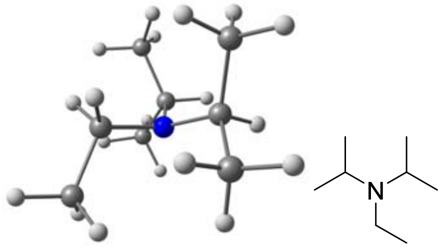

DIPEA

Sum of electronic and thermal Free Energies=**-370.726615**

7	0.002988000	0.127825000	-0.264268000
6	1.293760000	-0.552972000	-0.433450000
1	1.121256000	-1.325152000	-1.190385000
6	1.874866000	-1.243721000	0.813653000
6	2.325526000	0.430112000	-0.989392000
1	1.976269000	0.864206000	-1.928897000
1	2.496329000	1.243595000	-0.276766000
1	3.283899000	-0.066730000	-1.164789000
1	2.291852000	-0.515871000	1.515031000
1	2.691440000	-1.906814000	0.514715000
1	1.132592000	-1.839945000	1.345377000
6	-1.180967000	-0.701908000	-0.524030000
1	-0.996731000	-1.179556000	-1.492754000
6	-2.428950000	0.165406000	-0.682864000
6	-1.440793000	-1.820479000	0.496314000
1	-0.653815000	-2.575984000	0.468741000
1	-1.506066000	-1.422793000	1.513651000
1	-2.386975000	-2.320743000	0.271656000
1	-2.707141000	0.646287000	0.259657000
1	-3.271152000	-0.454334000	-0.999711000
1	-2.268969000	0.942403000	-1.433692000
6	-0.084175000	1.033184000	0.883753000
1	-0.855145000	0.697479000	1.588324000
1	0.856243000	1.006032000	1.439385000
6	-0.354514000	2.483231000	0.482356000
1	-1.313723000	2.585601000	-0.028027000
1	0.426971000	2.839383000	-0.194082000
1	-0.366566000	3.131037000	1.364392000

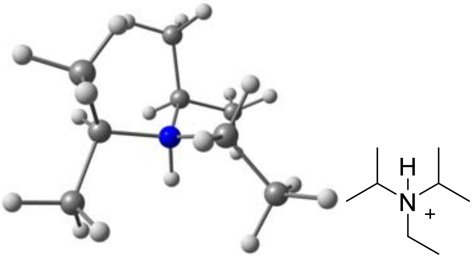

DIPEAH^{+}

Sum of electronic and thermal Free Energies=**-371.183790**

7	0.010457000	0.162654000	-0.314392000
6	1.384636000	-0.503709000	-0.382574000
1	1.228170000	-1.372587000	-1.023364000
6	1.907727000	-0.951910000	0.978528000
6	2.346860000	0.463823000	-1.052130000
1	2.023926000	0.710558000	-2.066273000
1	2.432255000	1.385593000	-0.469252000
1	3.334850000	0.003477000	-1.106493000
1	2.412956000	-0.134987000	1.496727000
1	2.643799000	-1.739787000	0.808543000
1	1.131707000	-1.349343000	1.630779000
6	-1.129417000	-0.827397000	-0.535556000
1	-0.894256000	-1.258509000	-1.511063000
6	-2.456293000	-0.092668000	-0.628912000
6	-1.160257000	-1.934393000	0.504670000
1	-0.277771000	-2.572759000	0.460755000
1	-1.272938000	-1.540638000	1.517459000
1	-2.030960000	-2.556787000	0.290033000
1	-2.767002000	0.307991000	0.338610000
1	-3.212225000	-0.808571000	-0.955824000
1	-2.416928000	0.717089000	-1.360706000
6	-0.156883000	1.090855000	0.867319000
1	-0.785819000	0.584844000	1.598474000
1	0.827168000	1.230665000	1.307440000
6	-0.717109000	2.441886000	0.460724000
1	-1.732208000	2.378585000	0.071707000
1	-0.076910000	2.908418000	-0.292603000
1	-0.728023000	3.088136000	1.340149000
1	-0.028578000	0.761116000	-1.146770000

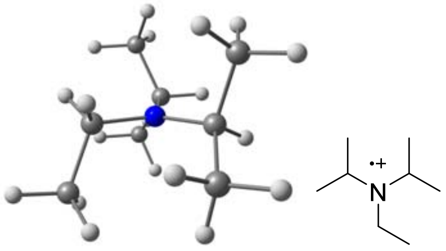

DIPEA$^{\cdot+}$

Sum of electronic and thermal Free Energies=**-370.545410**

7	0.006482000	-0.016793000	0.010274000
6	-1.270790000	-0.615677000	0.414318000
1	-1.009951000	-1.411569000	1.112006000
6	-2.005726000	-1.220927000	-0.786017000
6	-2.129161000	0.430540000	1.129800000
1	-1.595880000	0.872600000	1.972808000
1	-2.439729000	1.217658000	0.439693000
1	-3.022195000	-0.073960000	1.502812000
1	-2.441240000	-0.449834000	-1.422665000
1	-2.817055000	-1.835661000	-0.392423000
1	-1.346180000	-1.854046000	-1.381506000
6	1.235709000	-0.630976000	0.514114000
1	0.992502000	-0.983620000	1.519120000
6	2.426529000	0.311629000	0.575187000
6	1.517799000	-1.856278000	-0.377353000
1	0.690564000	-2.566330000	-0.351849000
1	1.698519000	-1.541739000	-1.407403000
1	2.413669000	-2.345197000	0.008034000
1	2.686866000	0.715786000	-0.405148000
1	3.278023000	-0.267369000	0.936376000
1	2.250796000	1.129350000	1.273915000
6	0.032980000	1.033748000	-0.993290000
1	0.862694000	0.819036000	-1.671165000
1	-0.901034000	0.983884000	-1.549763000
6	0.190966000	2.438112000	-0.388224000
1	1.172338000	2.583859000	0.056479000
1	-0.576715000	2.630443000	0.360567000
1	0.068794000	3.153236000	-1.203005000

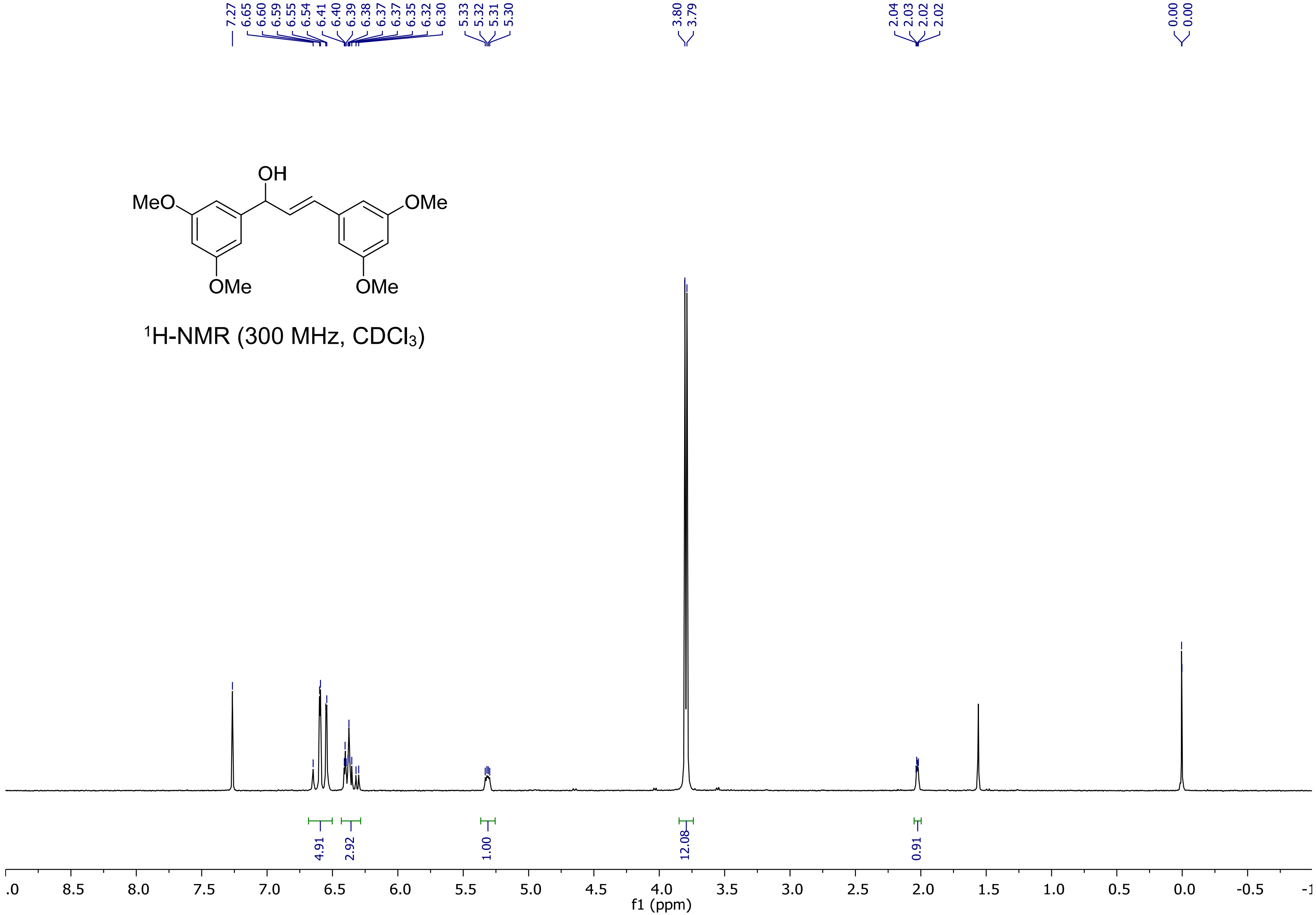

Supplementary Figure 31. ^{1}H-NMR spectra for ***(E)*-1,3-bis(3,5-dimethoxyphenyl)prop-2-en-1-ol**

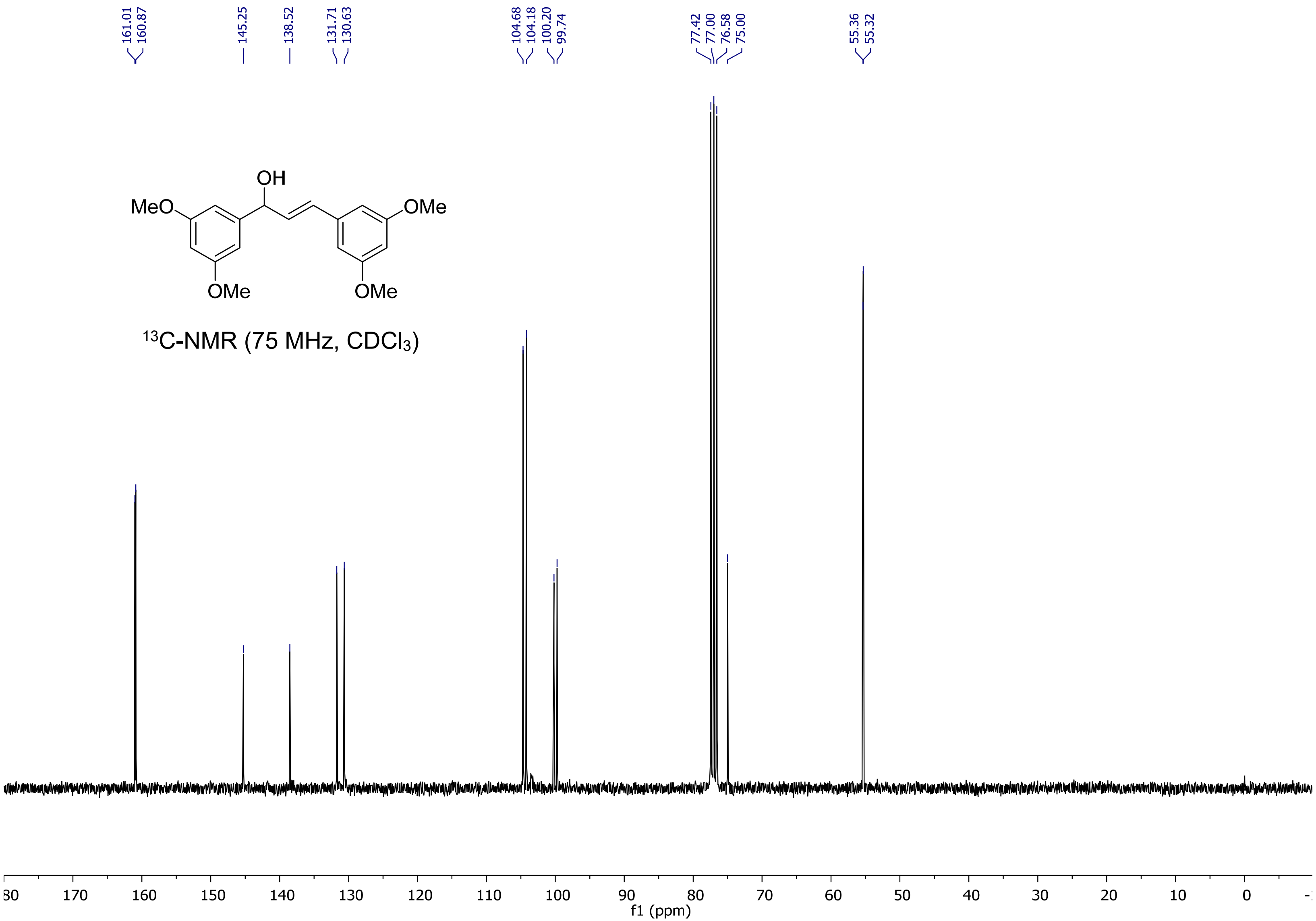

Supplementary Figure 32. ^{13}C-NMR spectra for ***(E)*-1,3-bis(3,5-dimethoxyphenyl)prop-2-en-1-ol**

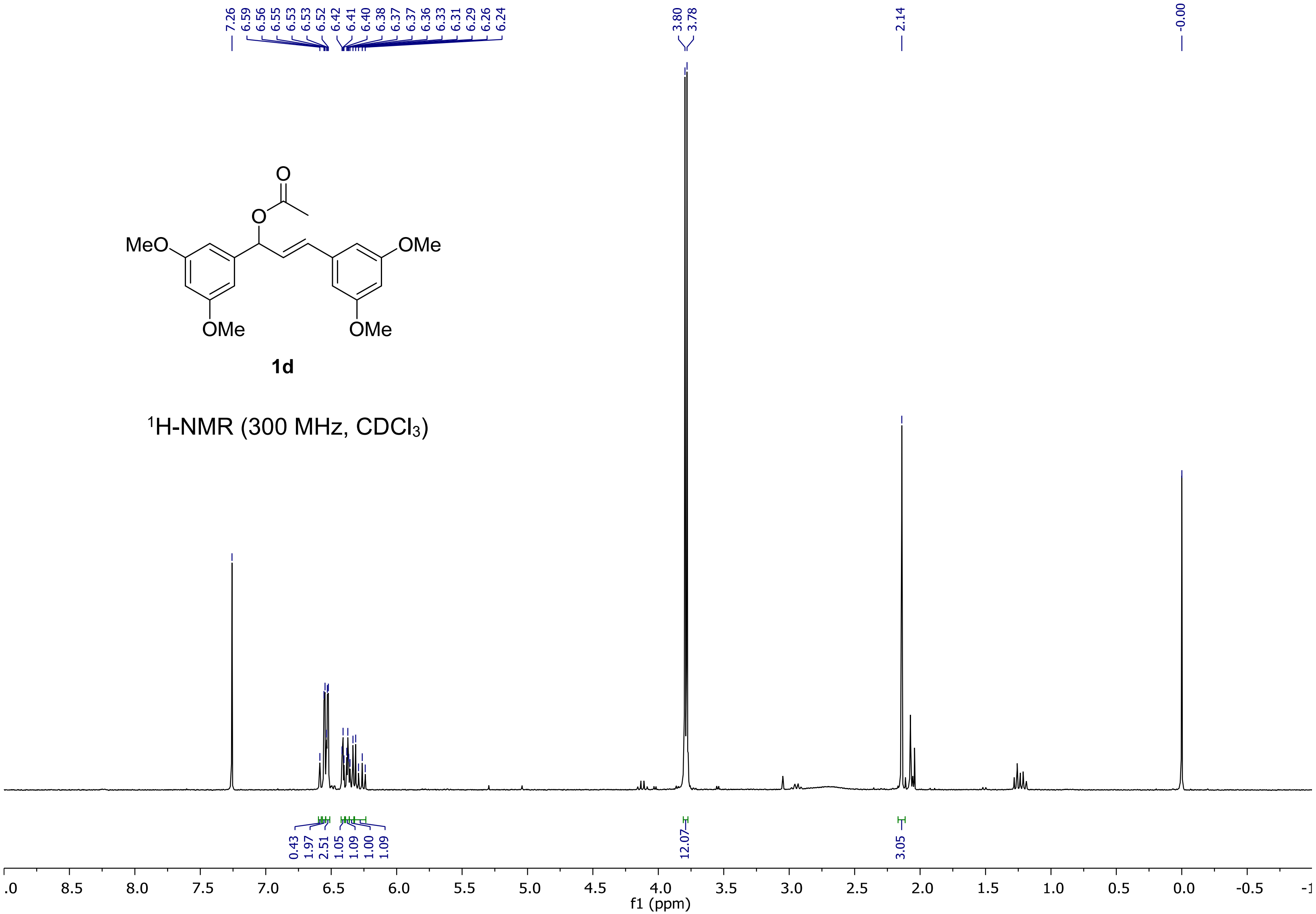

Supplementary Figure 33. ^{1}H-NMR spectra for compound **1d**

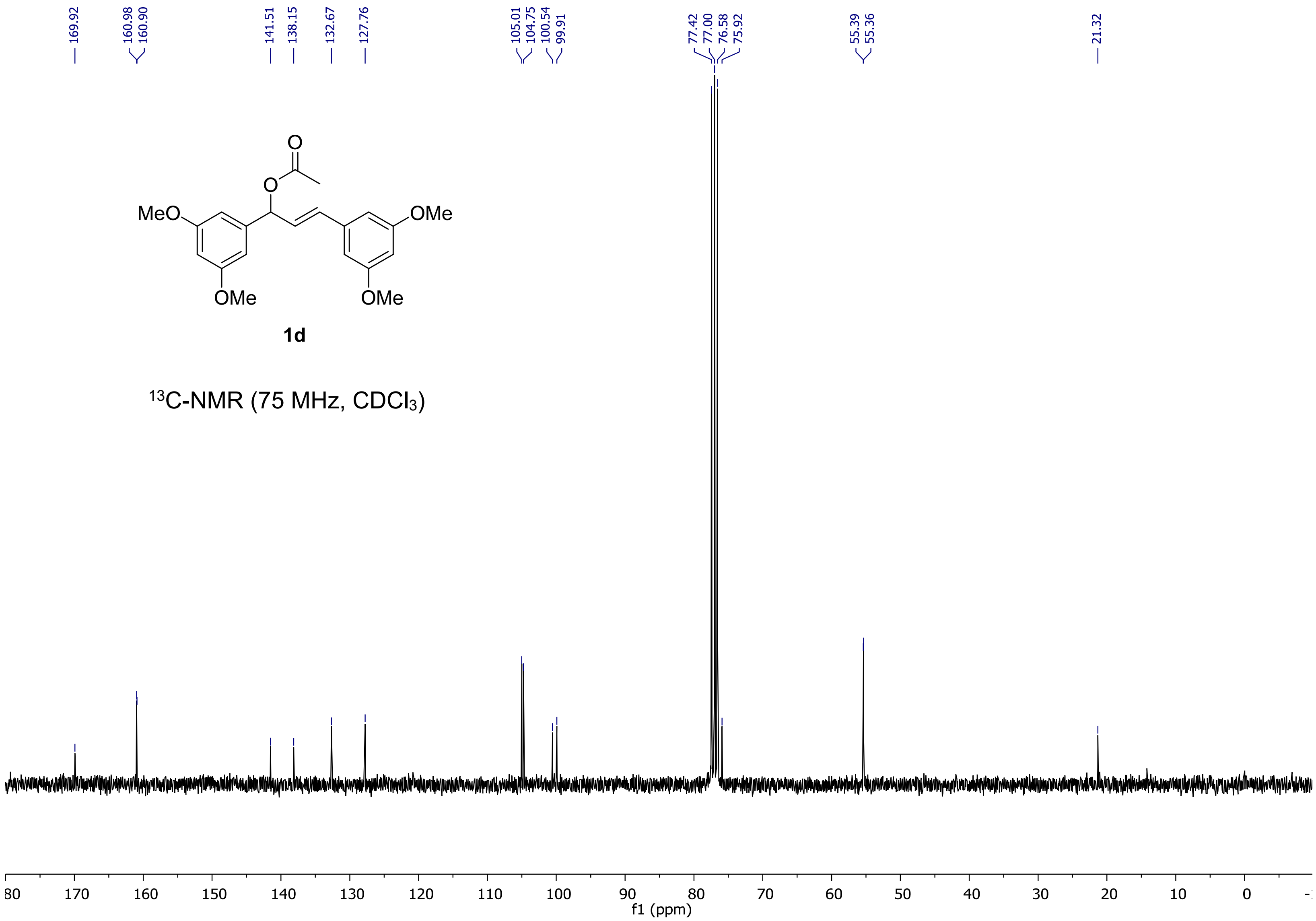

Supplementary Figure 34. ^{13}C-NMR spectra for compound **1d**

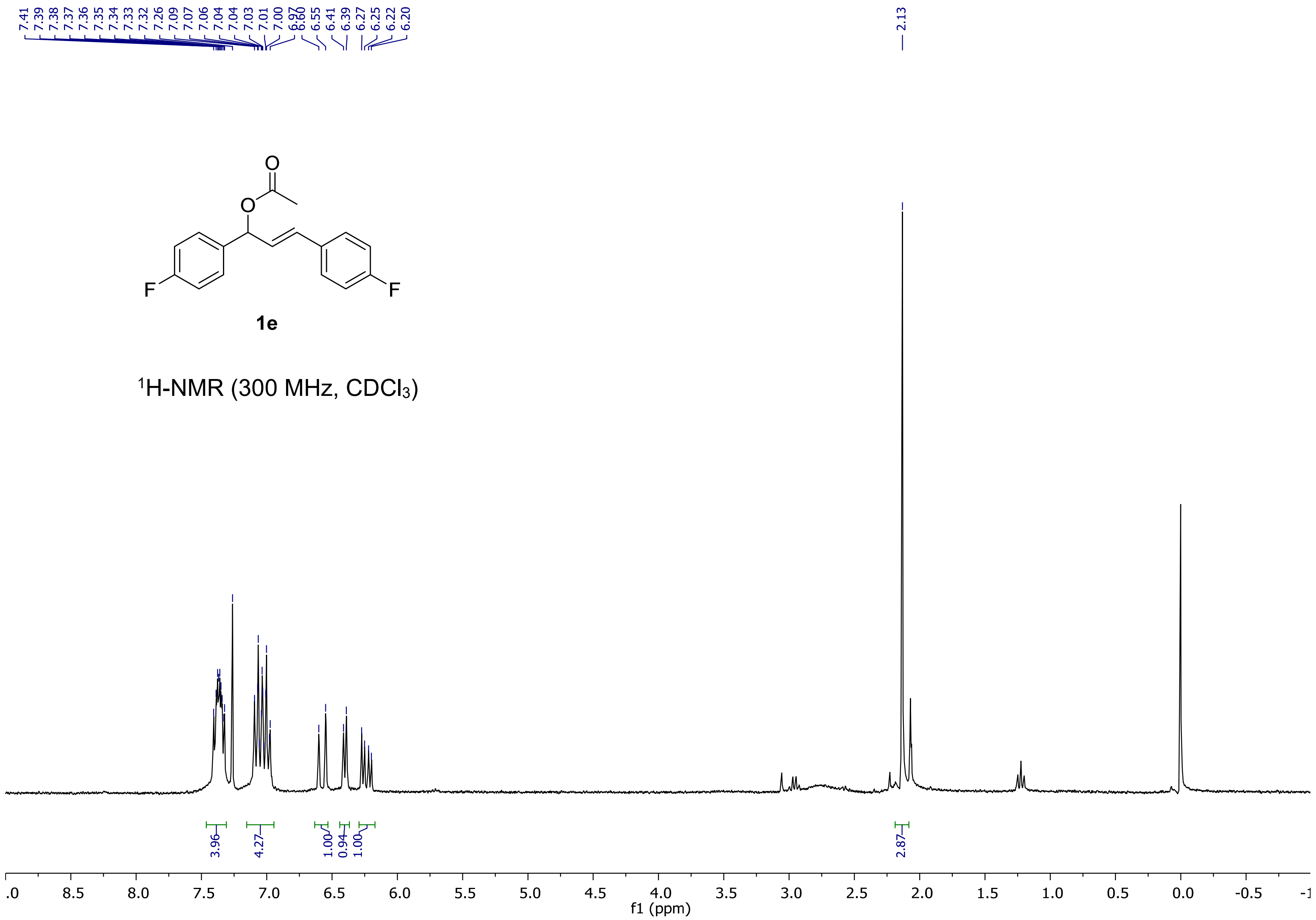

Supplementary Figure 35. ^{1}H-NMR spectra for compound **1e**

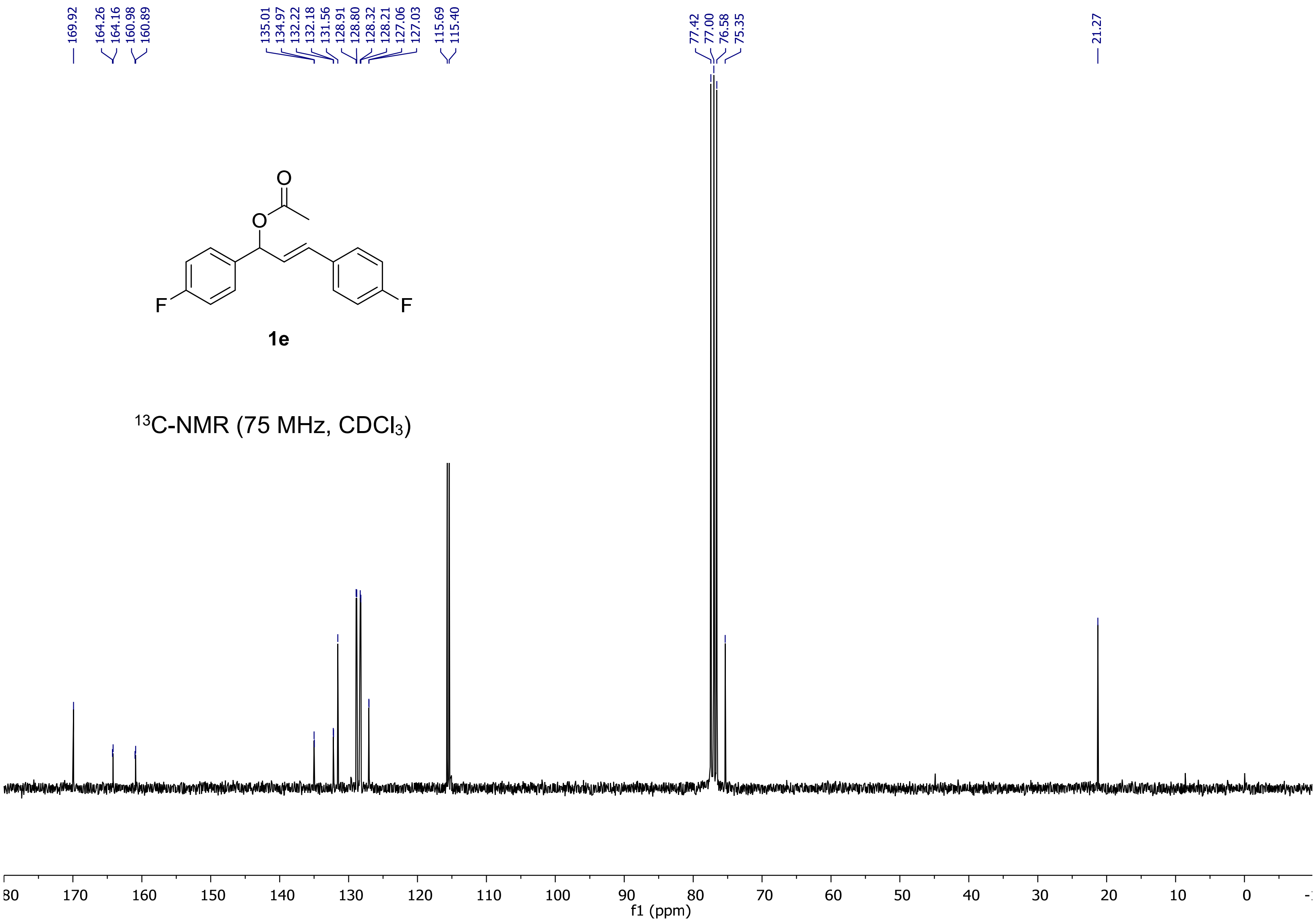

Supplementary Figure 36. ^{13}C-NMR spectra for compound **1e**

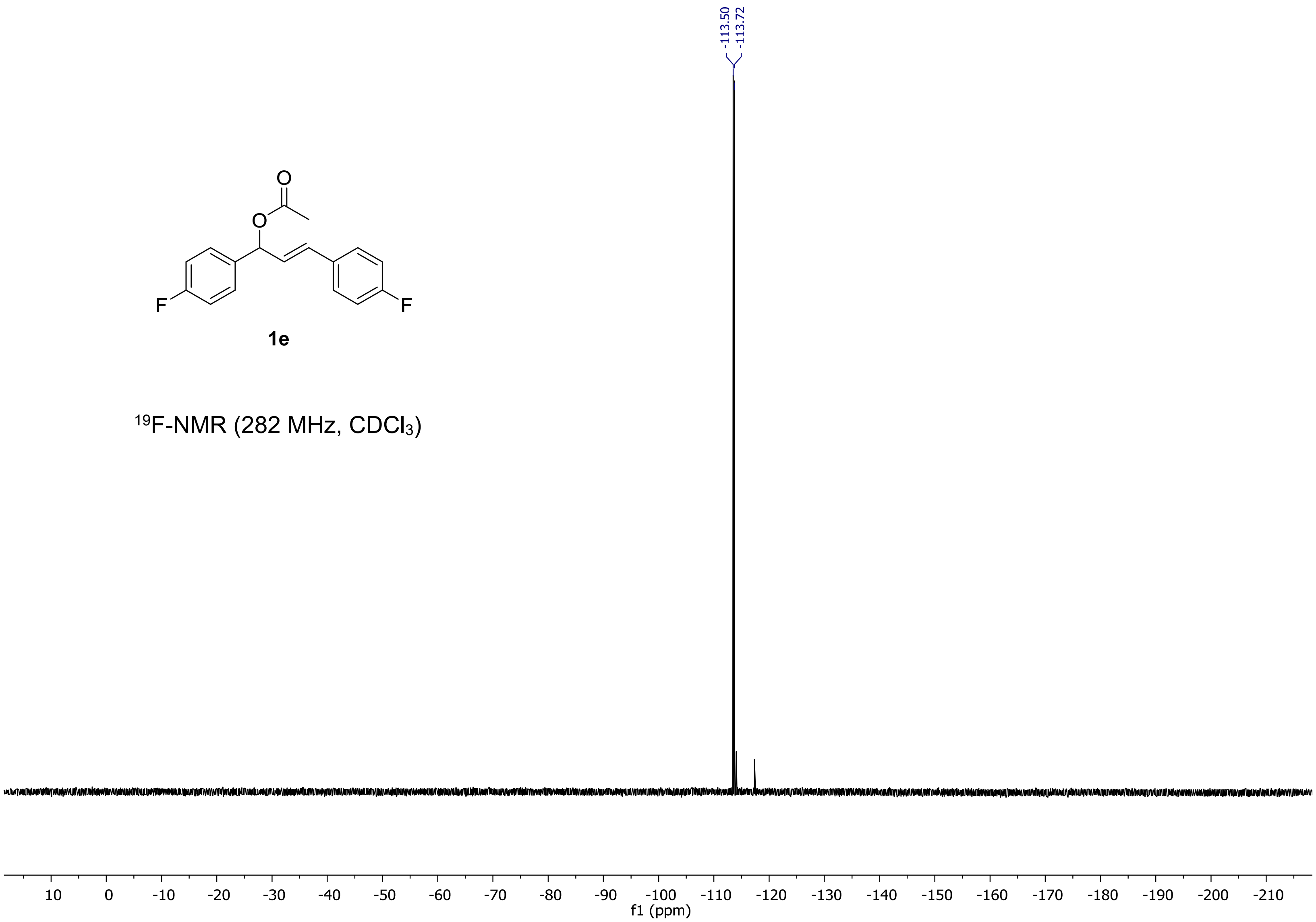

1e

^{19}F-NMR (282 MHz, $CDCl_3$)

Supplementary Figure 37. ^{19}F-NMR spectra for compound **1e**

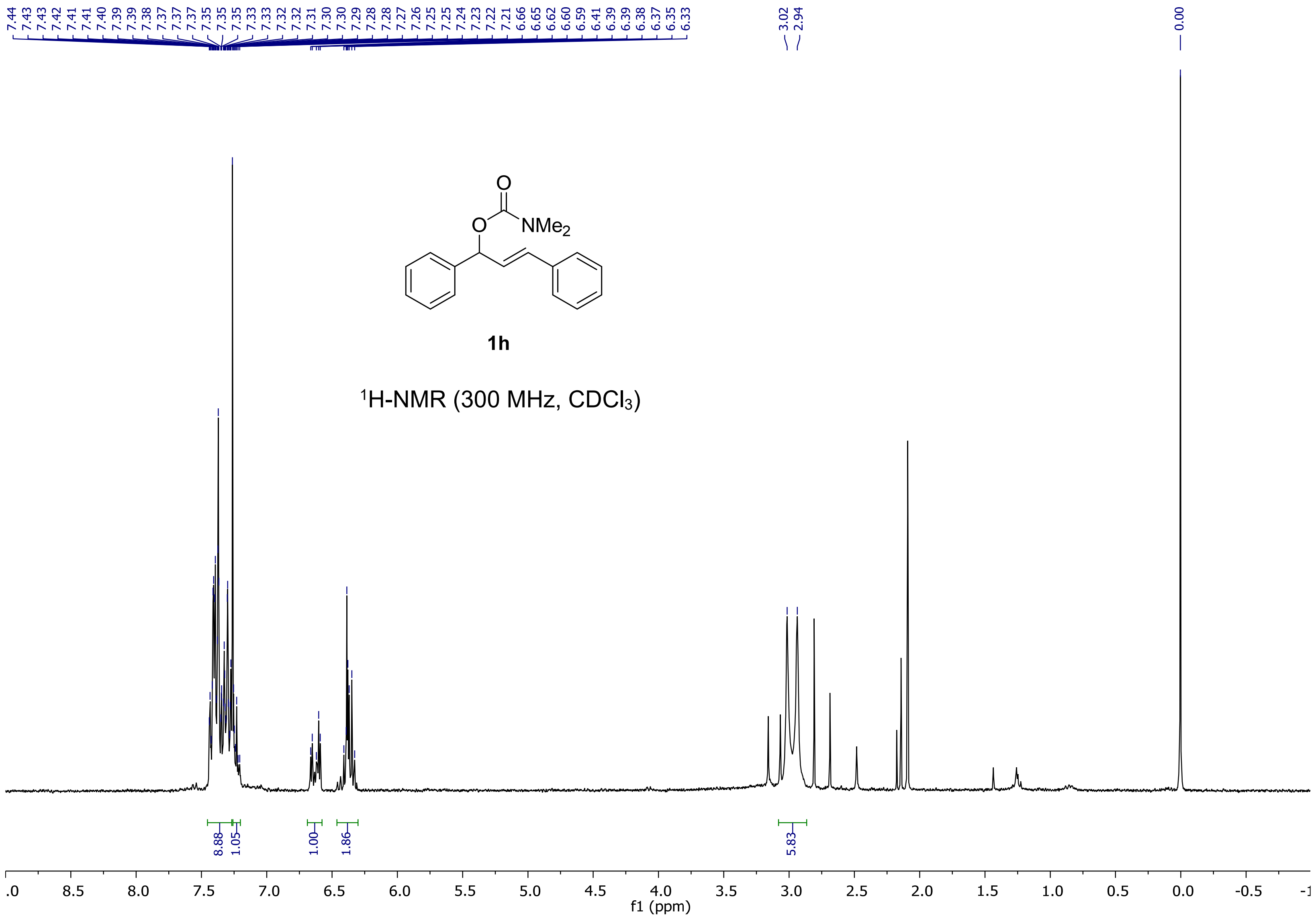

Supplementary Figure 38. ^{1}H-NMR spectra for compound **1h**

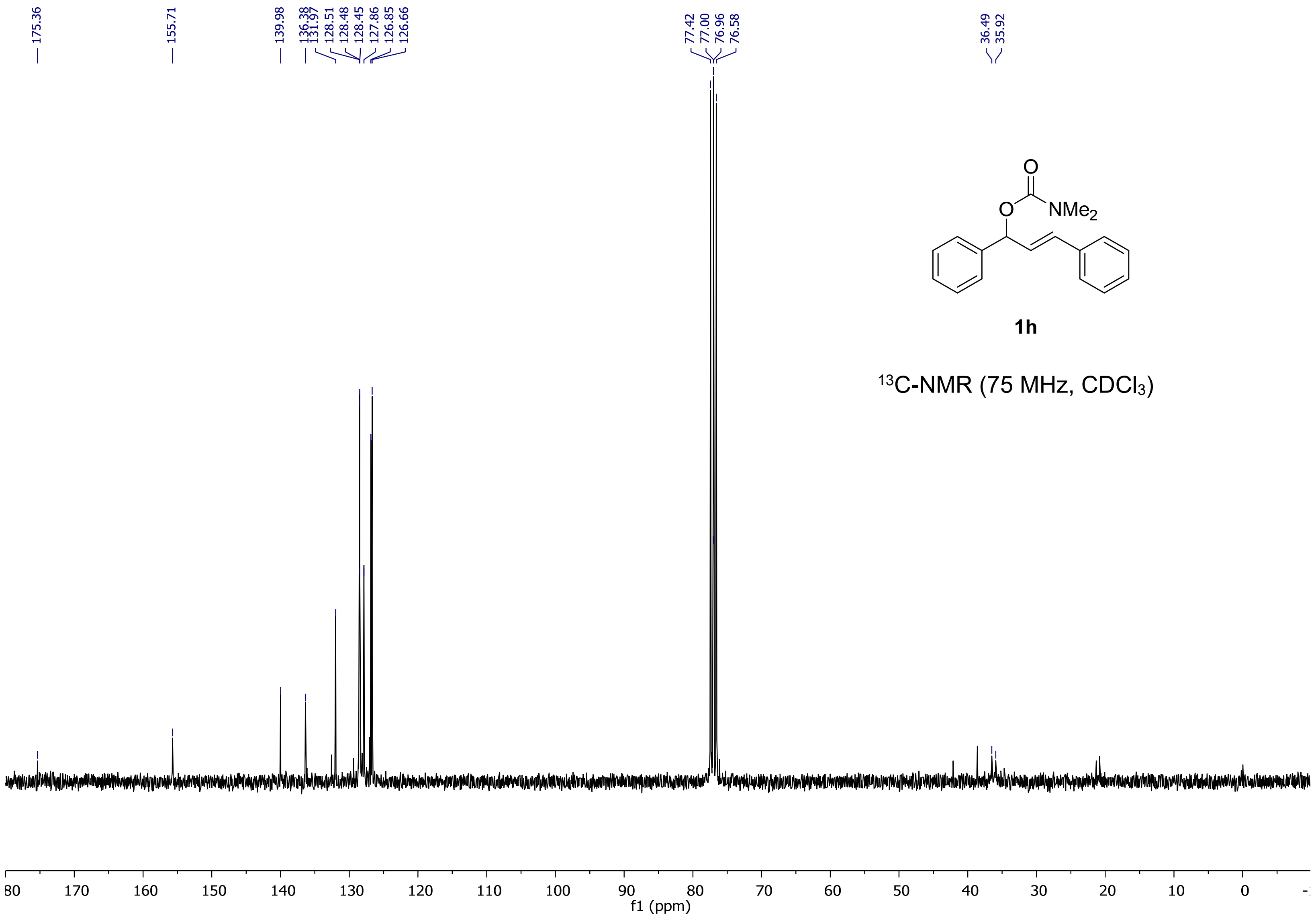

Supplementary Figure 39. ^{13}C-NMR spectra for compound **1h**

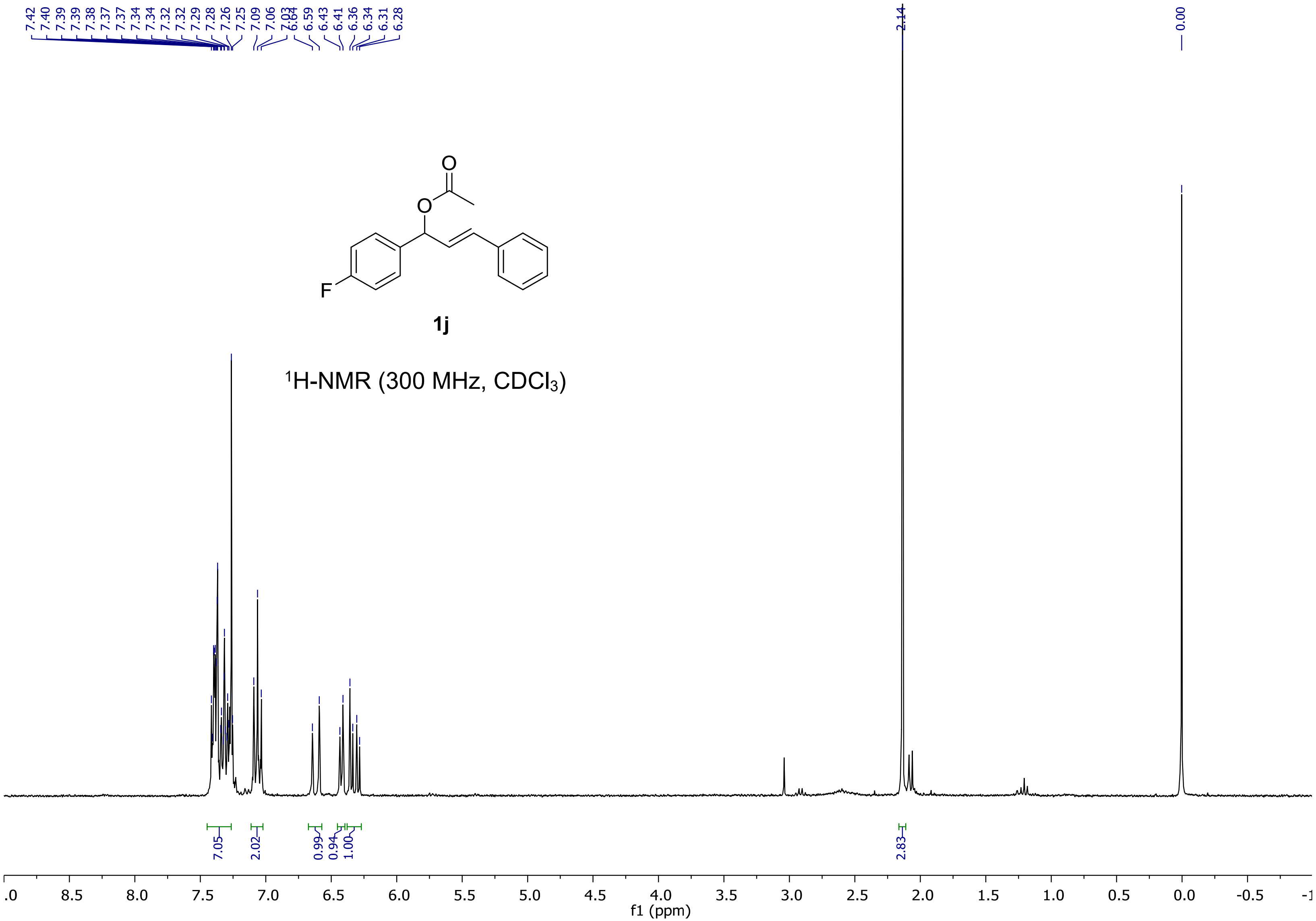

Supplementary Figure 40. ^{1}H-NMR spectra for compound **1j**

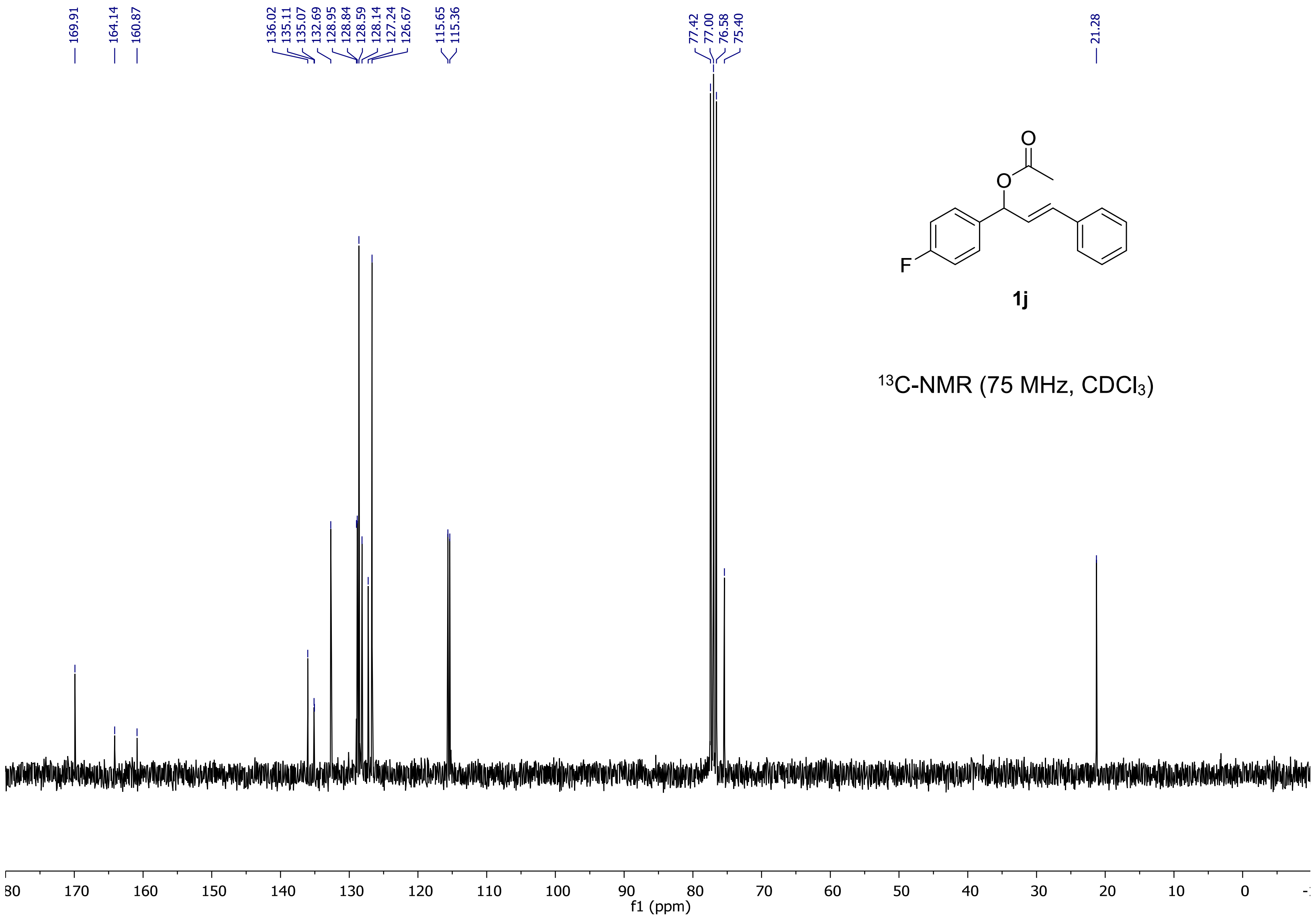

Supplementary Figure 41. ^{13}C-NMR spectra for compound **1j**

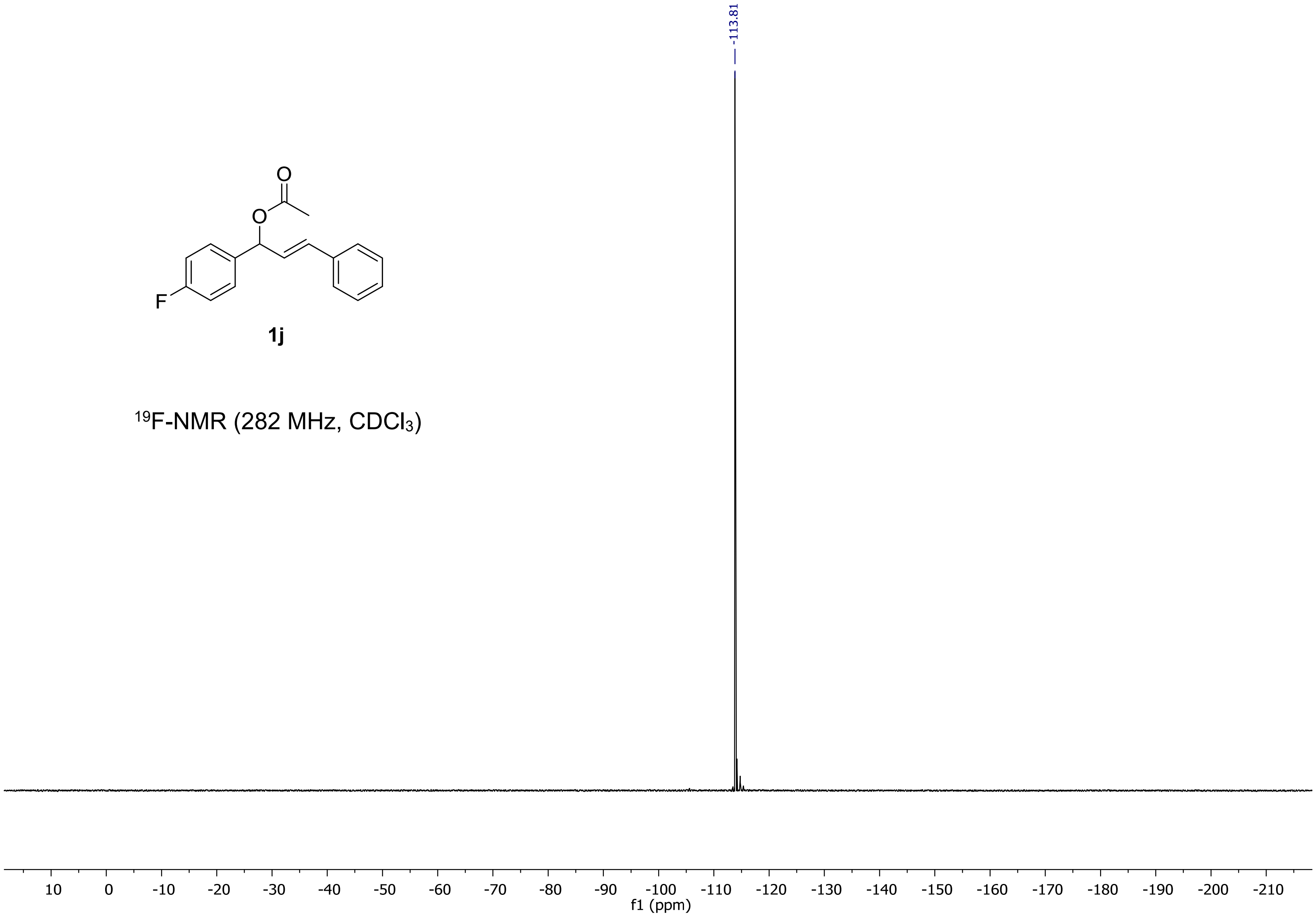

Supplementary Figure 42. ^{19}F-NMR spectra for compound **1j**

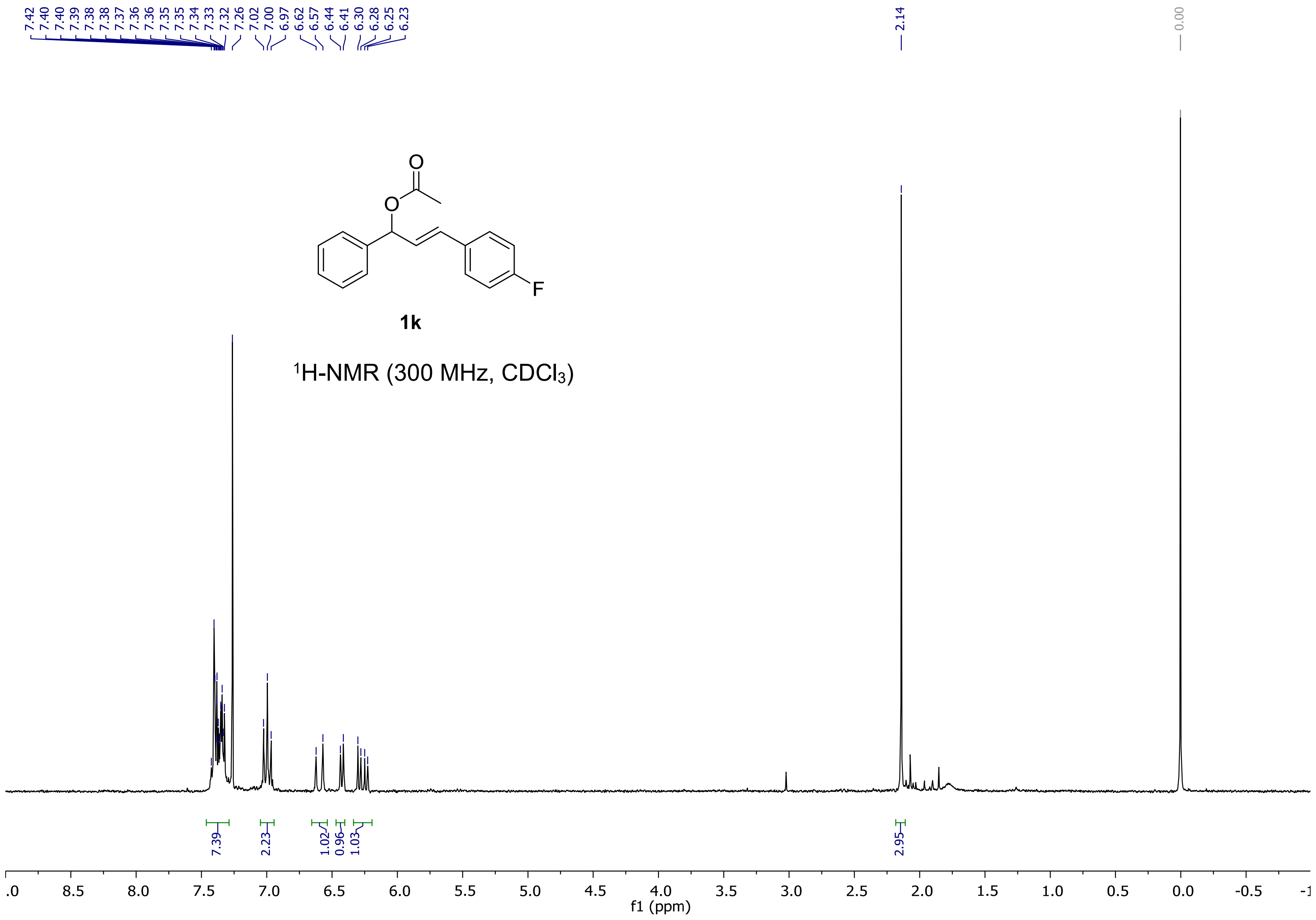

Supplementary Figure 43. ^{1}H-NMR spectra for compound **1k**

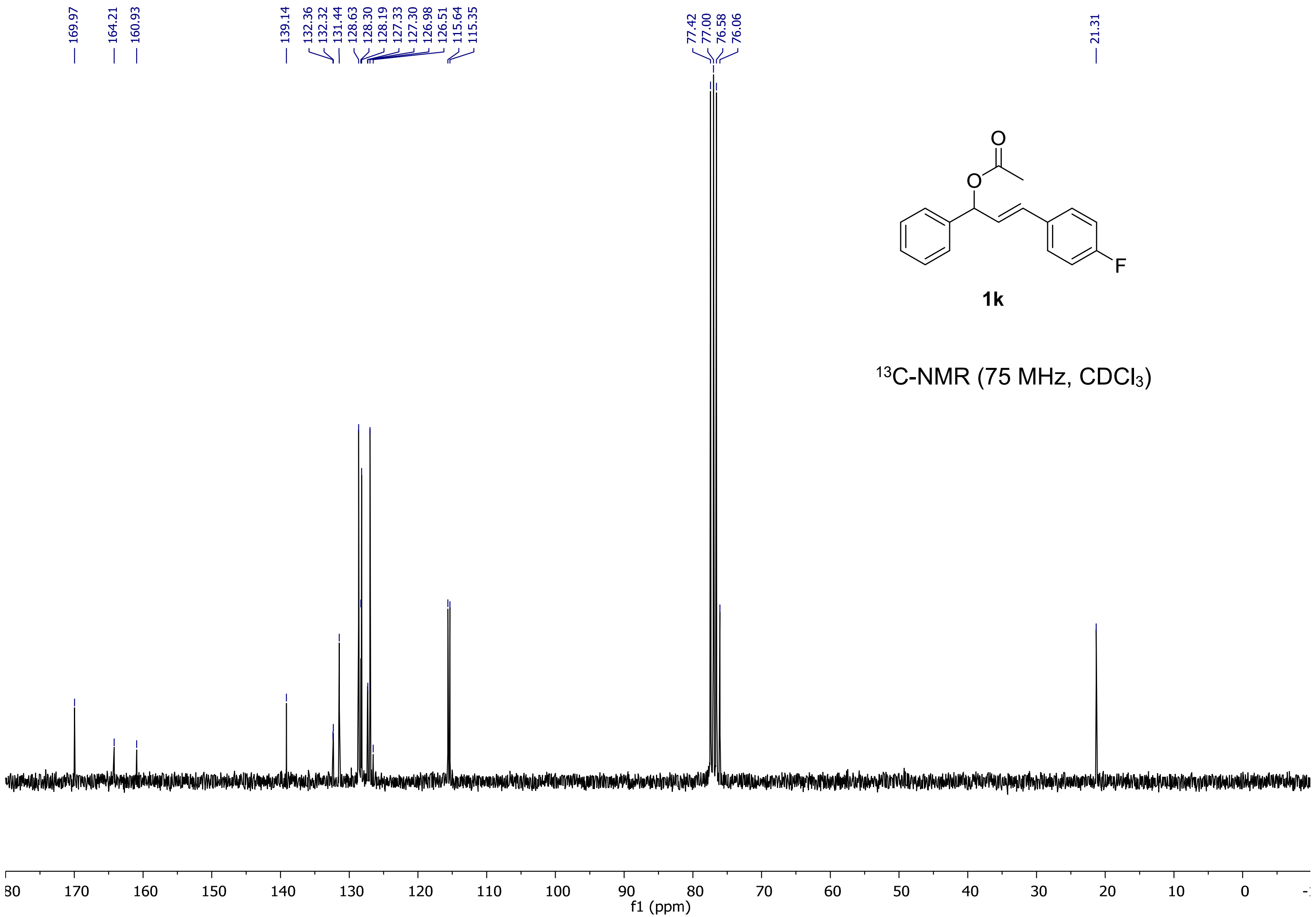

Supplementary Figure 44. ^{13}C-NMR spectra for compound **1k**

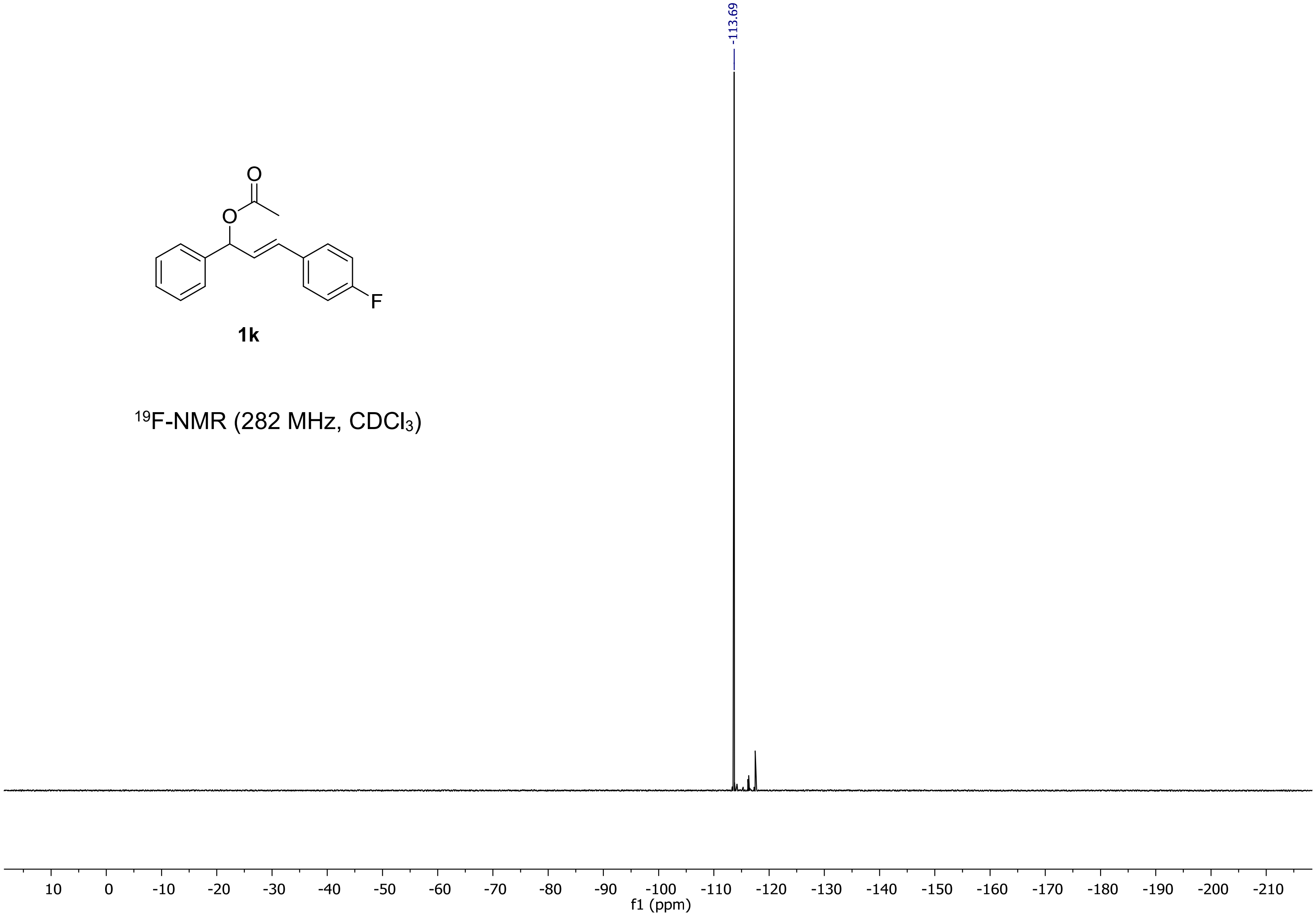

Supplementary Figure 45. ^{19}F-NMR spectra for compound **1k**

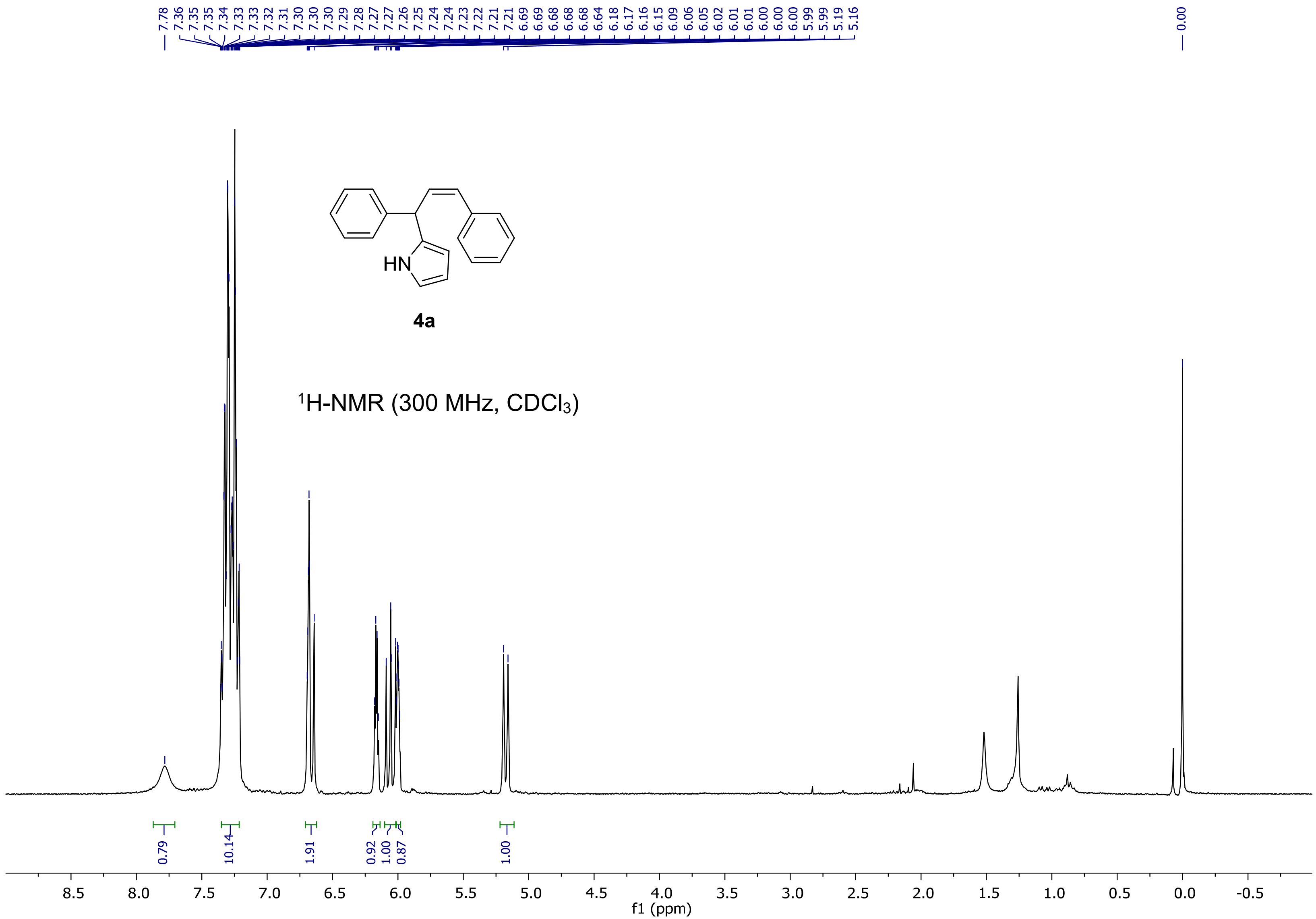

Supplementary Figure 46. ^{1}H-NMR spectra for compound **4a**

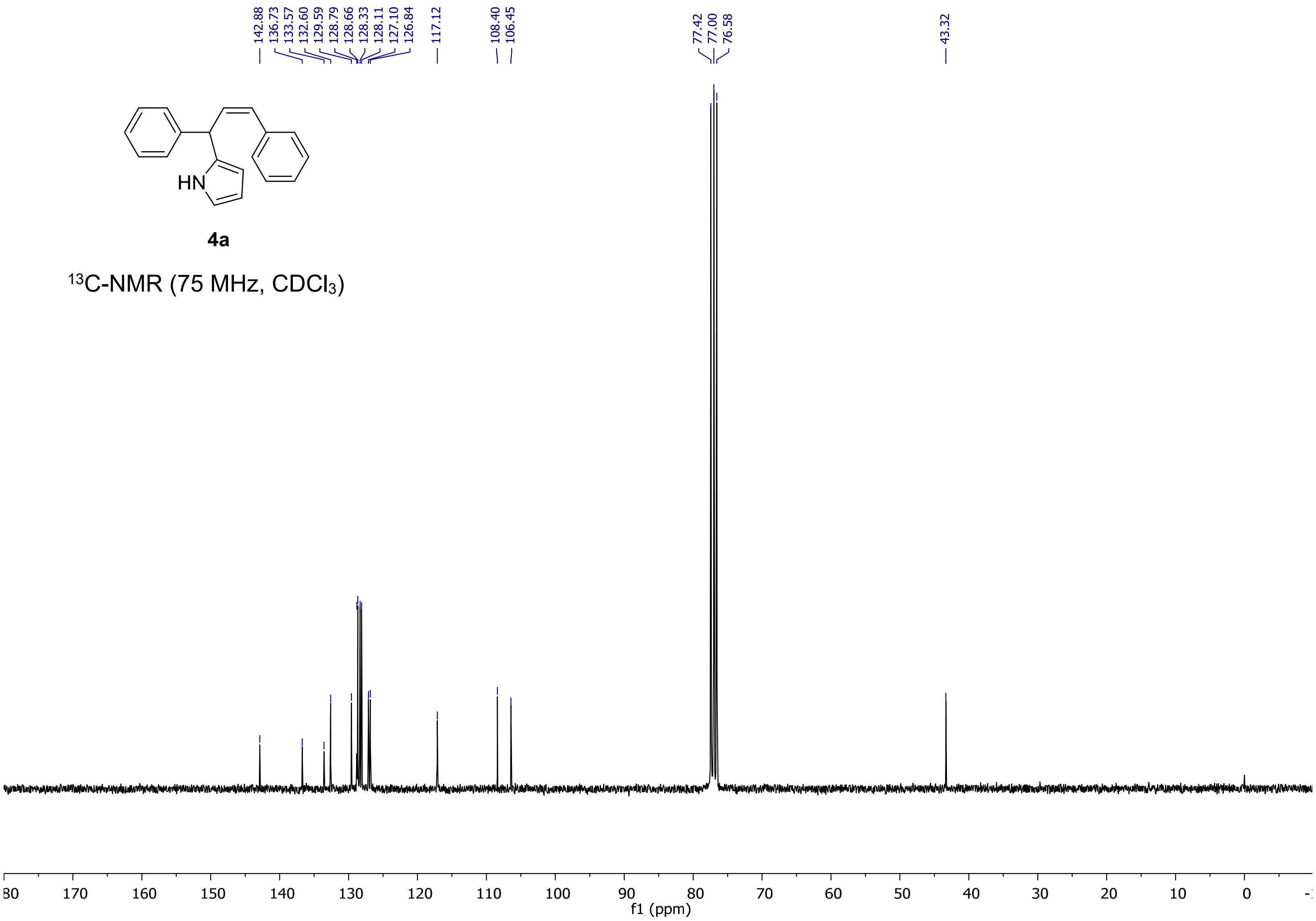

Supplementary Figure 47. ^{13}C-NMR spectra for compound **4a**

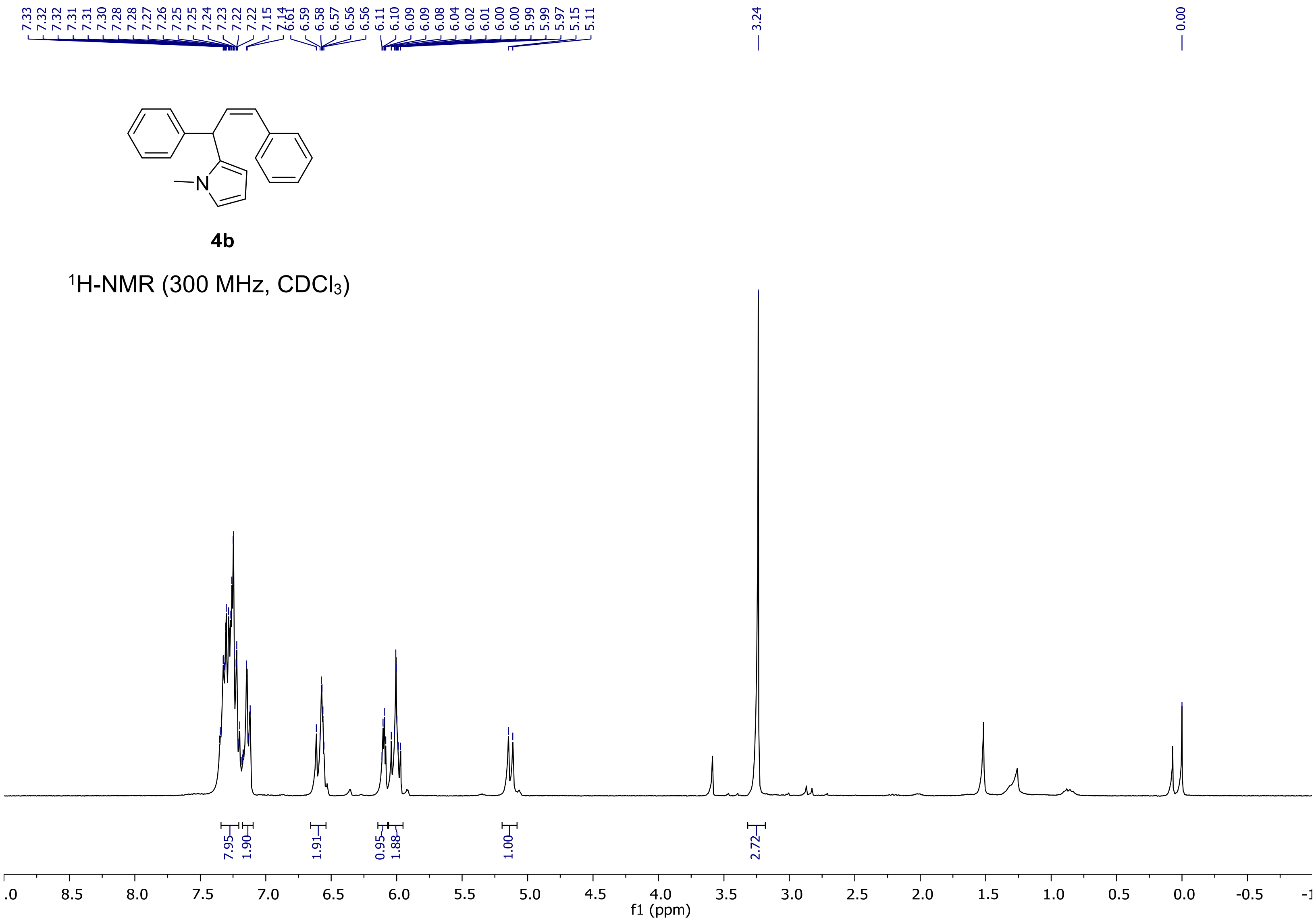

Supplementary Figure 48. ^{1}H-NMR spectra for compound **4b**

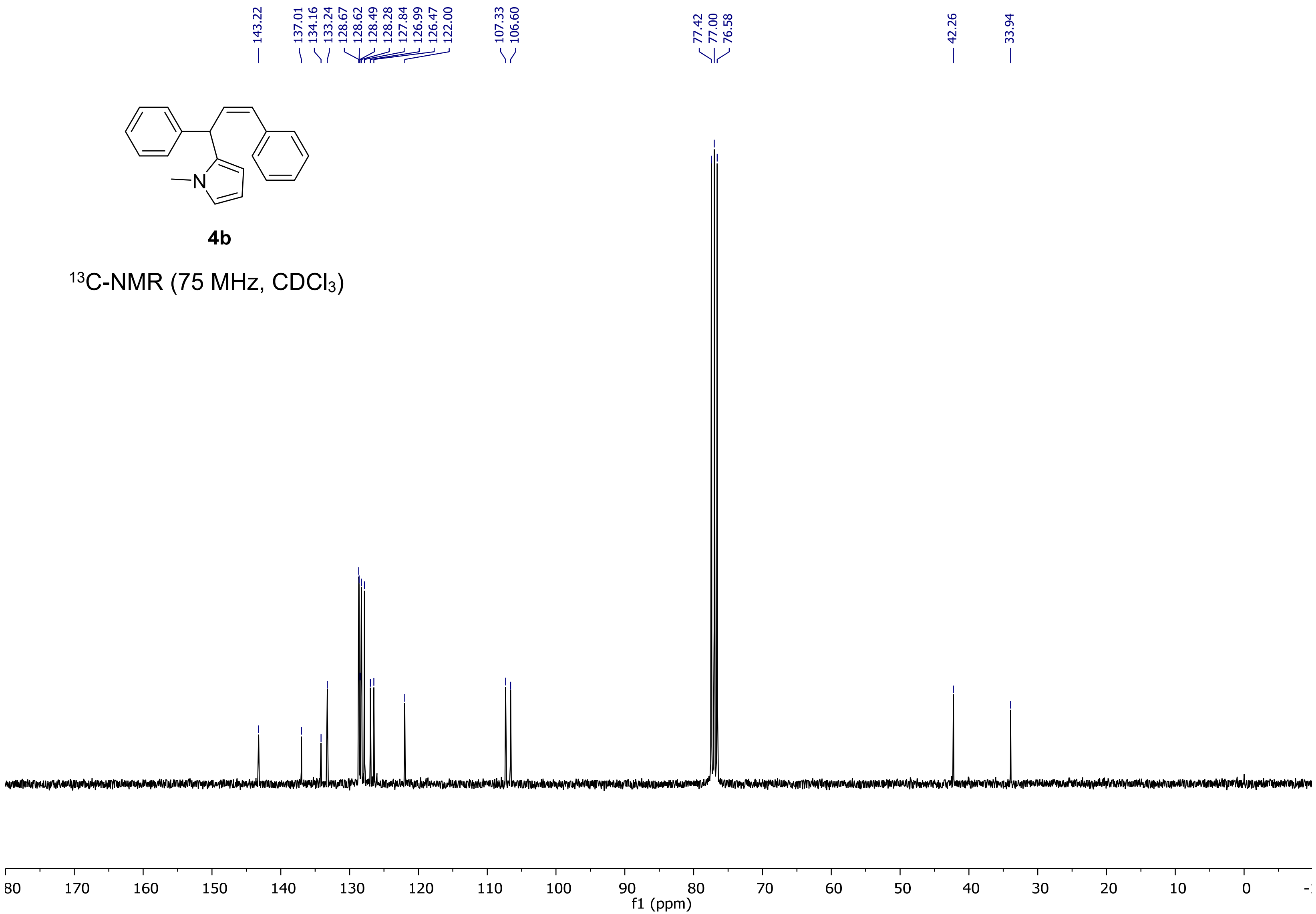

Supplementary Figure 49. ^{13}C-NMR spectra for compound **4b**

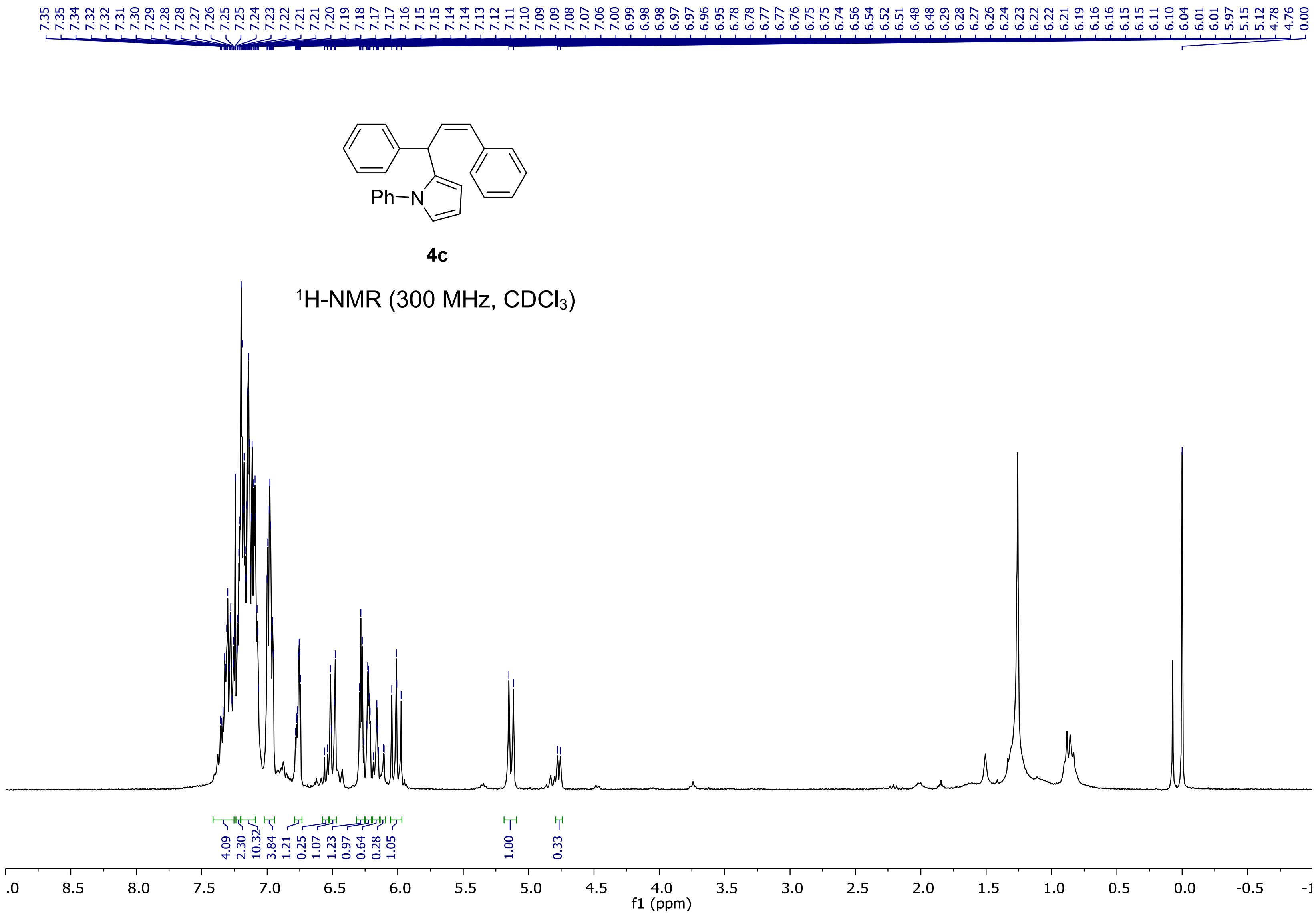

Supplementary Figure 50. ^{1}H-NMR spectra for compound **4c**

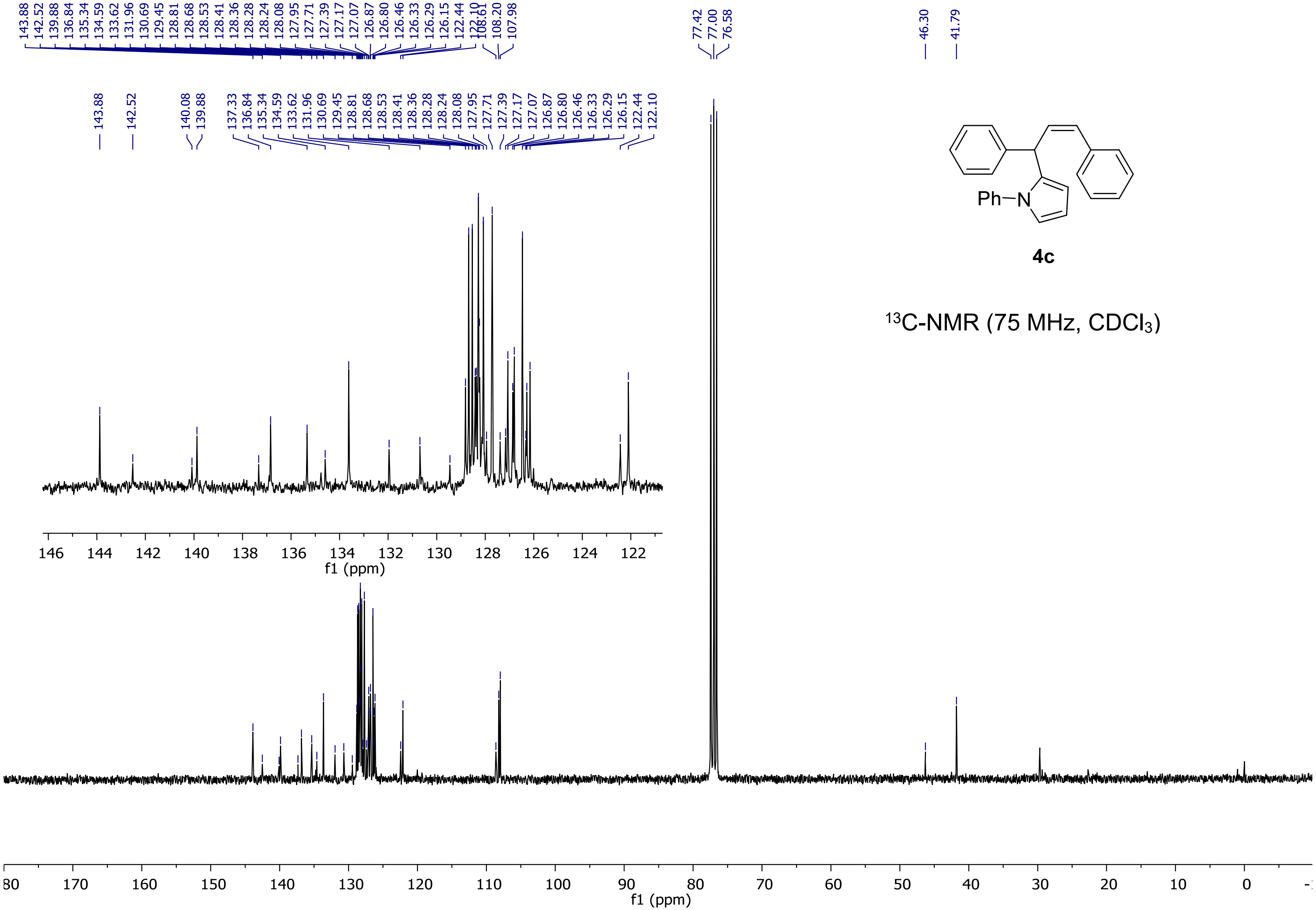

Supplementary Figure 51. ^{13}C-NMR spectra for compound **4c**

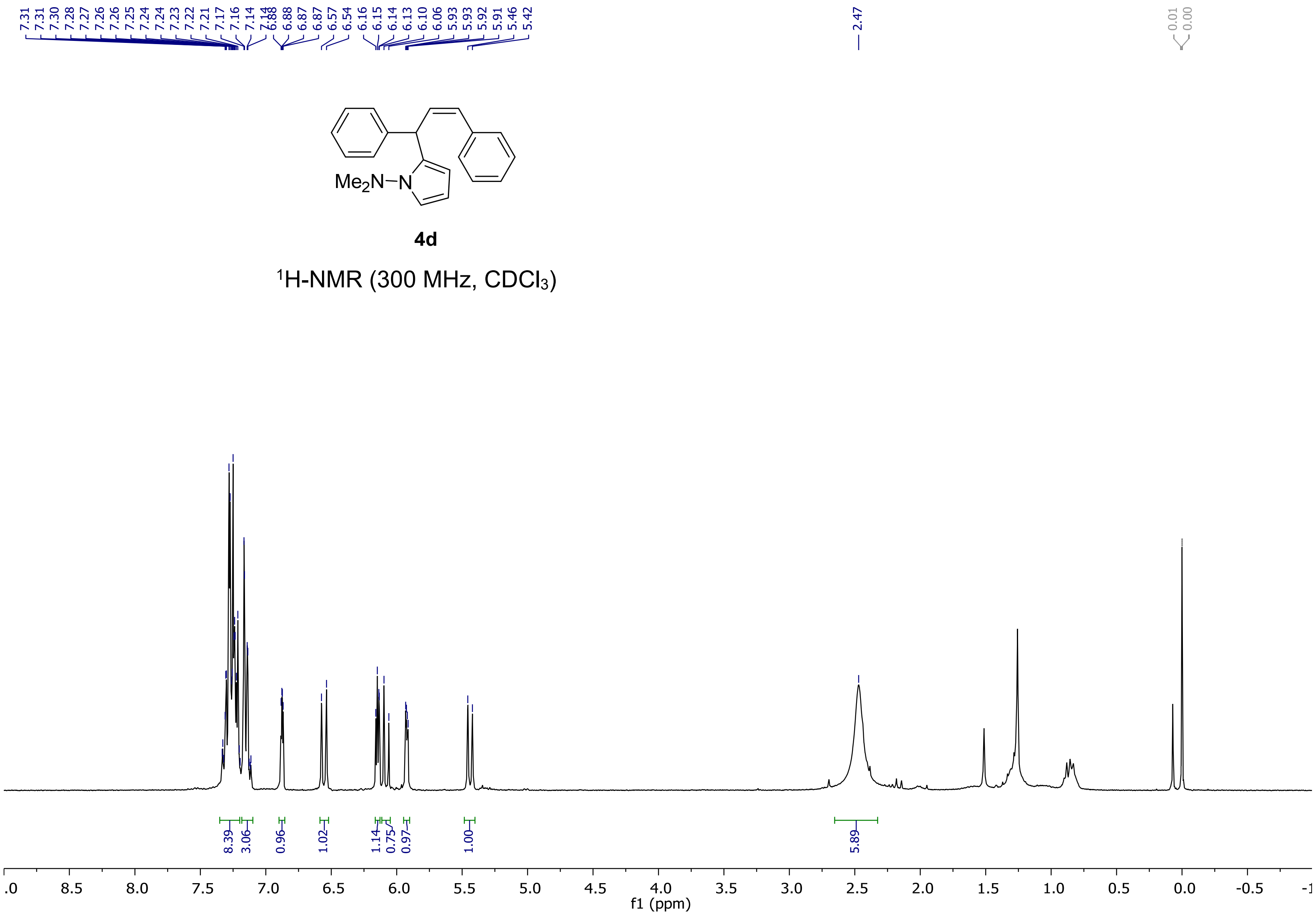

Supplementary Figure 52. ^{1}H-NMR spectra for compound **4d**

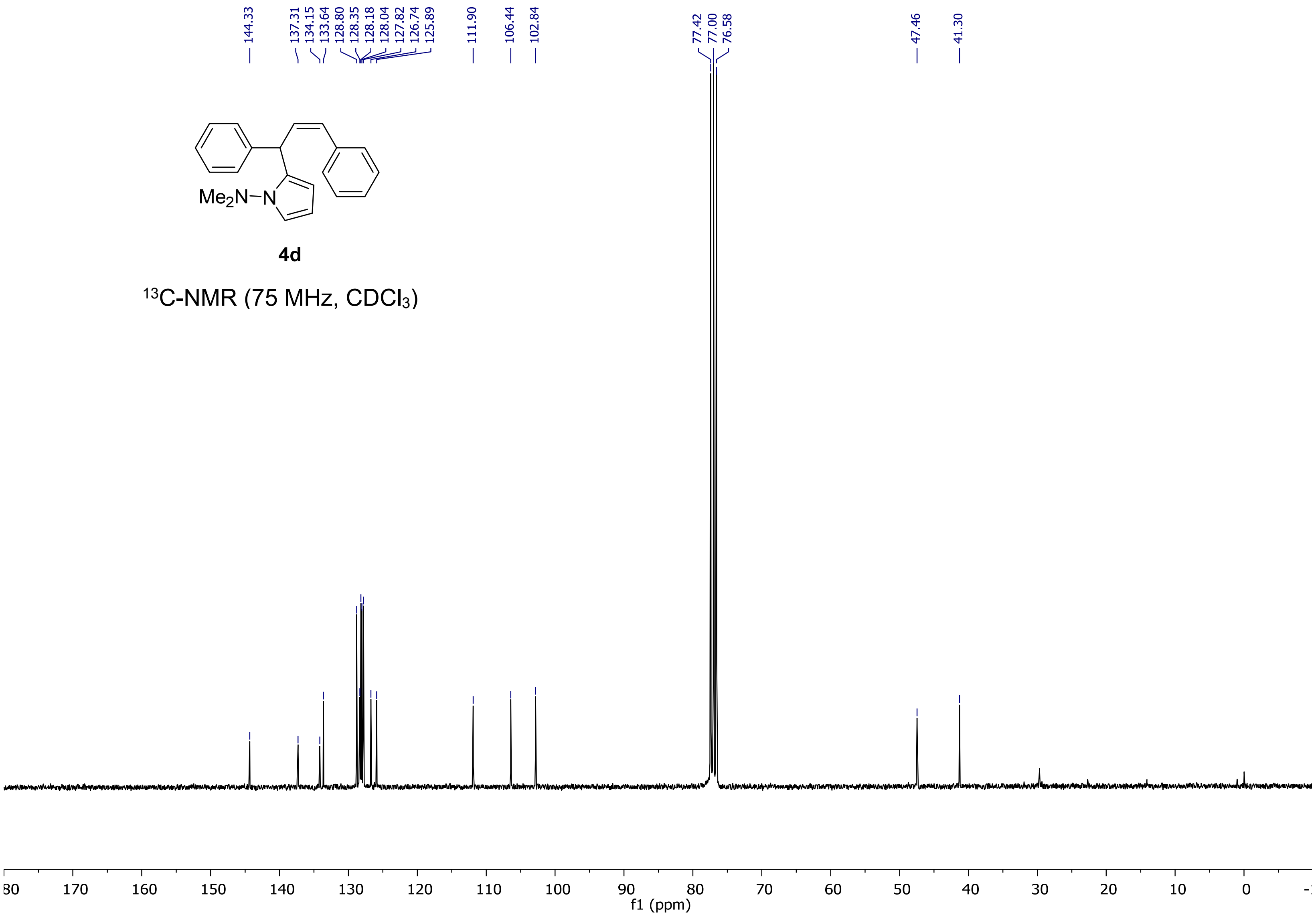

Supplementary Figure 53. ^{13}C-NMR spectra for compound **4d**

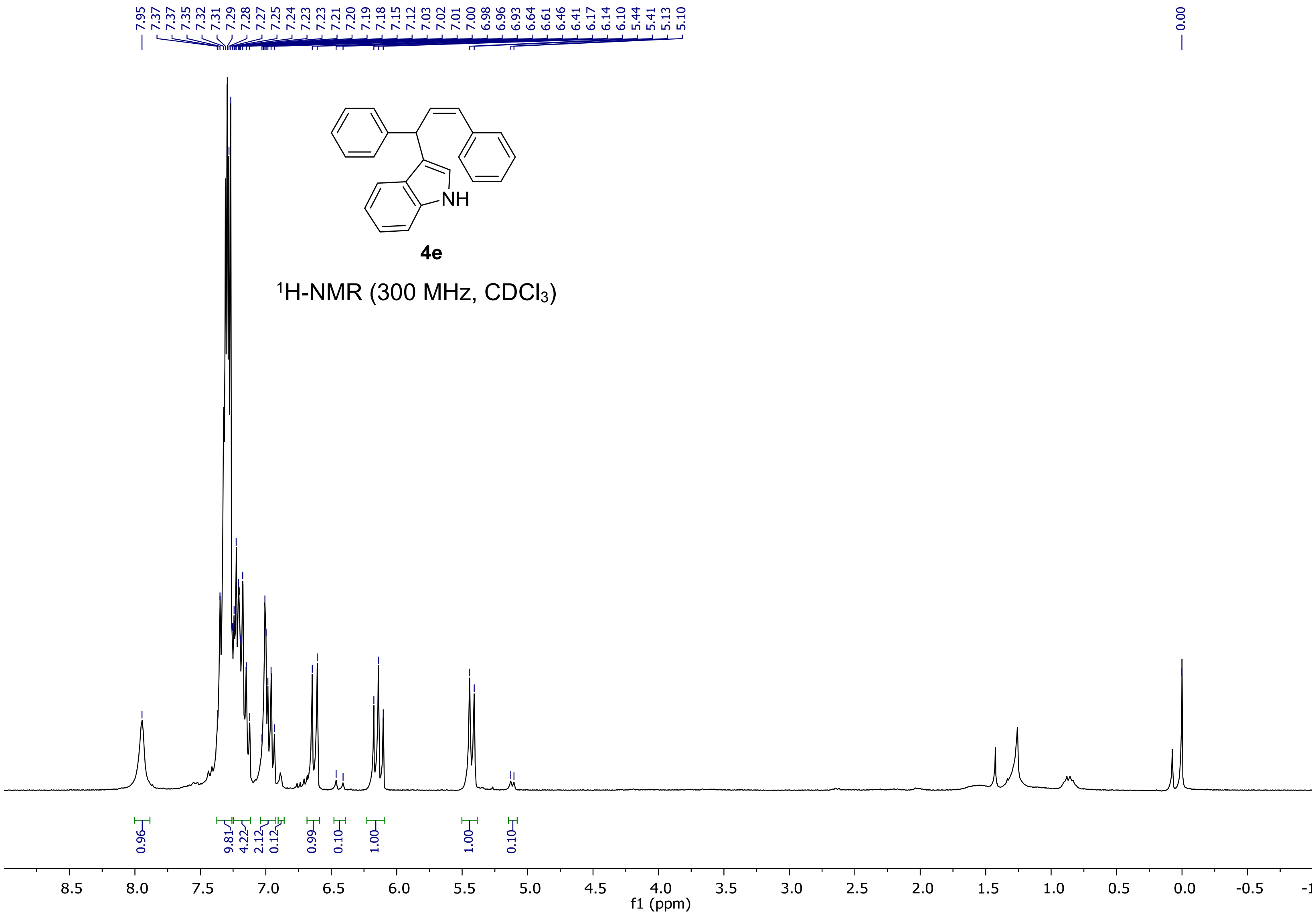

Supplementary Figure 54. ^{1}H-NMR spectra for compound **4e**

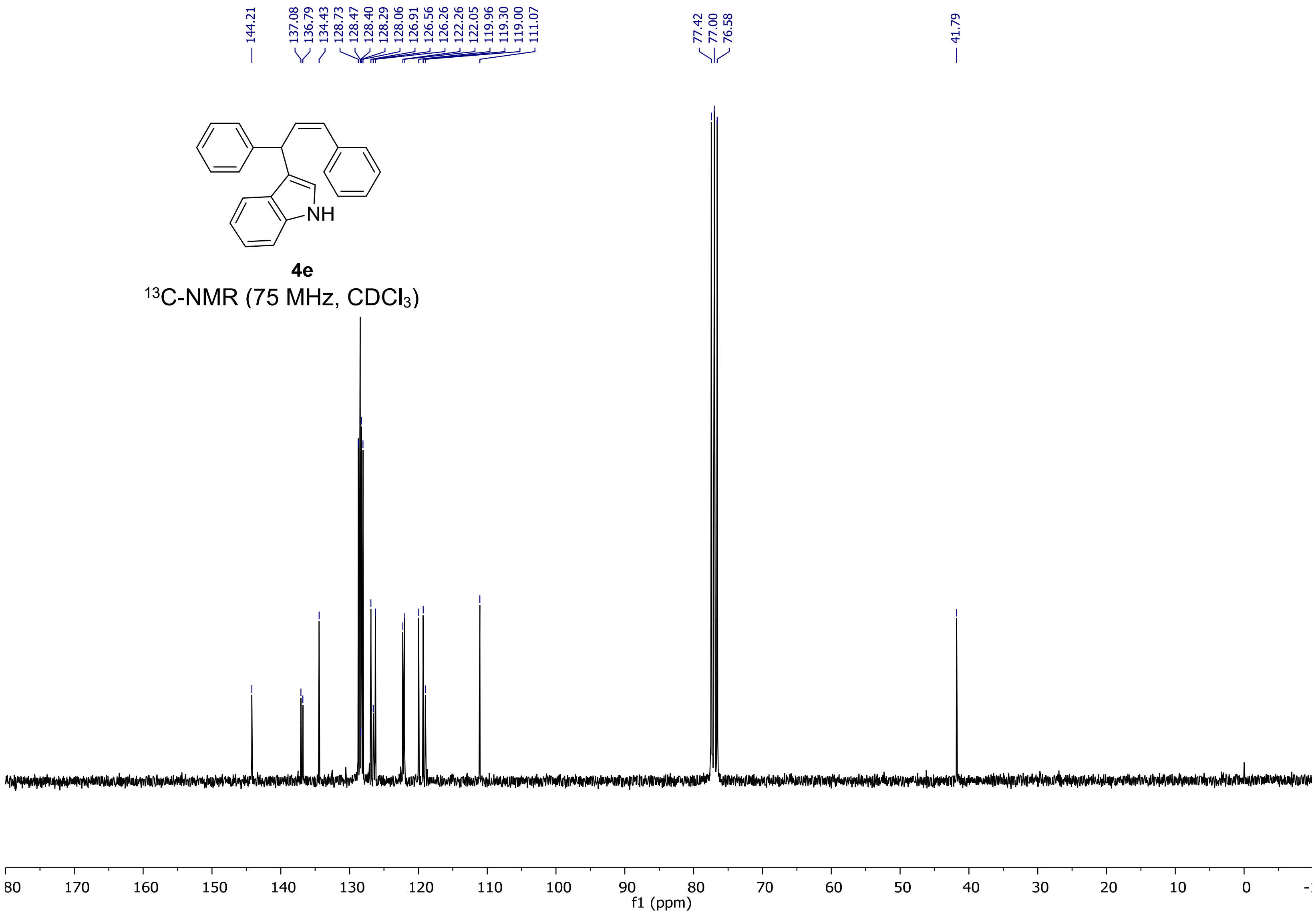

Supplementary Figure 55. ^{13}C-NMR spectra for compound **4e**

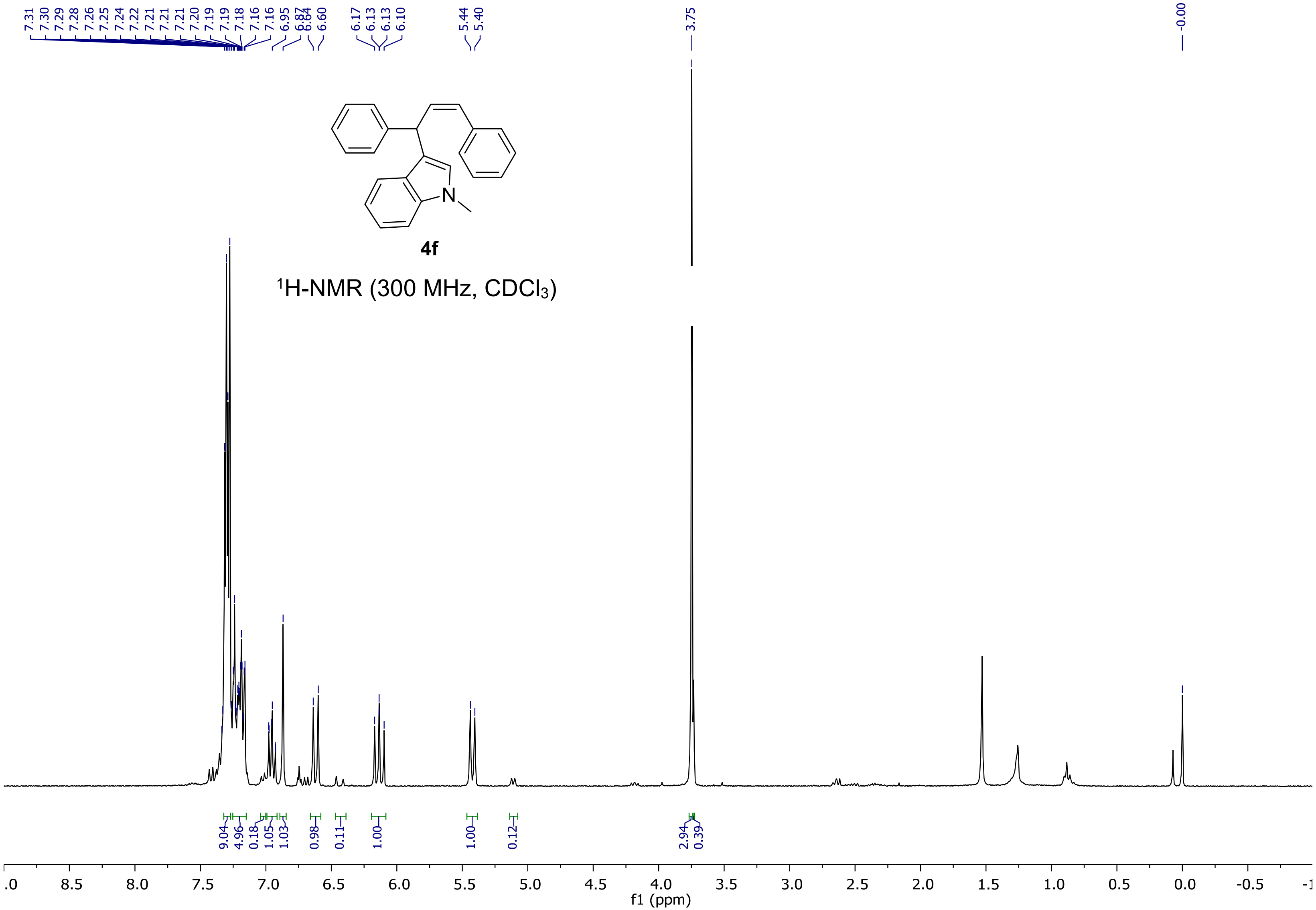

Supplementary Figure 56. ^{1}H-NMR spectra for compound **4f**

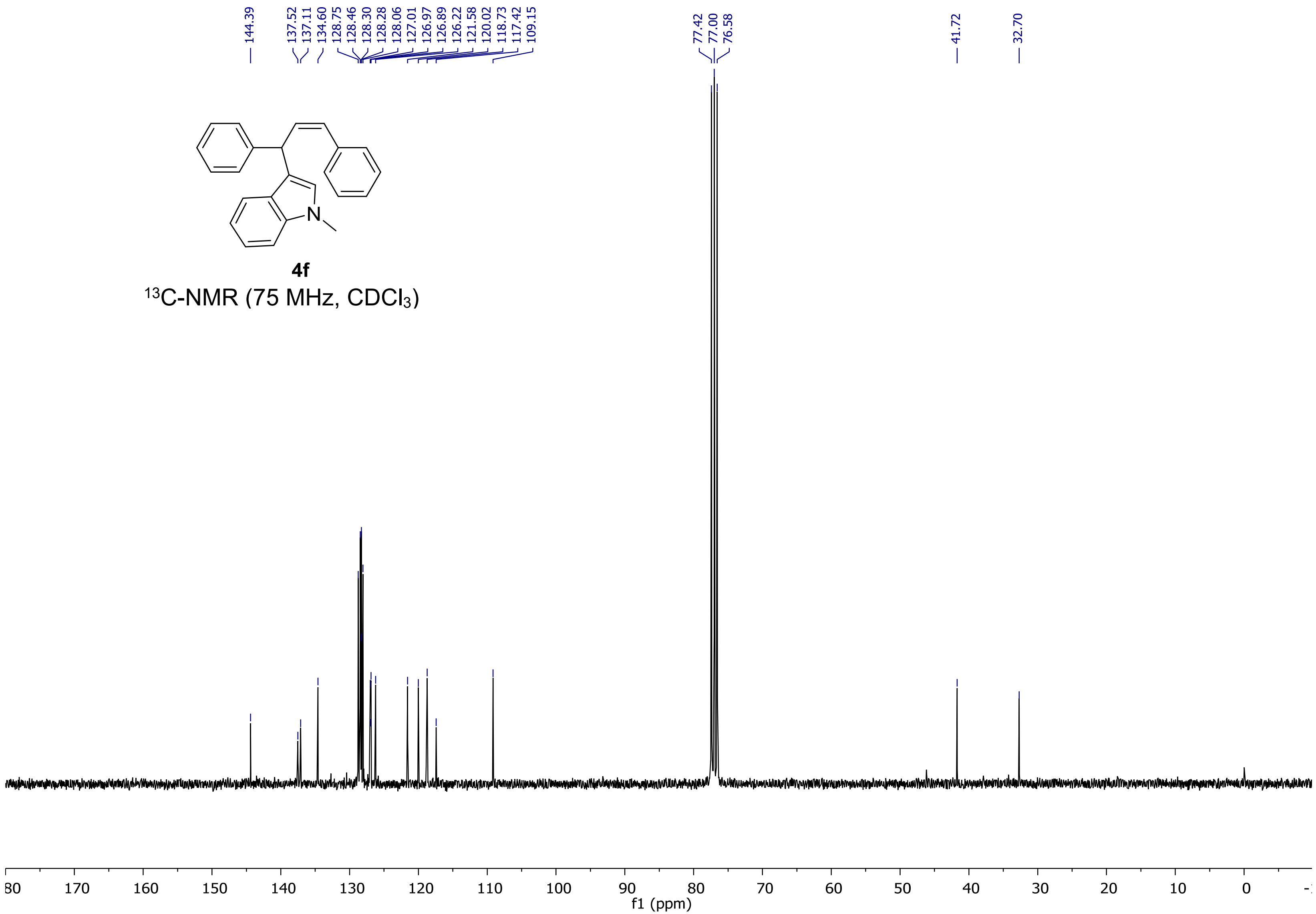

Supplementary Figure 57. ^{13}C-NMR spectra for compound **4f**

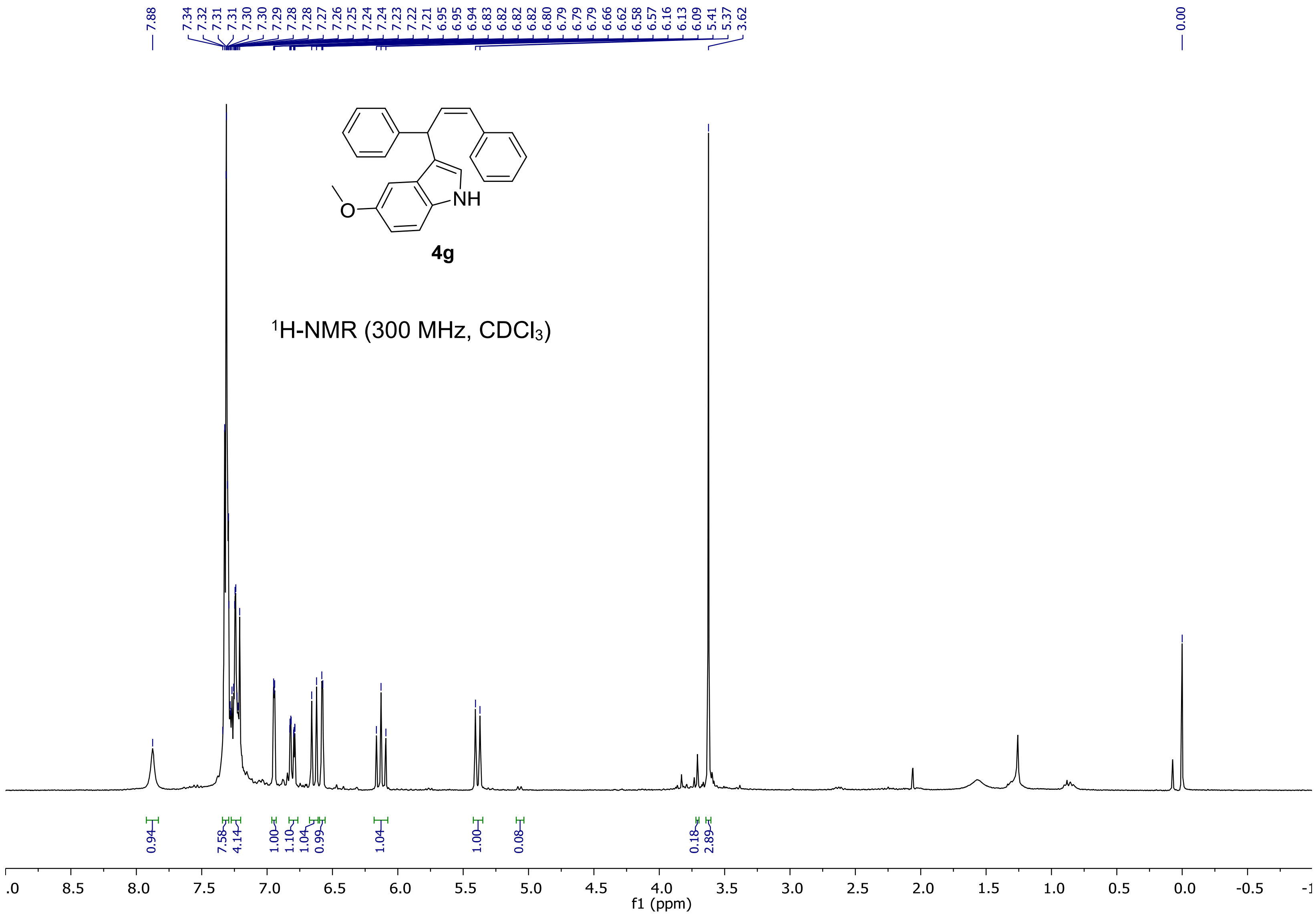

Supplementary Figure 58. ^{1}H-NMR spectra for compound **4g**

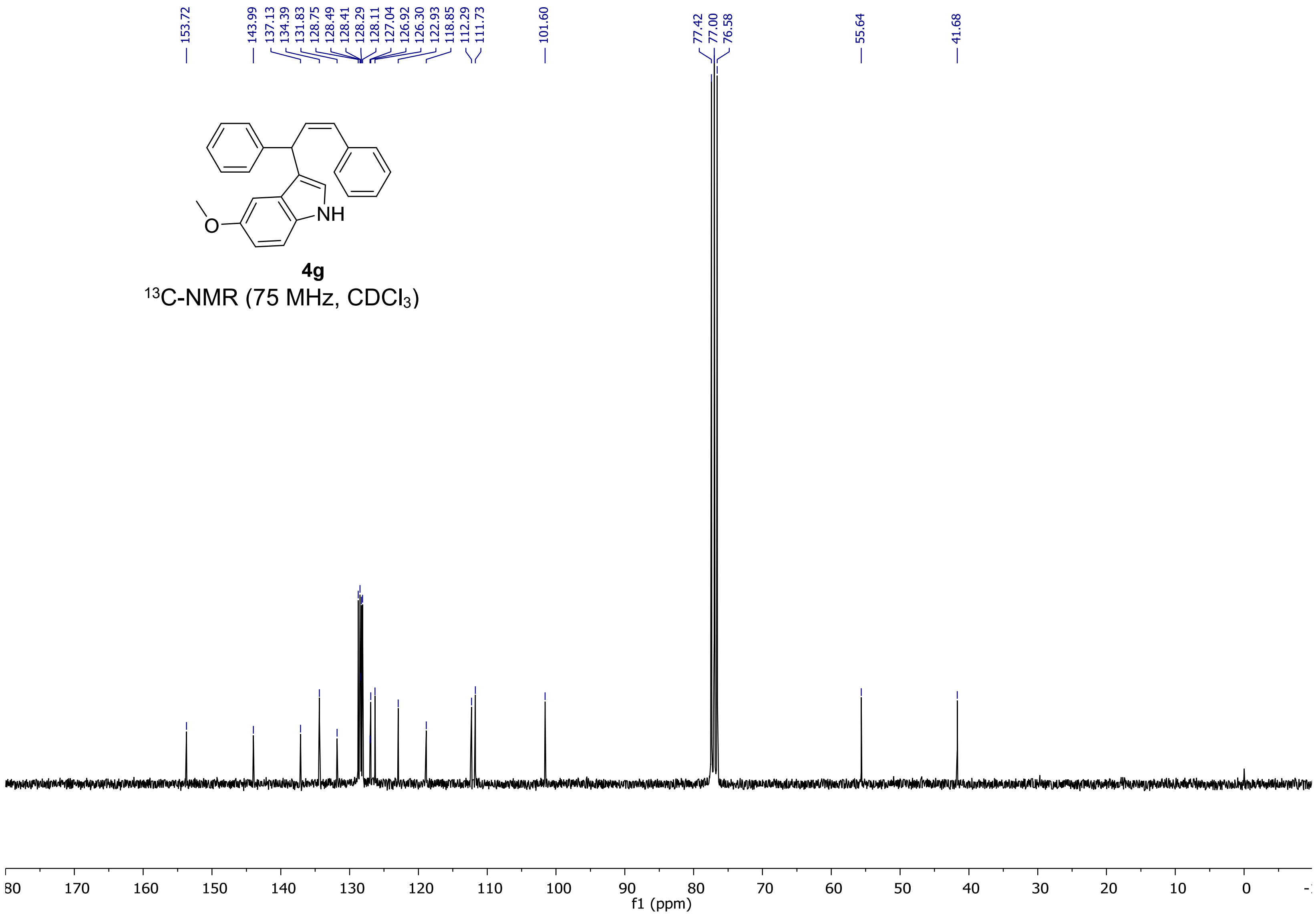

Supplementary Figure 59. ^{13}C-NMR spectra for compound **4g**

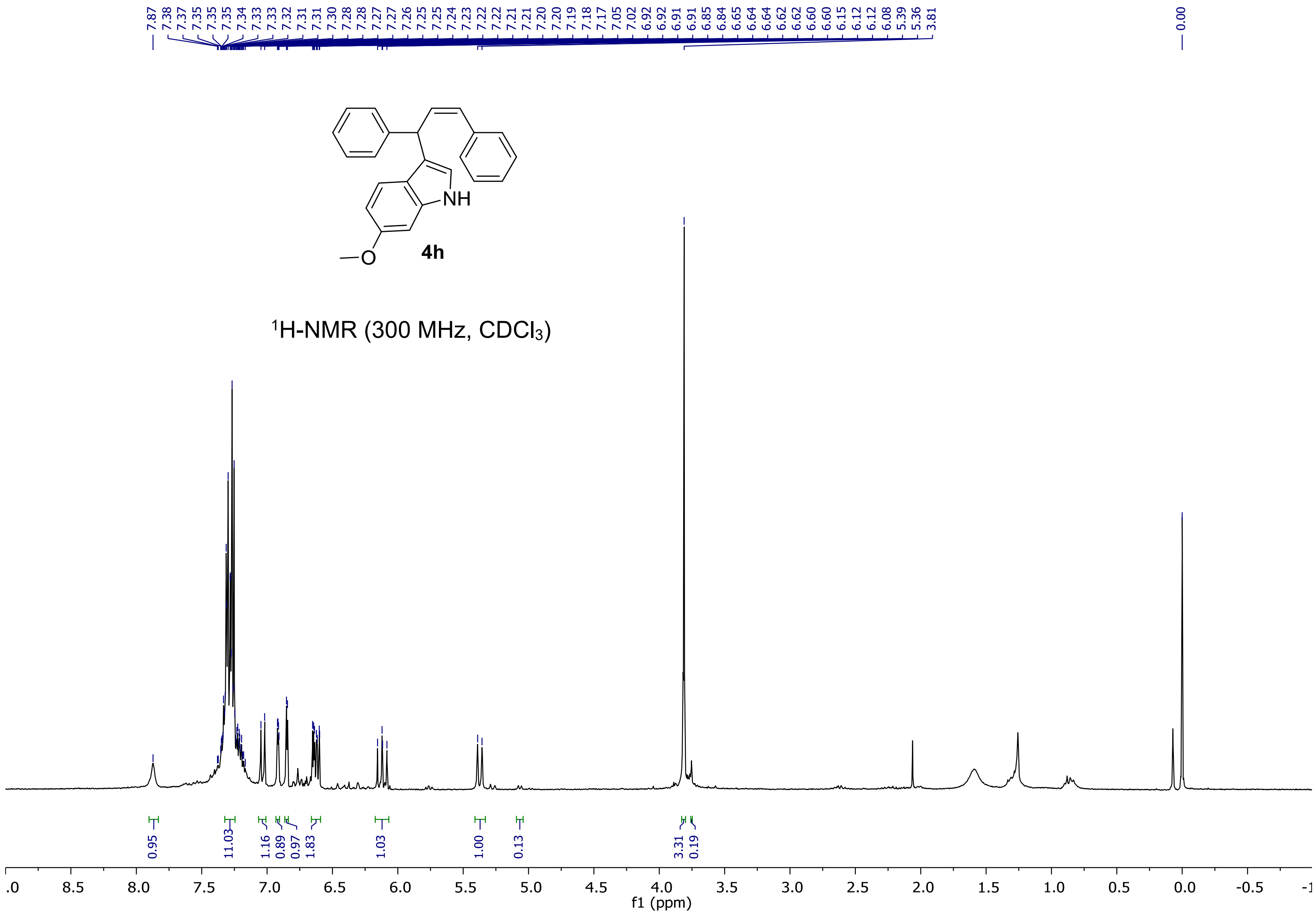

Supplementary Figure 60. ^{1}H-NMR spectra for compound **4h**

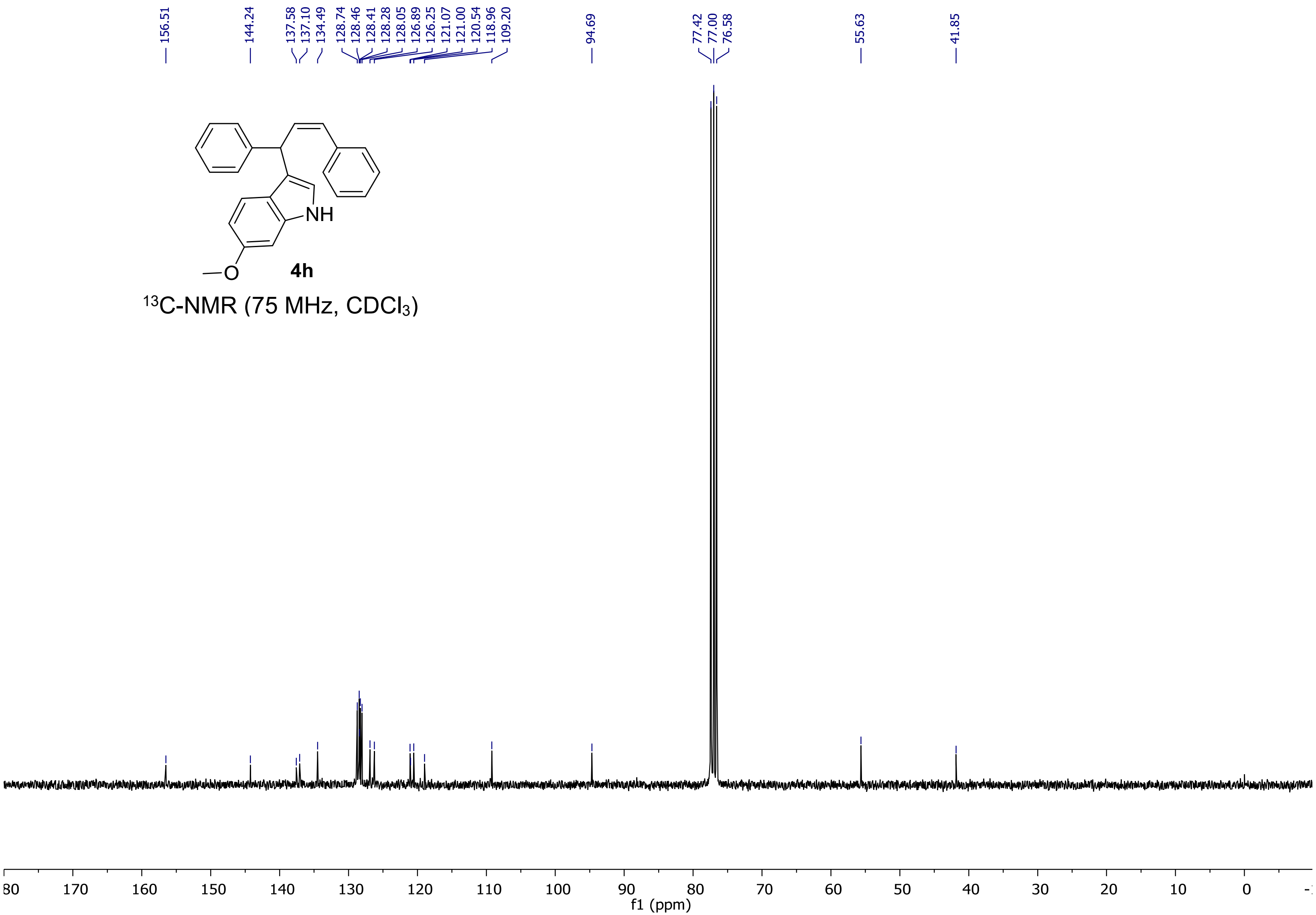

Supplementary Figure 61. ^{13}C-NMR spectra for compound **4h**

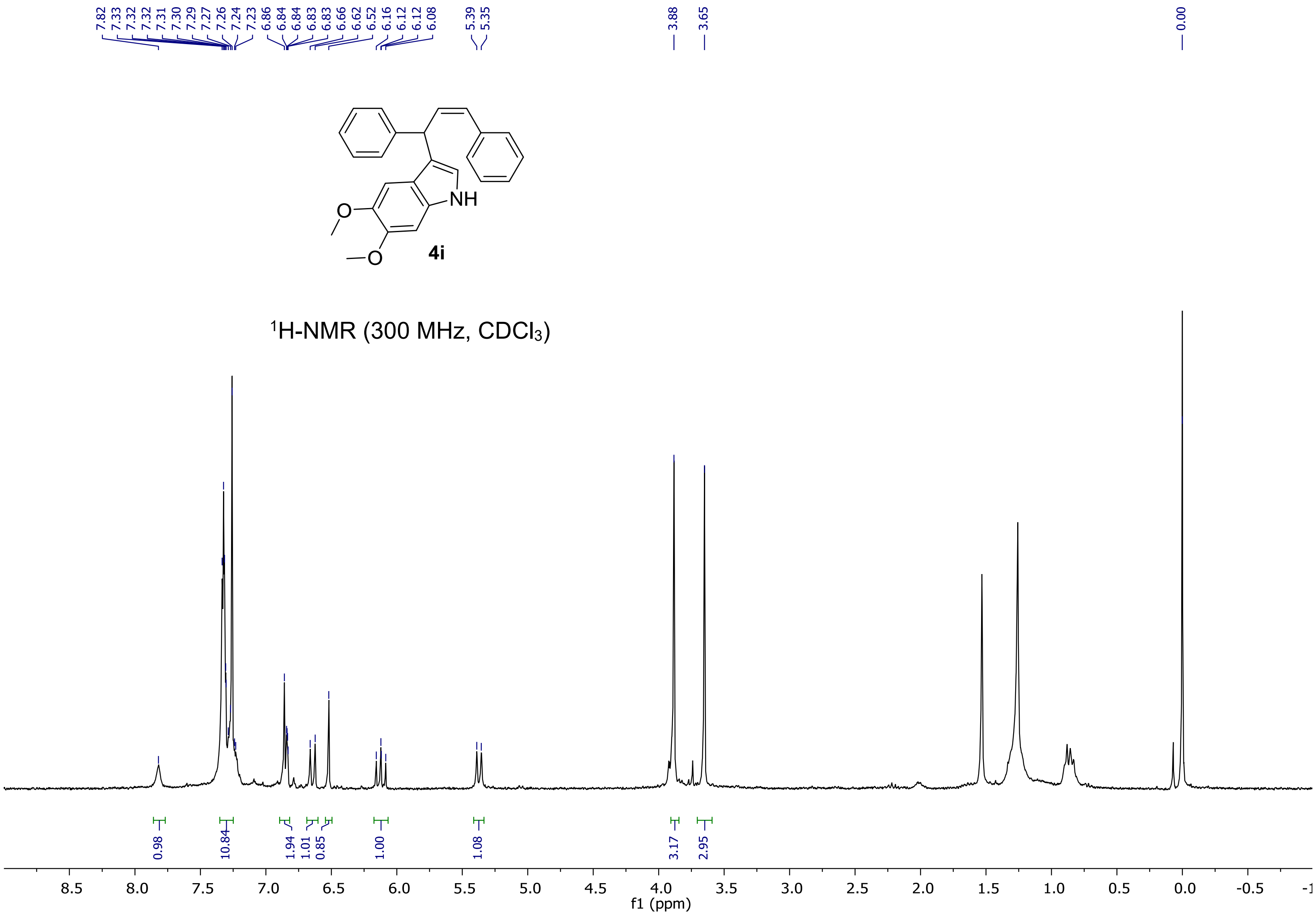

Supplementary Figure 62. ^{1}H-NMR spectra for compound **4i**

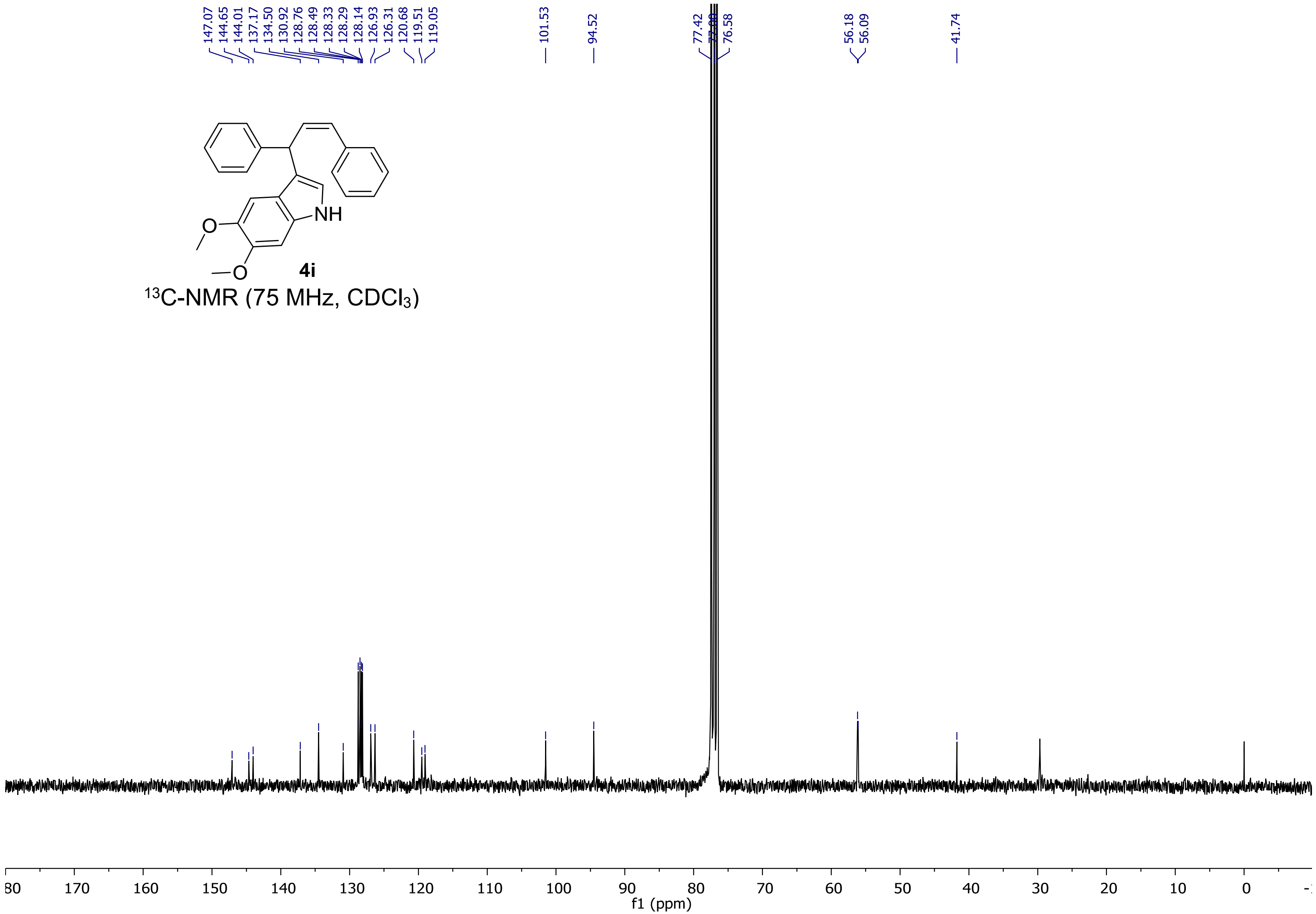

Supplementary Figure 63. ^{13}C-NMR spectra for compound **4i**

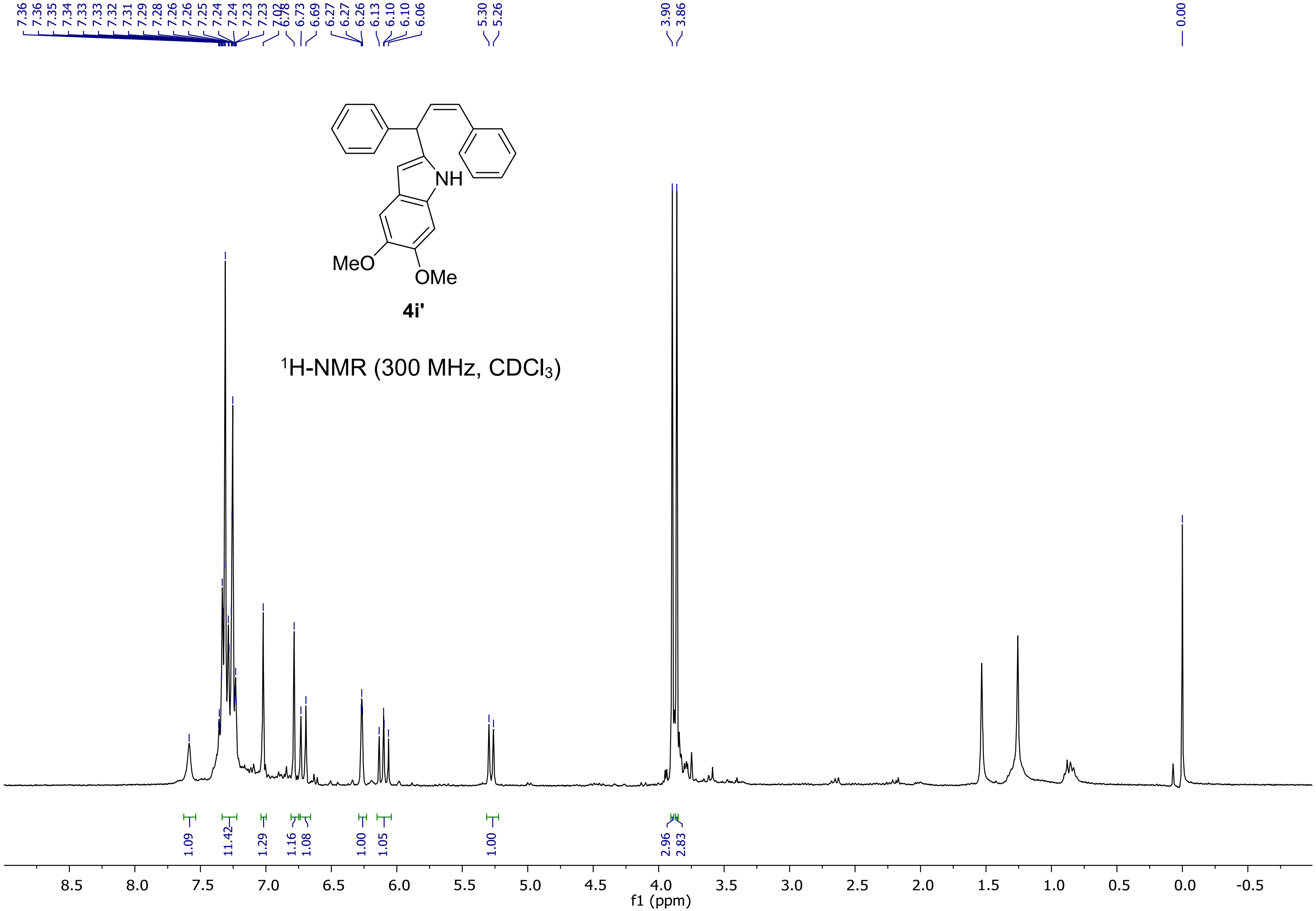

Supplementary Figure 64. ^{1}H-NMR spectra for compound **4i’**

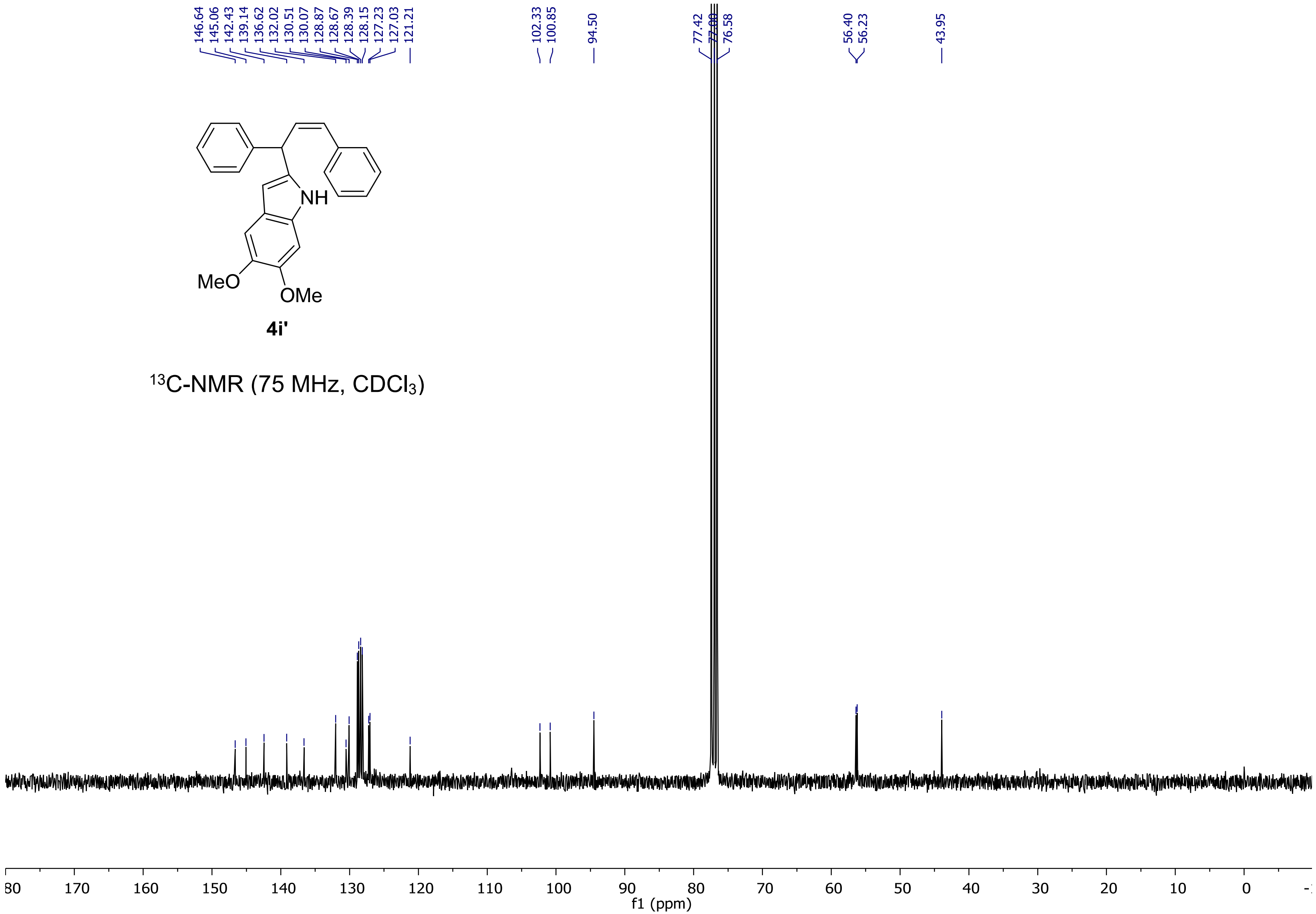

Supplementary Figure 65. ^{13}C-NMR spectra for compound **4i’**

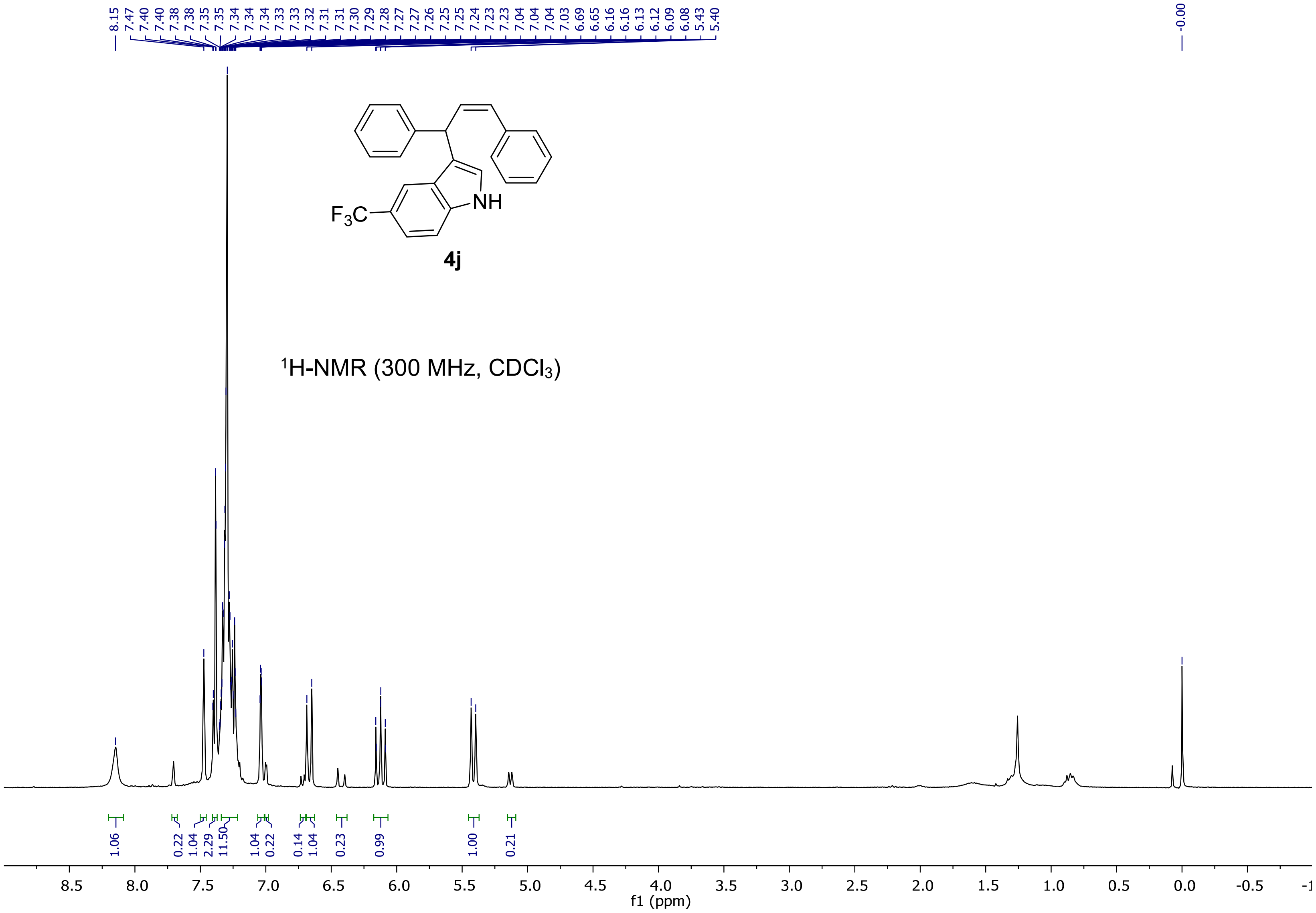

Supplementary Figure 66. ^{1}H-NMR spectra for compound **4j**

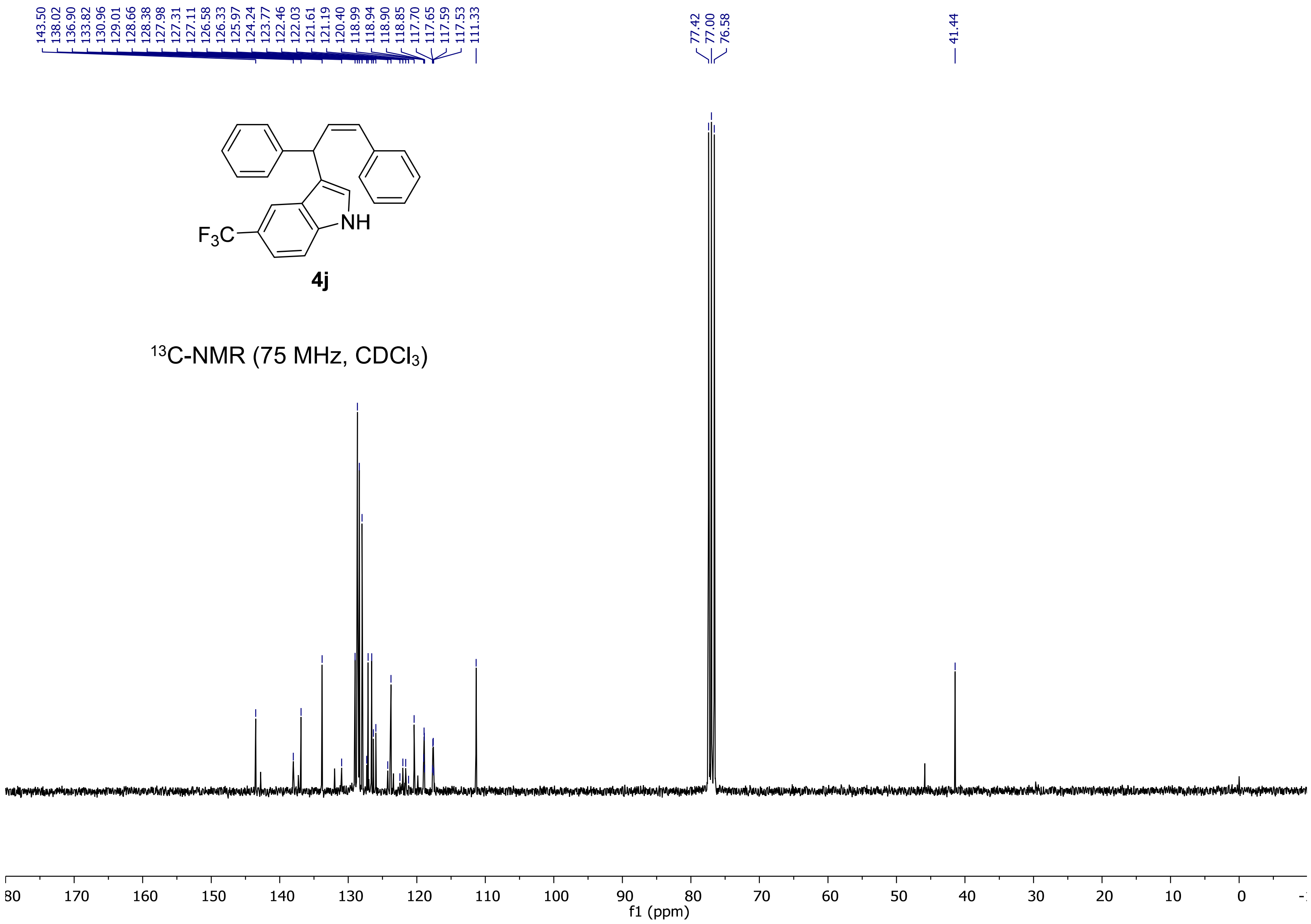

Supplementary Figure 67. ^{13}C-NMR spectra for compound **4j**

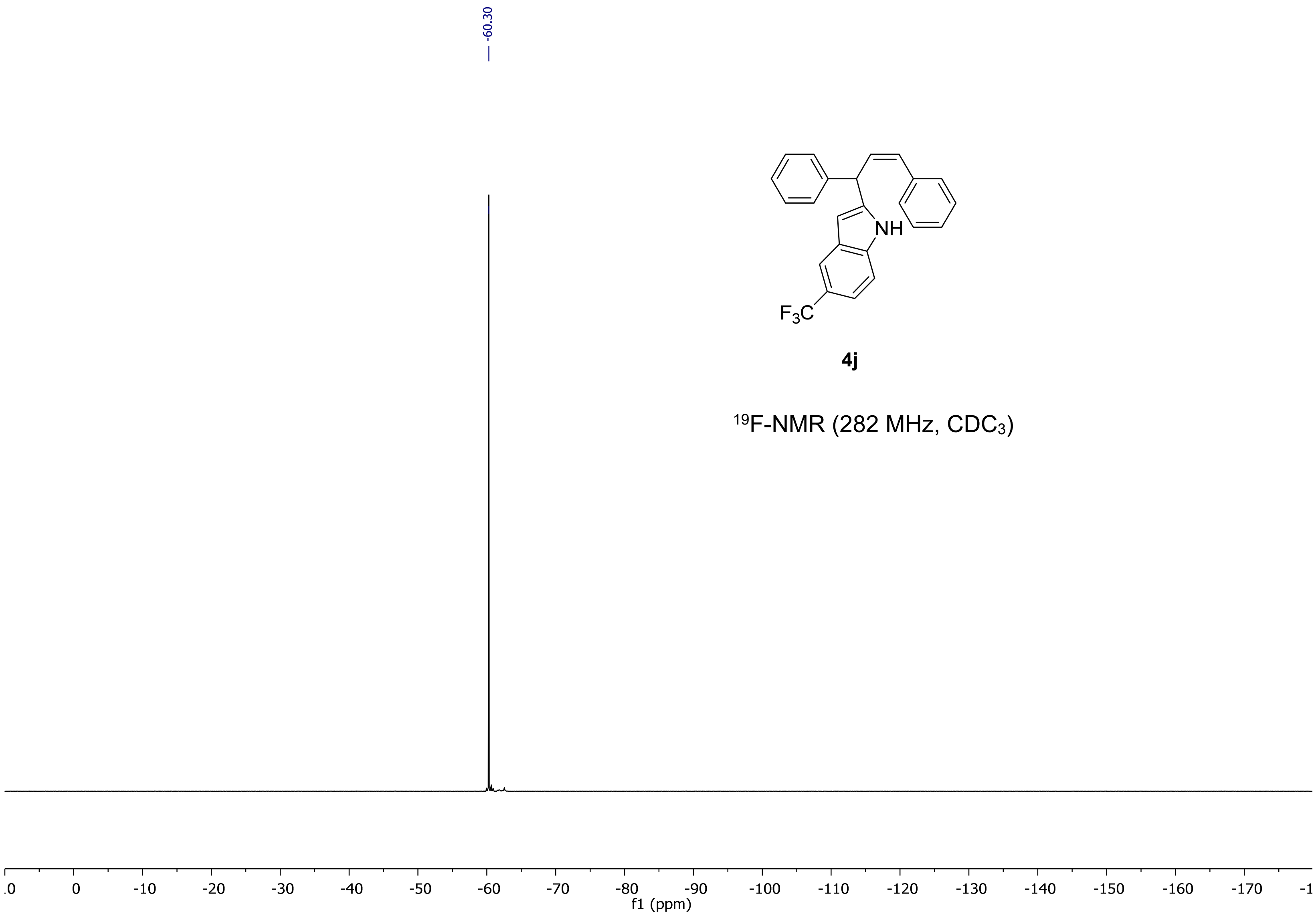

Supplementary Figure 68. ^{19}F-NMR spectra for compound **4j**

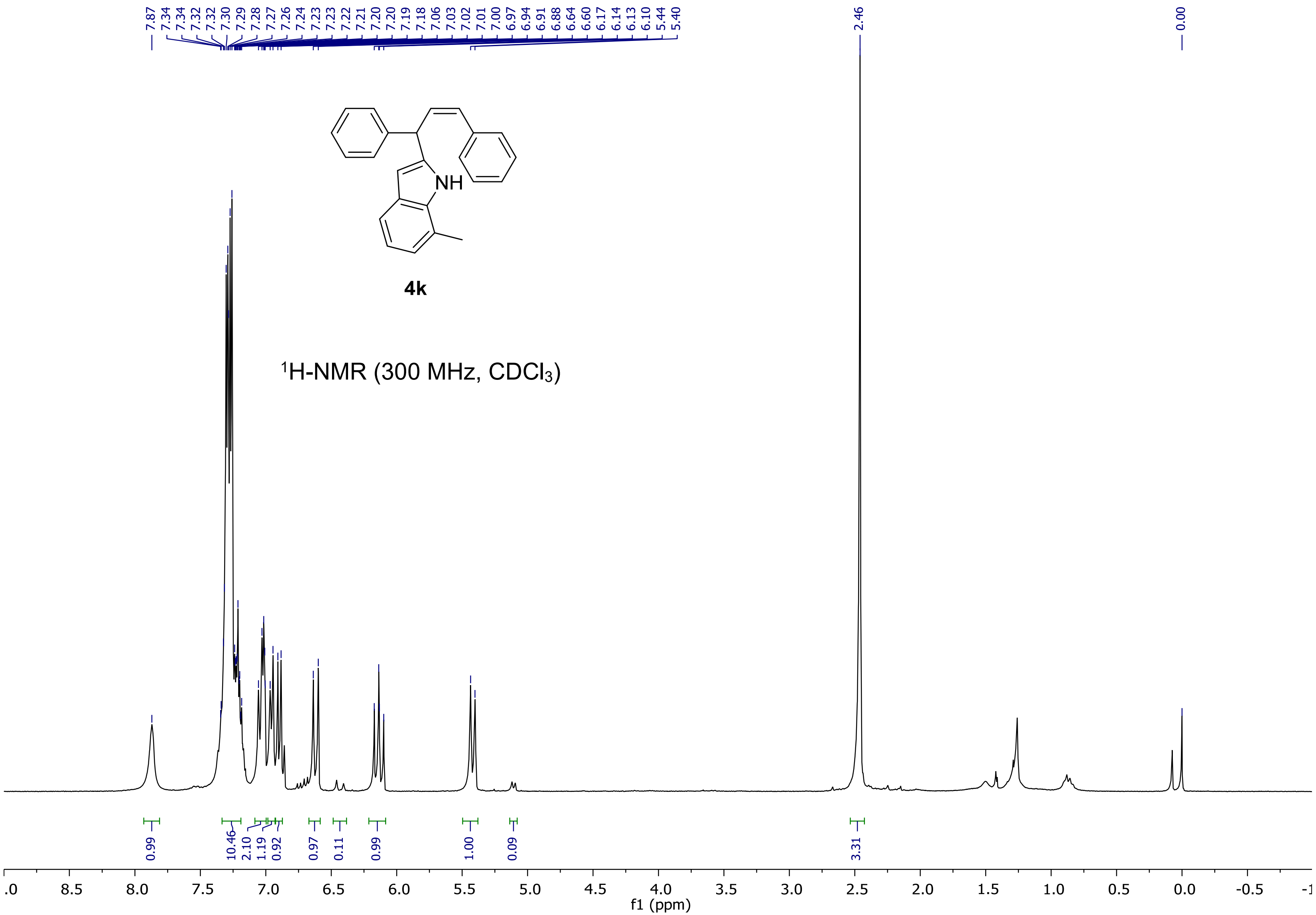

Supplementary Figure 69. ^{1}H-NMR spectra for compound **4k**

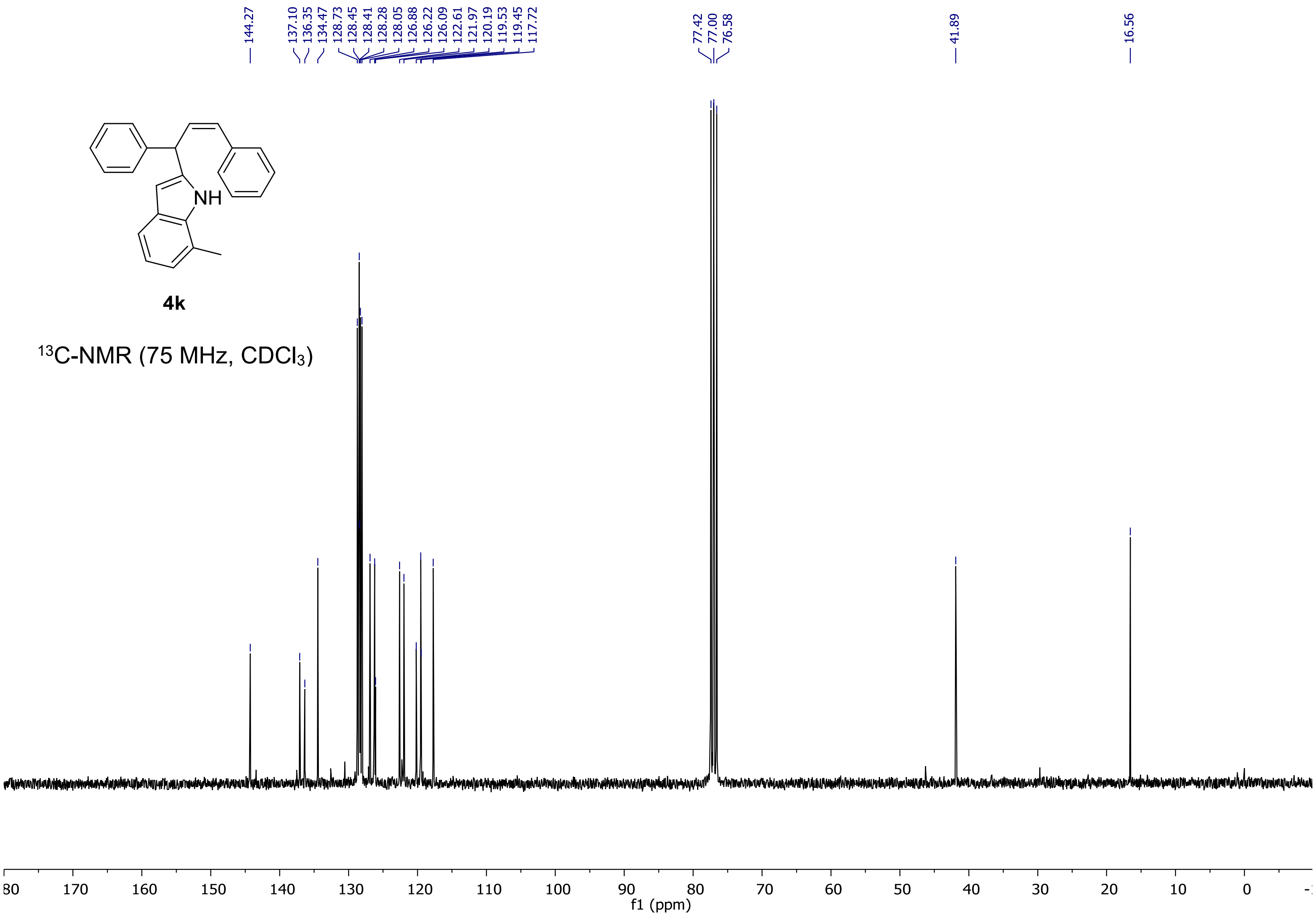

Supplementary Figure 70. ^{13}C-NMR spectra for compound **4k**

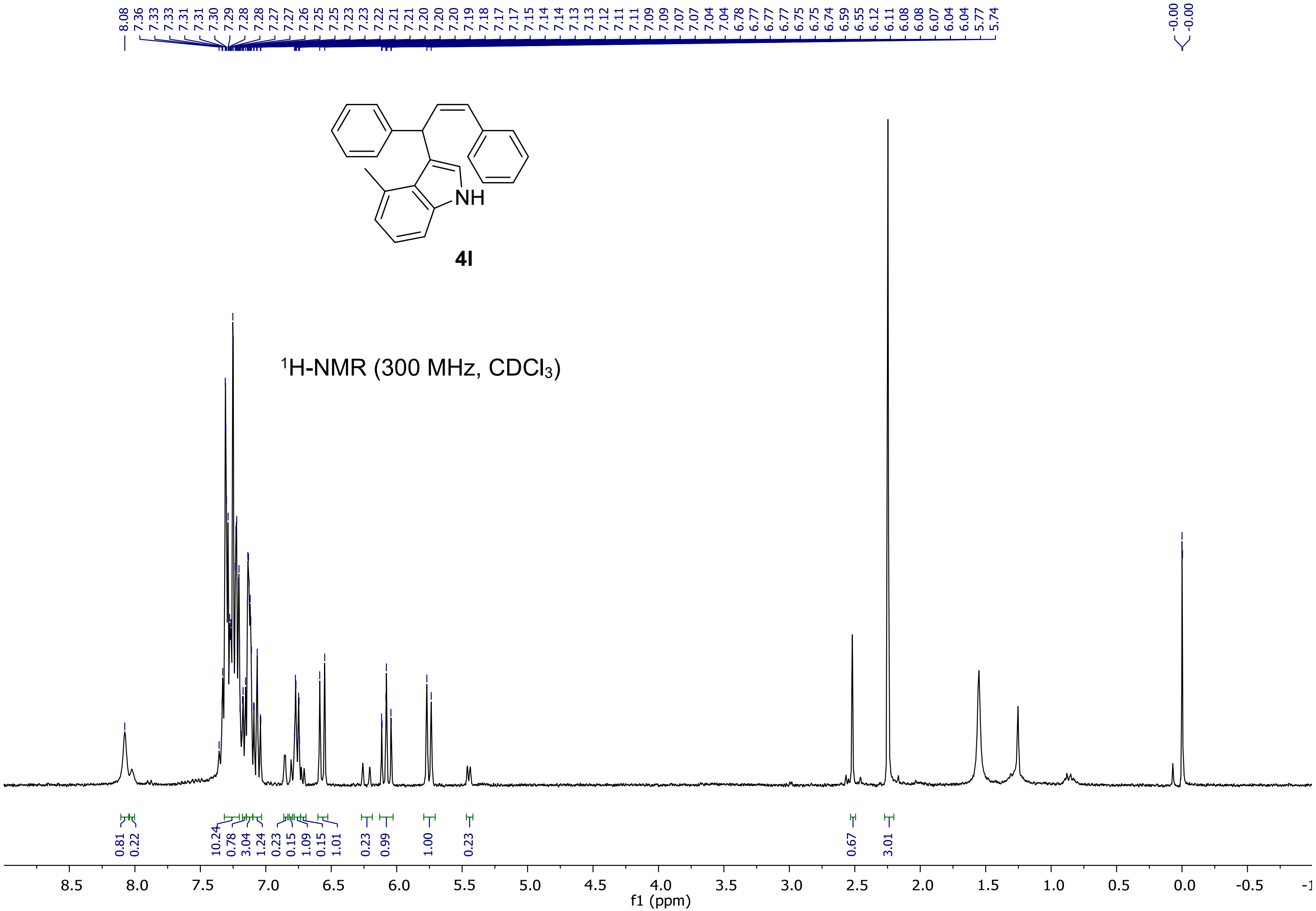

Supplementary Figure 71. ^{1}H-NMR spectra for compound **4l**

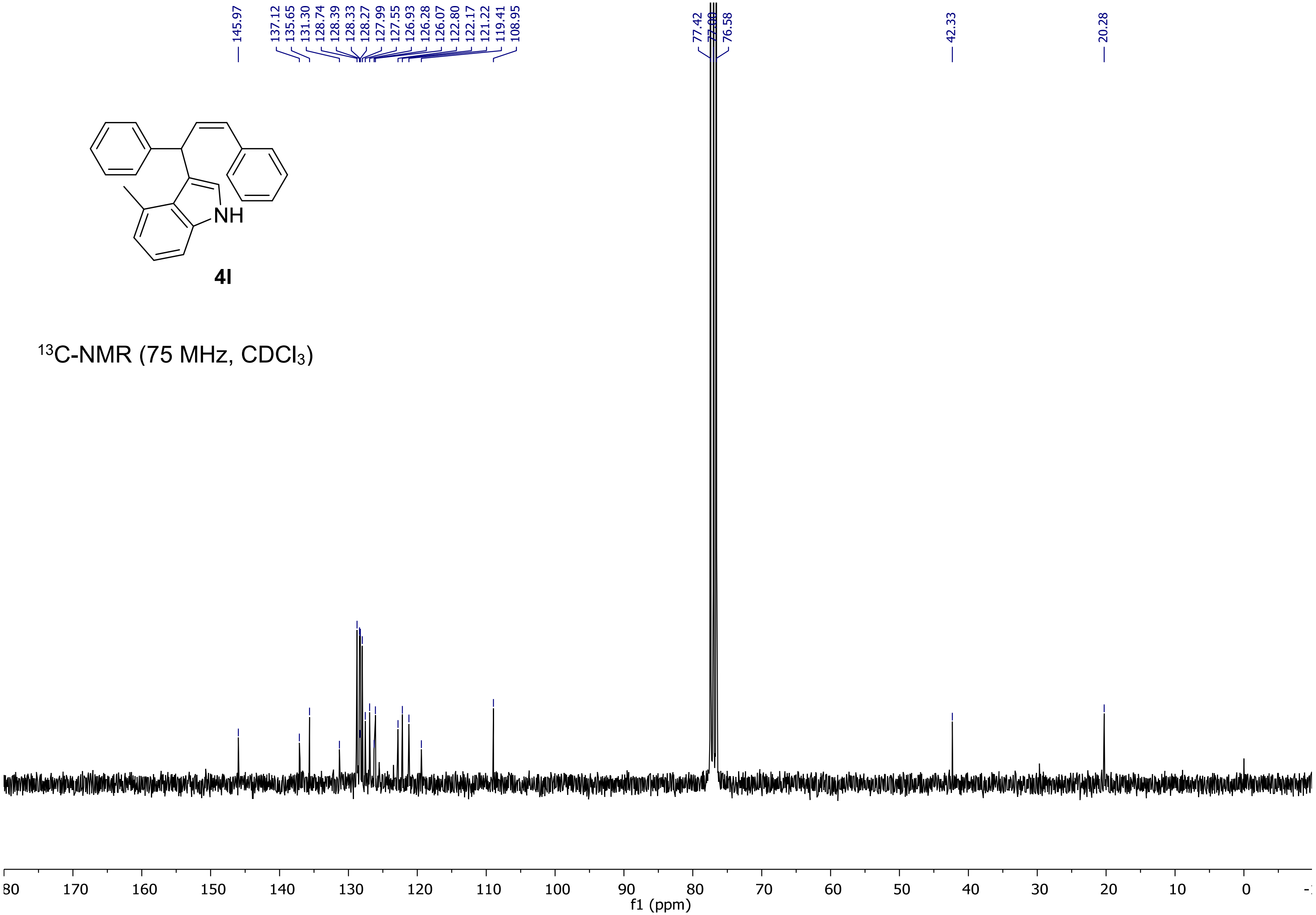

Supplementary Figure 72. ^{13}C-NMR spectra for compound **4l**

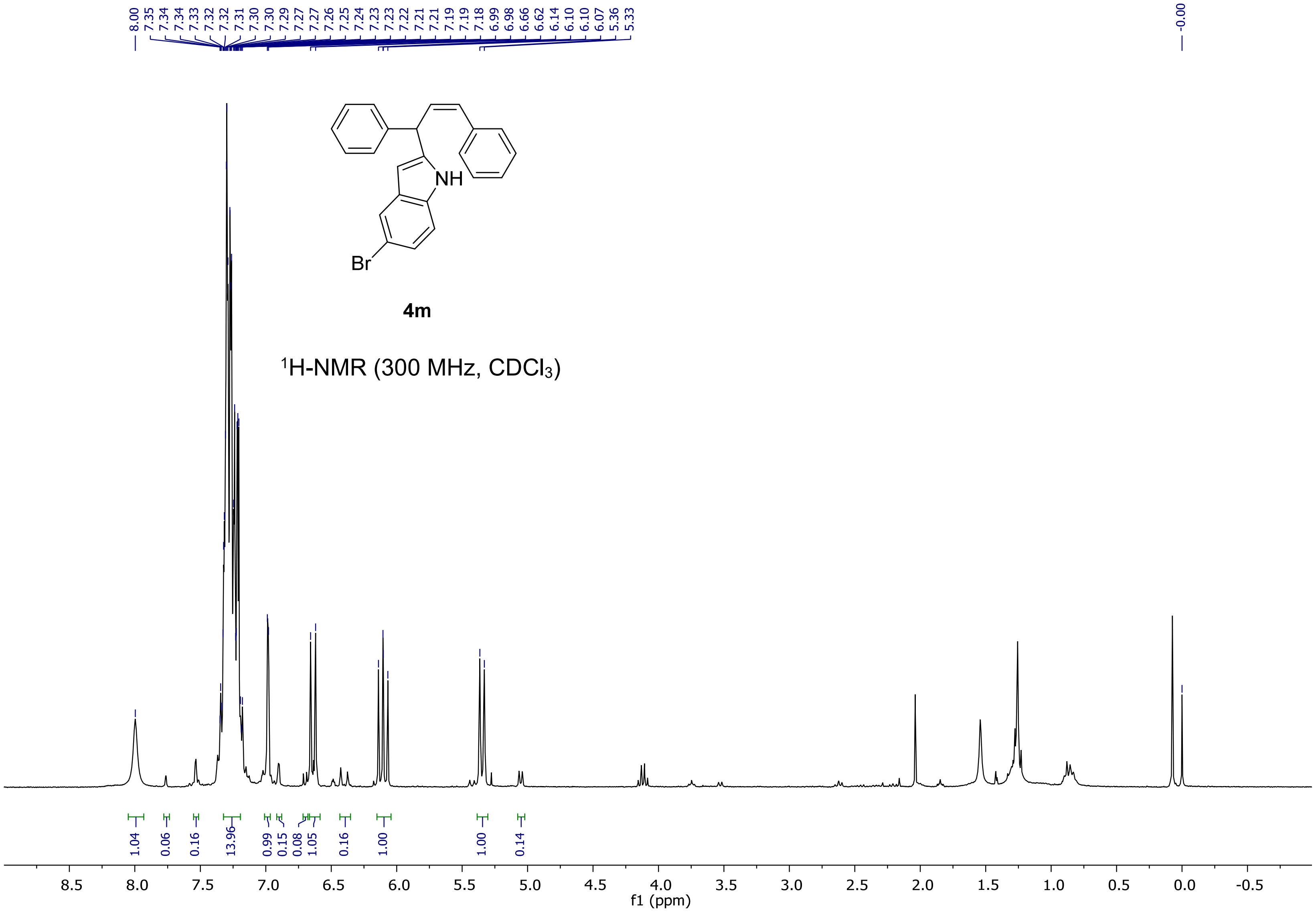

Supplementary Figure 73. ^{1}H-NMR spectra for compound **4m**

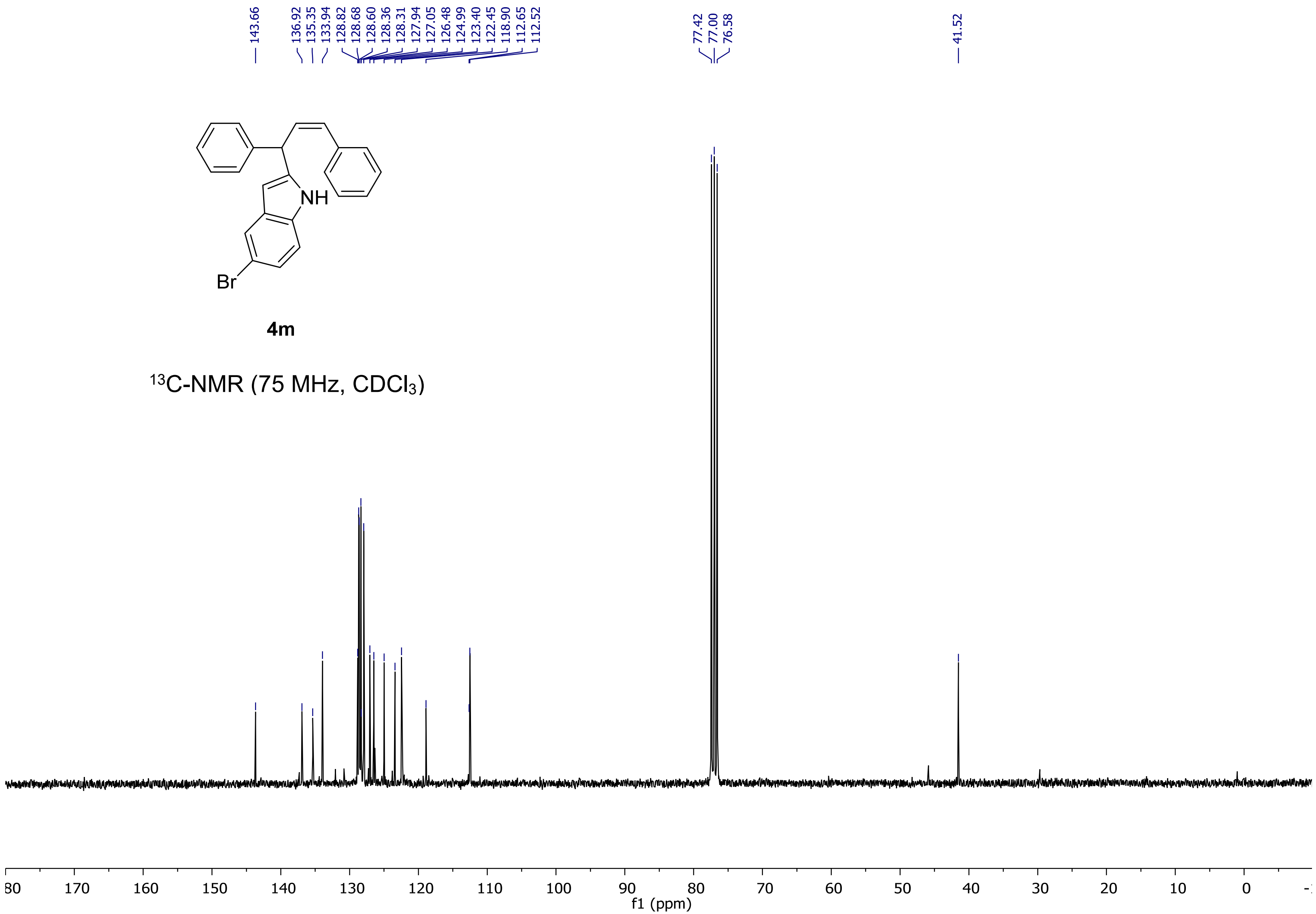

Supplementary Figure 74. ^{13}C-NMR spectra for compound **4m**

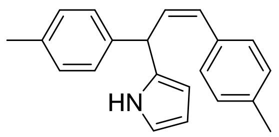

4n

^{1}H-NMR (300 MHz, $CDCl_3$)

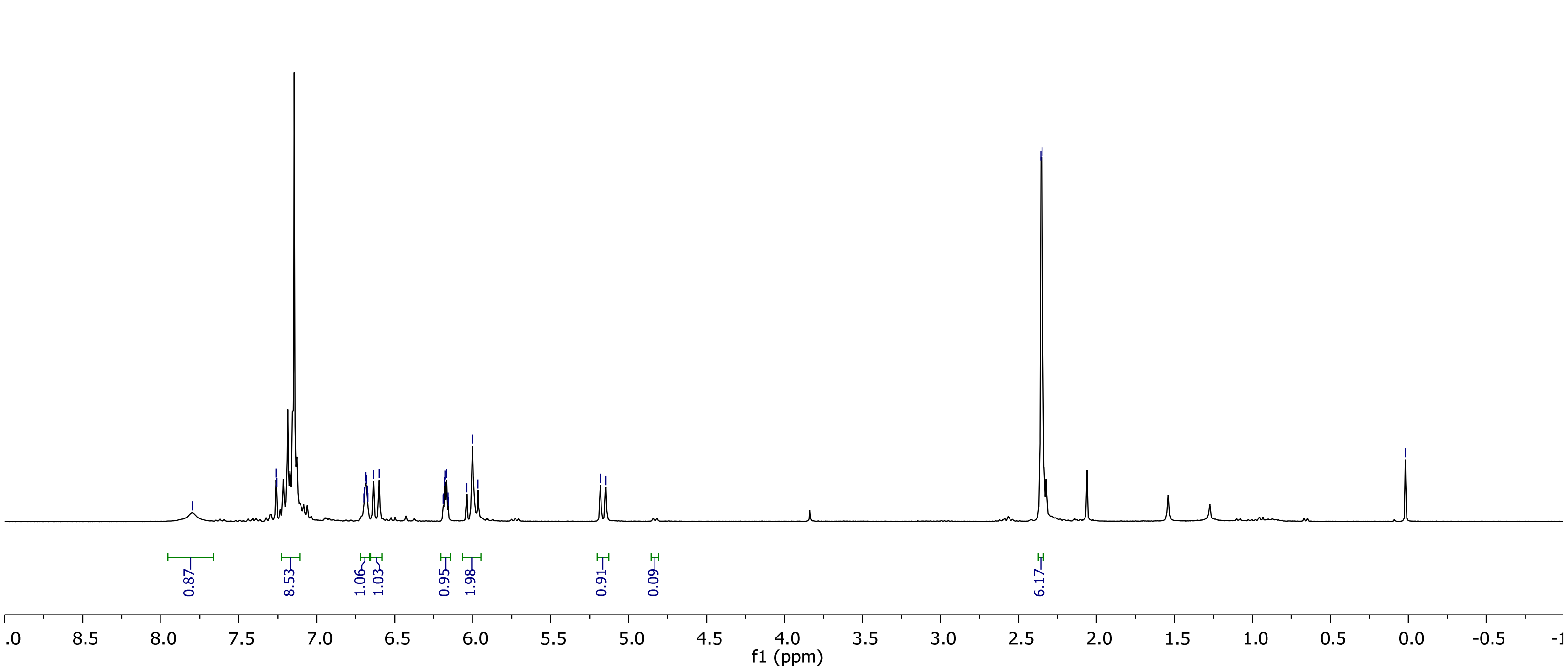

Supplementary Figure 75. ^{1}H-NMR spectra for compound **4n**

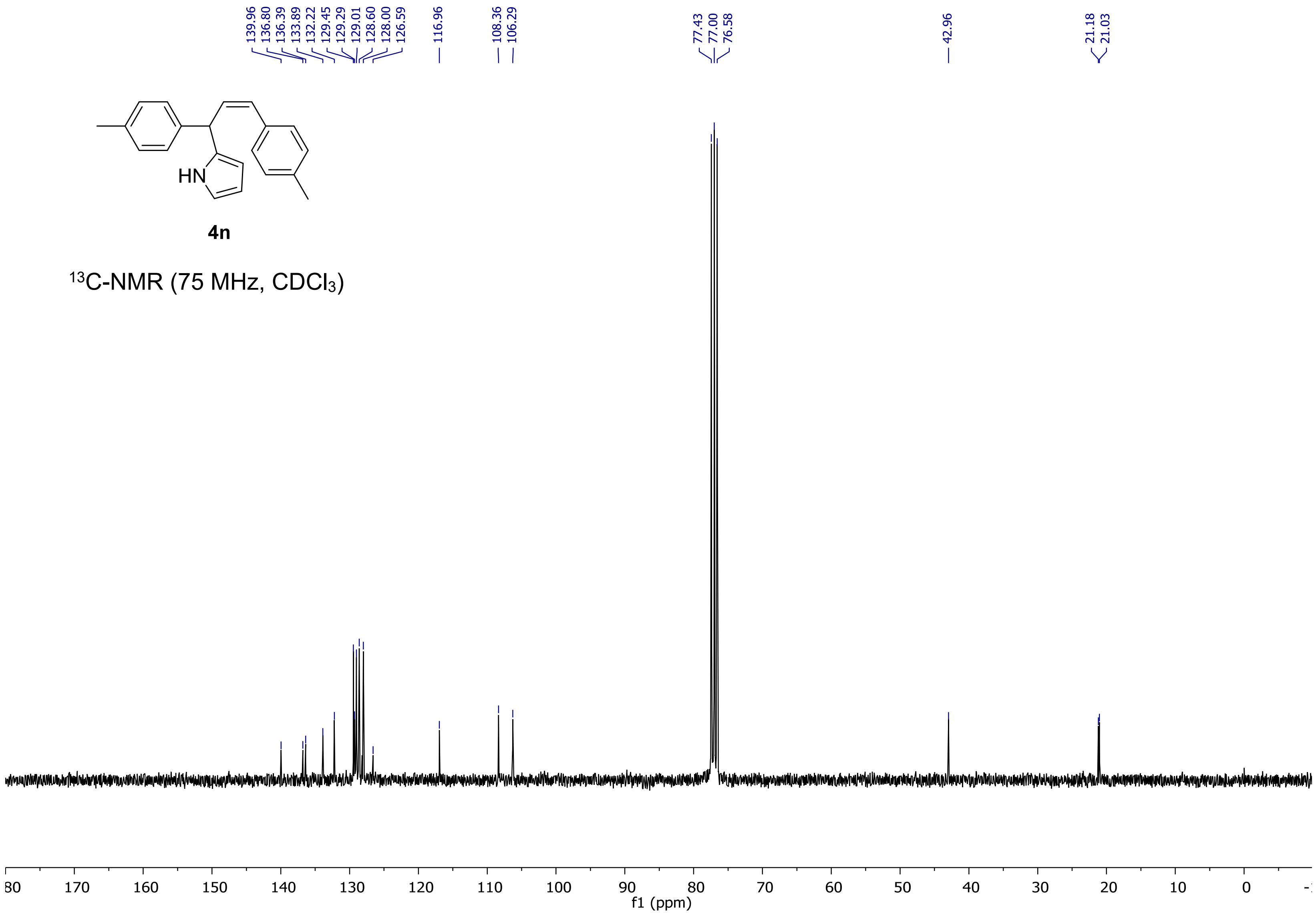

Supplementary Figure 76. ^{13}C-NMR spectra for compound **4n**

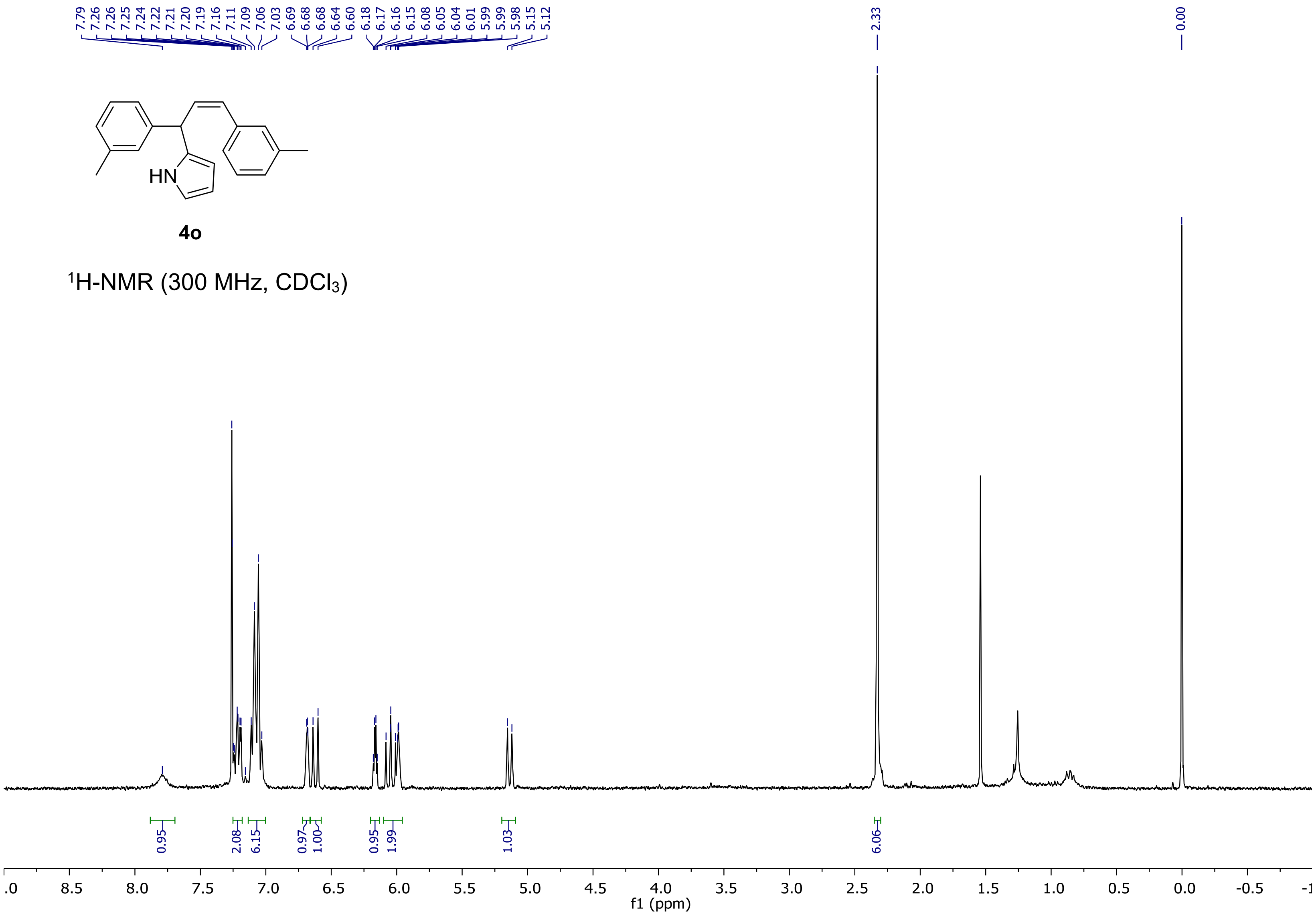

Supplementary Figure 77. ^{1}H-NMR spectra for compound **4o**

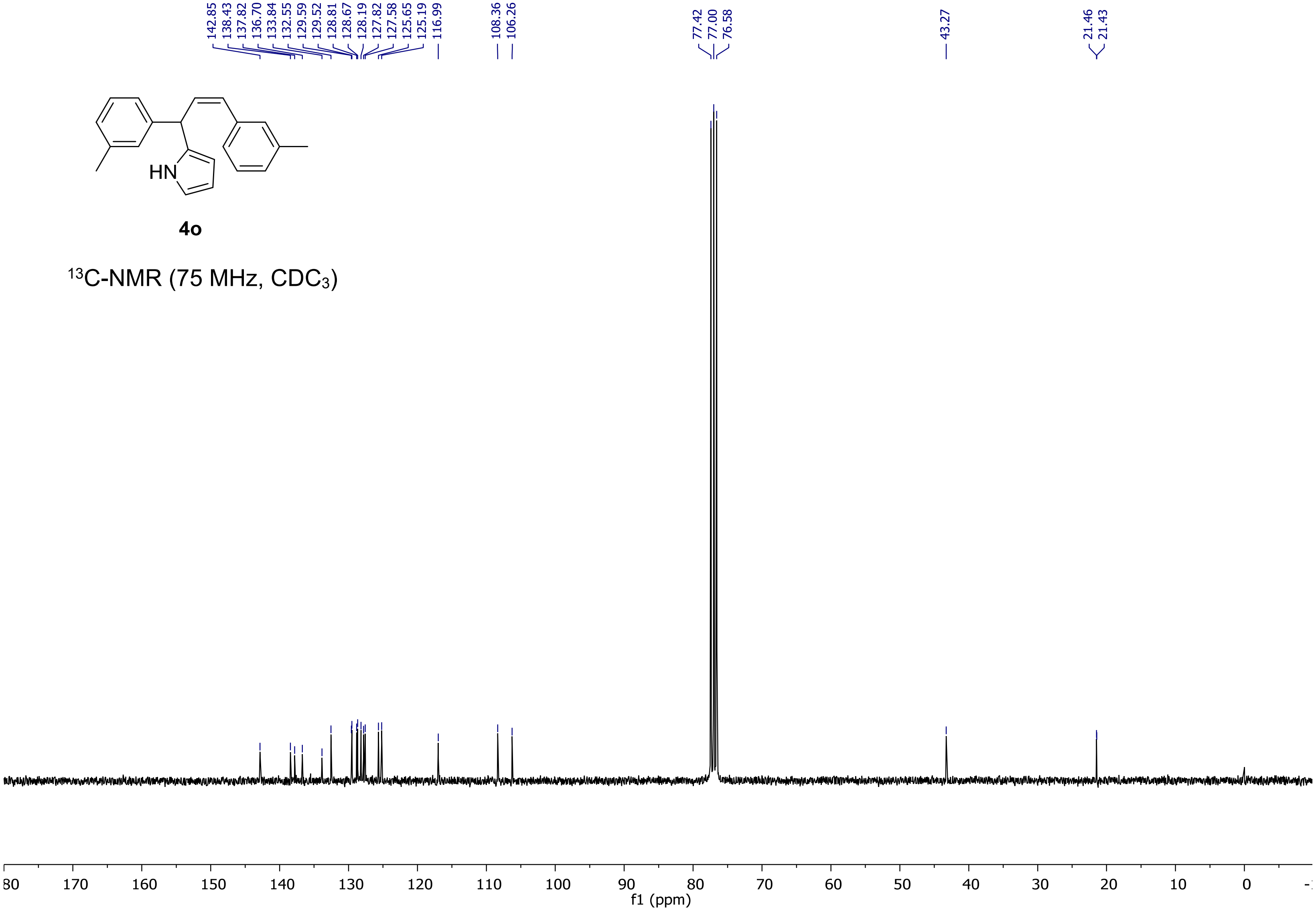

Supplementary Figure 78. ^{13}C-NMR spectra for compound **4o**

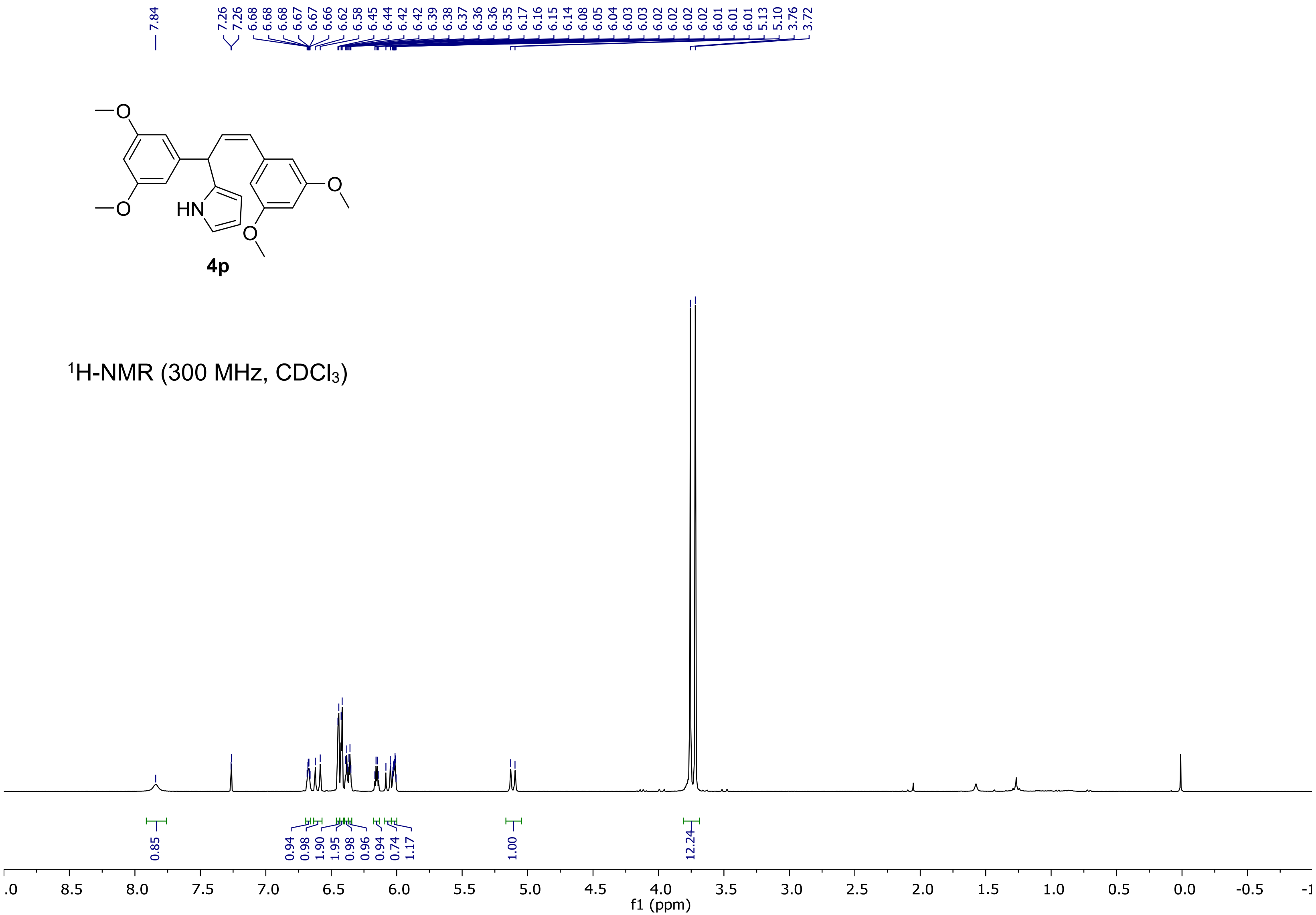

Supplementary Figure 79. ^{1}H-NMR spectra for compound **4p**

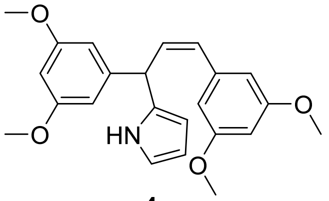

4p

^{13}C-NMR (300 MHz, $CDCl_3$)

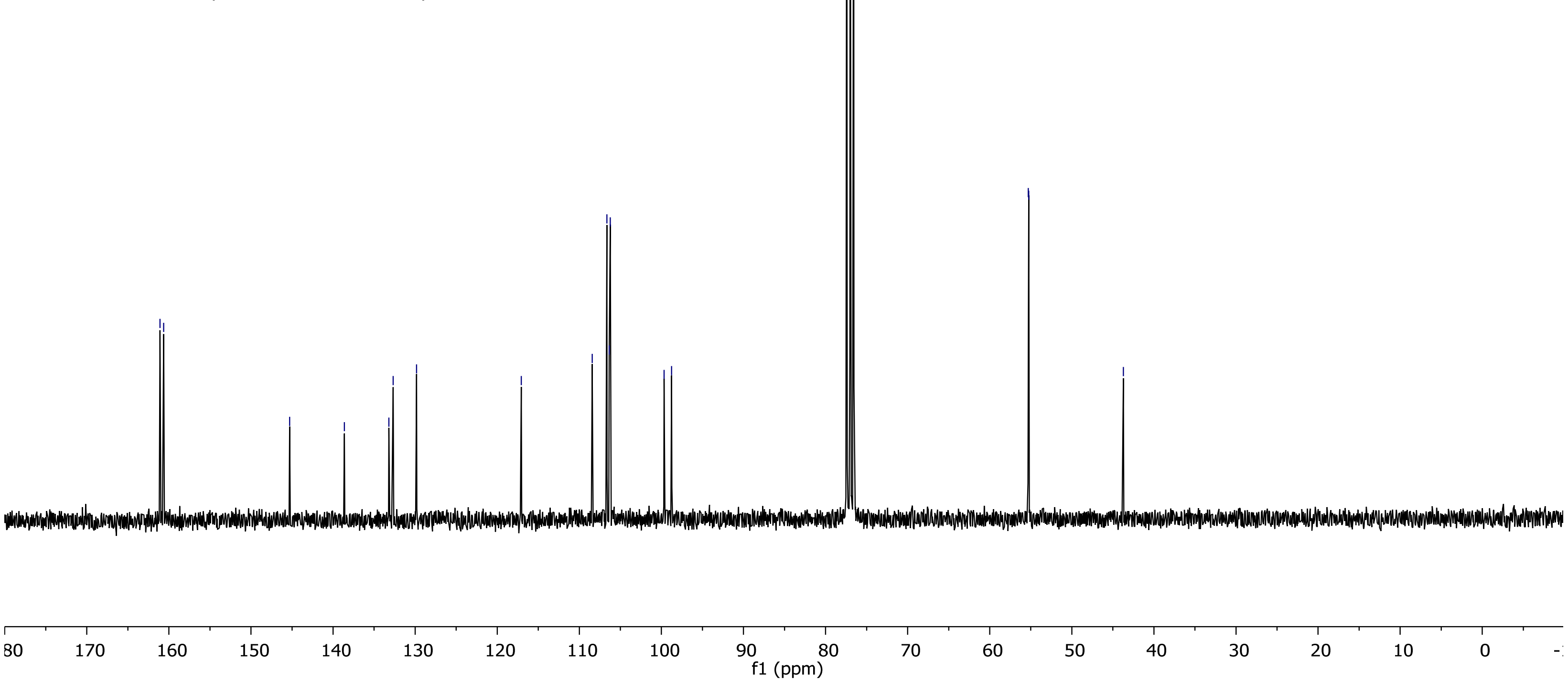

Supplementary Figure 80. ^{13}C-NMR spectra for compound **4p**

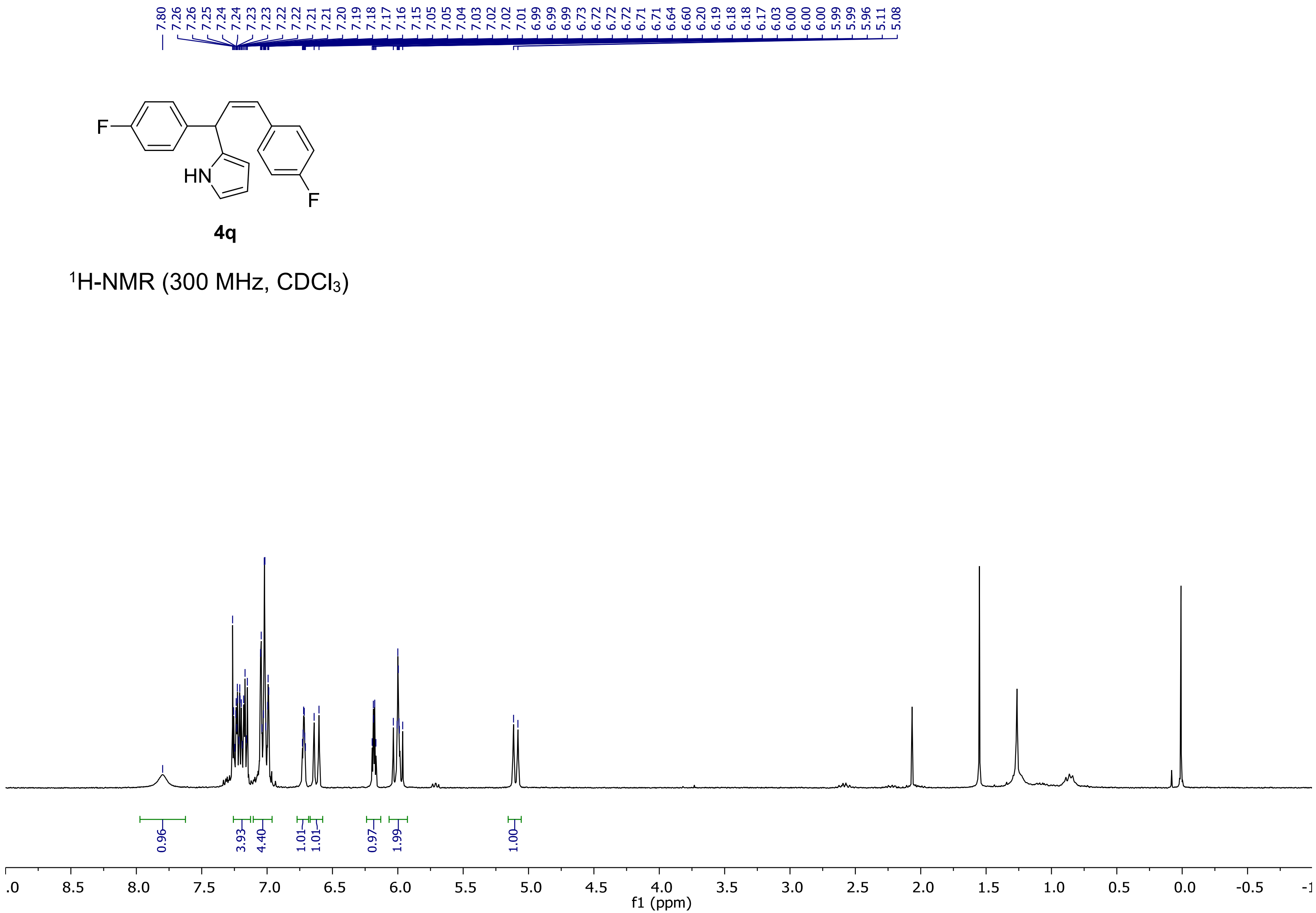

Supplementary Figure 81. ^{1}H-NMR spectra for compound **4q**

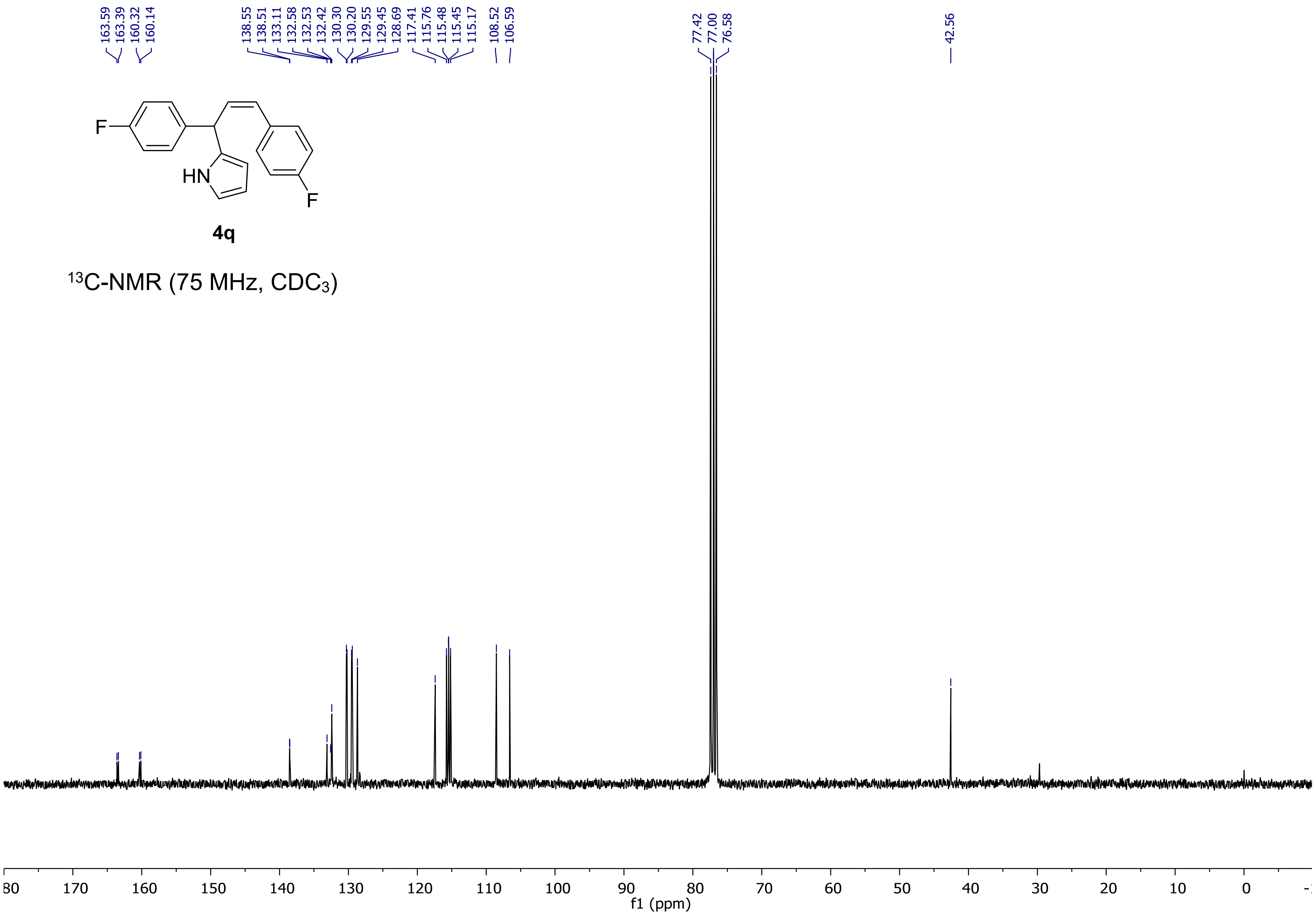

Supplementary Figure 82. ^{13}C-NMR spectra for compound **4q**

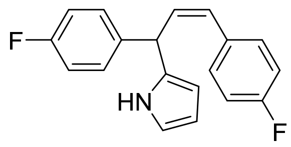

4q

^{19}F-NMR (282 MHz, CDC$_3$)

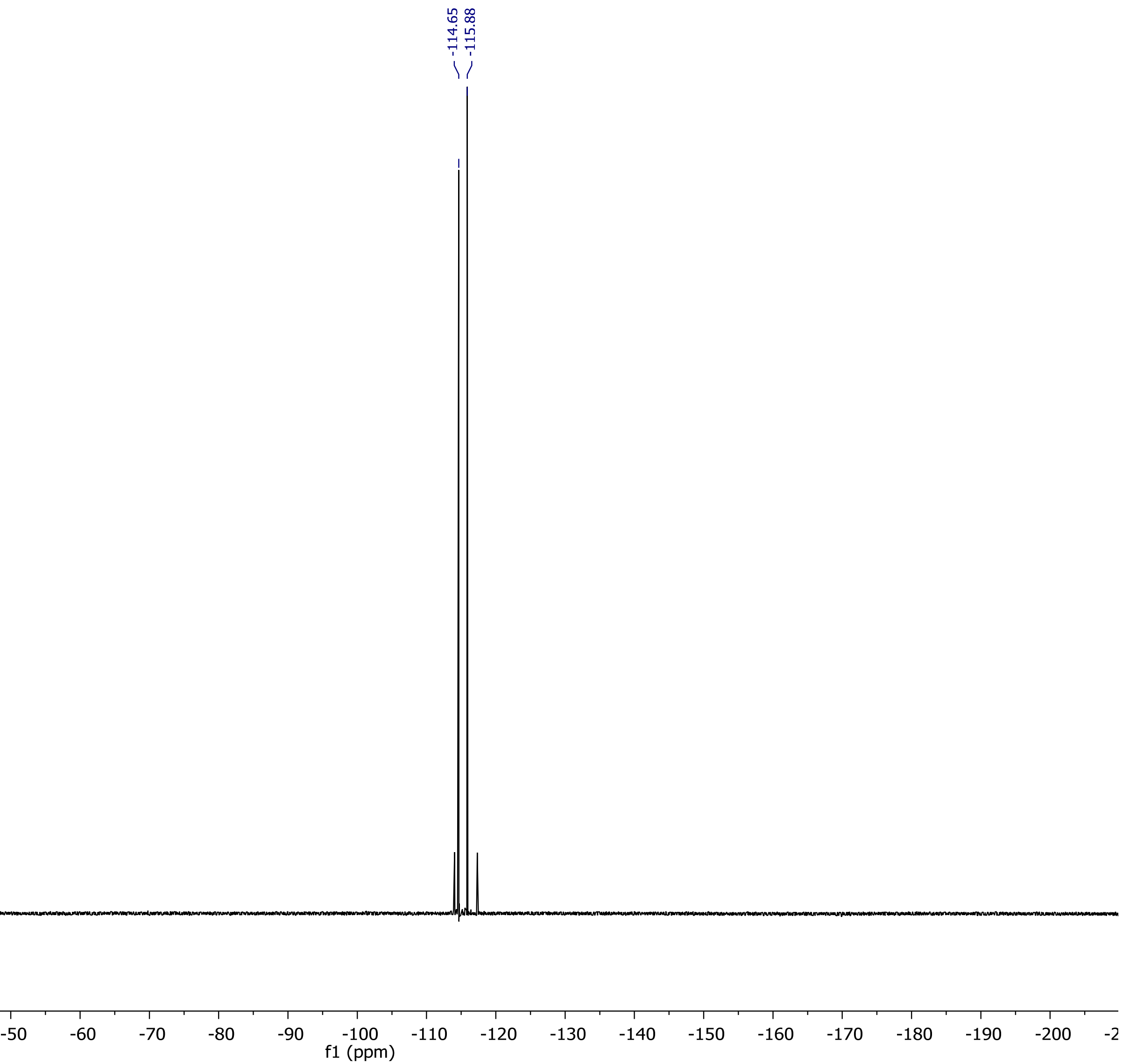

Supplementary Figure 83. ^{19}F-NMR spectra for compound **4q**

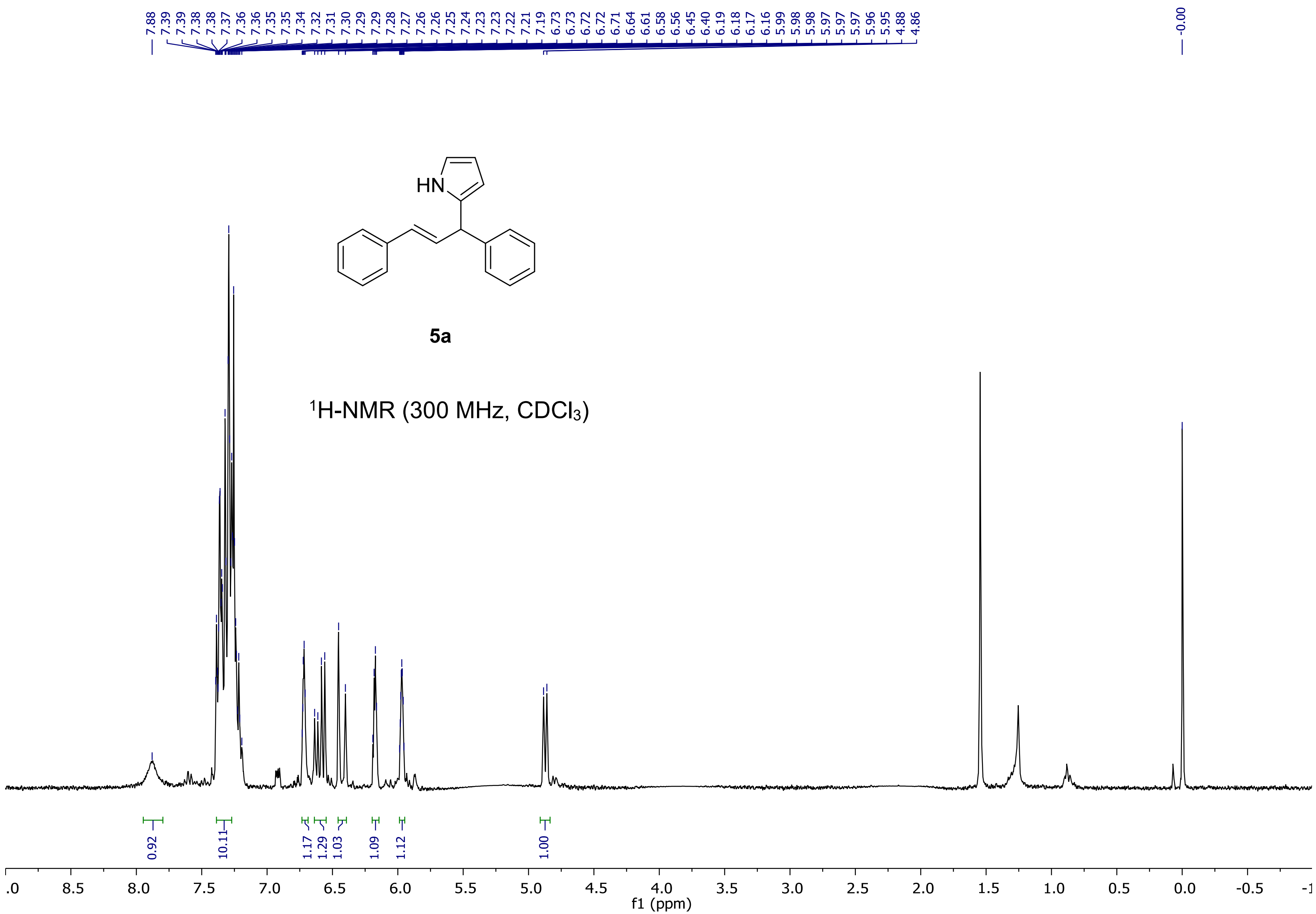

Supplementary Figure 84. ^{1}H-NMR spectra for compound **5a**

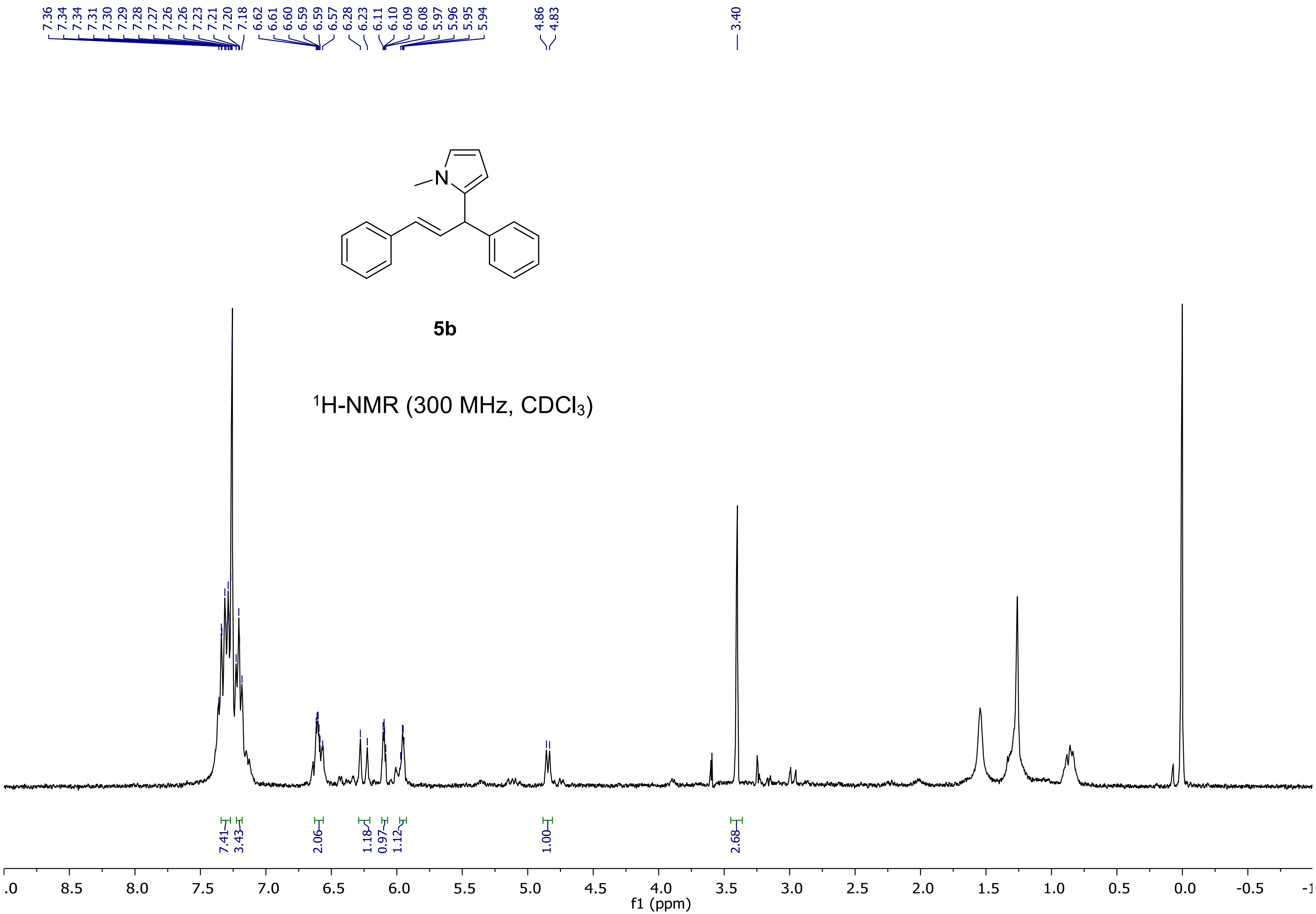

Supplementary Figure 85. ^{1}H-NMR spectra for compound **5b**

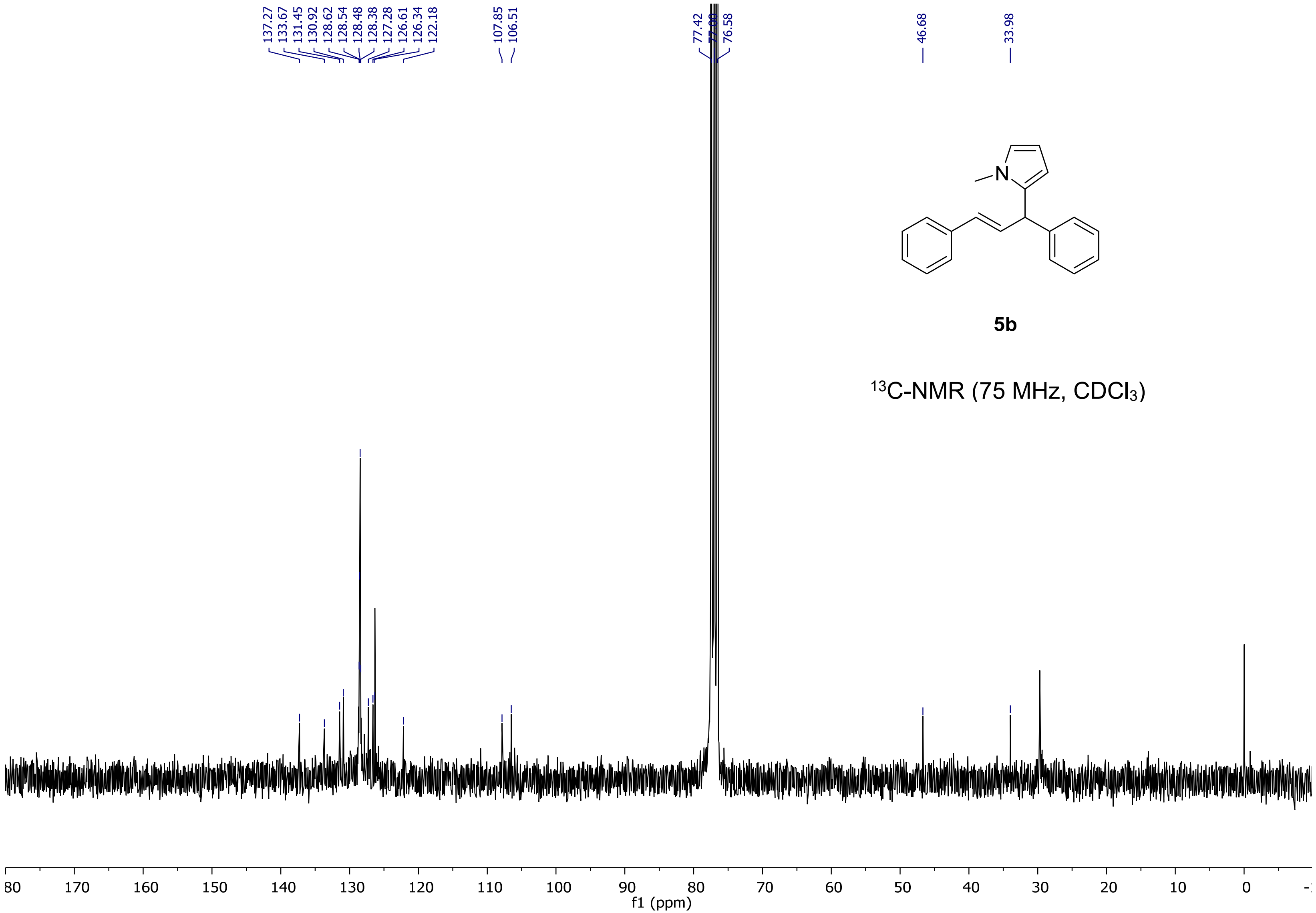

Supplementary Figure 86. ^{13}C-NMR spectra for compound **5b**

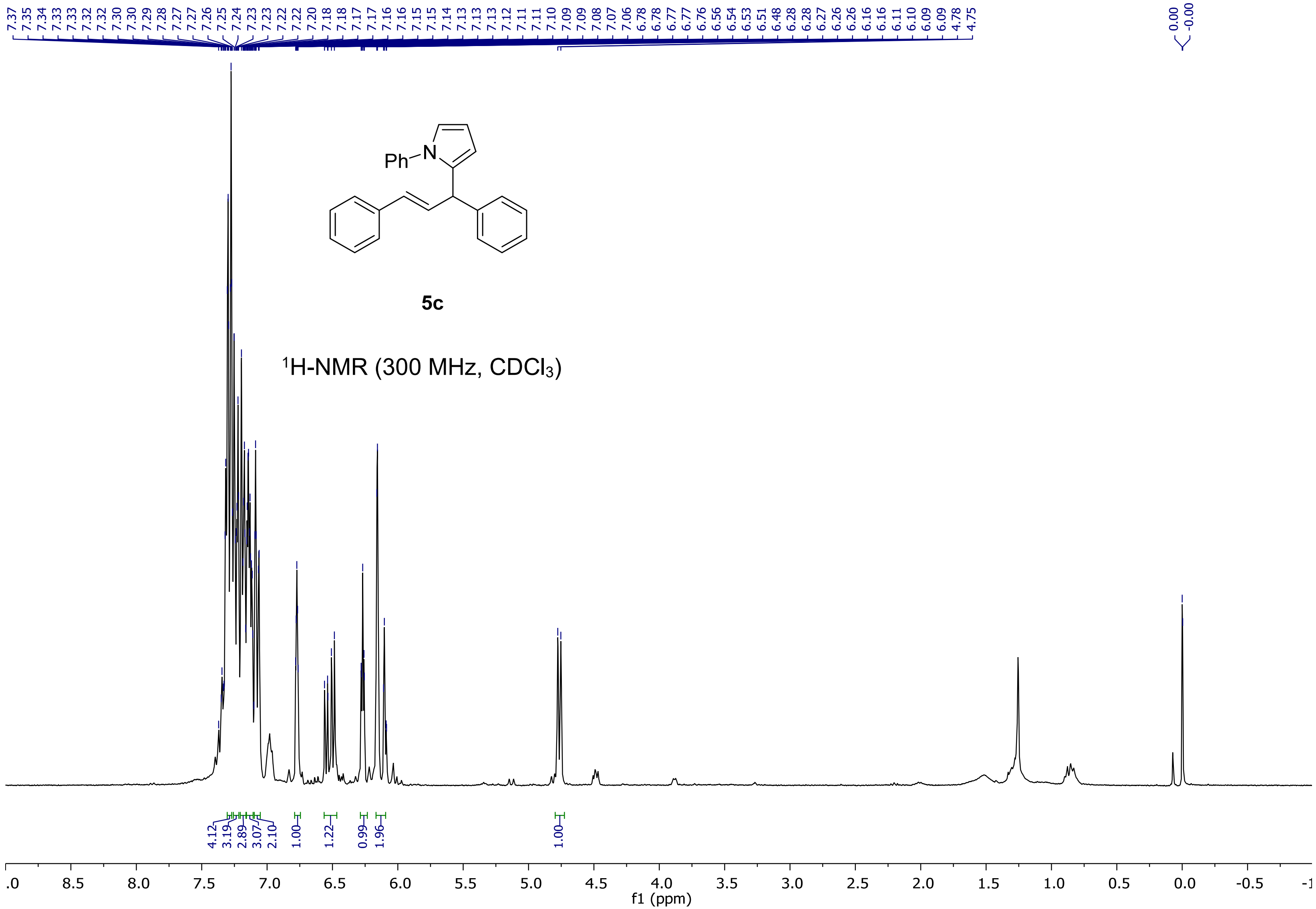

Supplementary Figure 87. ^{1}H-NMR spectra for compound **5c**

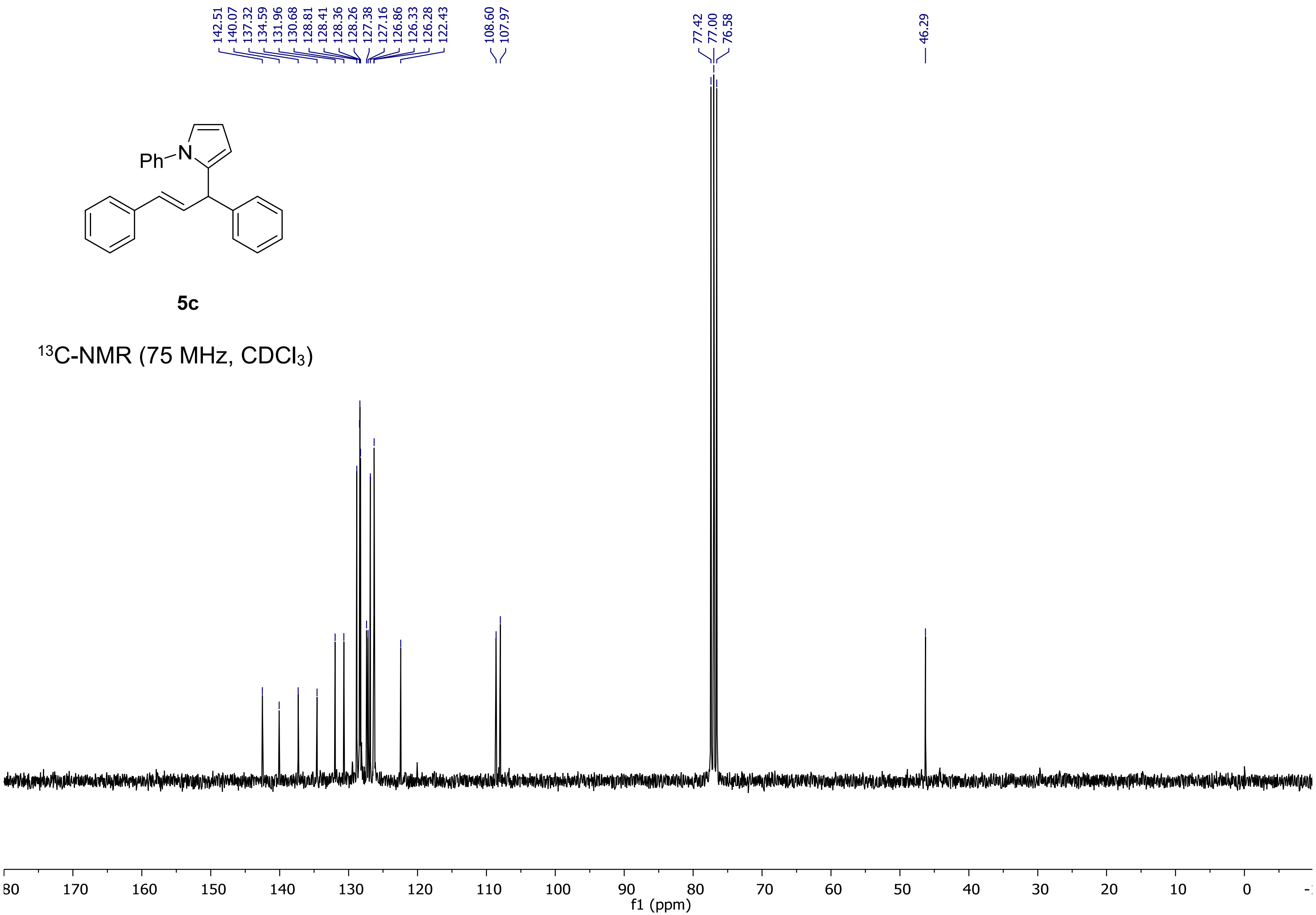

Supplementary Figure 88. ^{13}C-NMR spectra for compound **5c**

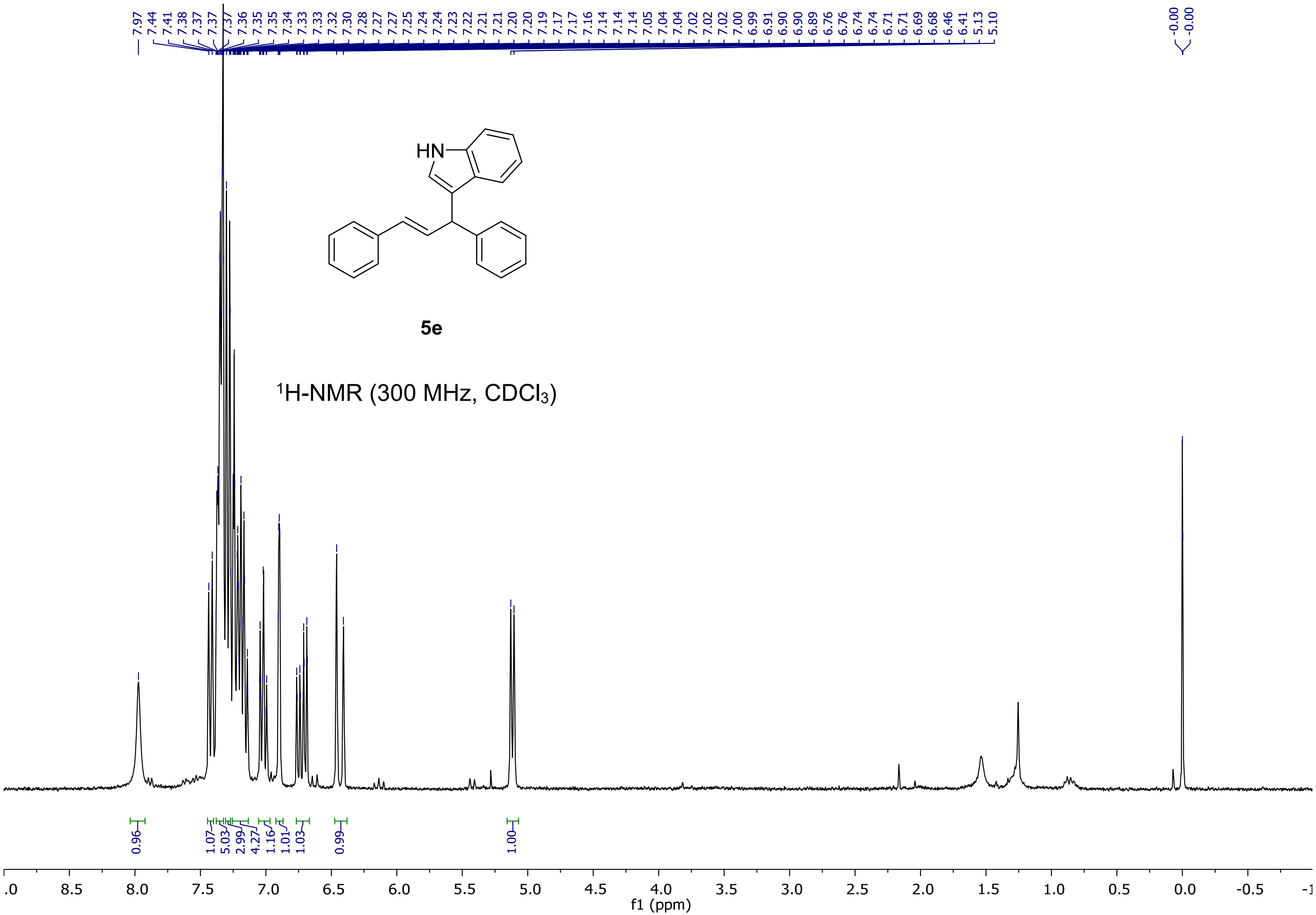

Supplementary Figure 89. ^{1}H-NMR spectra for compound **5e**

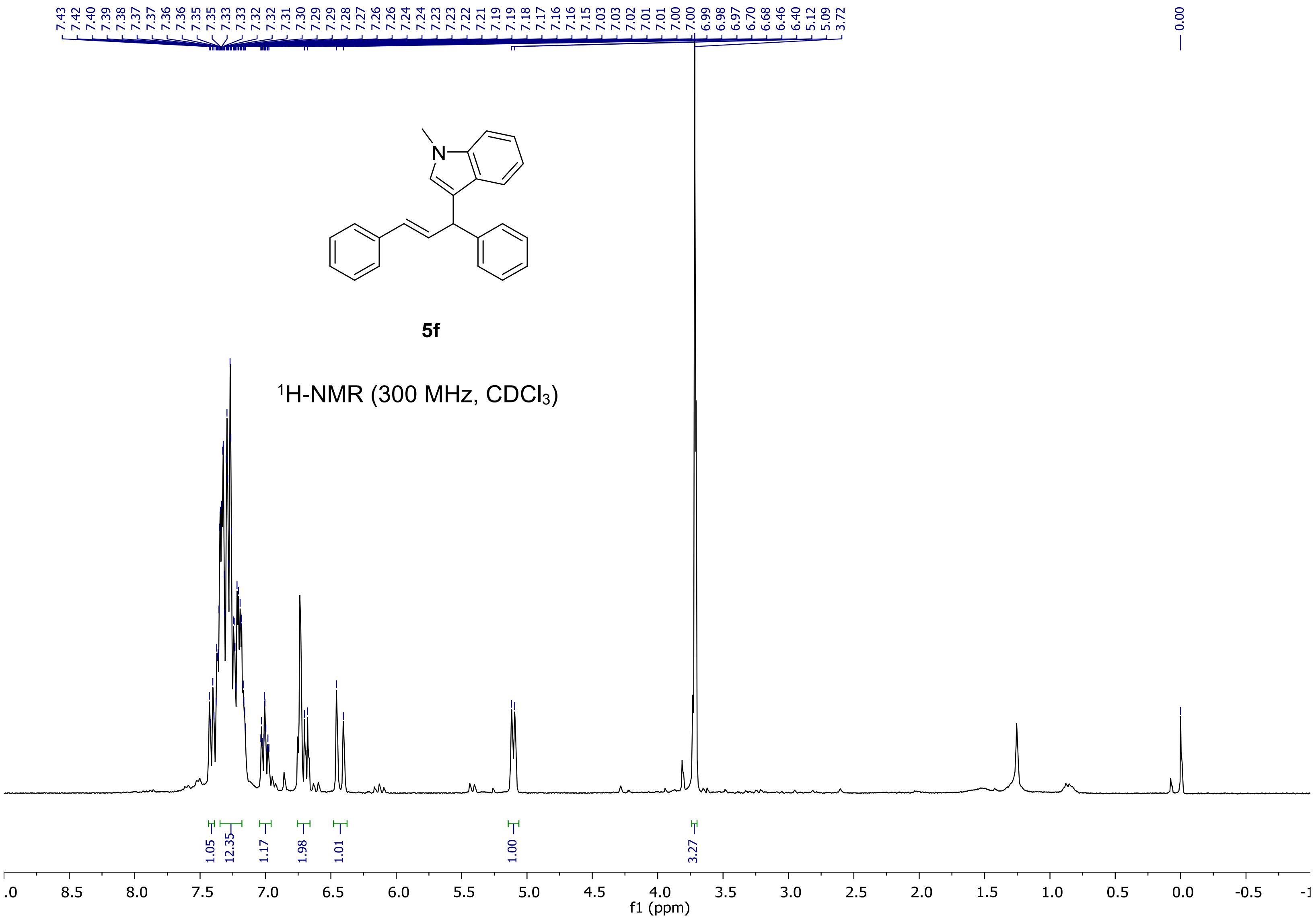

Supplementary Figure 90. ^{1}H-NMR spectra for compound **5f**

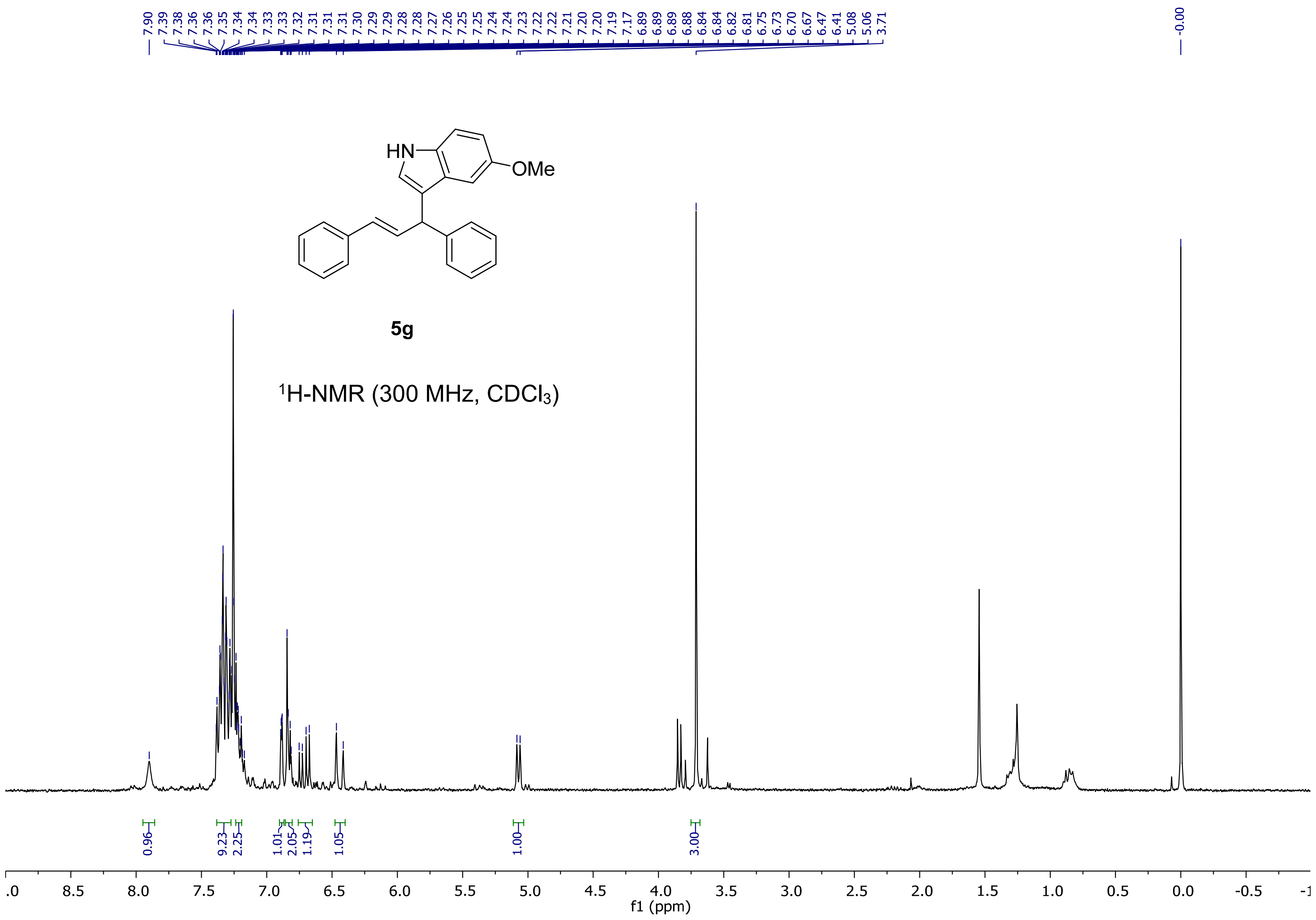

Supplementary Figure 91. ^{1}H-NMR spectra for compound **5g**

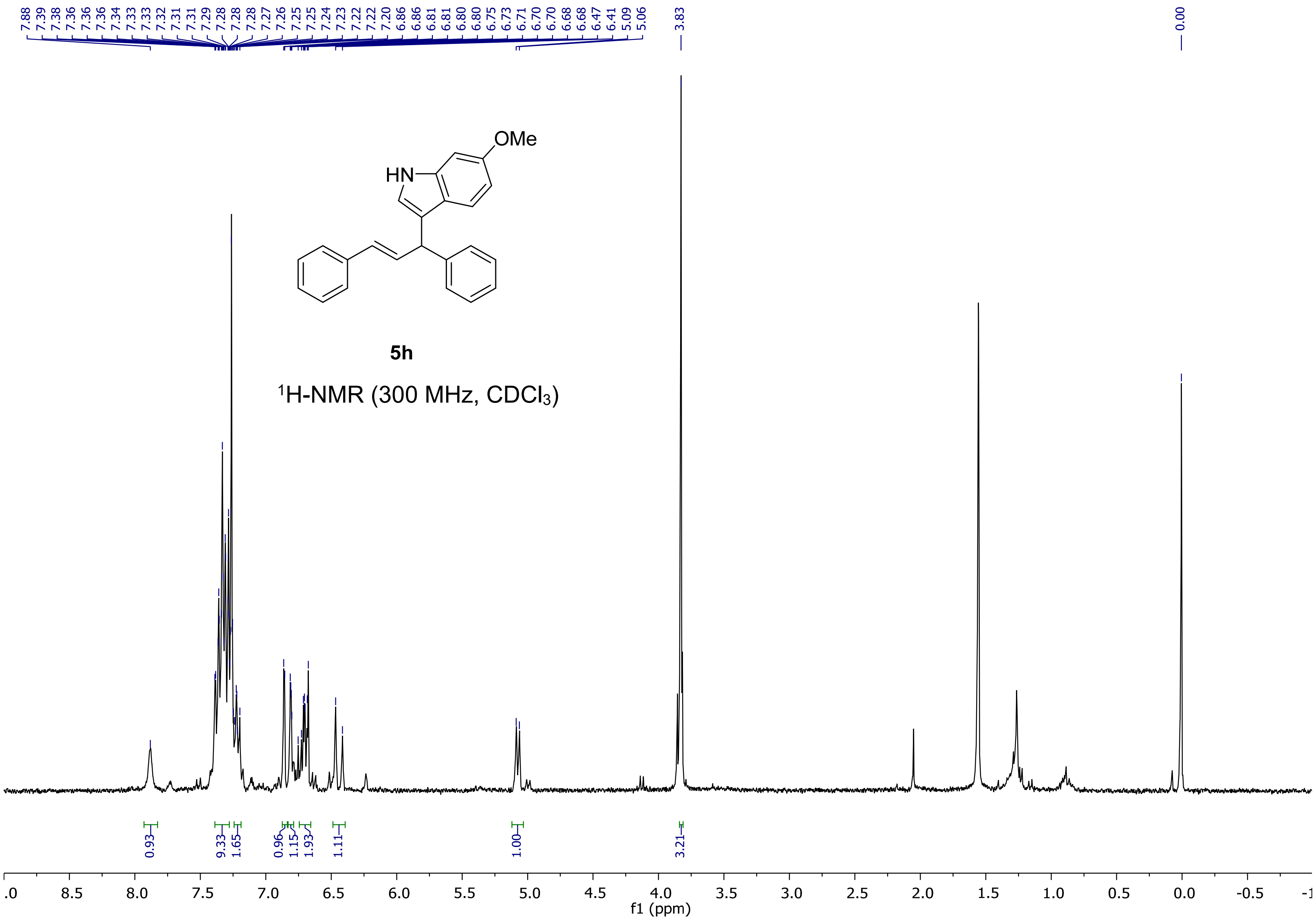

Supplementary Figure 92. ¹H-NMR spectra for compound **5h**

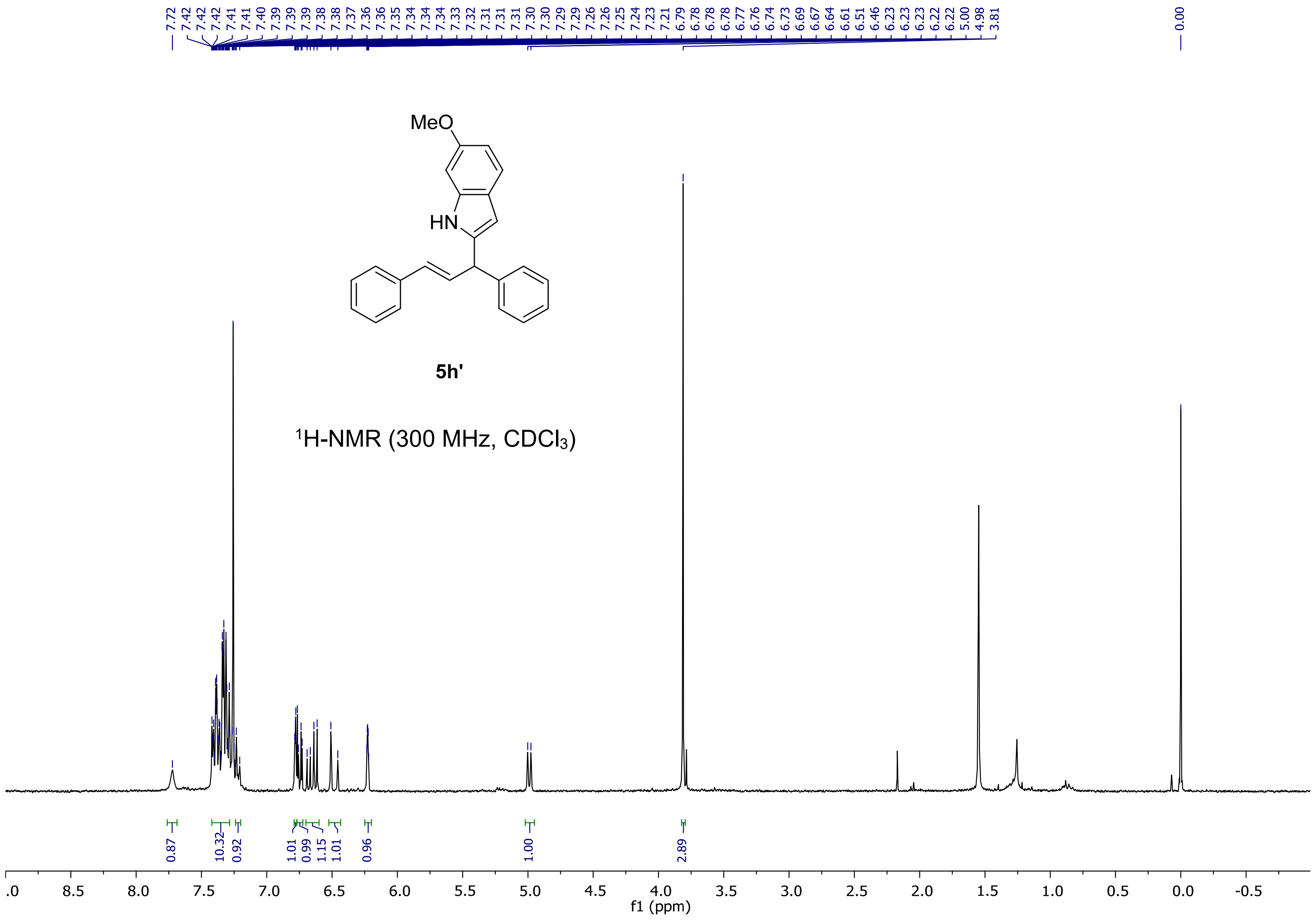

Supplementary Figure 93. ¹H-NMR spectra for compound **5h'**

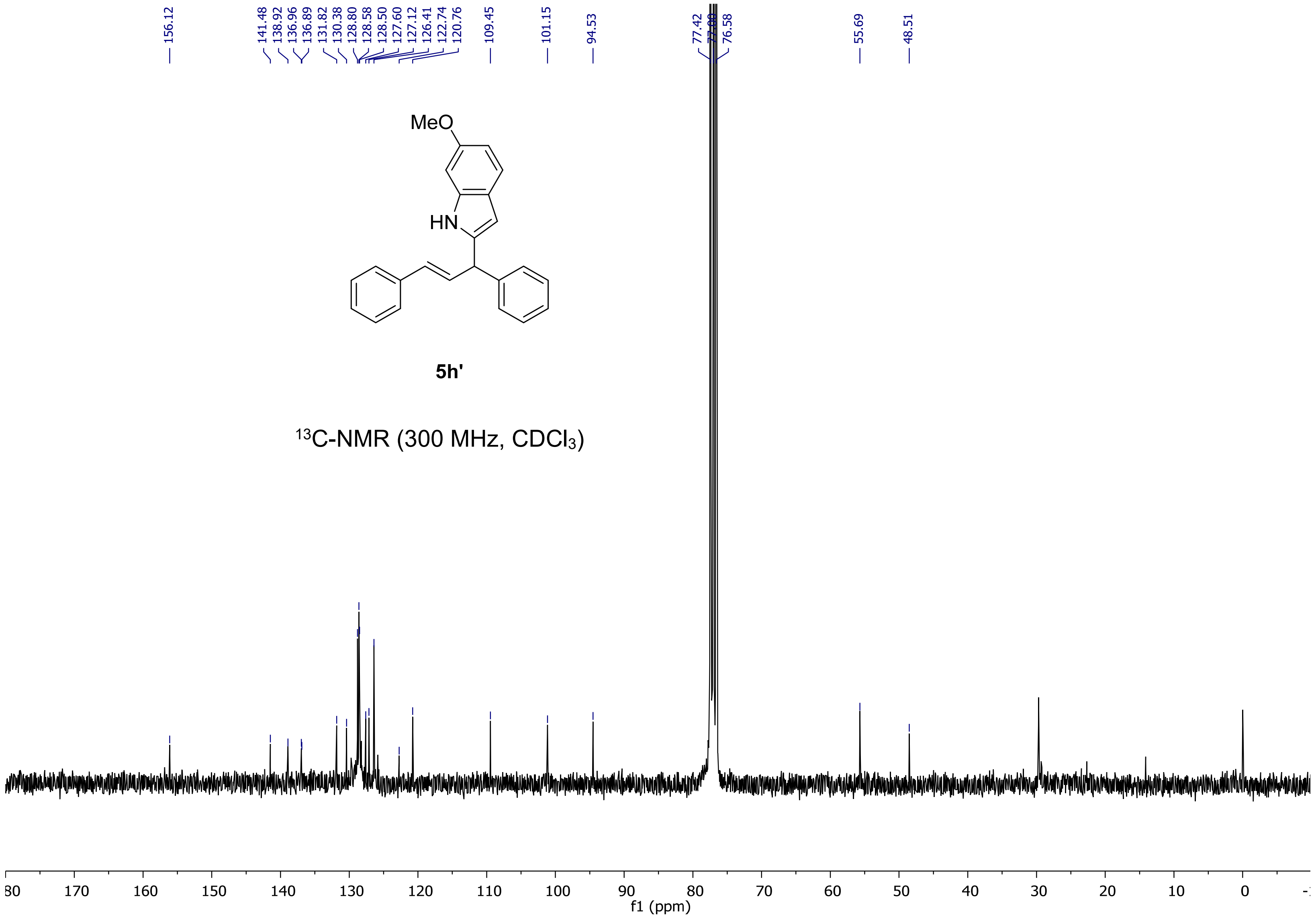

Supplementary Figure 94. ^{13}C-NMR spectra for compound **5h'**

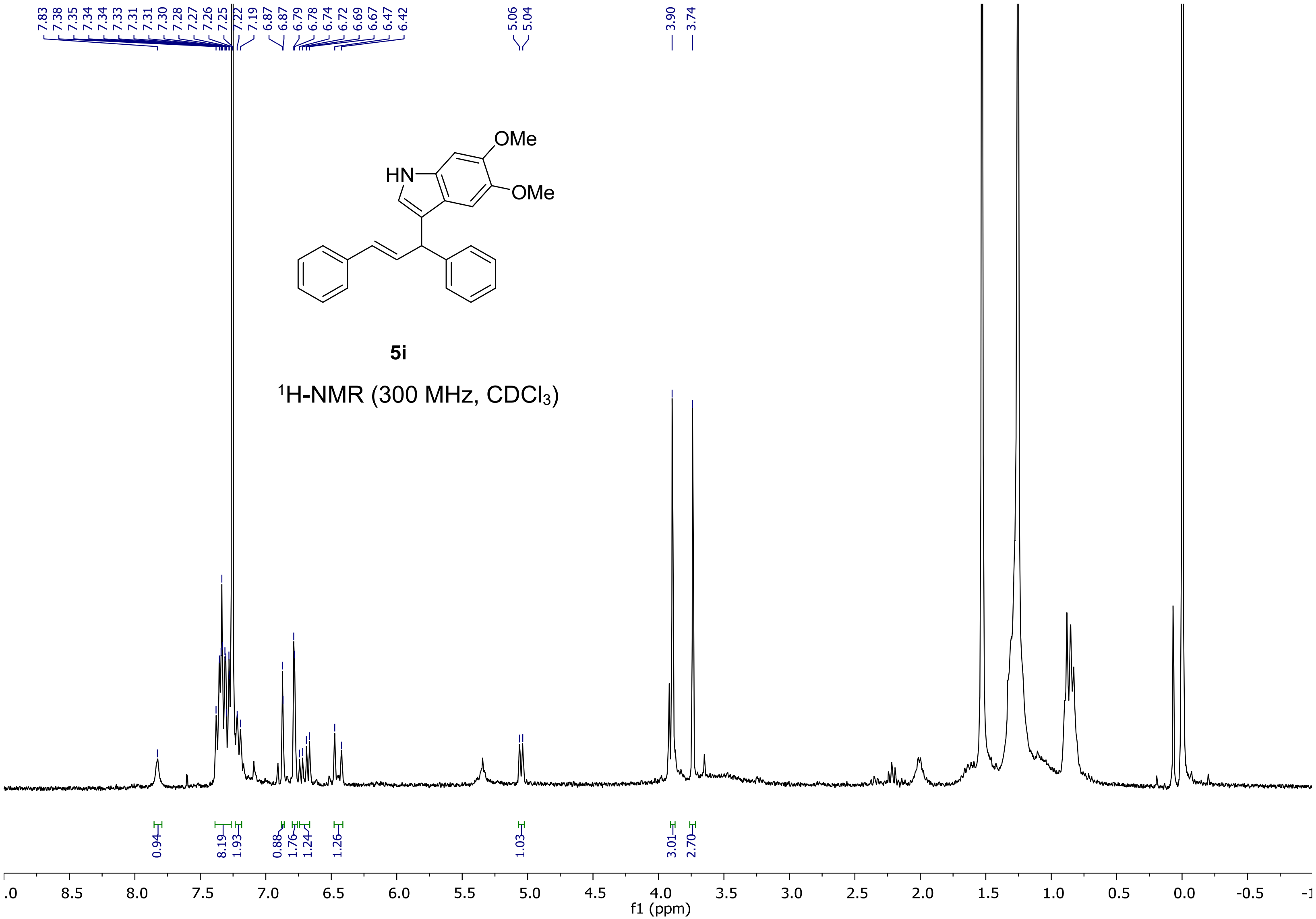

Supplementary Figure 95. ^{1}H-NMR spectra for compound **5i**

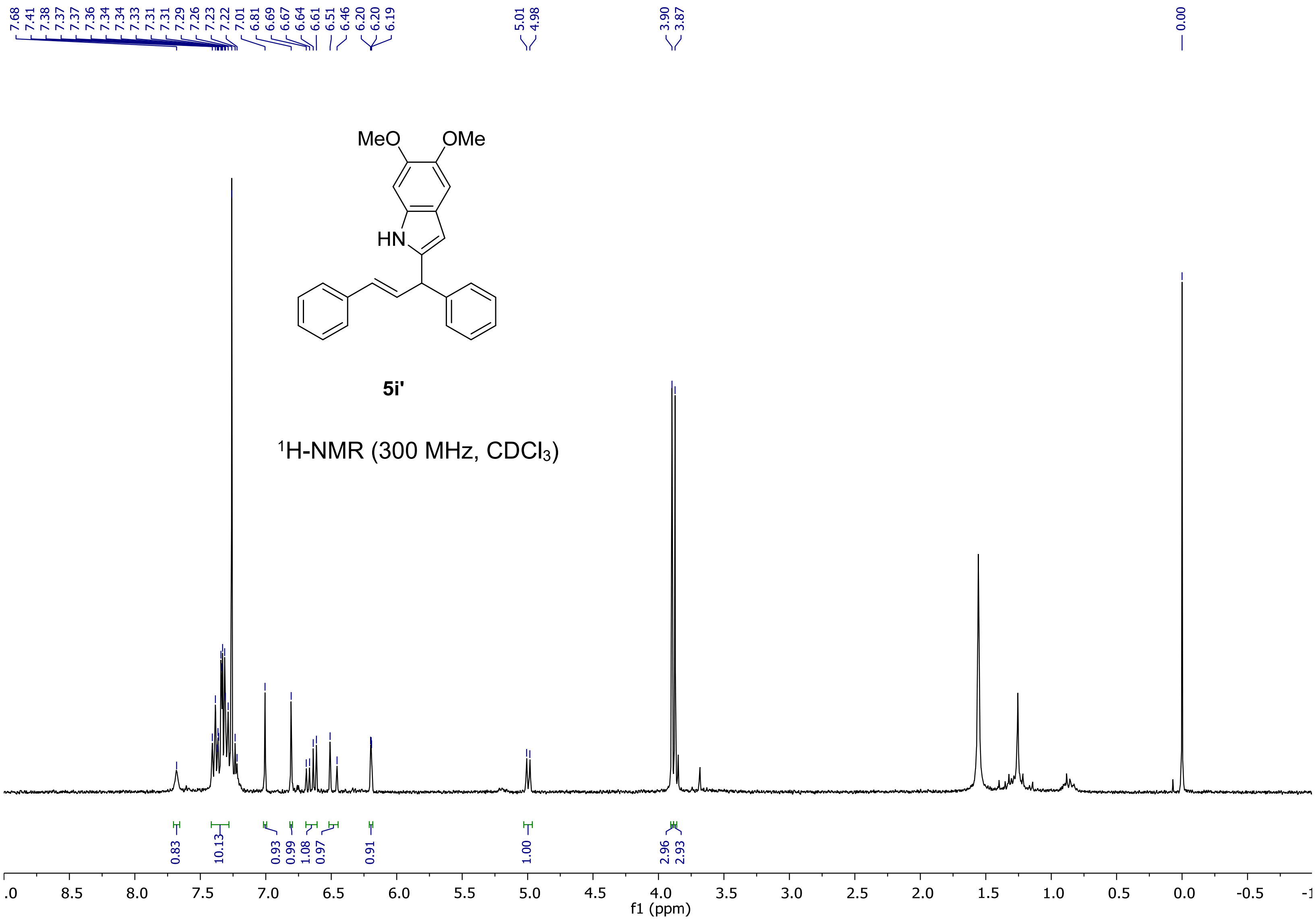

Supplementary Figure 96. ^{1}H-NMR spectra for compound **5i'**

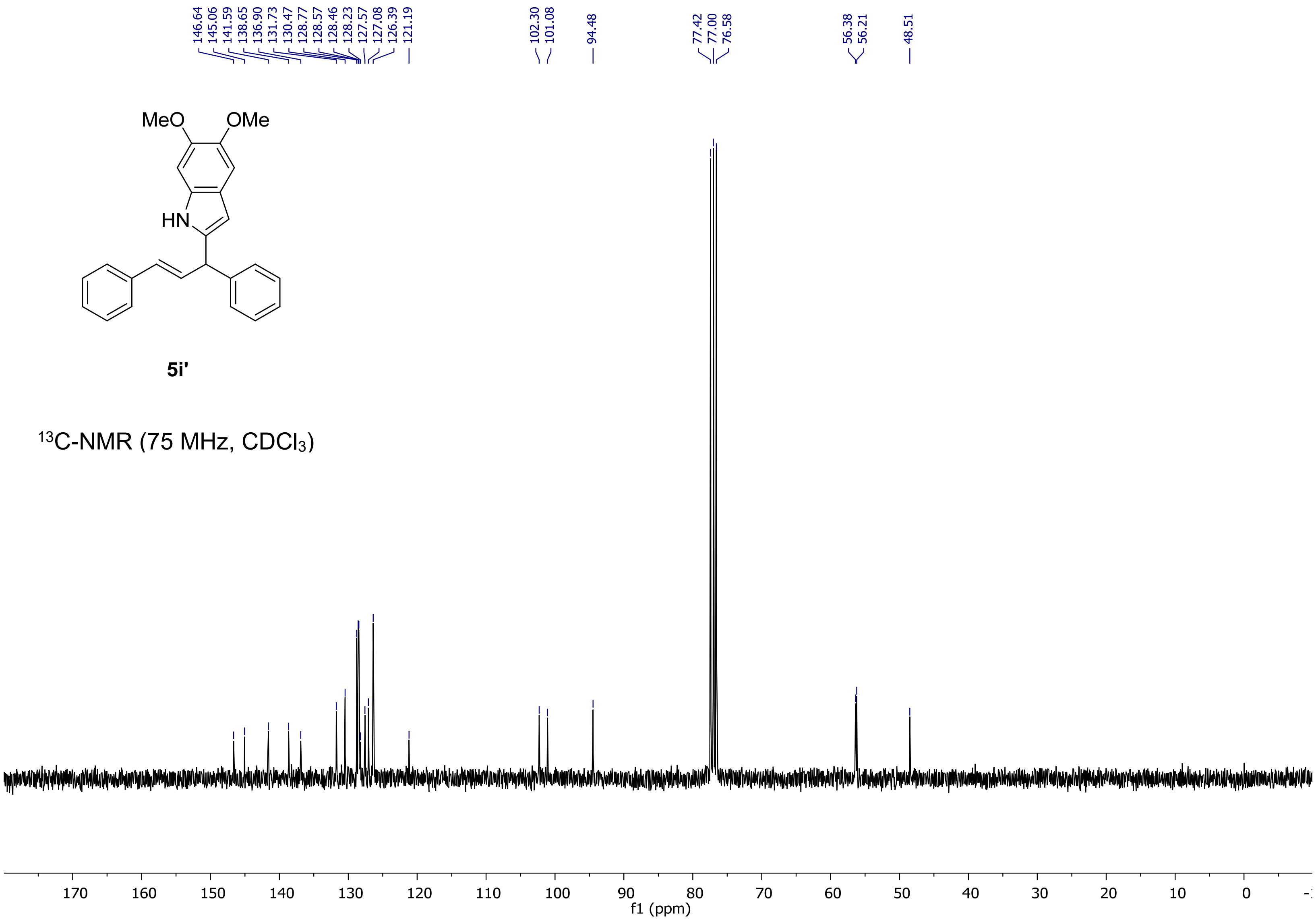

Supplementary Figure 97. ^{13}C-NMR spectra for compound **5i'**

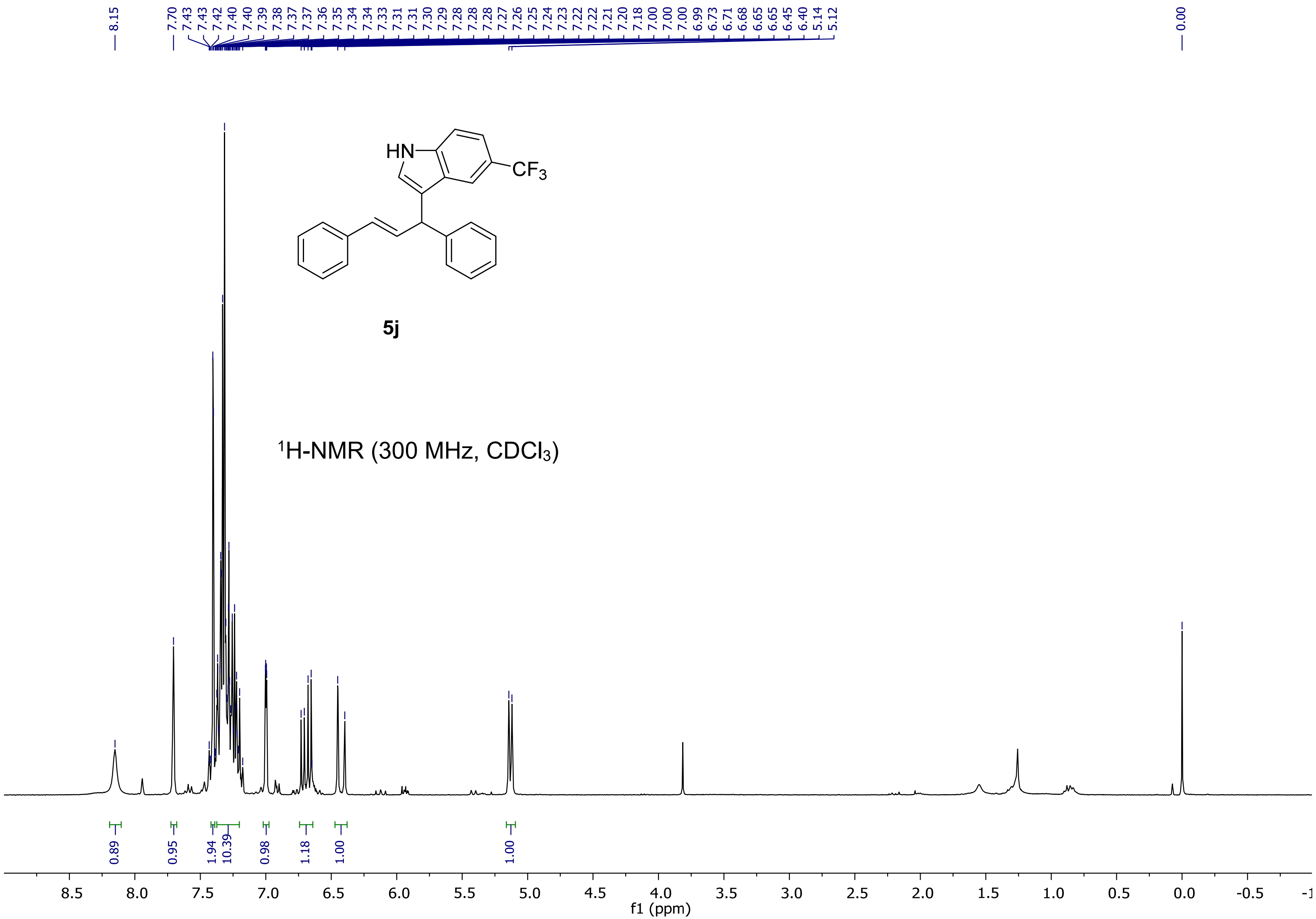

Supplementary Figure 98. ^{1}H-NMR spectra for compound **5j**

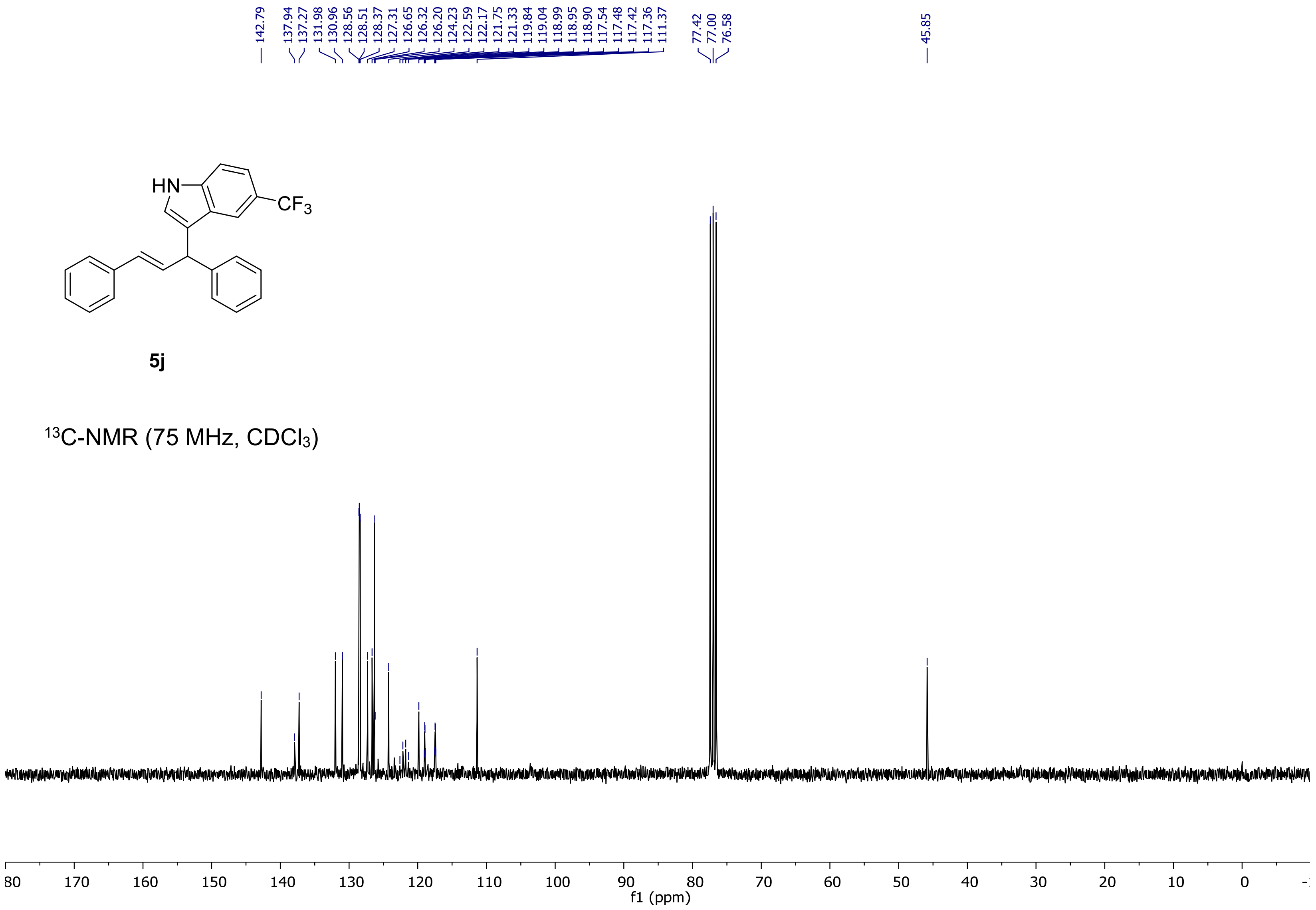

Supplementary Figure 99. ^{13}C-NMR spectra for compound **5j**

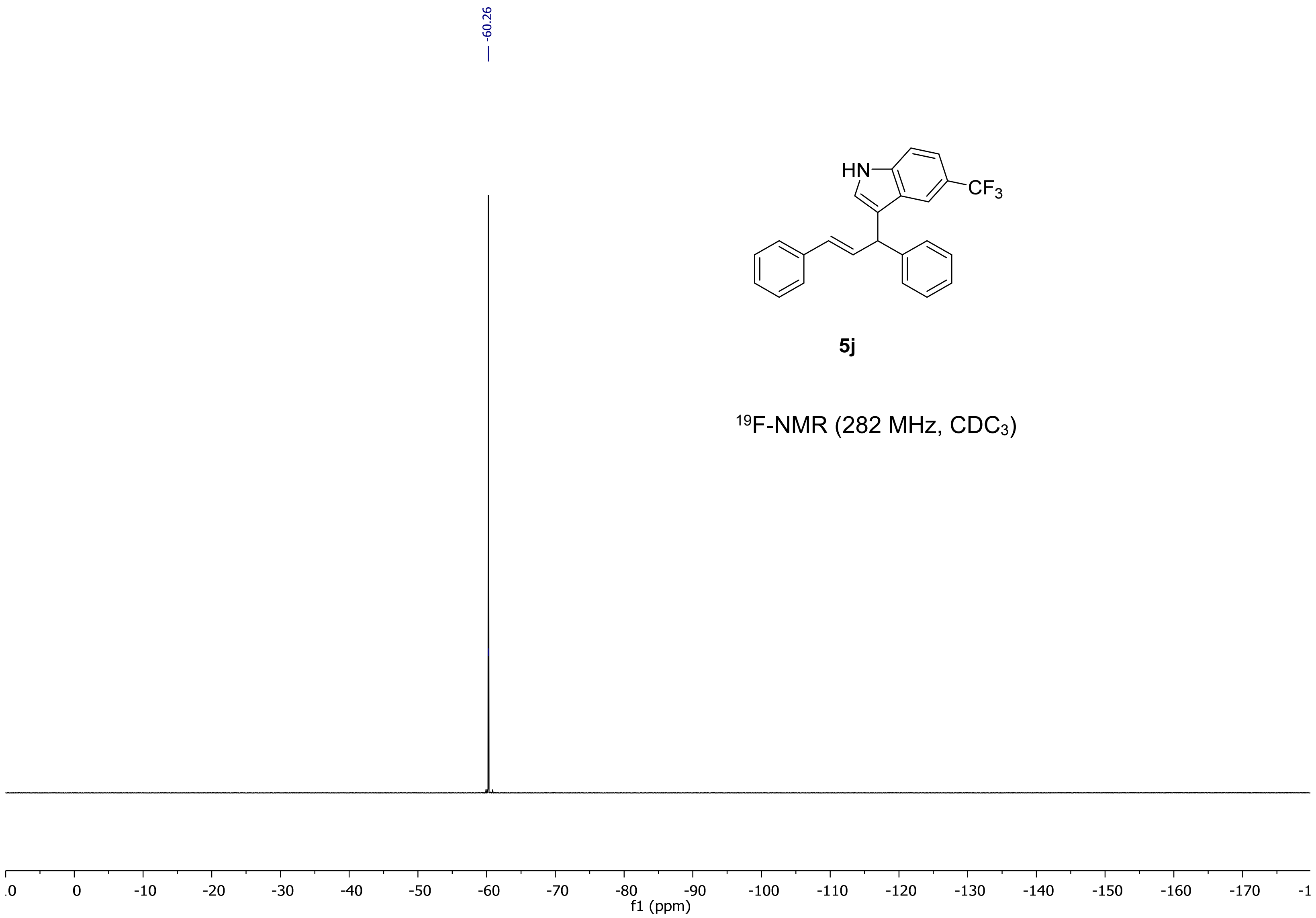

Supplementary Figure 100. ^{19}F-NMR spectra for compound **5j**

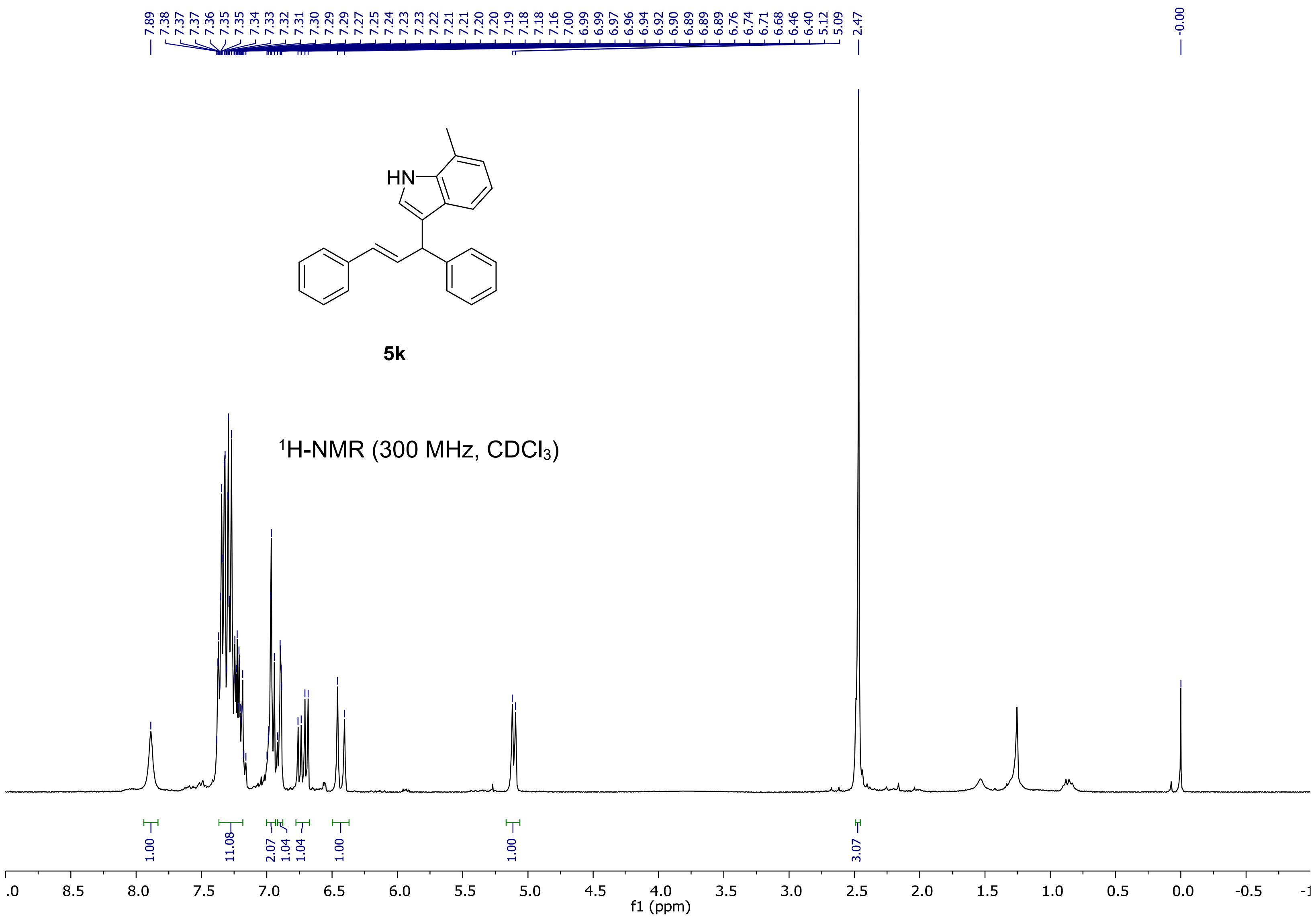

Supplementary Figure 101. ^{1}H-NMR spectra for compound **5k**

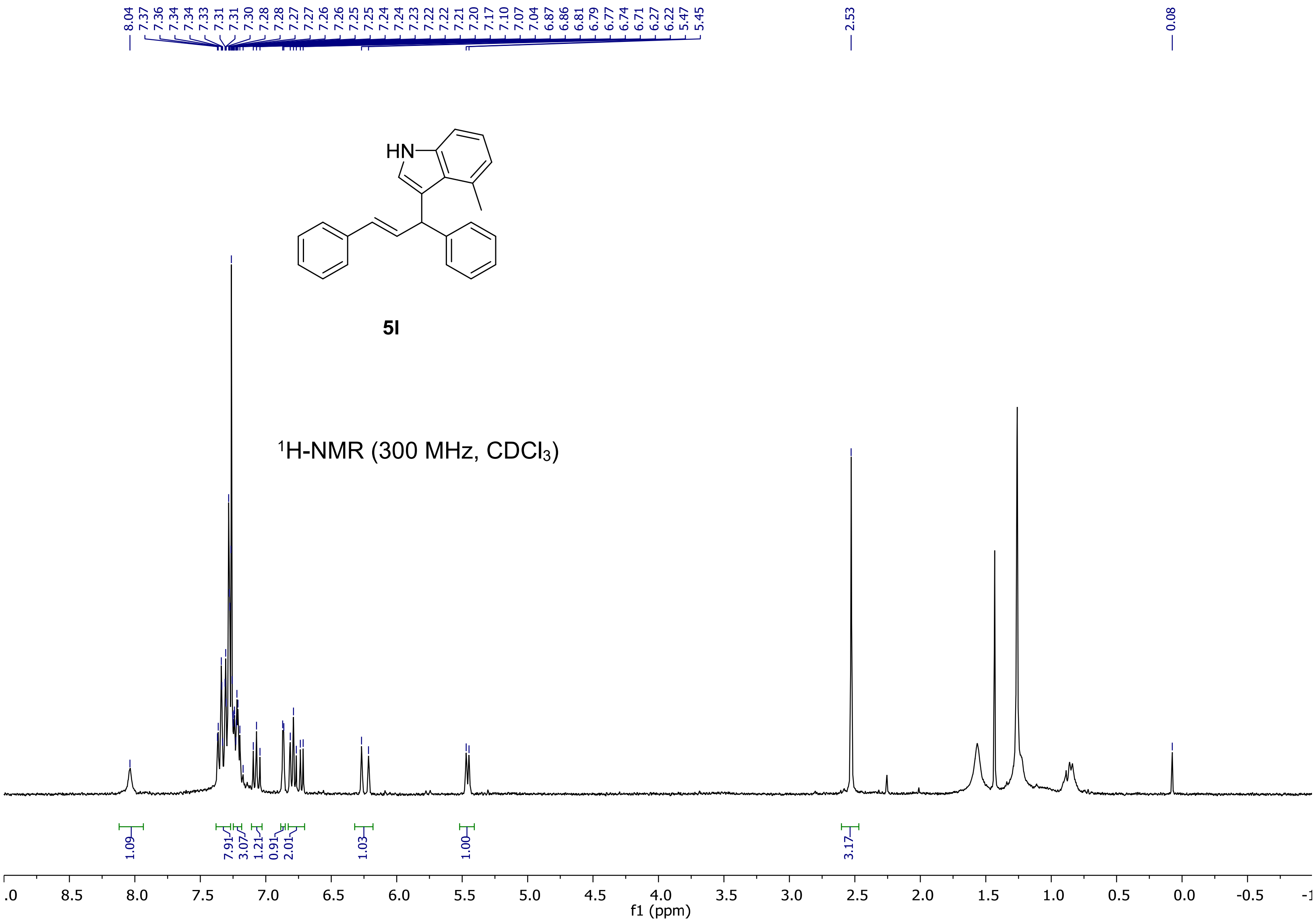

Supplementary Figure 102. ^{1}H-NMR spectra for compound **5l**

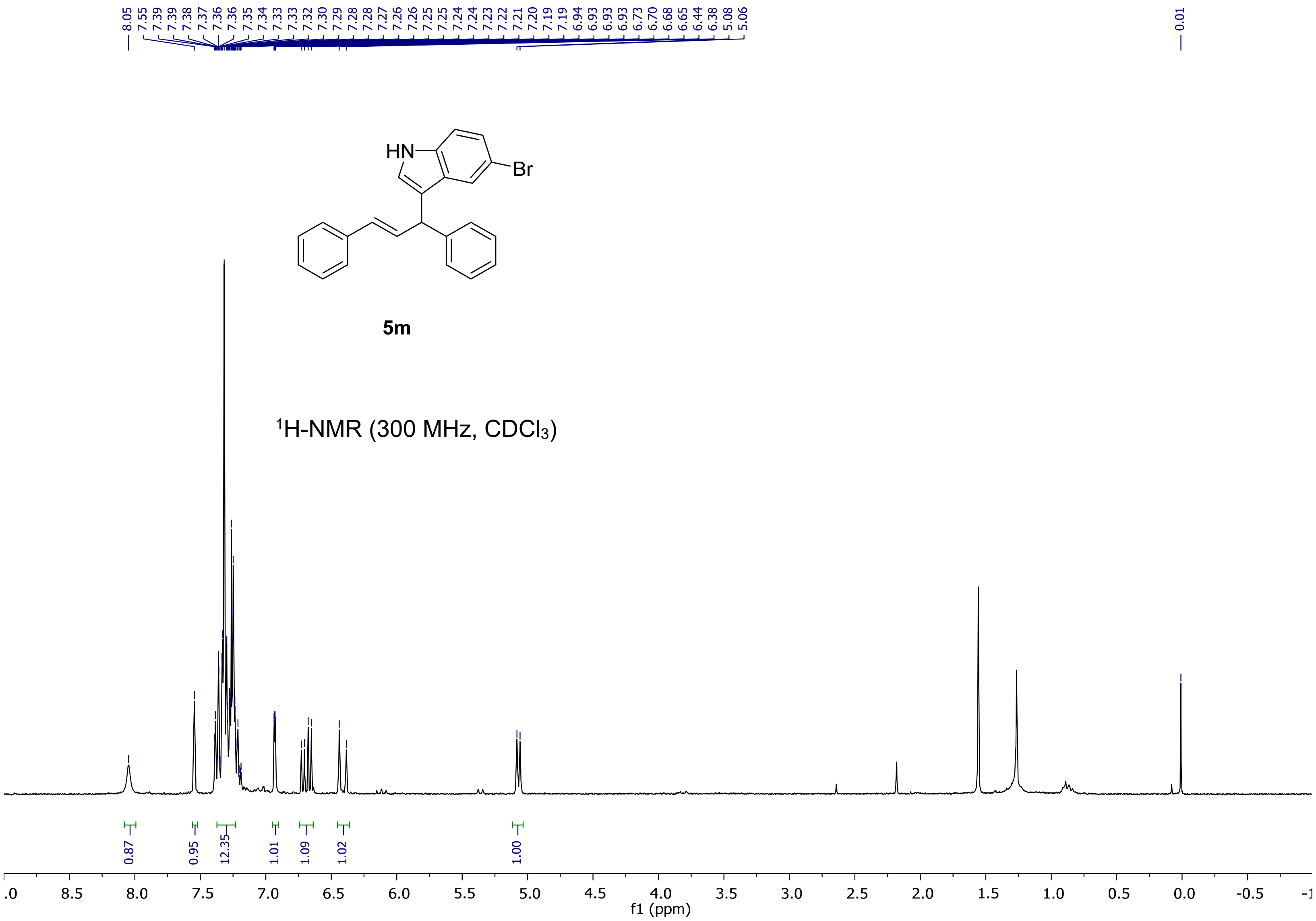

Supplementary Figure 103. ^{1}H-NMR spectra for compound **5m**

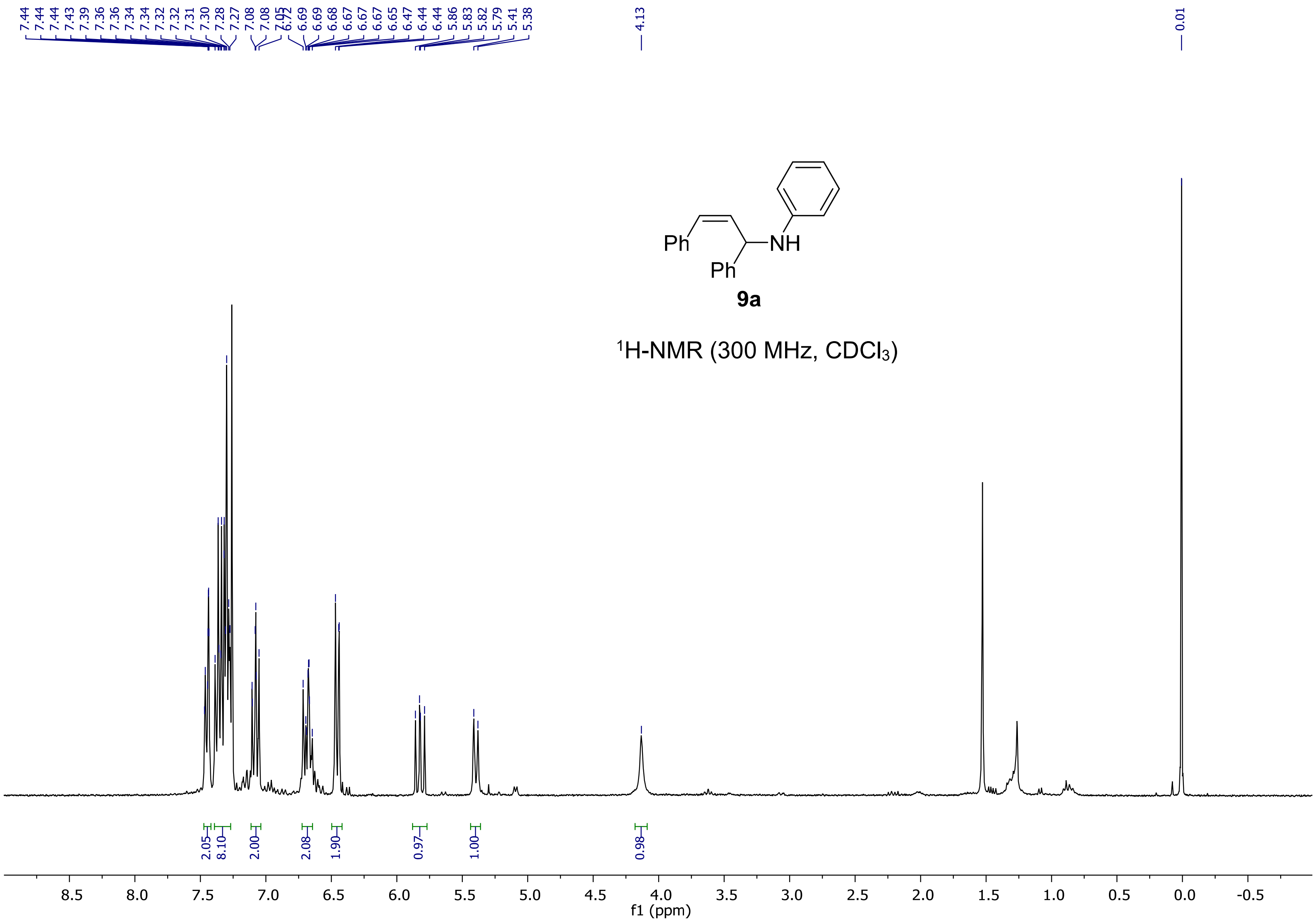

Supplementary Figure 104. ^{1}H-NMR spectra for compound **9a**

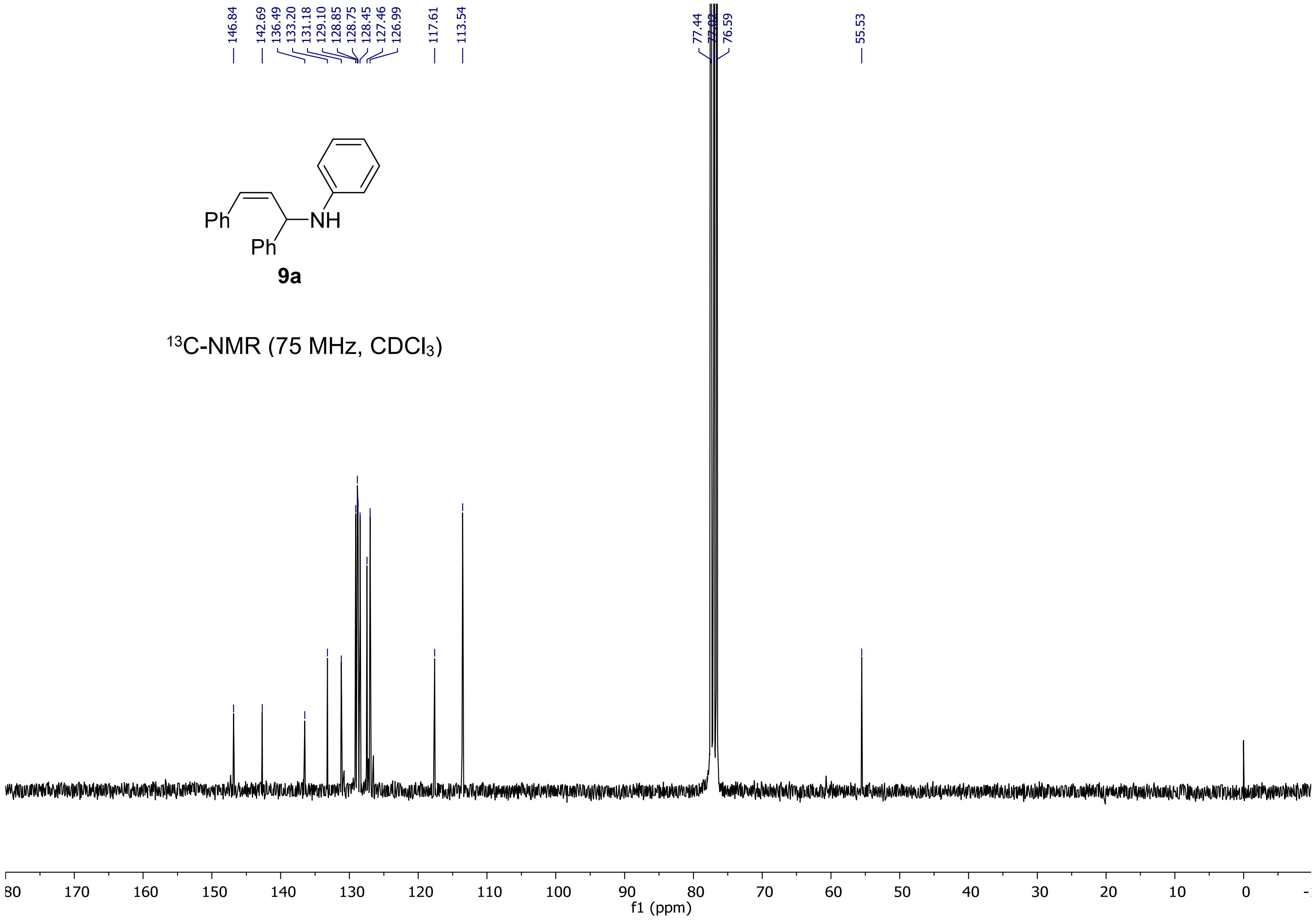

Supplementary Figure 105. ^{13}C-NMR spectra for compound **9a**

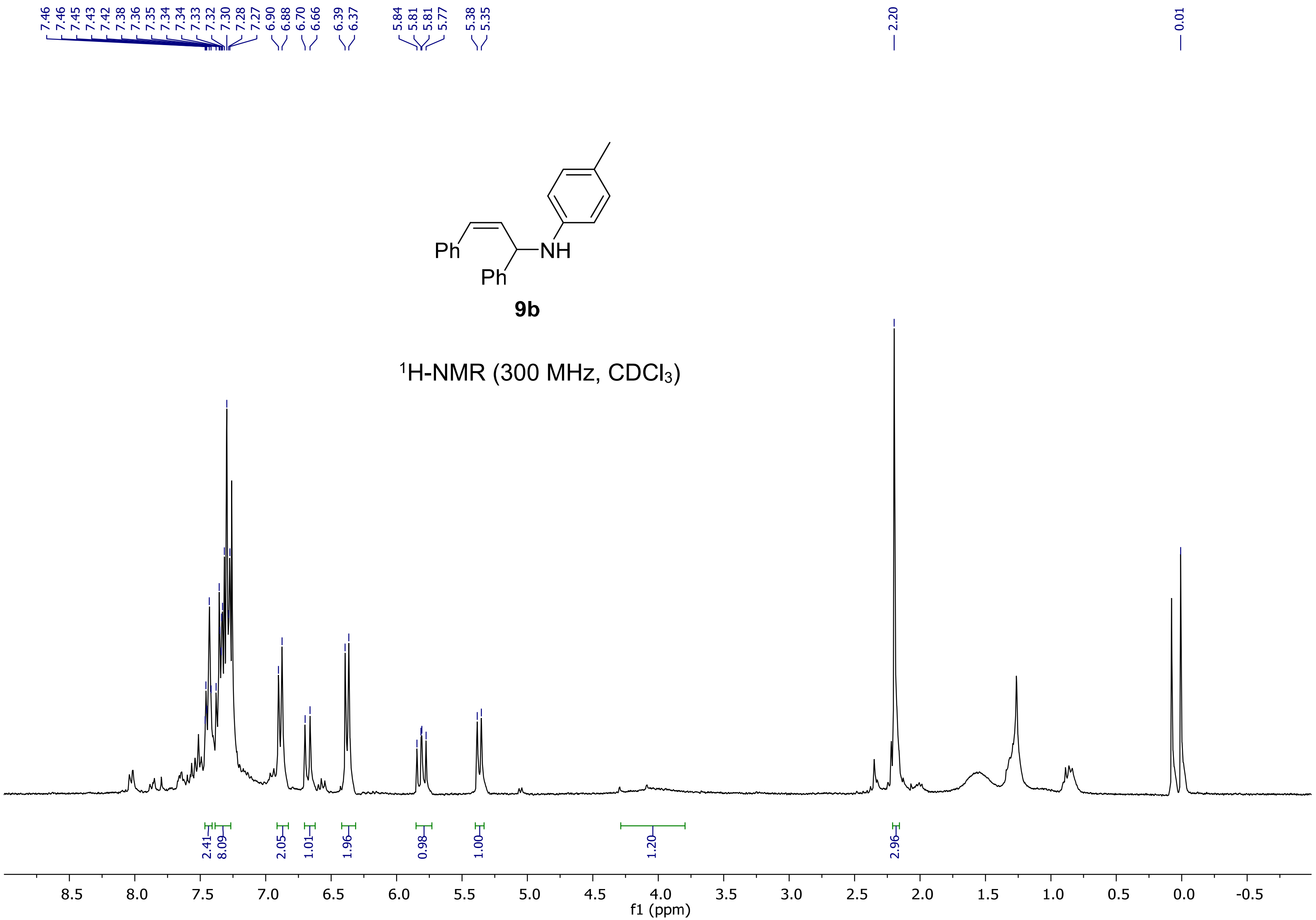

Supplementary Figure 106. ^{1}H-NMR spectra for compound **9b**

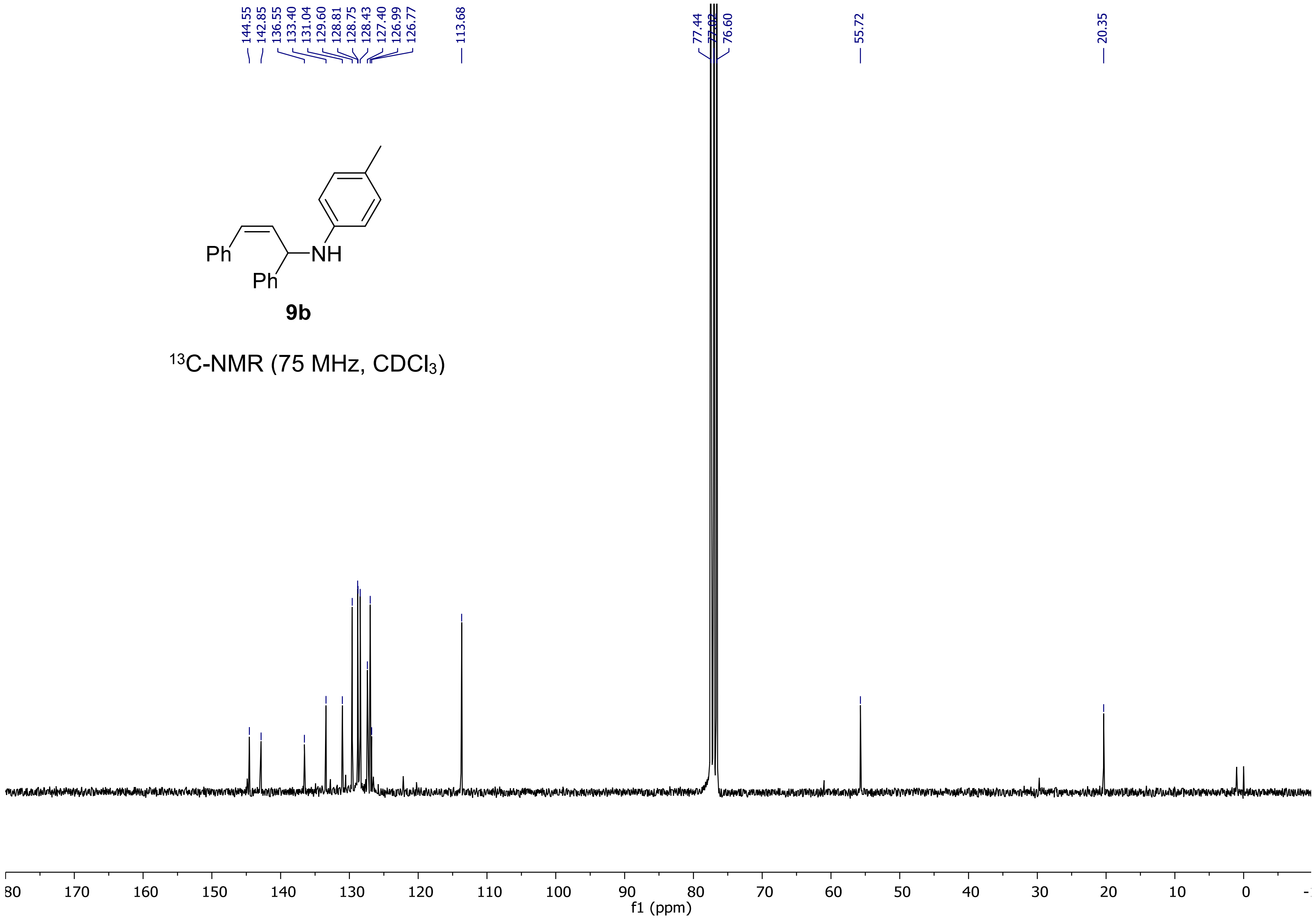

Supplementary Figure 107. ^{13}C-NMR spectra for compound **9b**

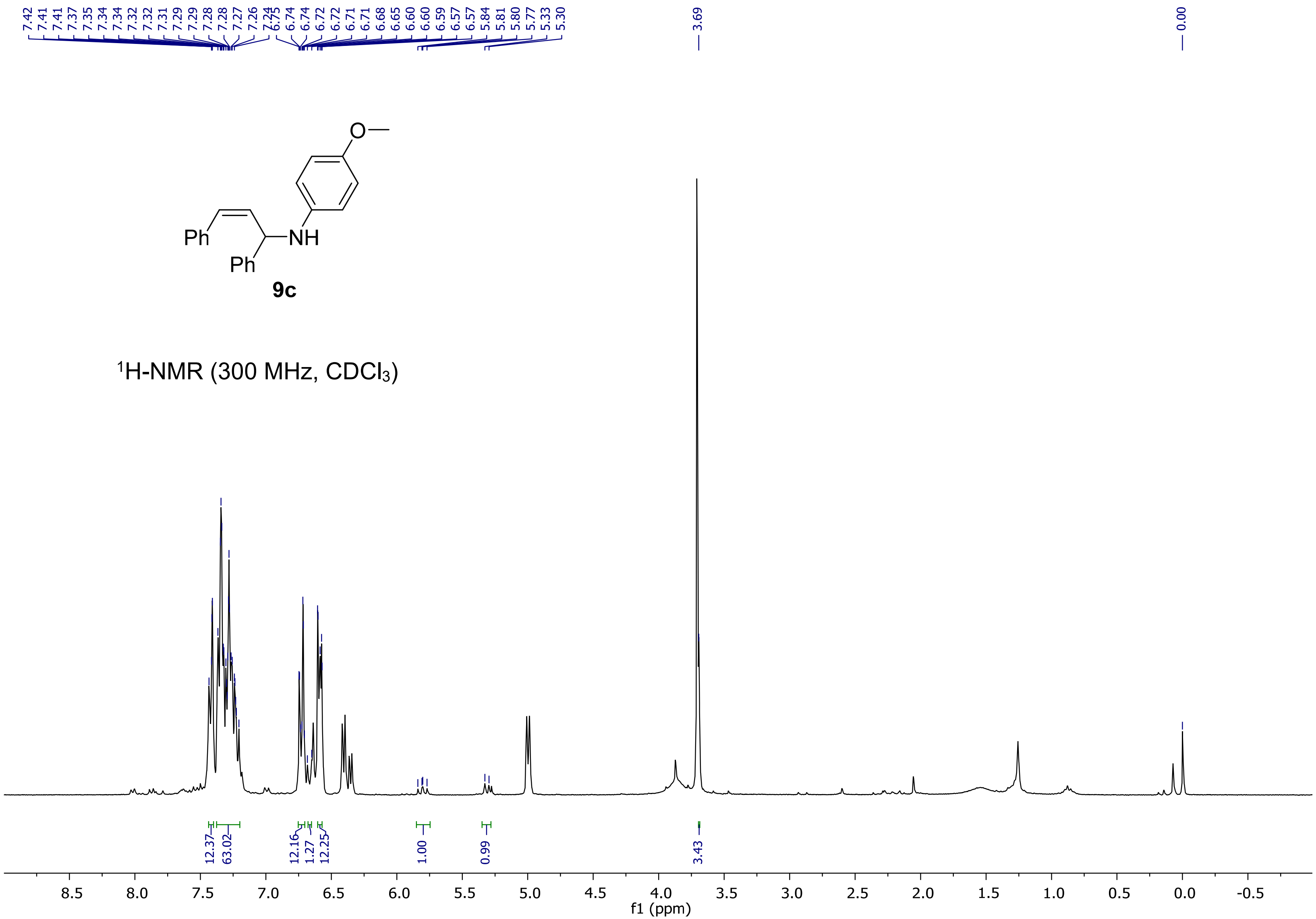

Supplementary Figure 108. ^{1}H-NMR spectra for compound **9c**

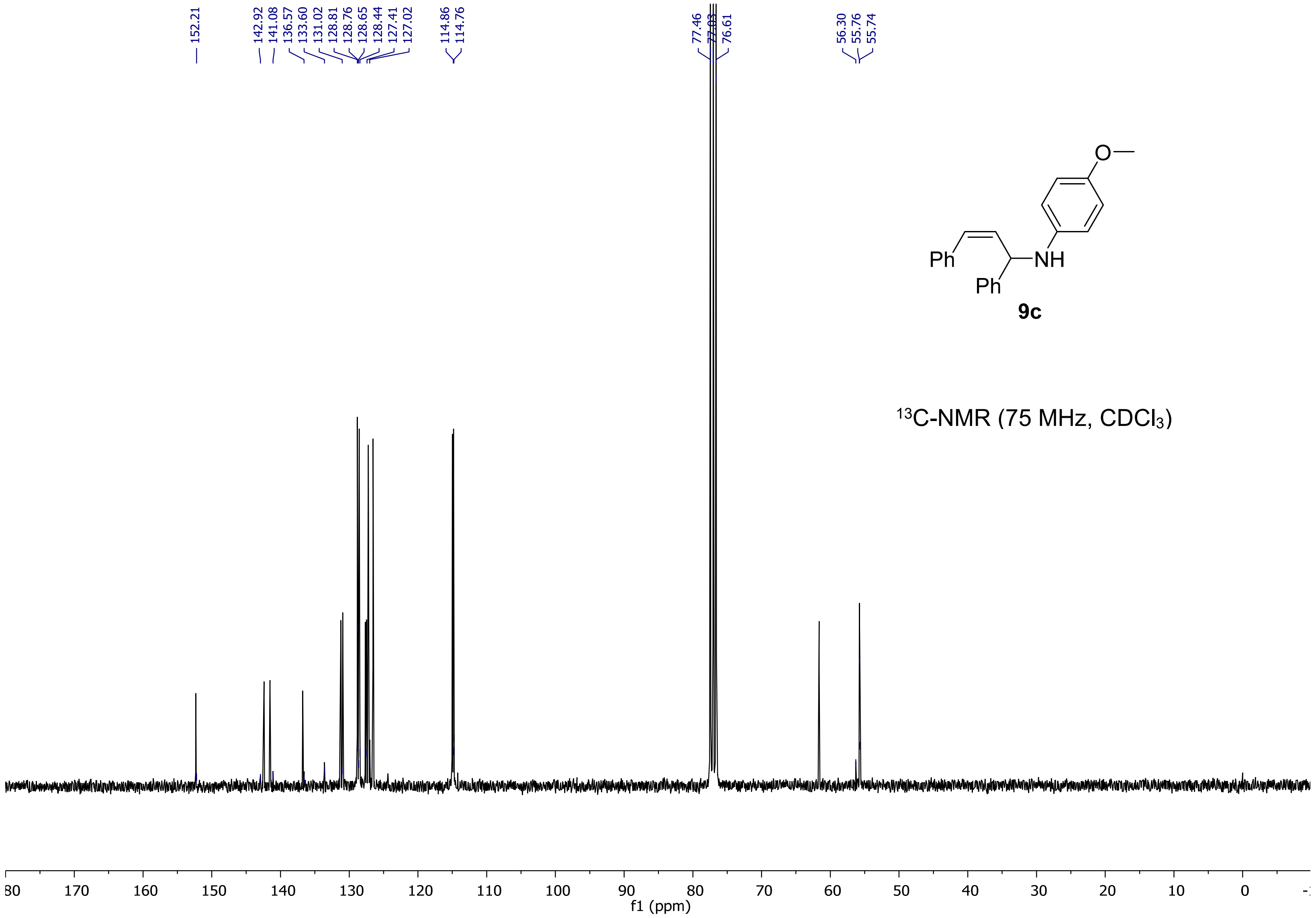

Supplementary Figure 109. ^{13}C-NMR spectra for compound **9c**

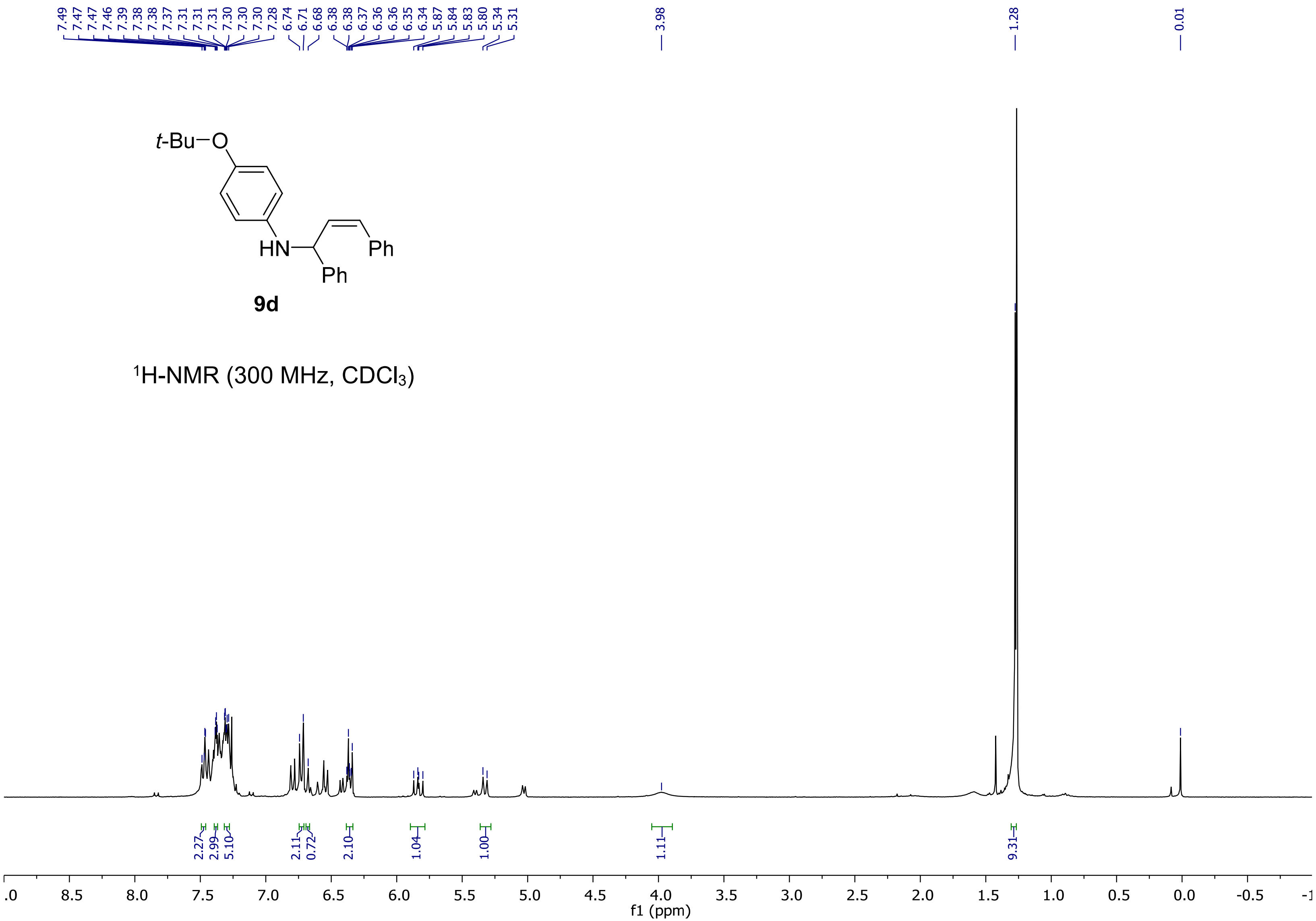

Supplementary Figure 110. ^{1}H-NMR spectra for compound **9d**

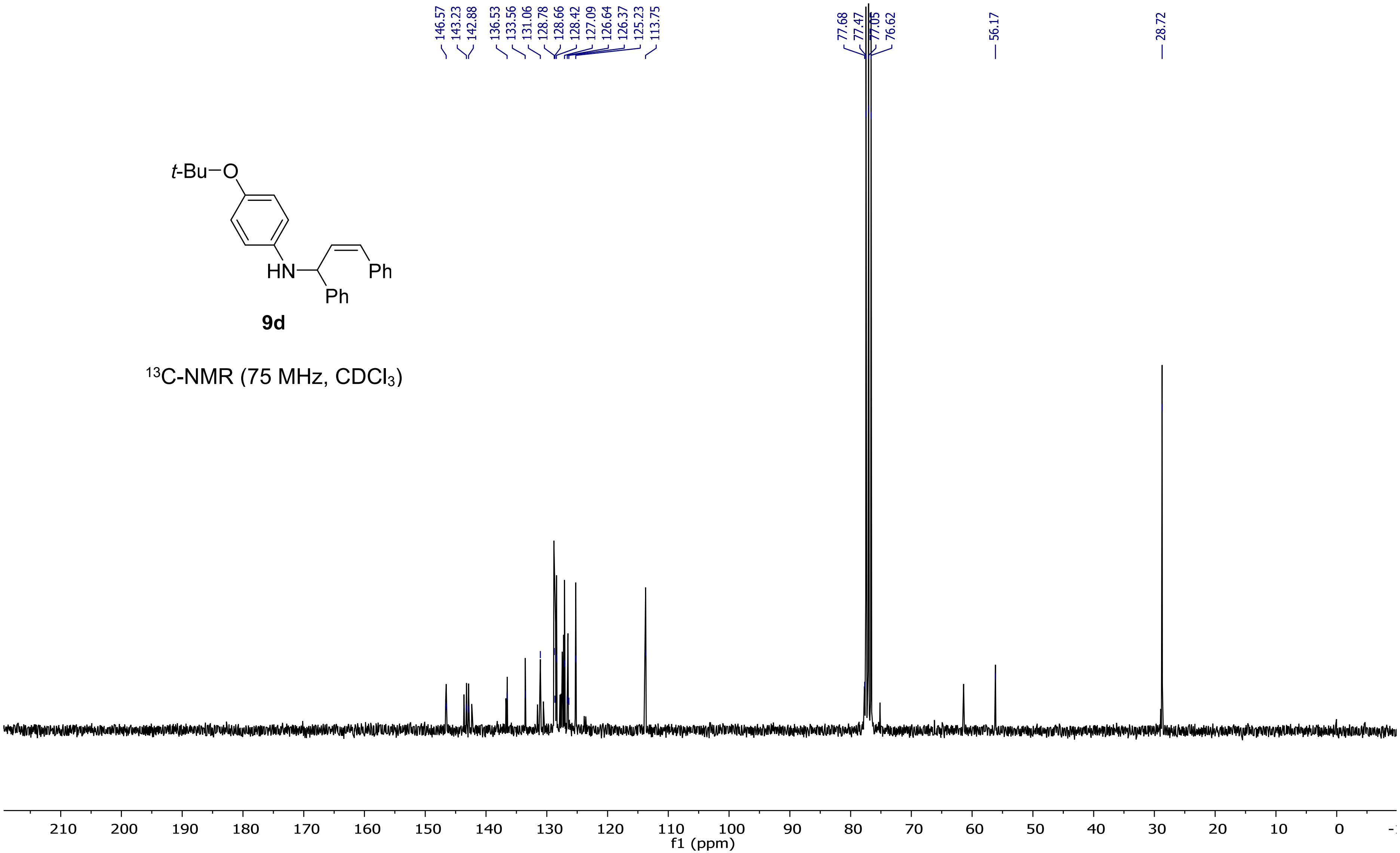

Supplementary Figure 111. ^{13}C-NMR spectra for compound **9d**

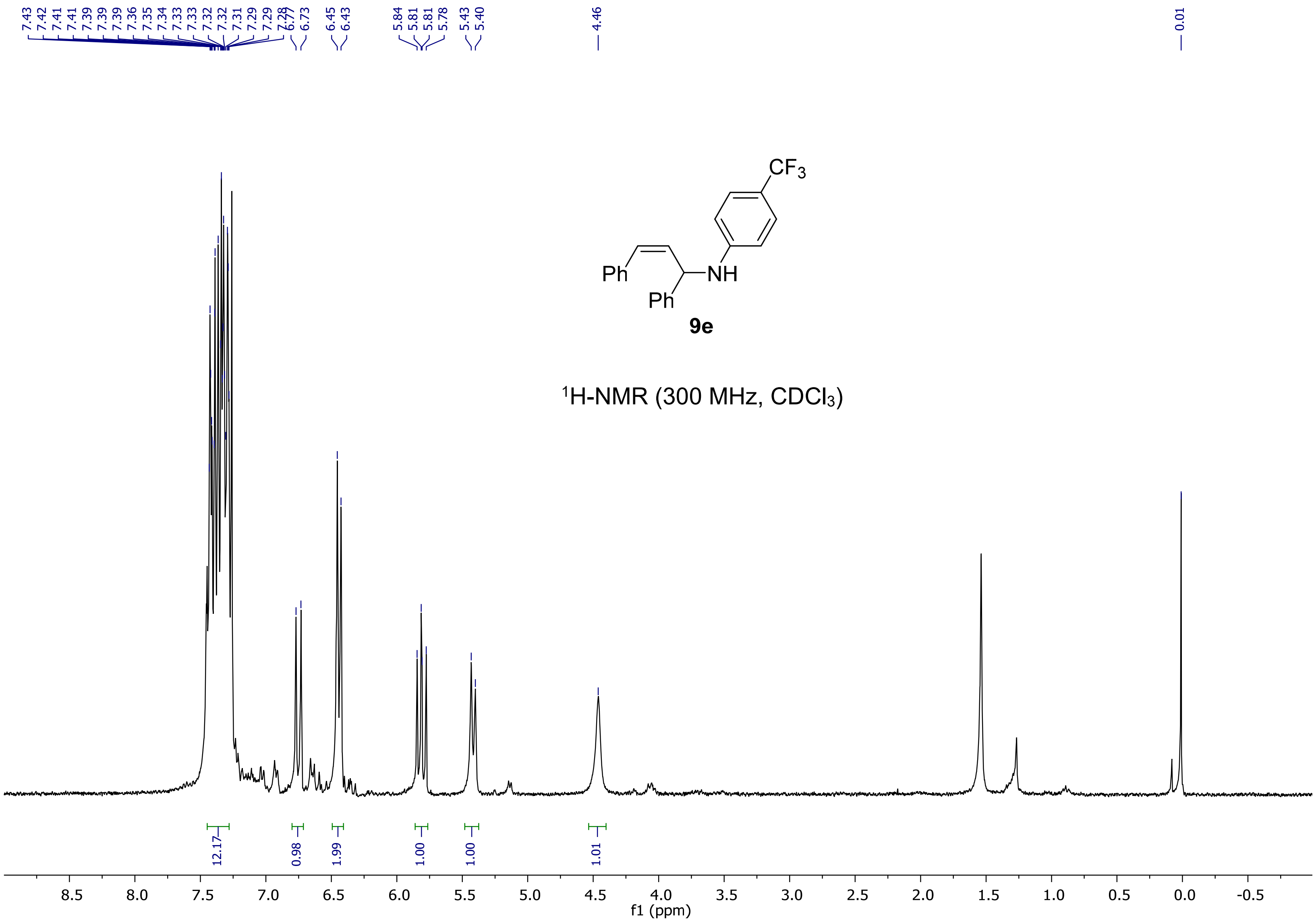

Supplementary Figure 112. ^{1}H-NMR spectra for compound **9e**

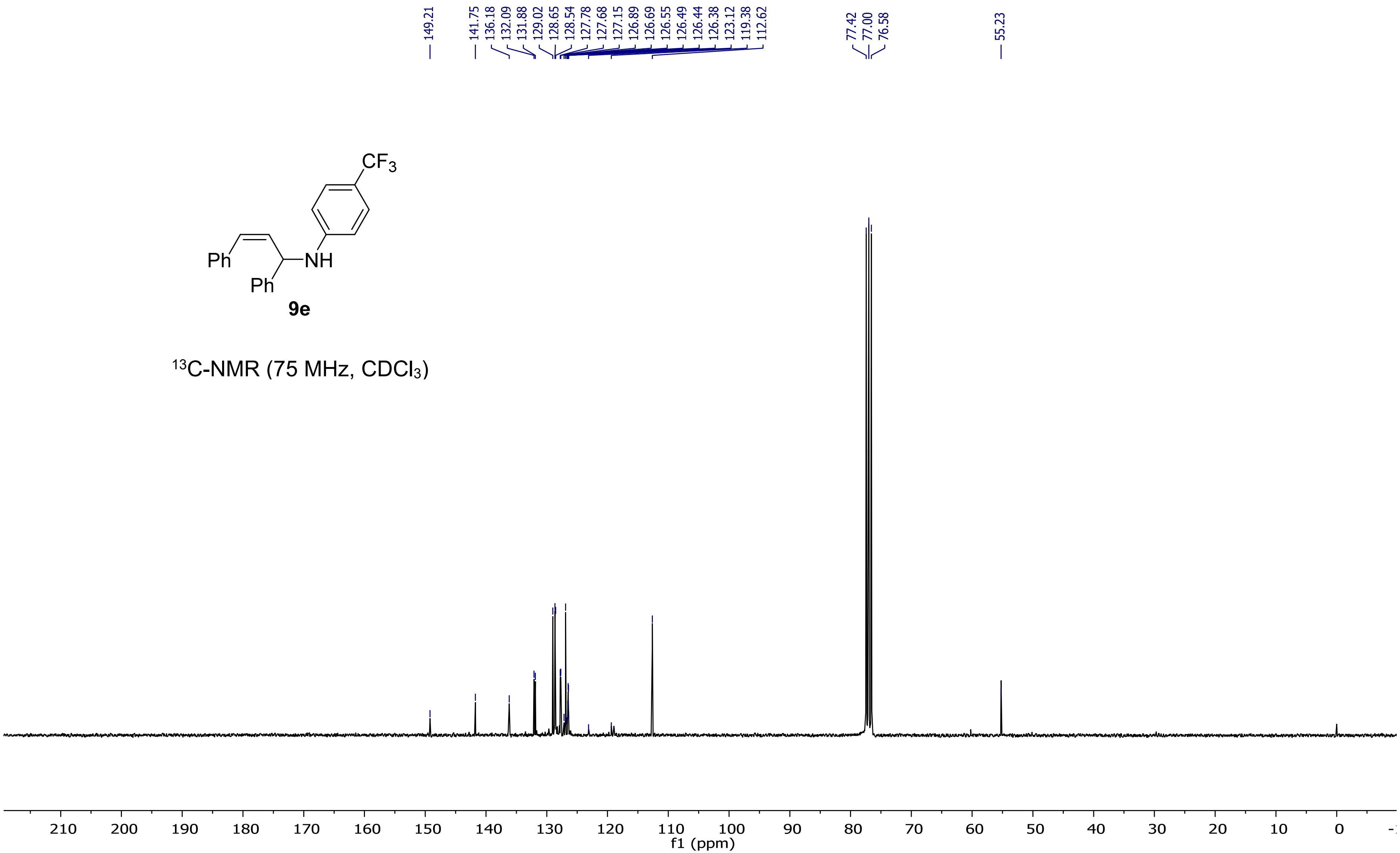

Supplementary Figure 113. ^{13}C-NMR spectra for compound **9e**

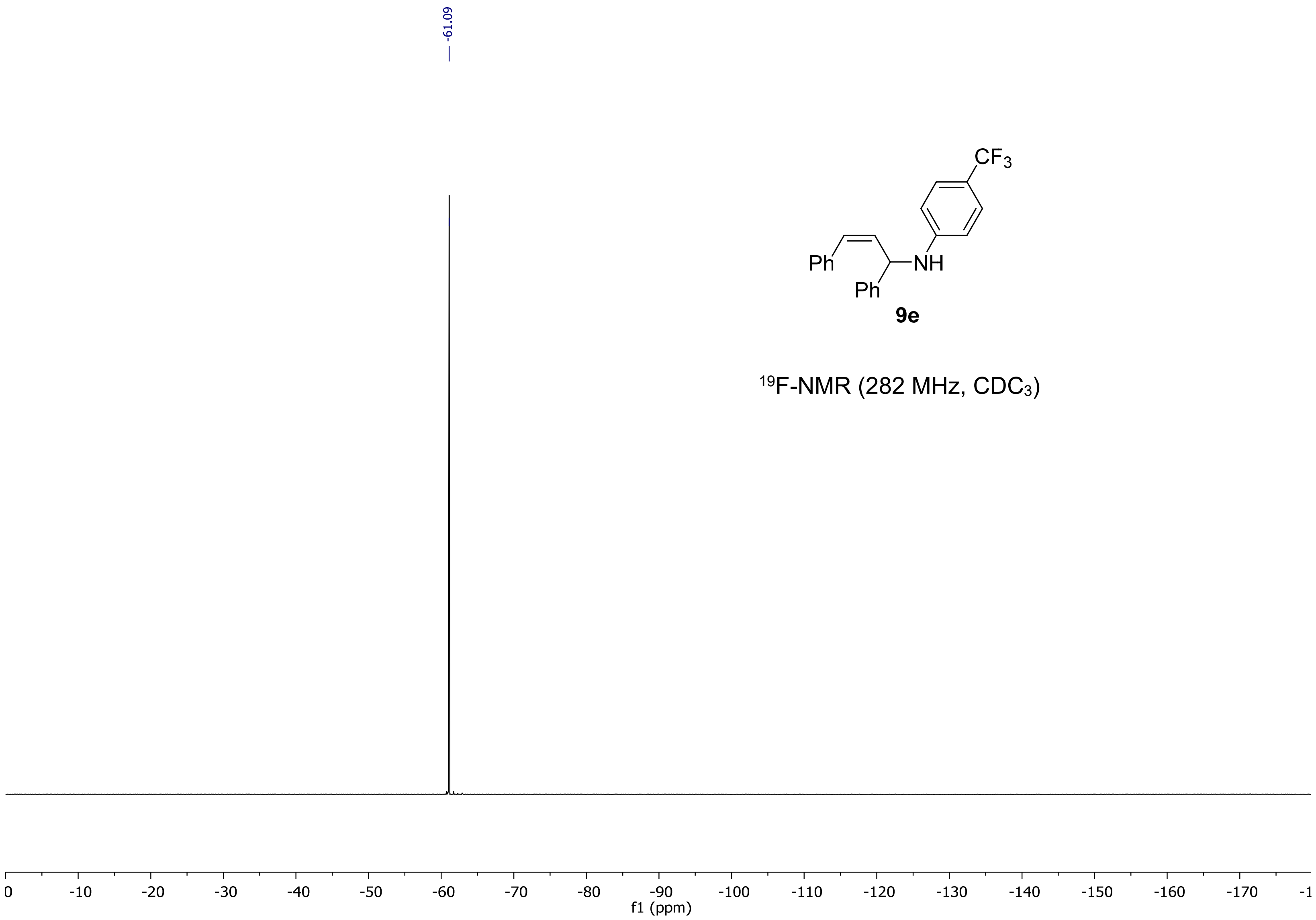

Supplementary Figure 114. ^{19}F-NMR spectra for compound **9e**

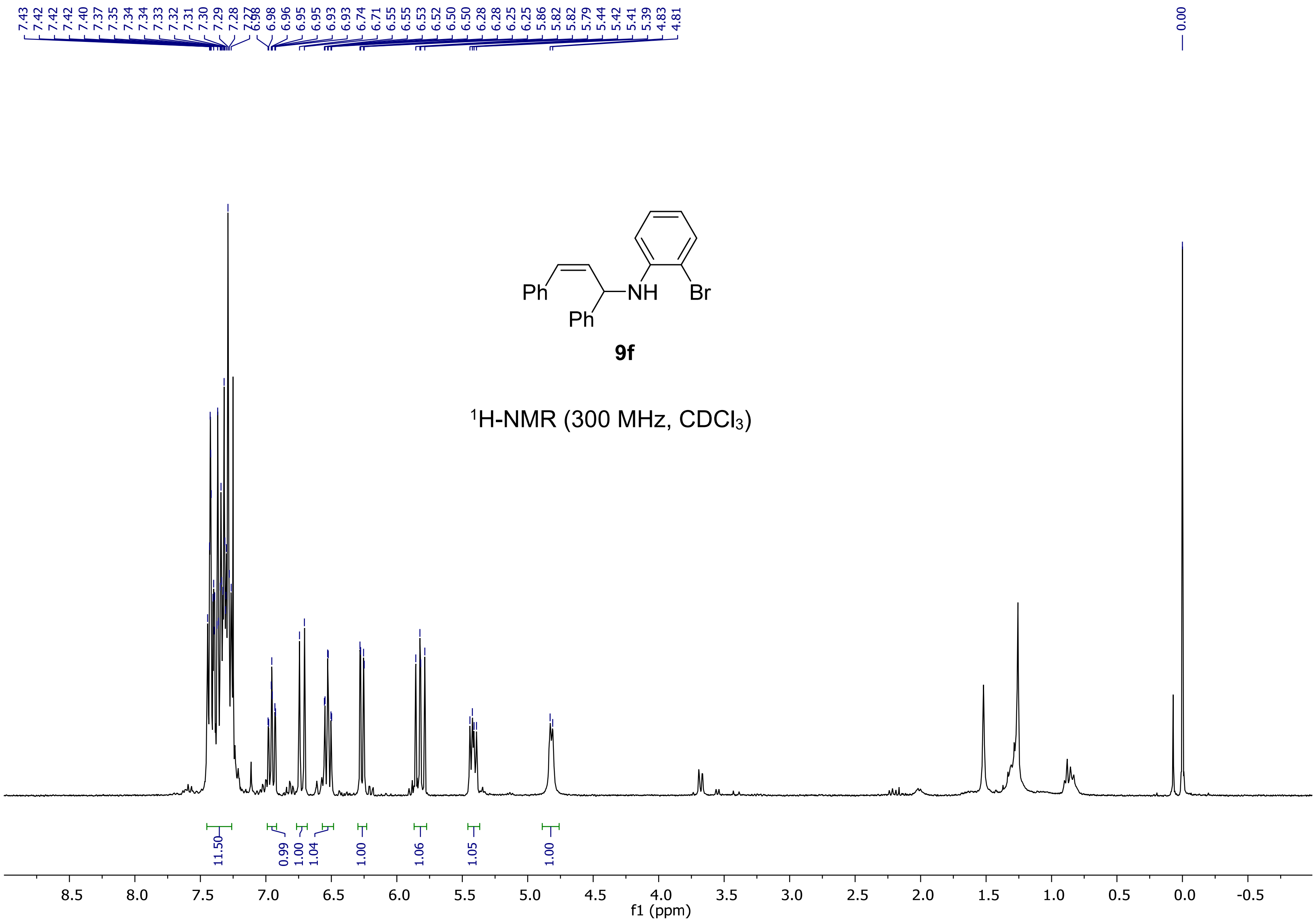

Supplementary Figure 115. ^{1}H-NMR spectra for compound **9f**

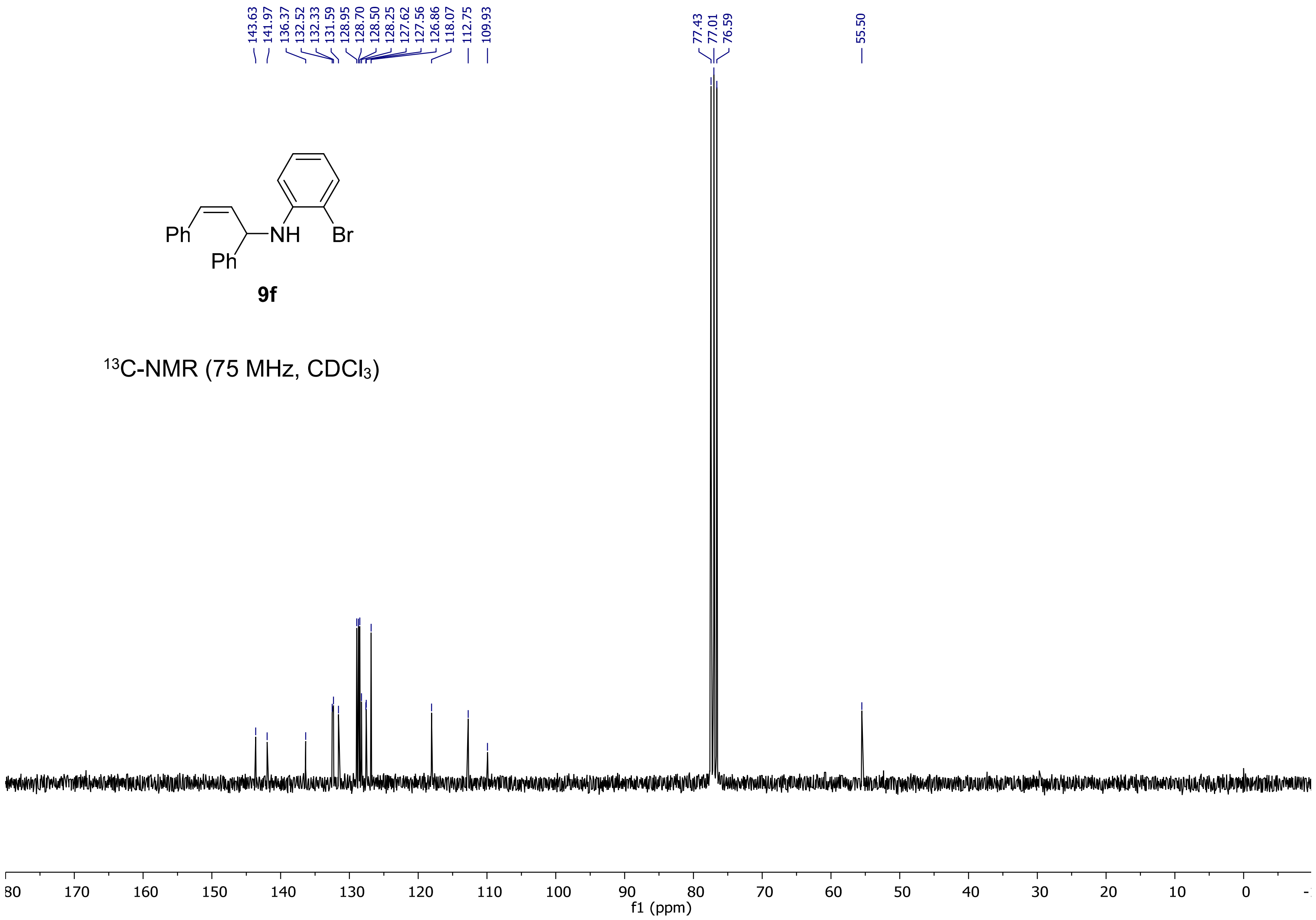

Supplementary Figure 116. ^{13}C-NMR spectra for compound **9f**

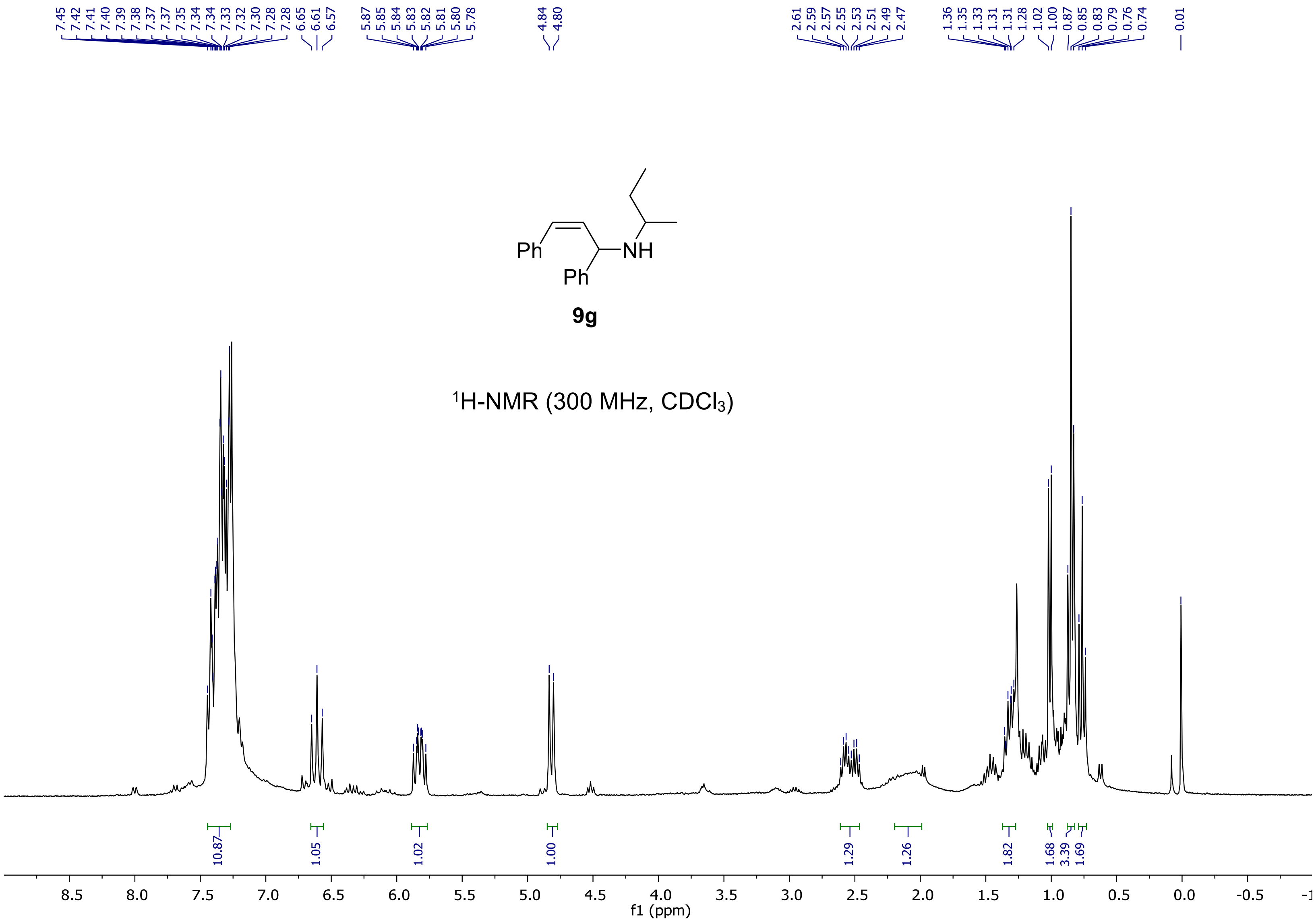

Supplementary Figure 117. ¹H-NMR spectra for compound **9g**

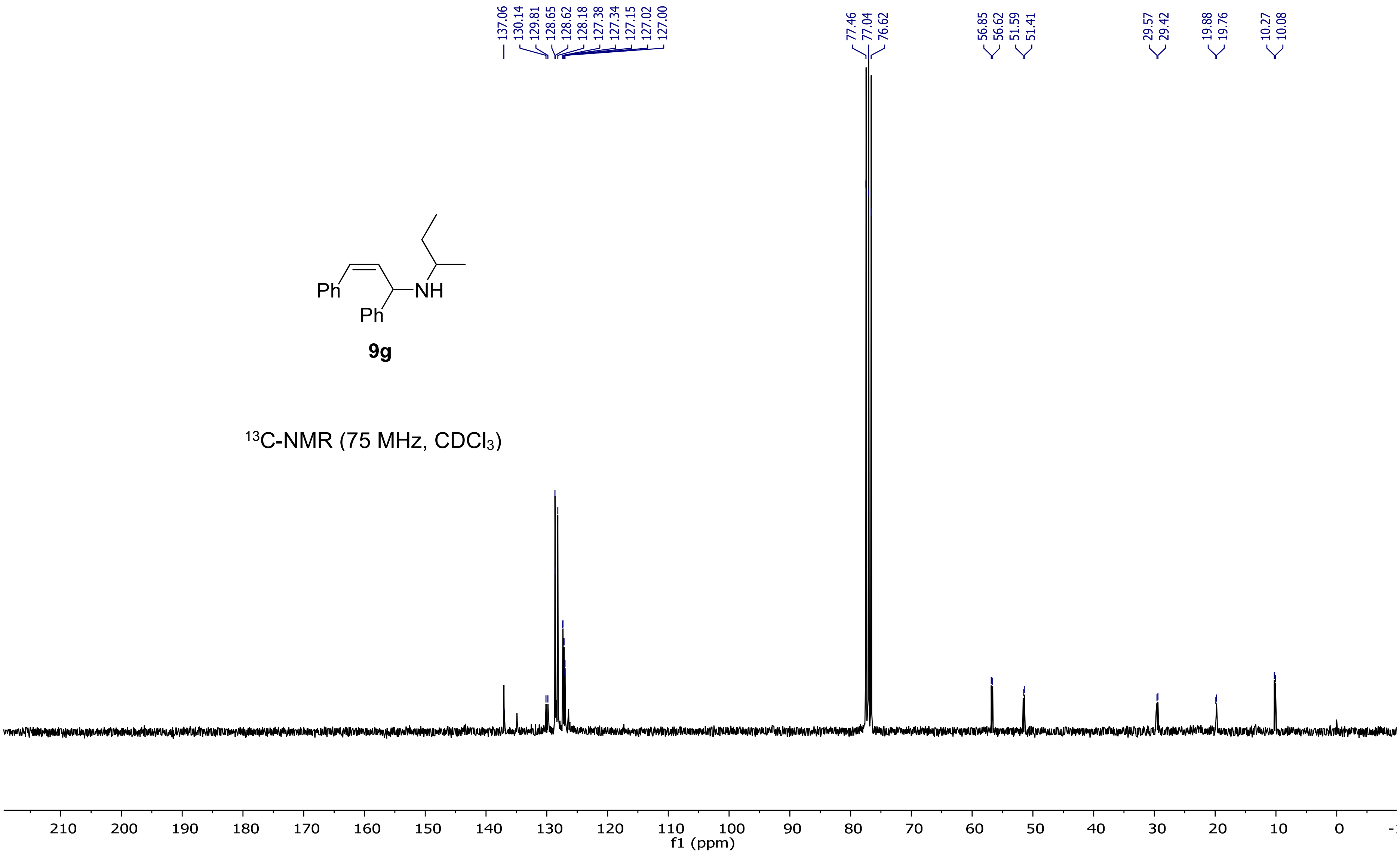

Supplementary Figure 118. ^{13}C-NMR spectra for compound **9g**

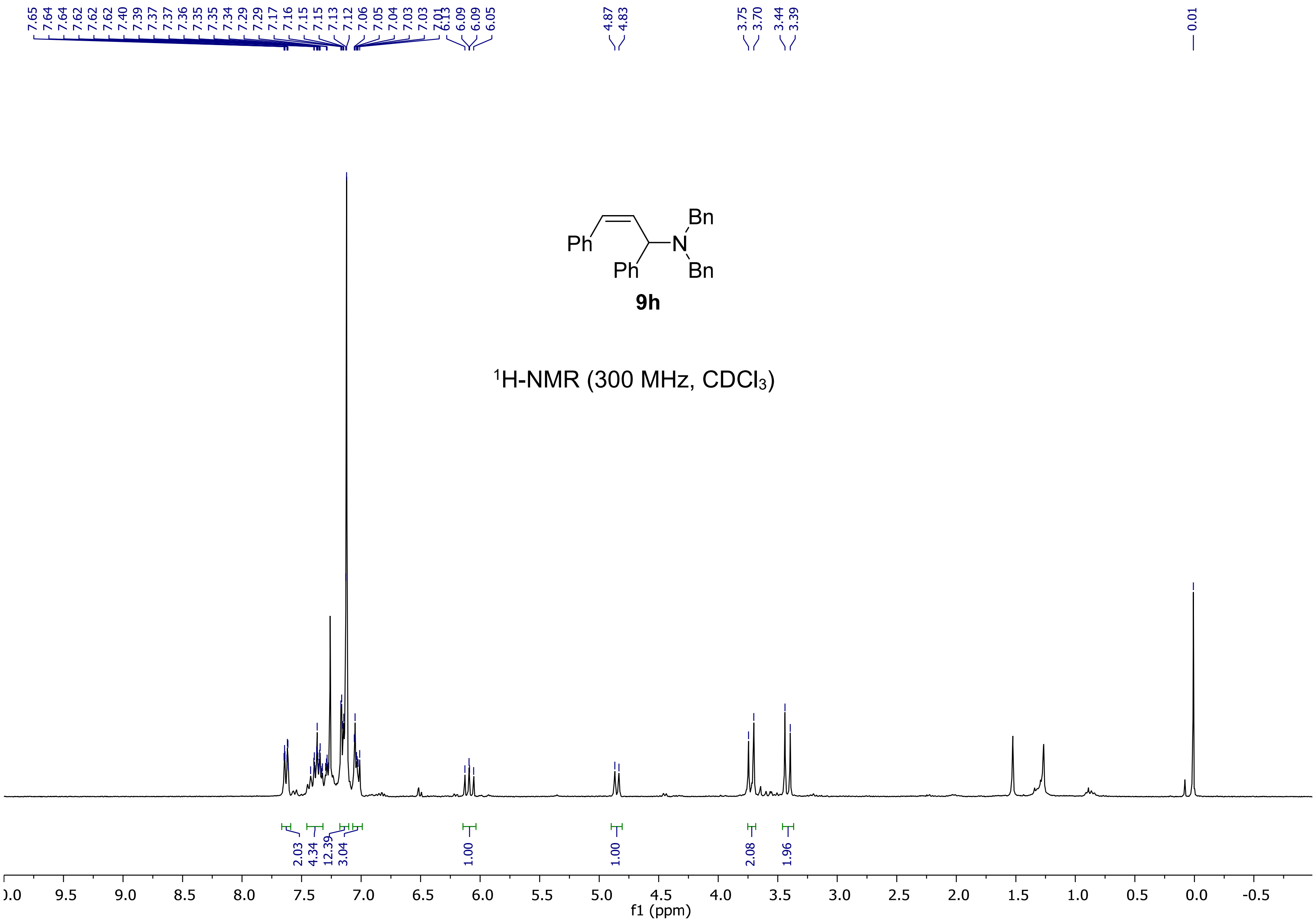

Supplementary Figure 119. ^{1}H-NMR spectra for compound **9h**

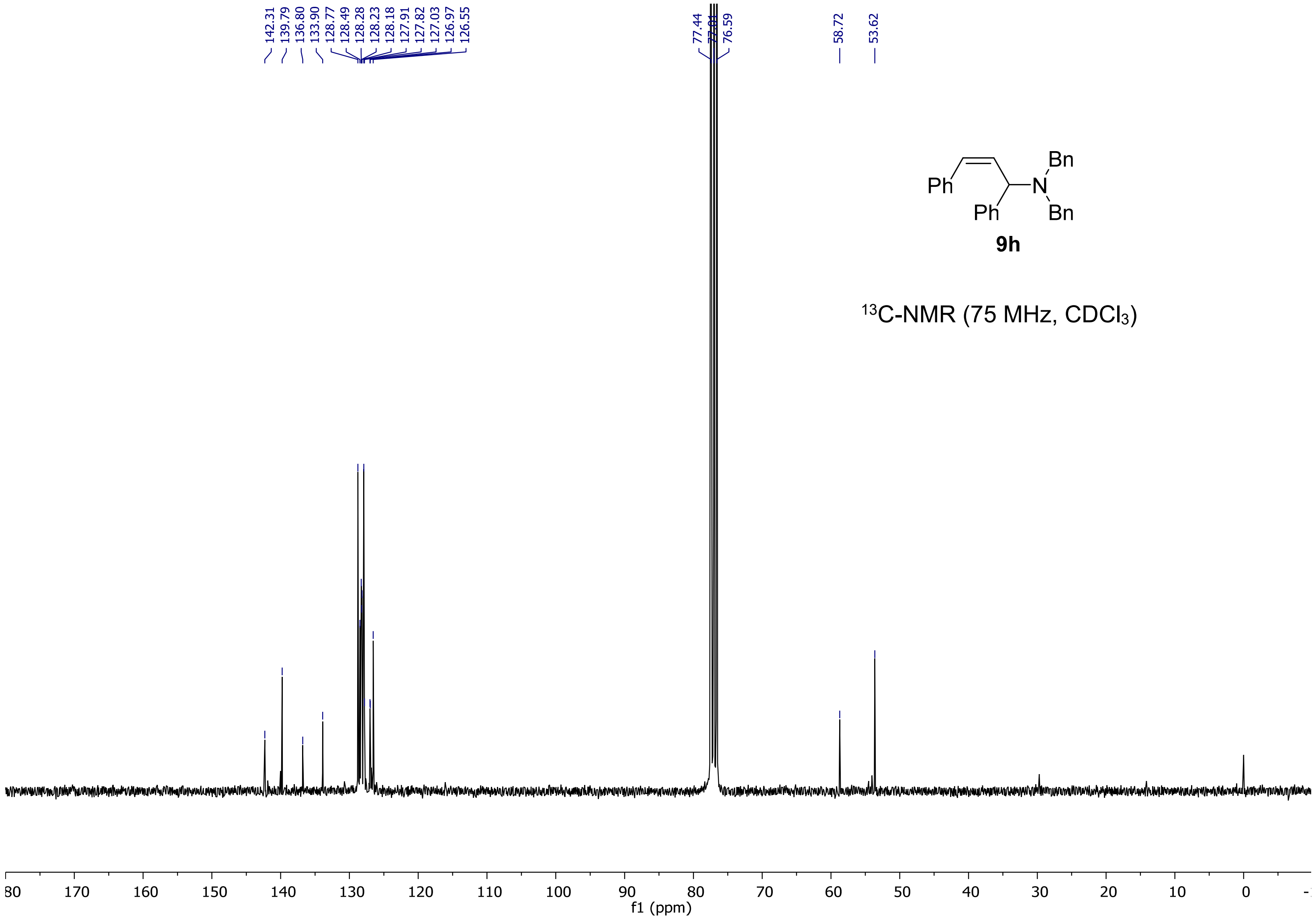

Supplementary Figure 120. ^{13}C-NMR spectra for compound **9h**

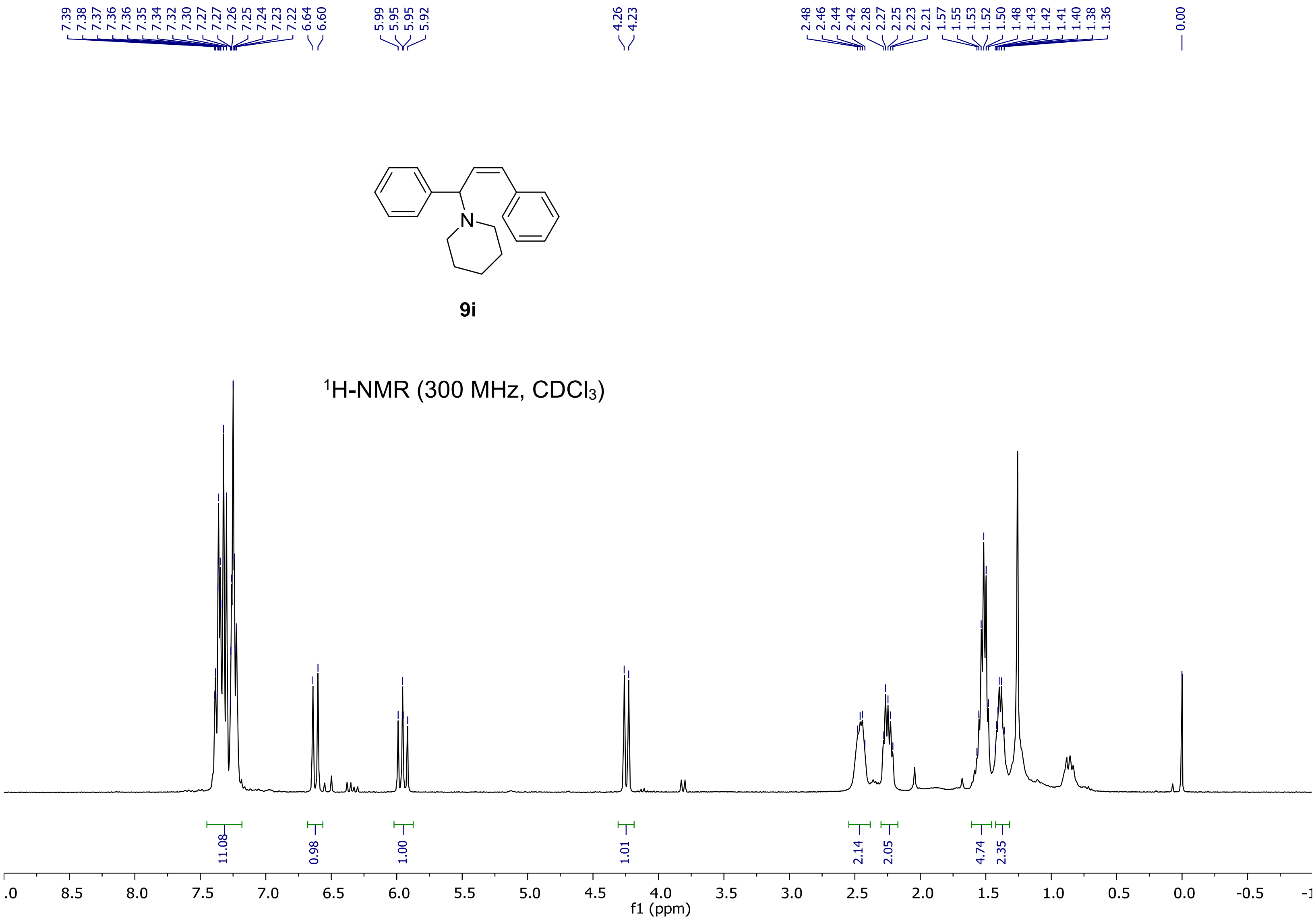

Supplementary Figure 121. ^{1}H-NMR spectra for compound **9i**

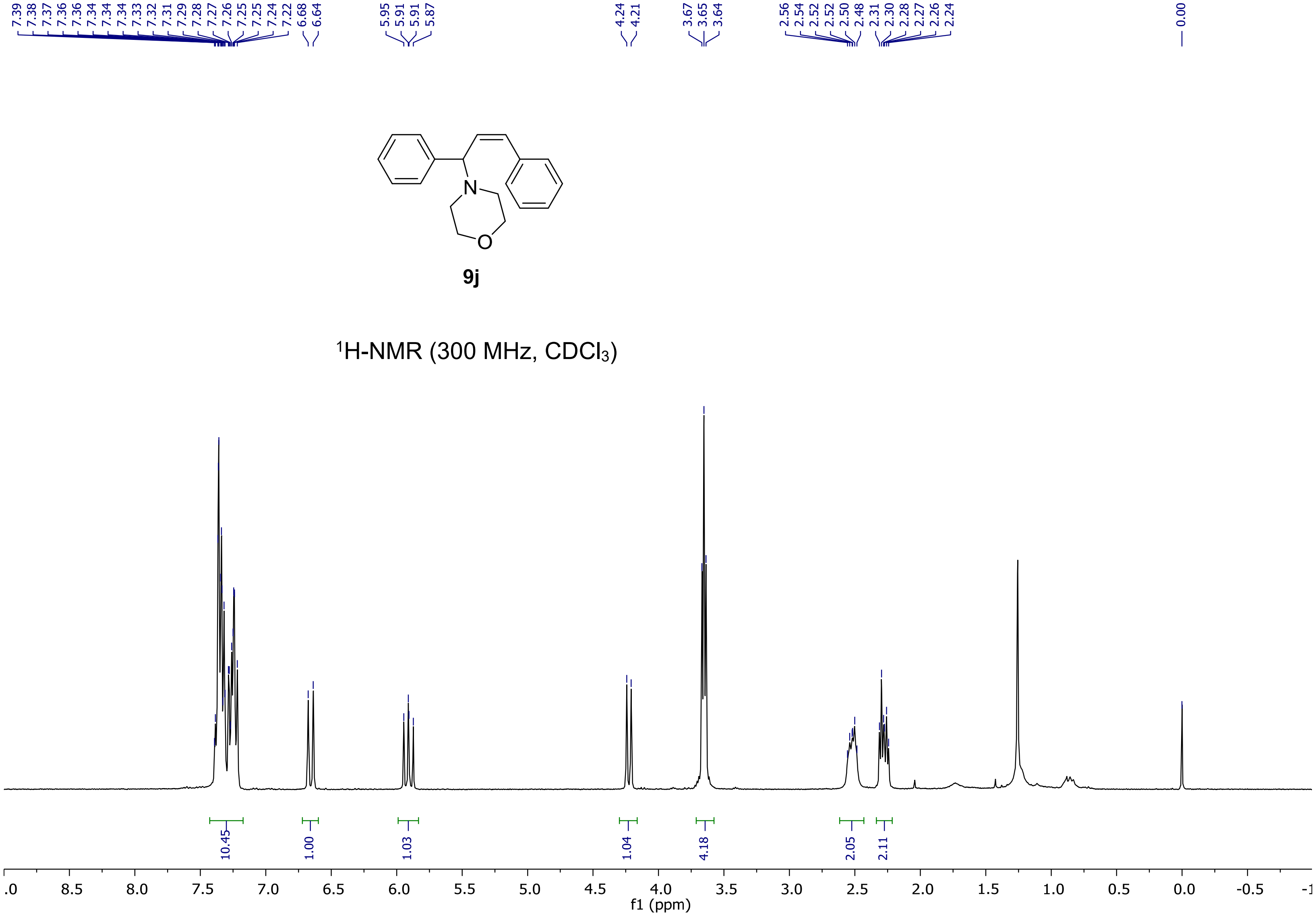

Supplementary Figure 122. ^{1}H-NMR spectra for compound **9j**

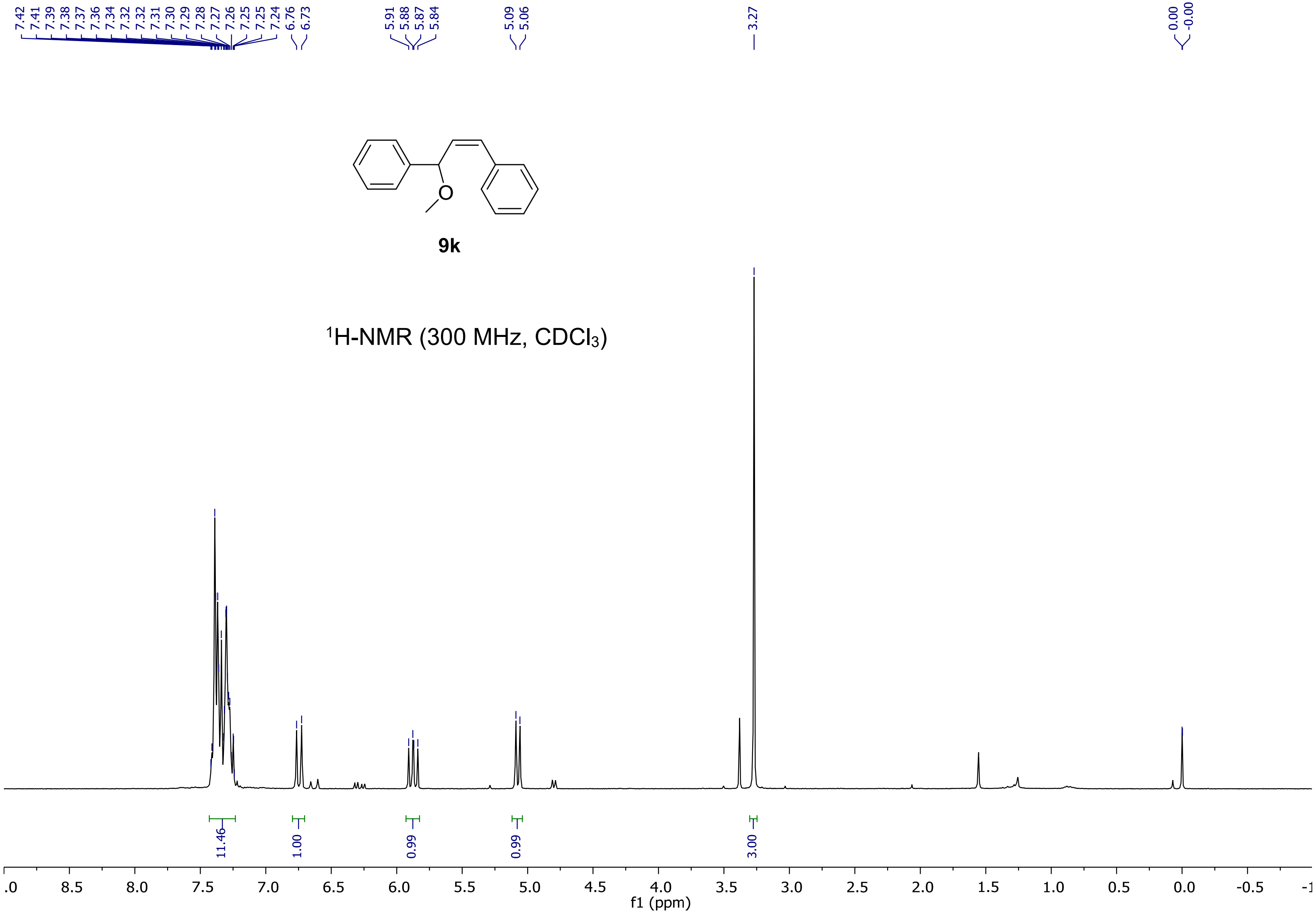

Supplementary Figure 123. ^{1}H-NMR spectra for compound **9k**

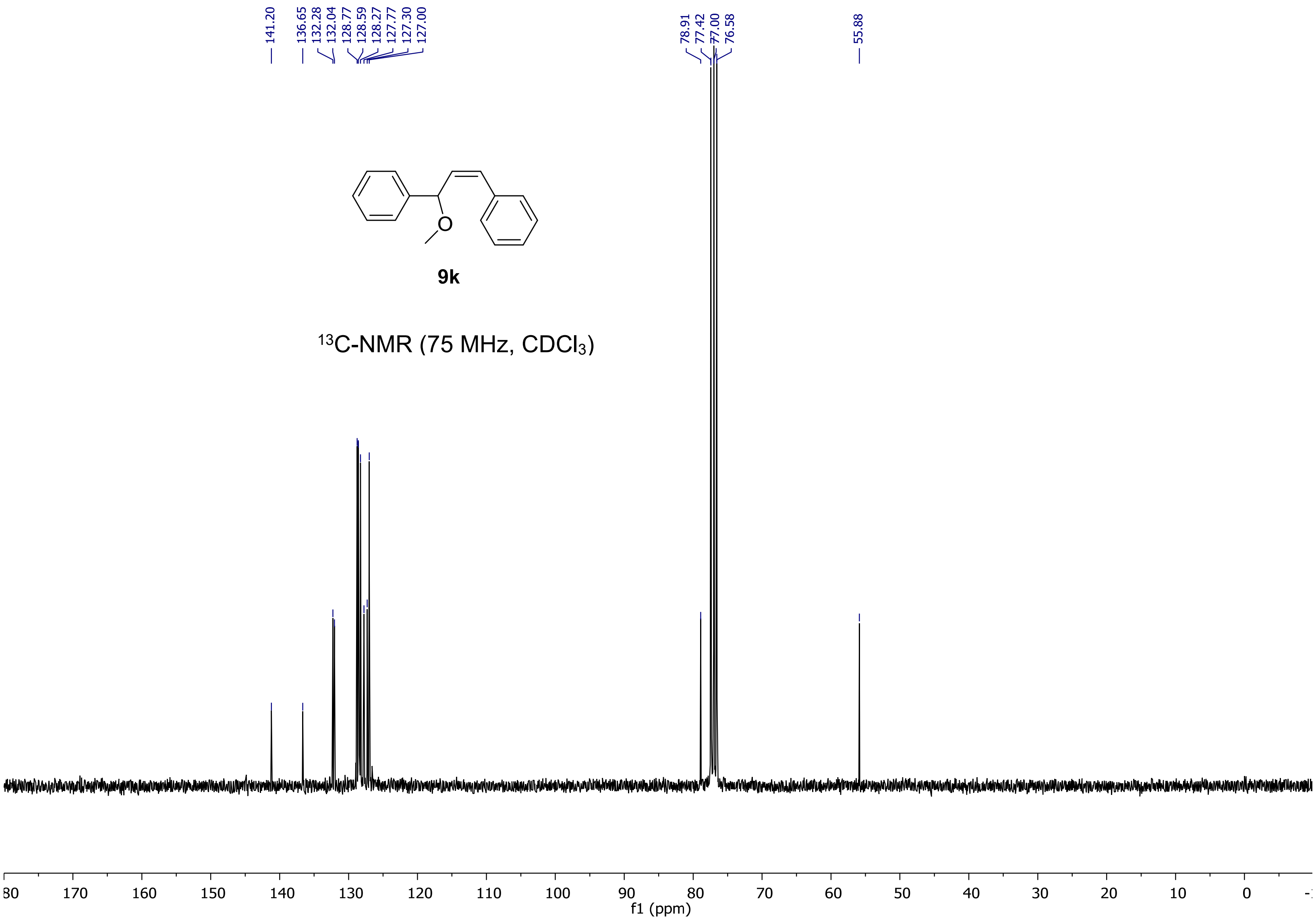

Supplementary Figure 124. ^{13}C-NMR spectra for compound **9k**

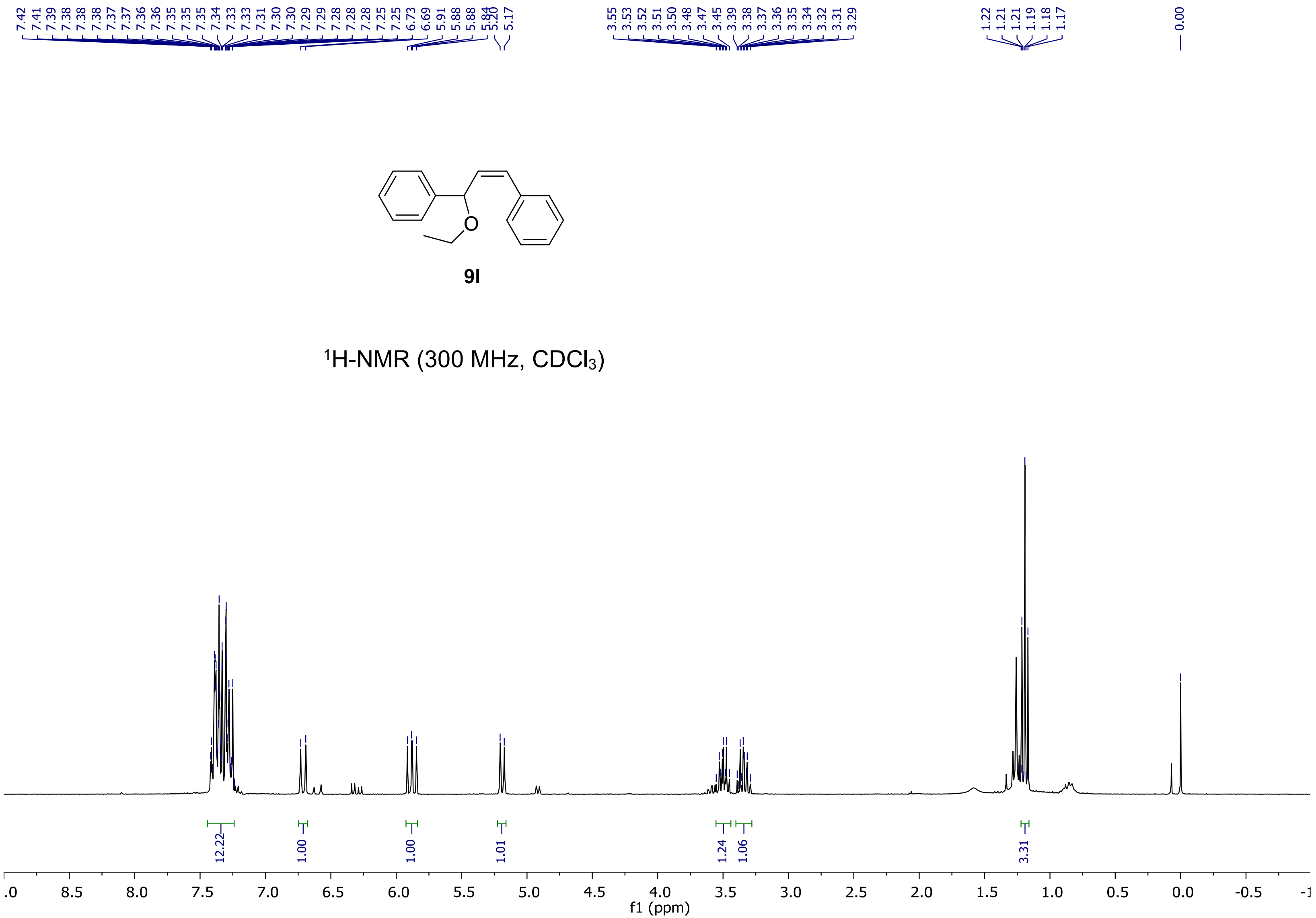

Supplementary Figure 125. ^{1}H-NMR spectra for compound **9l**

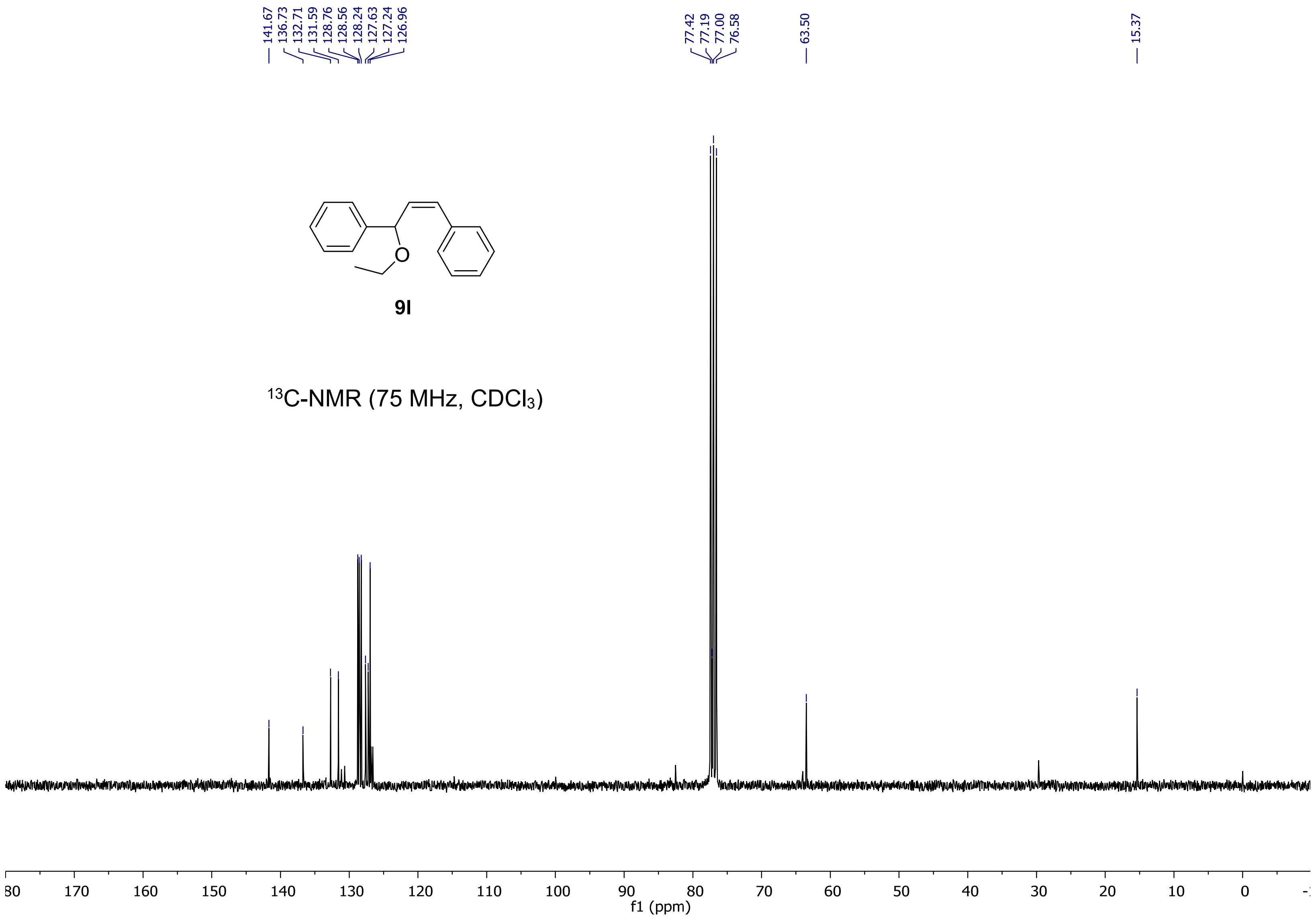

Supplementary Figure 126. ^{13}C-NMR spectra for compound **9l**

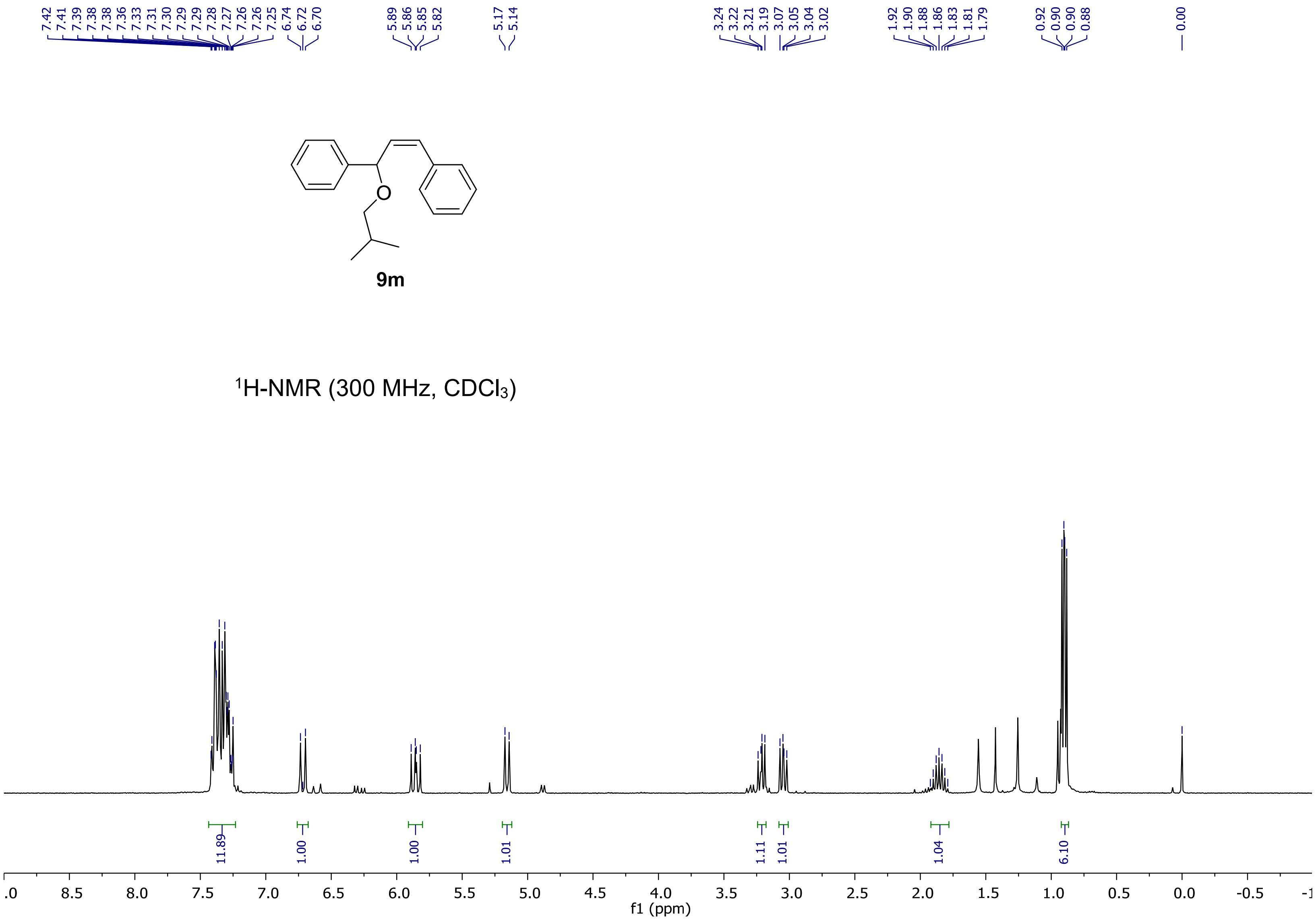

Supplementary Figure 127. ^{1}H-NMR spectra for compound **9m**

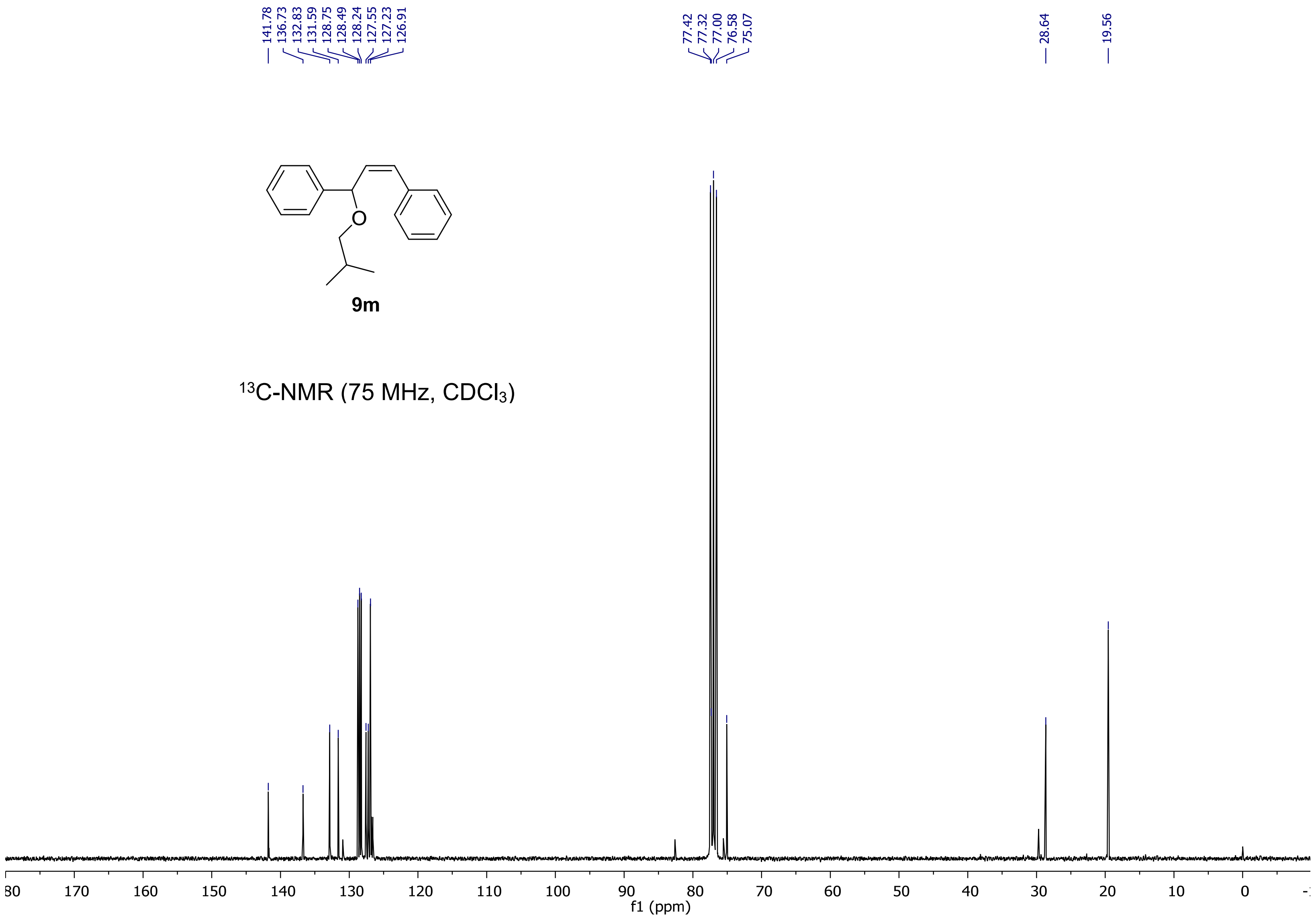

Supplementary Figure 128. ^{13}C-NMR spectra for compound **9m**

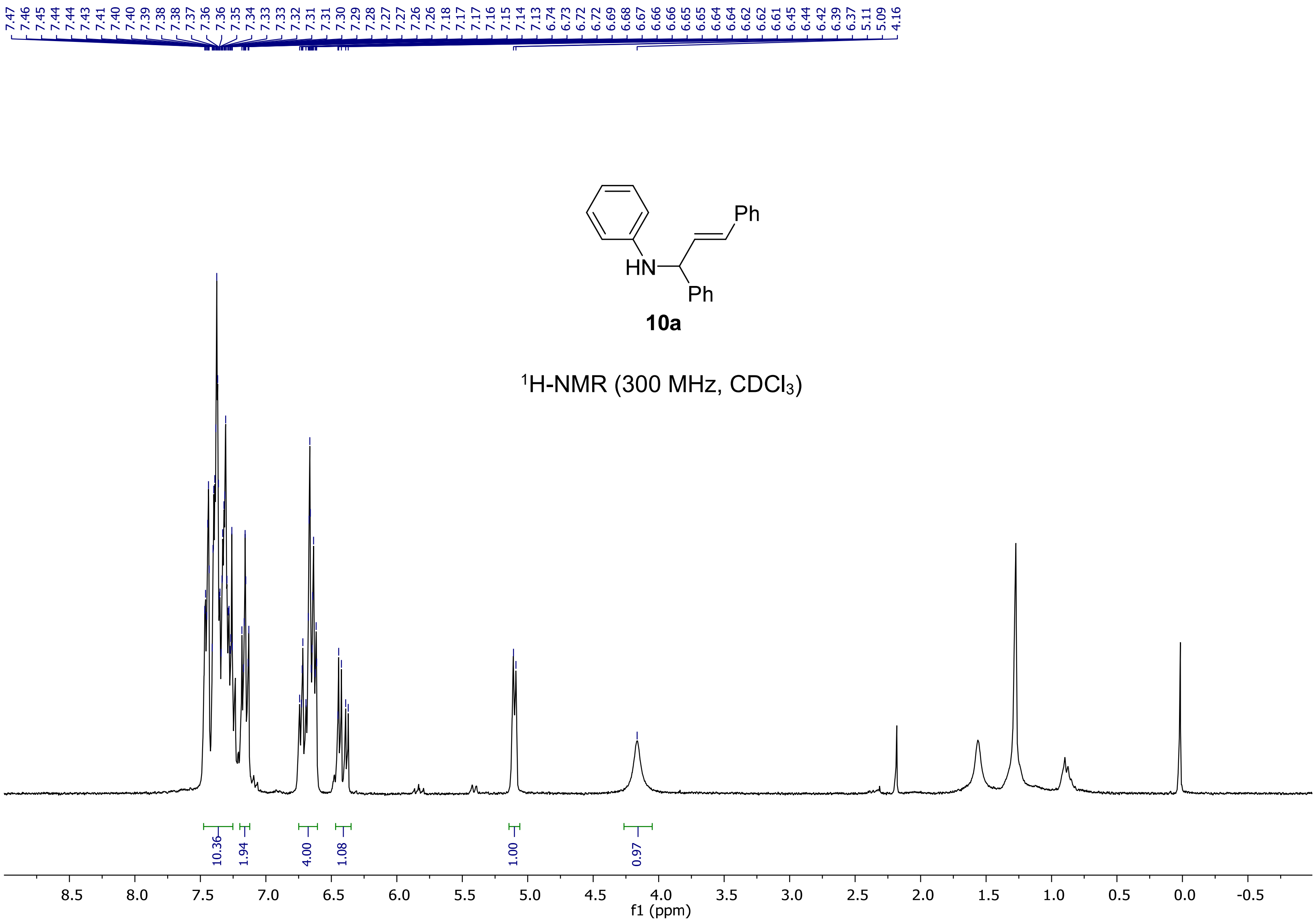

Supplementary Figure 129. ^{1}H-NMR spectra for compound **10a**

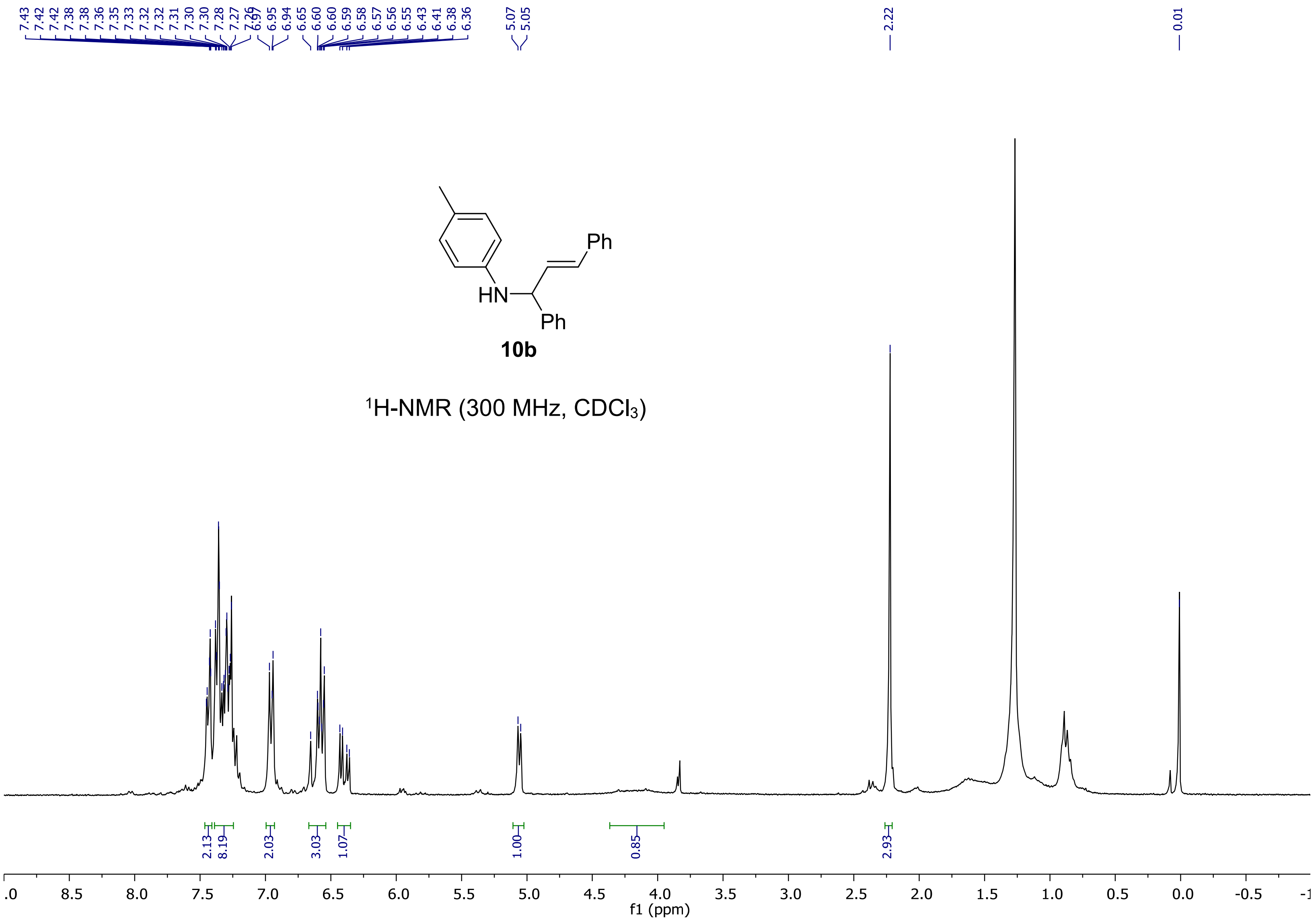

Supplementary Figure 130. ^{1}H-NMR spectra for compound **10b**

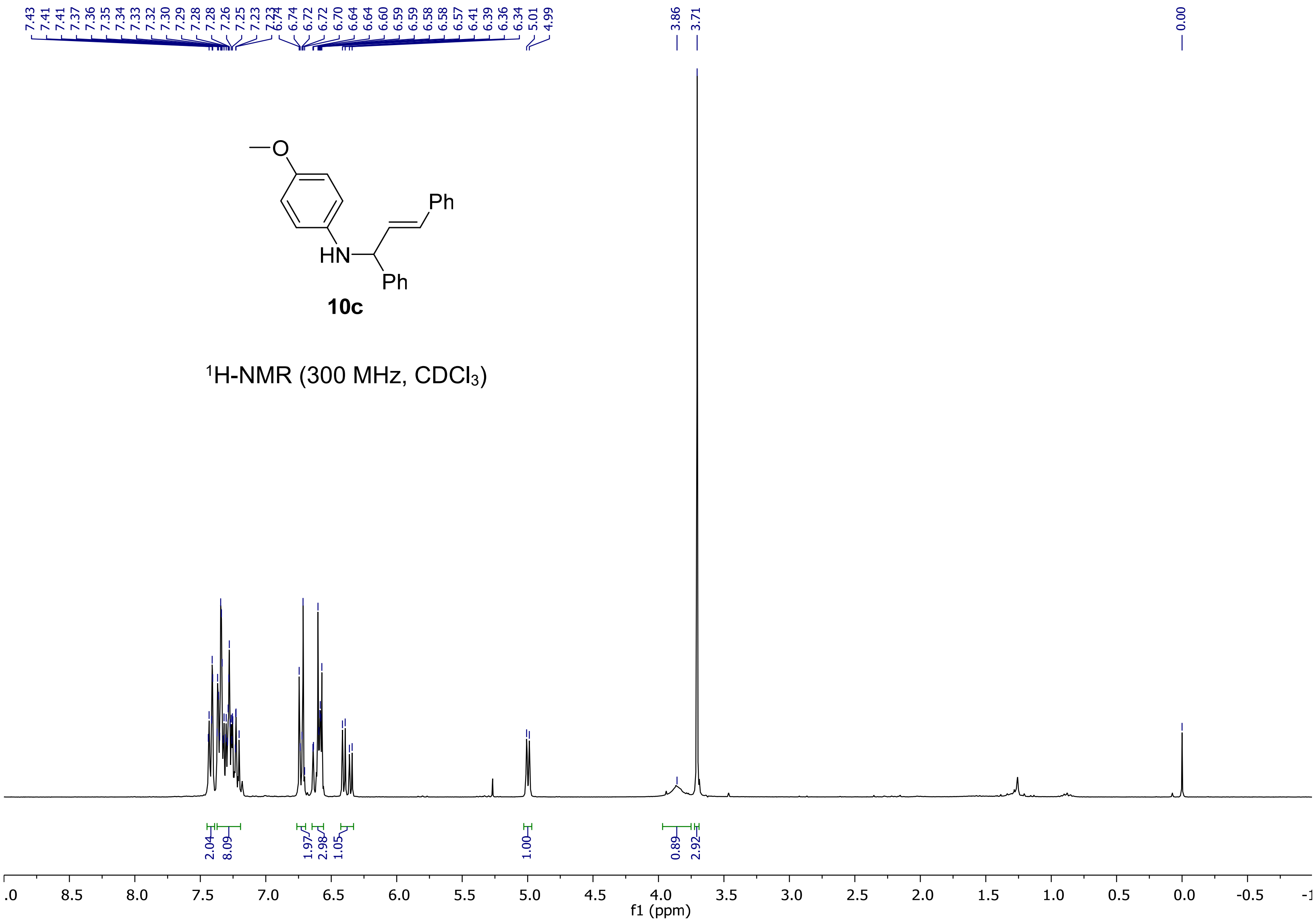

Supplementary Figure 131. ^{1}H-NMR spectra for compound **10c**

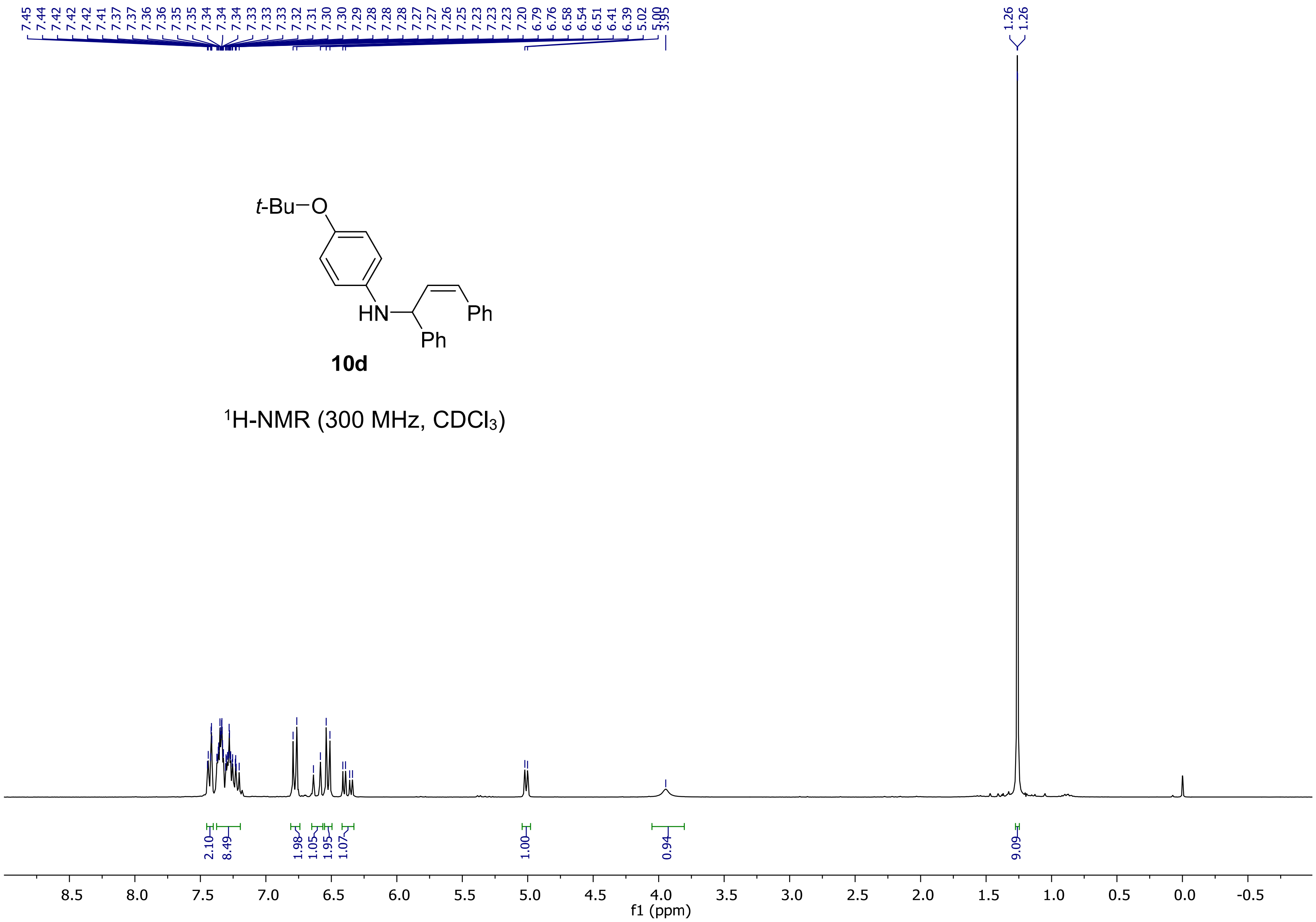

Supplementary Figure 132. ^{1}H-NMR spectra for compound **10d**

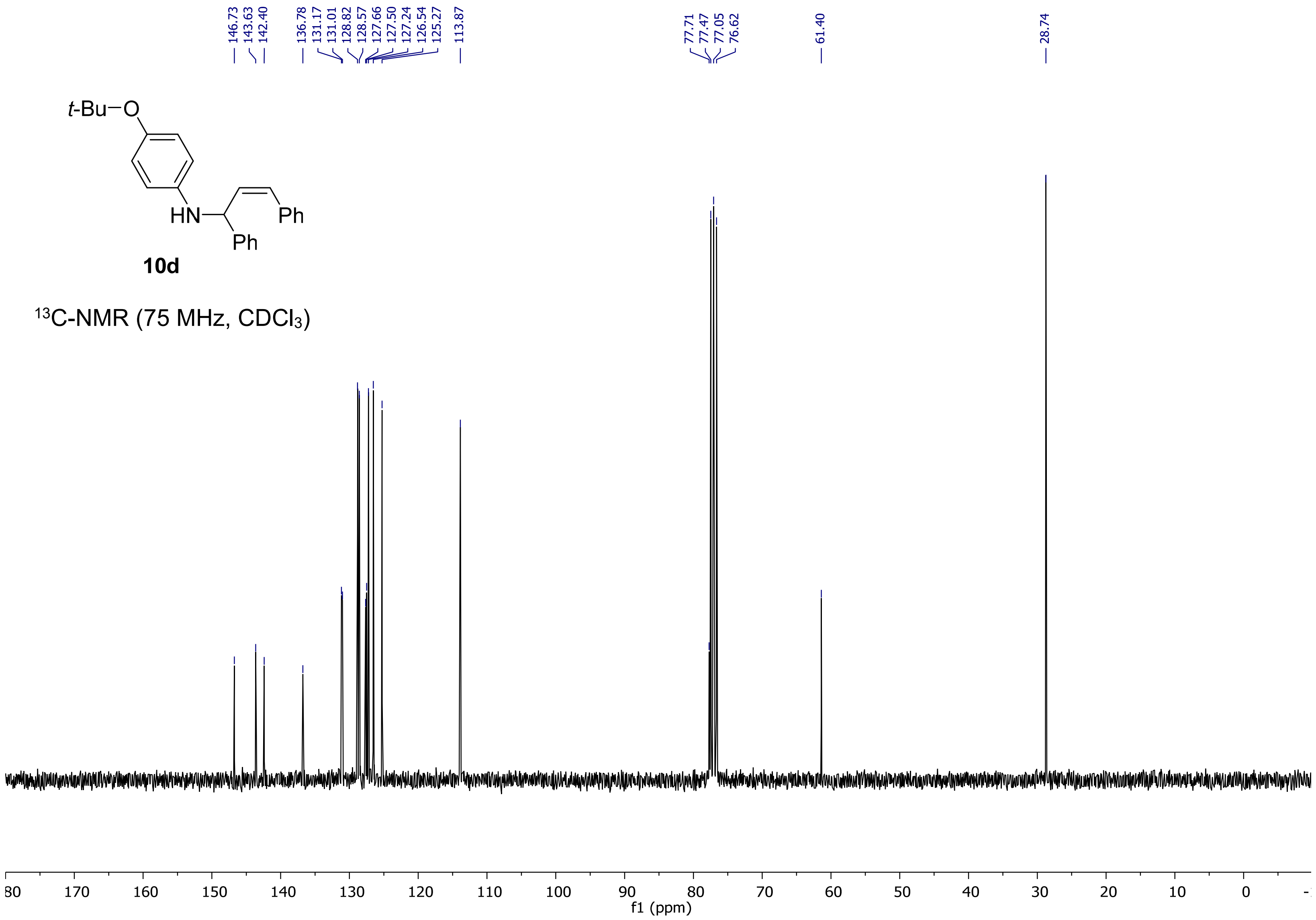

Supplementary Figure 133. ^{13}C-NMR spectra for compound **10d**

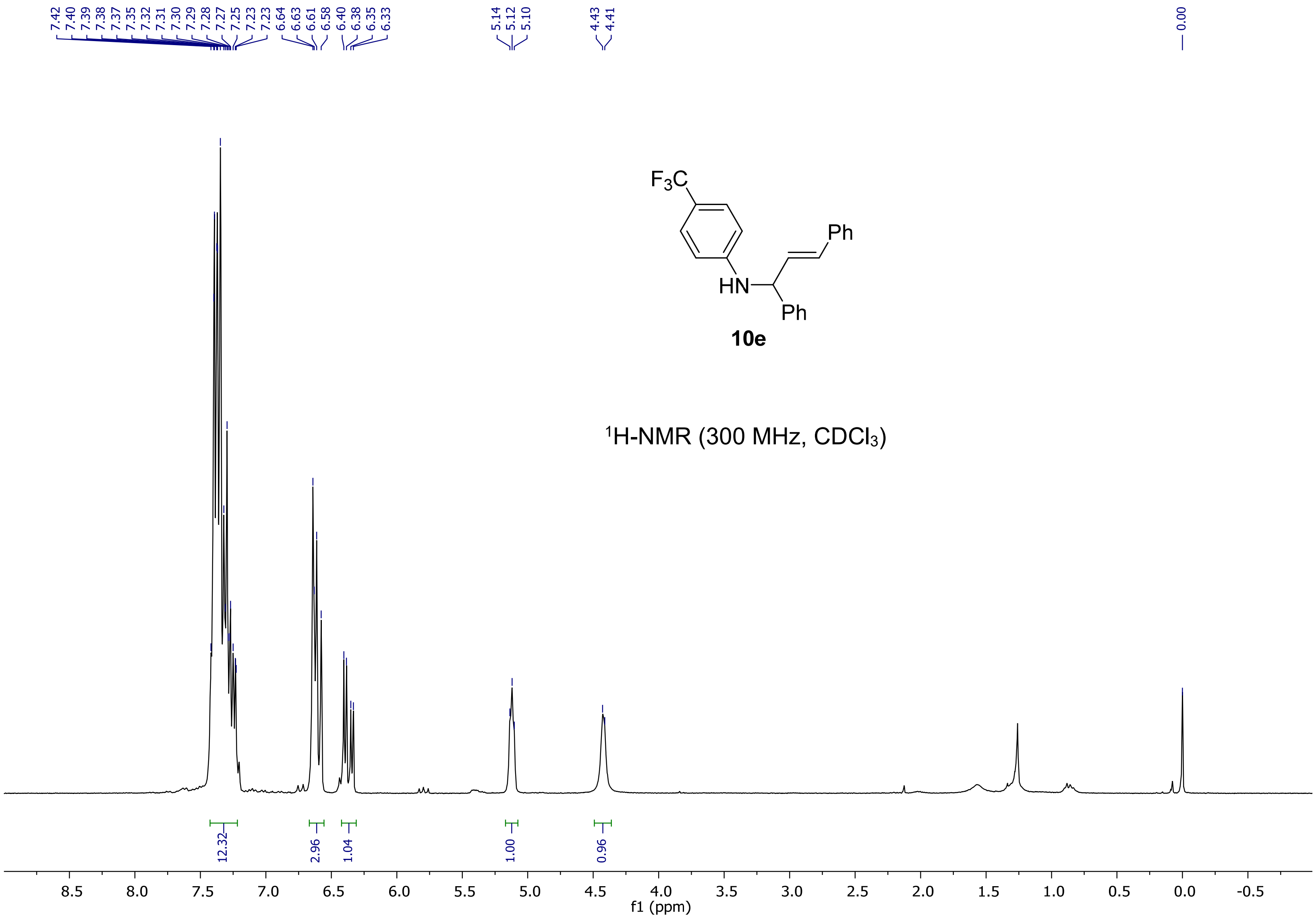

Supplementary Figure 134. ^{1}H-NMR spectra for compound **10e**

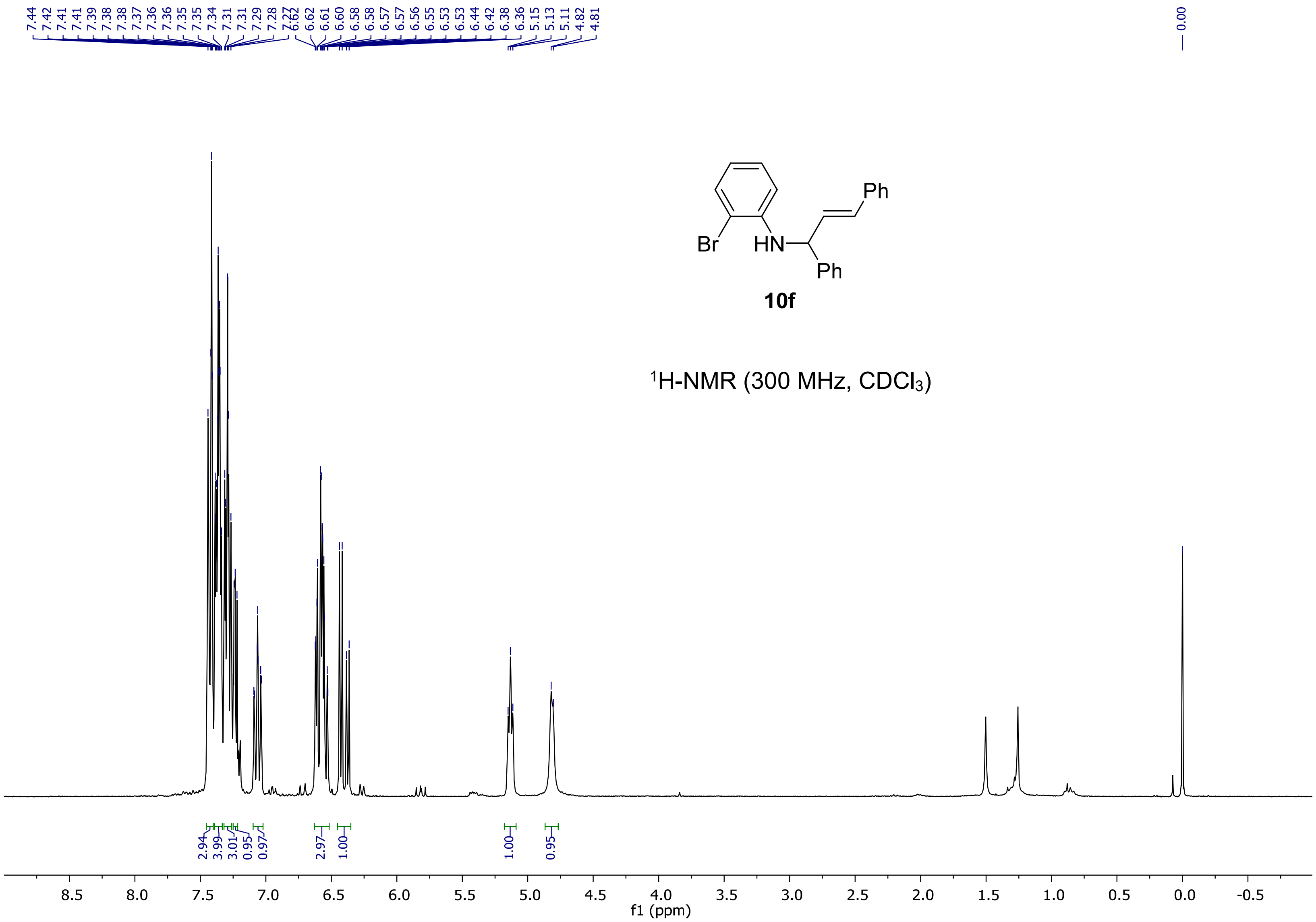

Supplementary Figure 135. ^{1}H-NMR spectra for compound **10f**

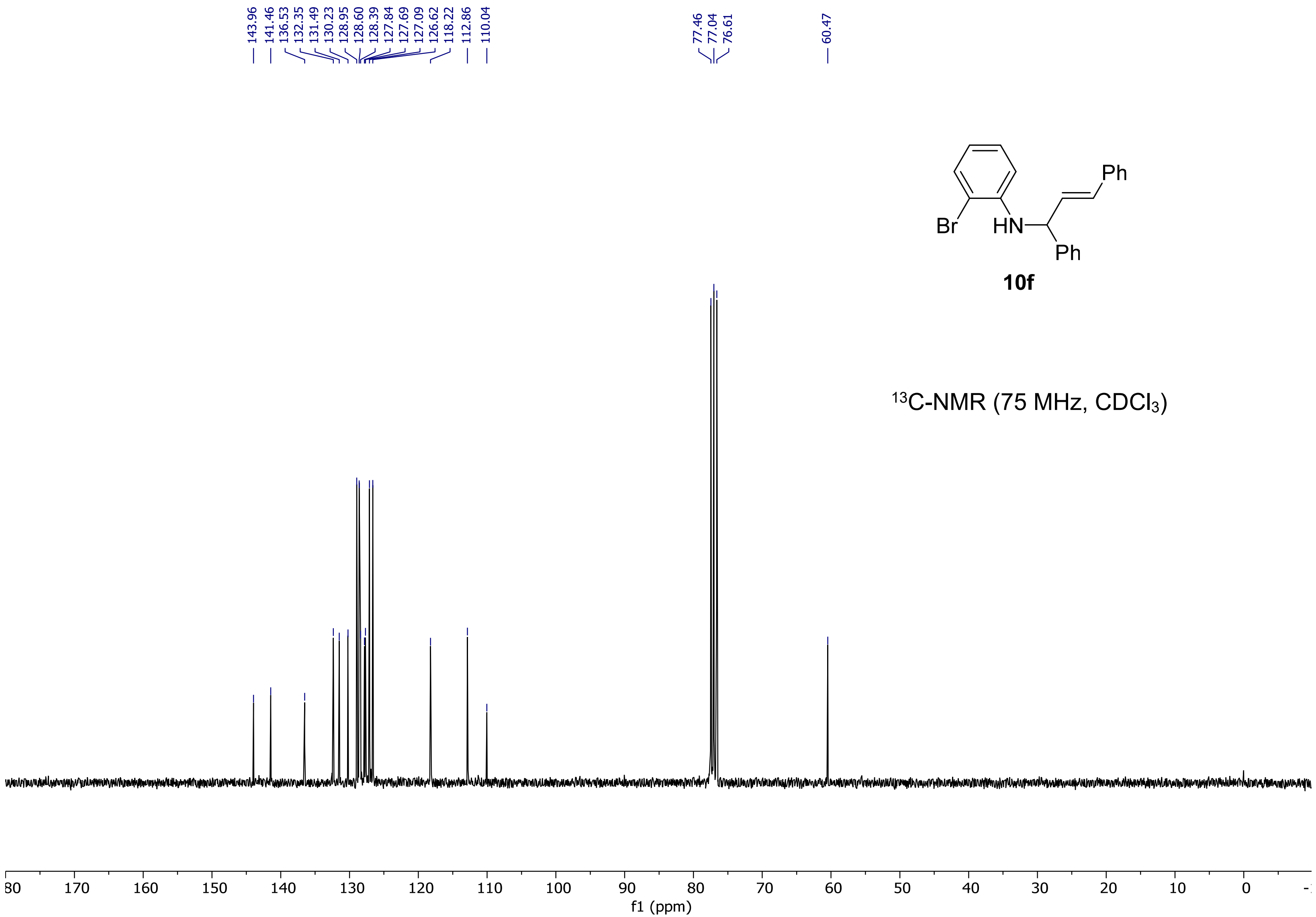

Supplementary Figure 136. ^{13}C-NMR spectra for compound **10f**

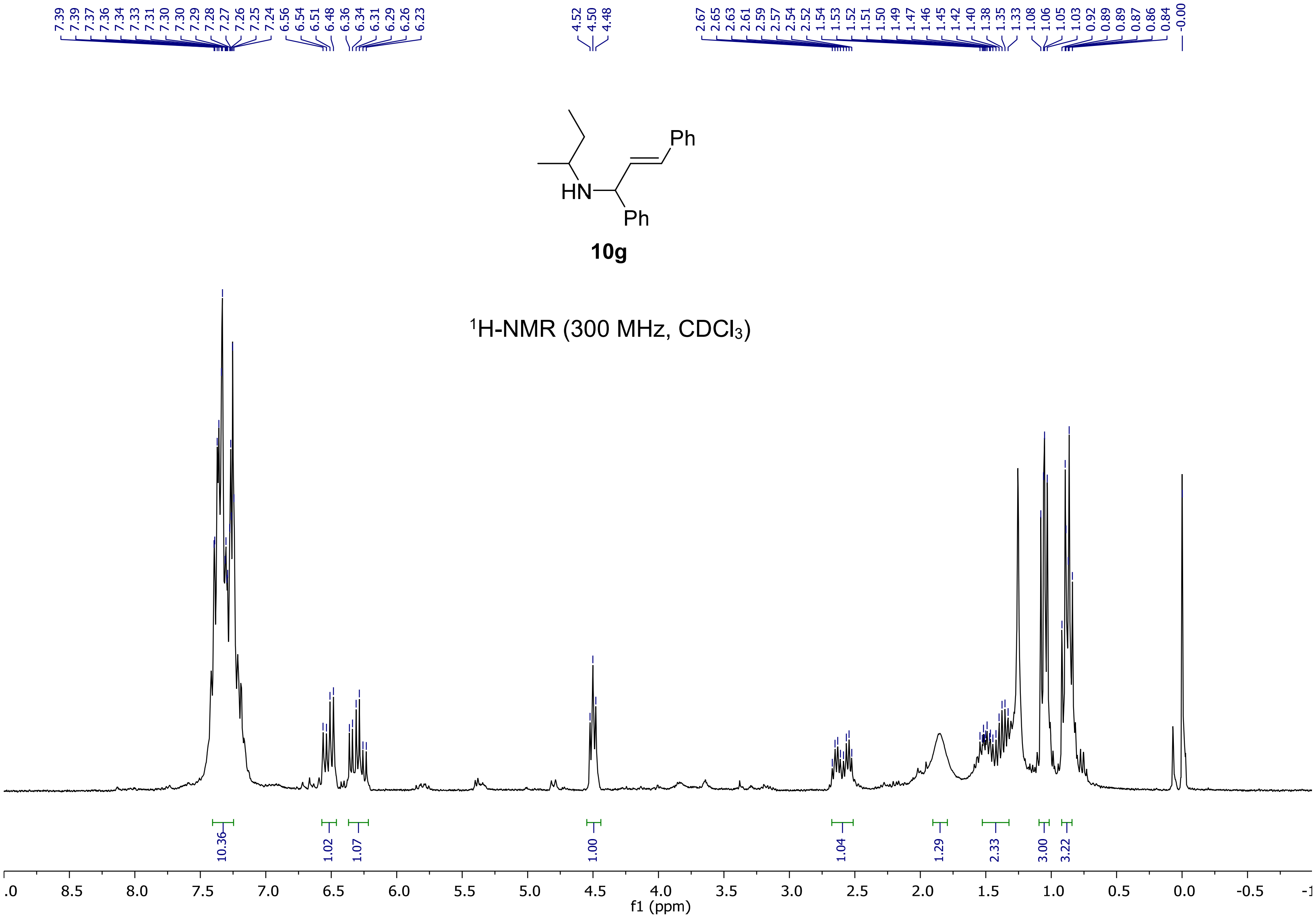

Supplementary Figure 137. ^{1}H-NMR spectra for compound **10g**

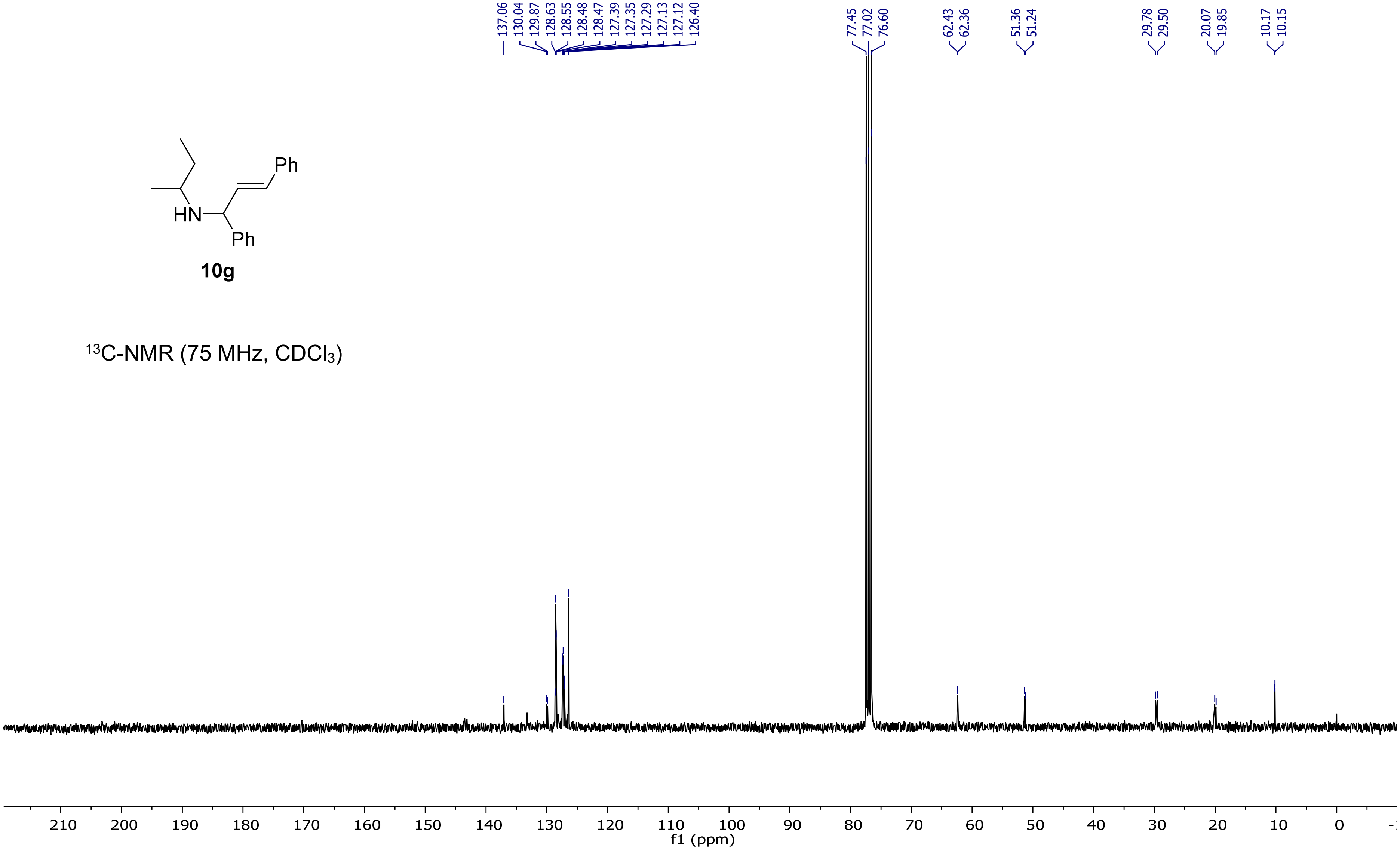

Supplementary Figure 138. ^{13}C-NMR spectra for compound **10g**

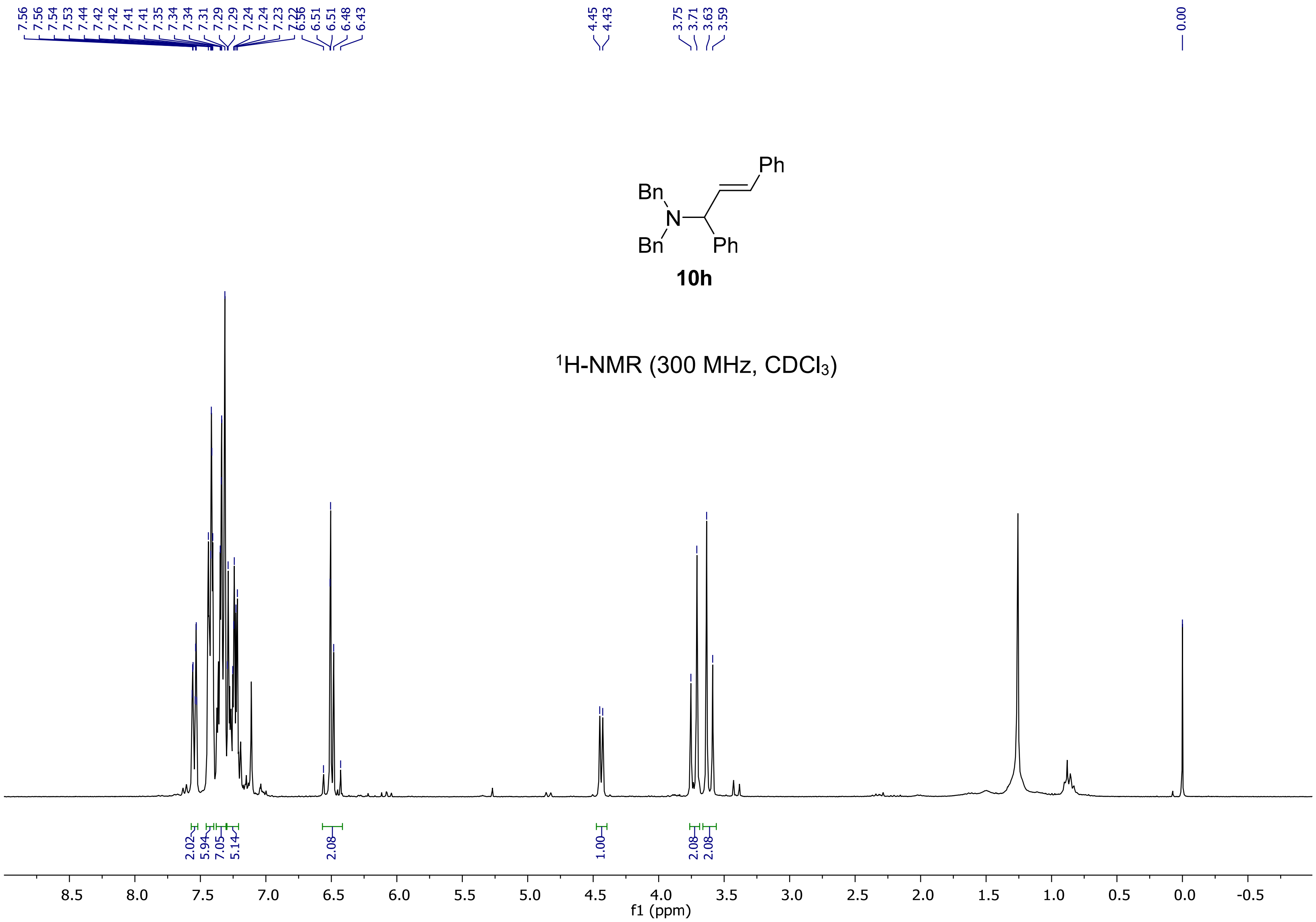

Supplementary Figure 139. ^{1}H-NMR spectra for compound **10h**

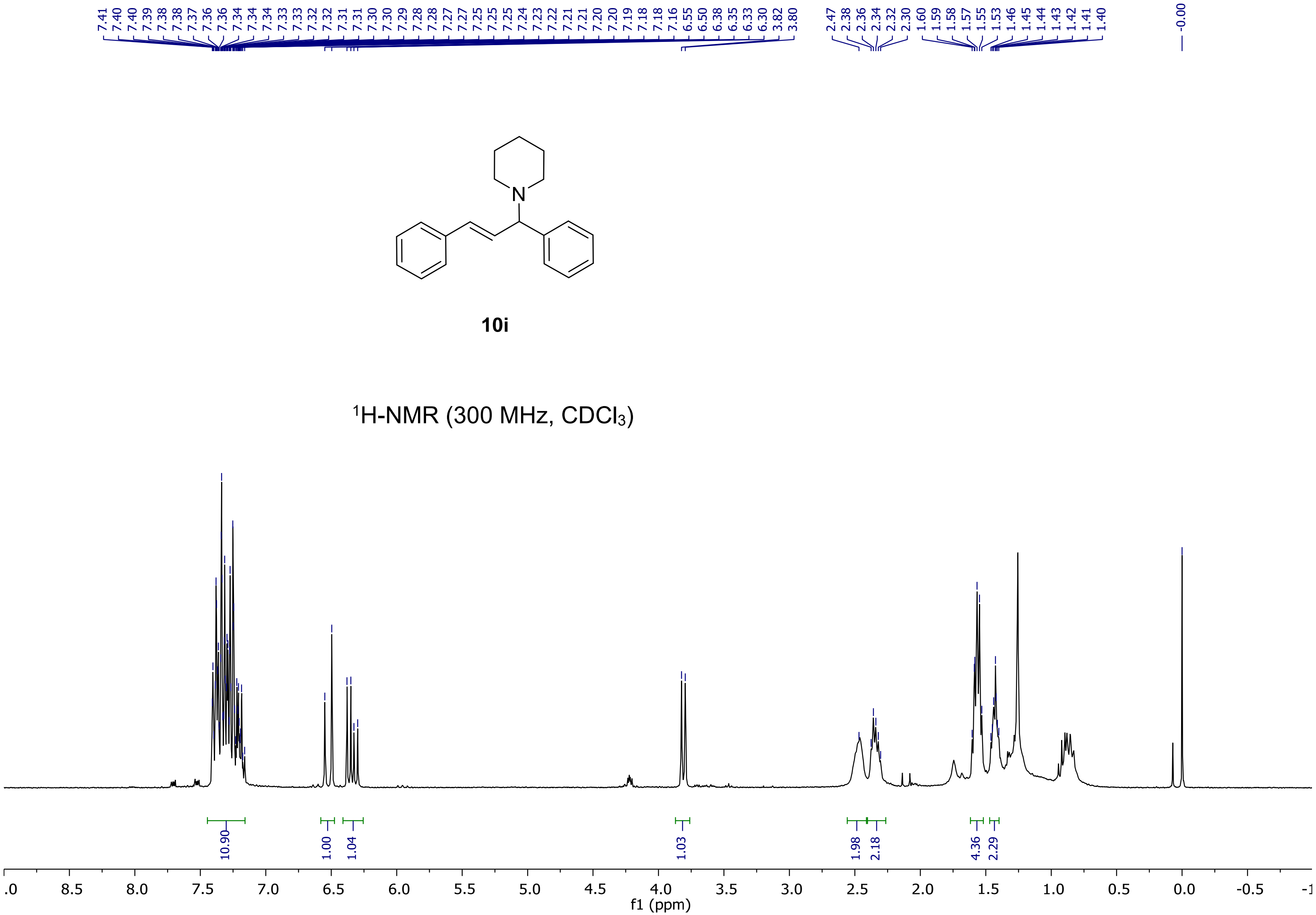

Supplementary Figure 140. ^{1}H-NMR spectra for compound **10i**

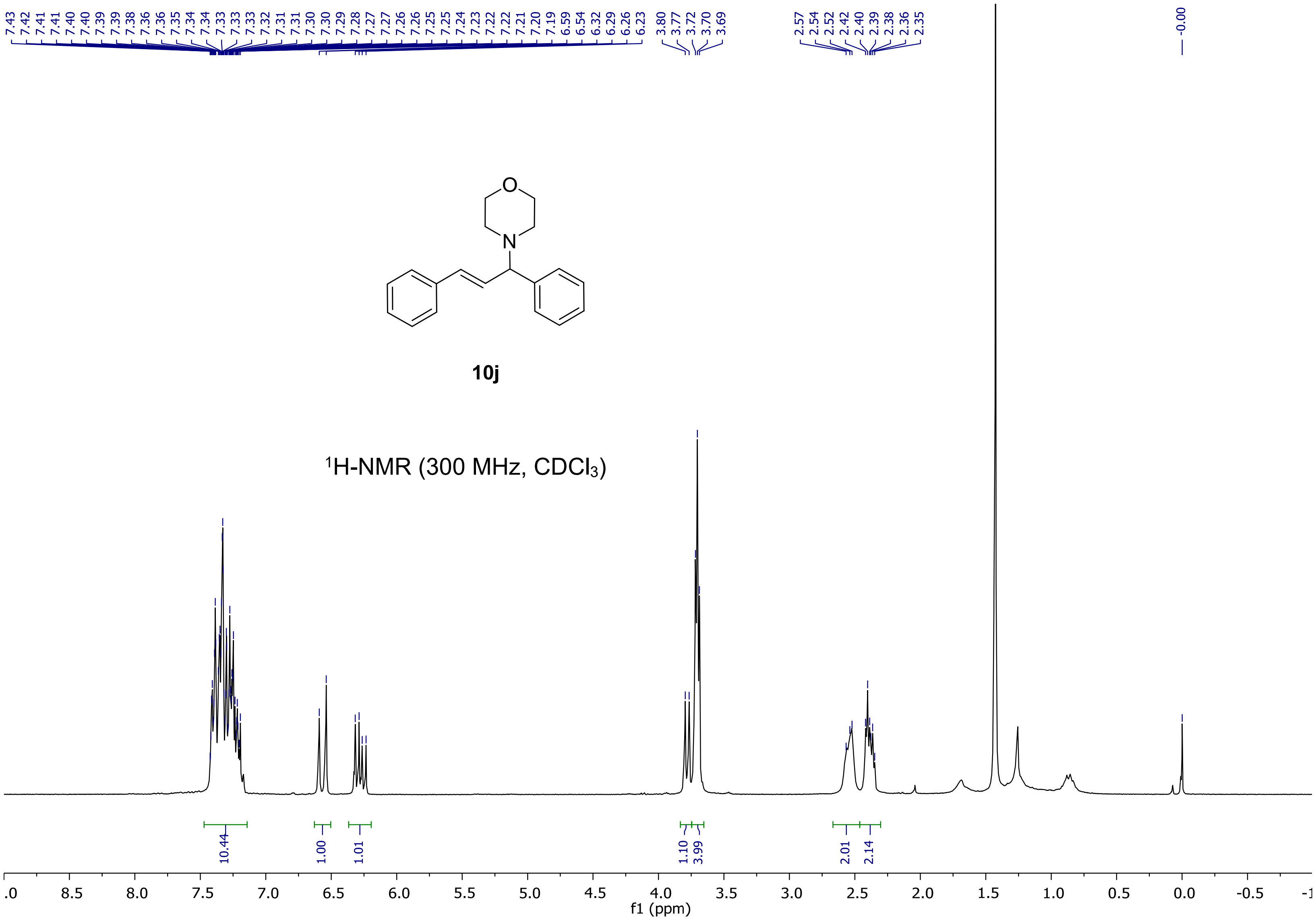

Supplementary Figure 141. ^{1}H-NMR spectra for compound **10j**

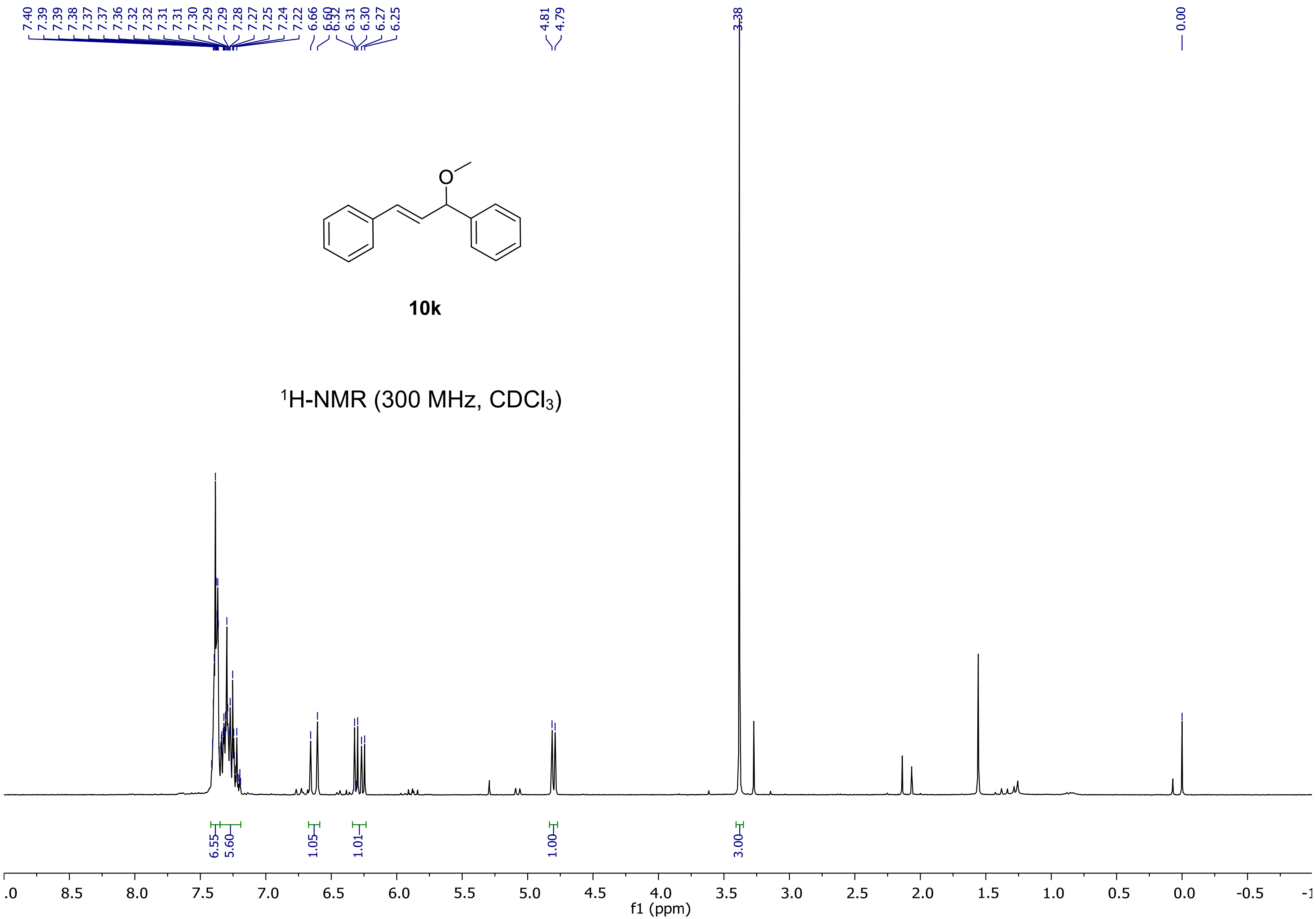

Supplementary Figure 142. ^{1}H-NMR spectra for compound **10k**

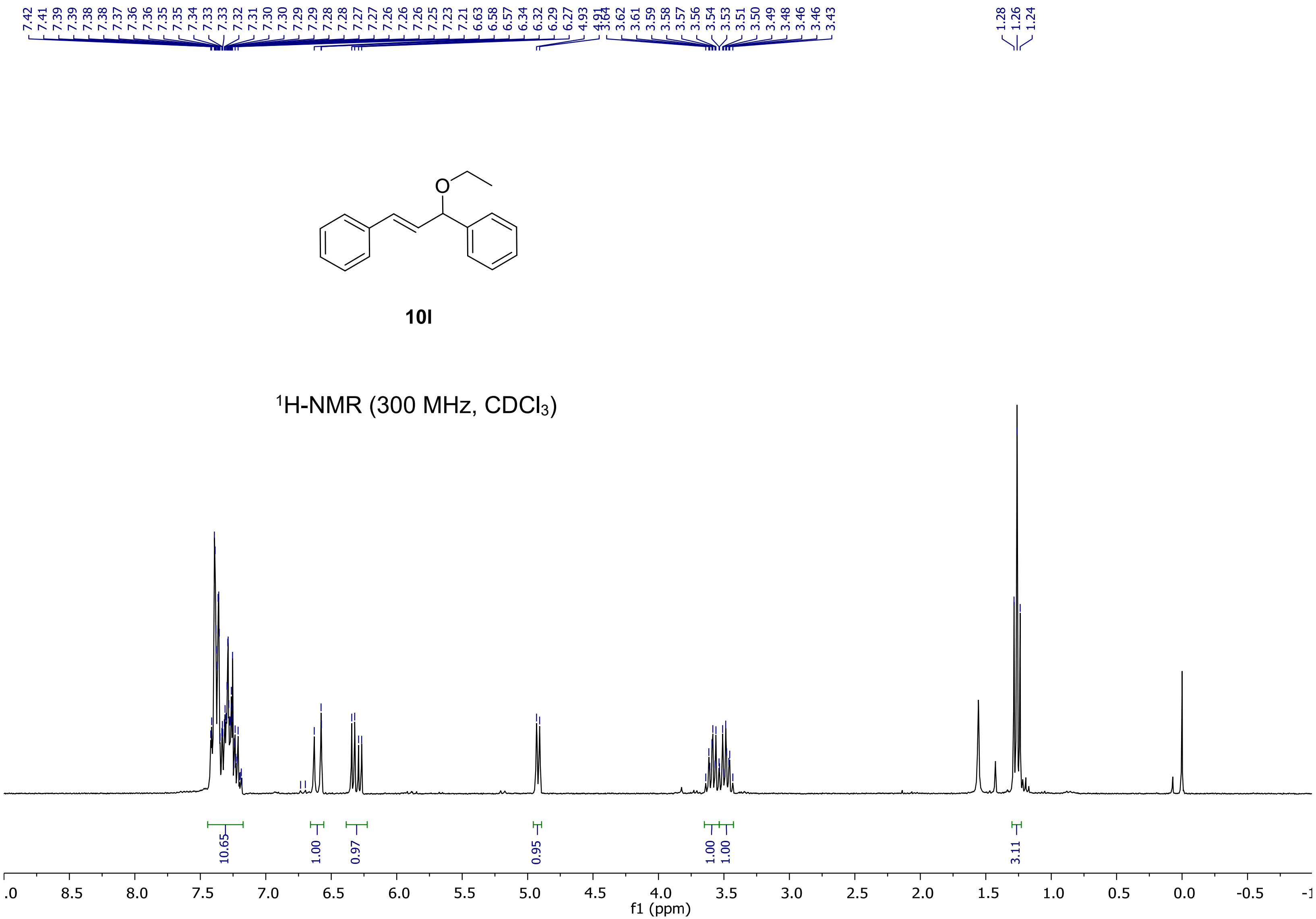

Supplementary Figure 143. ^{1}H-NMR spectra for compound **10l**

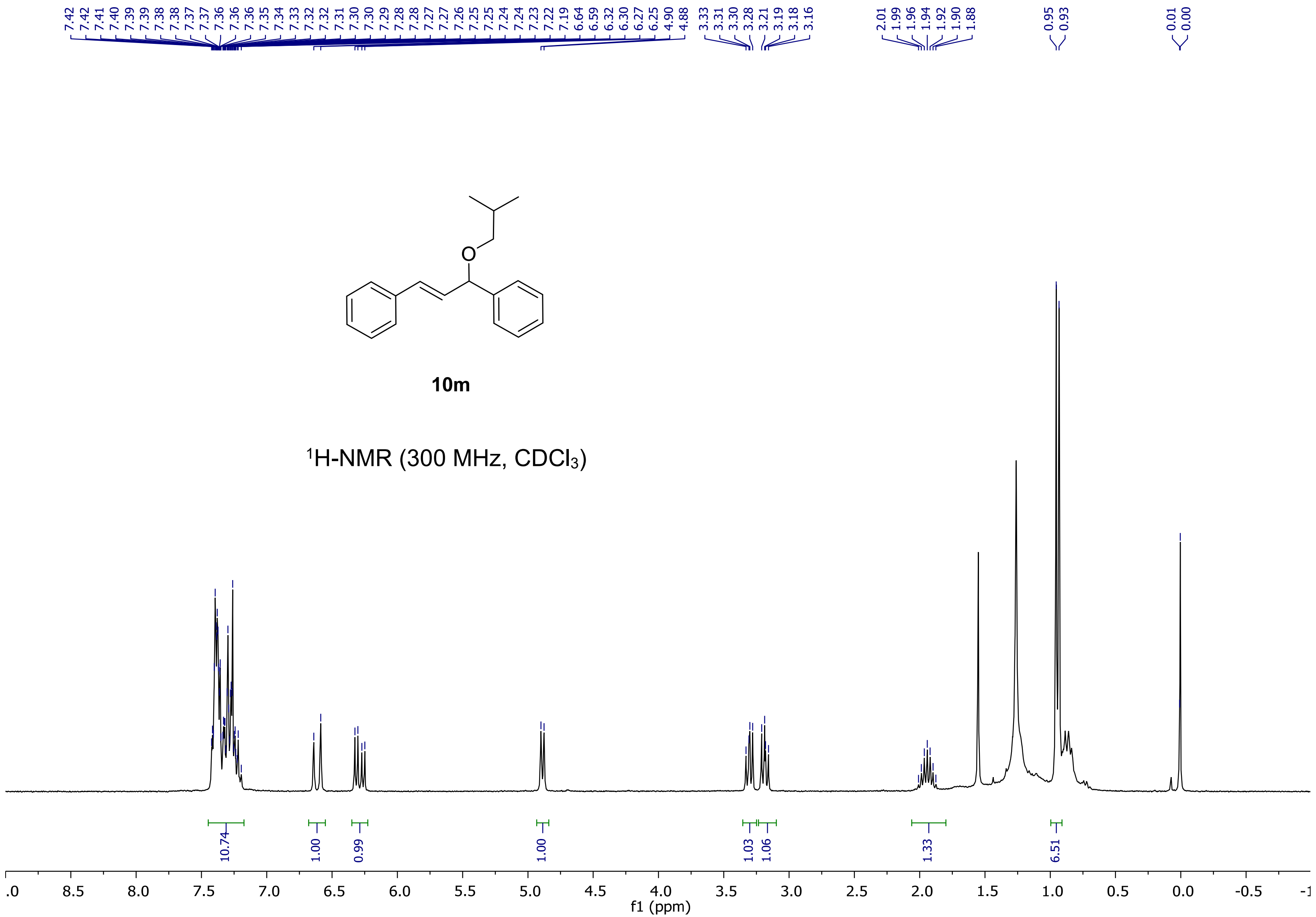

Supplementary Figure 144. ^{1}H-NMR spectra for compound **10m**

Supplementary References.

[1] Wang, Y.; Gu, M. The concept of spectral accuracy for MS. *Anal. Chem.* **82,** 7055–7062 (2010).
[2] Wang, Y. Methods for Operating MS Instruments Systems, United States Patent No. 6,983,213 (2006).
[3] Ochiaia, N.; Sasamoto, K.; MacNamara, K. Characterization of sulphur compounds in whisky by full evaporation dynamic headspace and selectable one-dimensional/two-dimensional retention time locked gas chromatography-mass spectrometry with simultaneous element-specific detection. *J. Chromatogr. A*, **1270**, 296–304 (2012).
[4] Ho, H.-P.; Lee, R.-Y.; Chen, C.-Y.; Wang, S.-R.; Li, Z.-G.; Lee, M.-R. Identification of new minor metabolites of penicillin G in human serum by multiple-stage tandem mass spectrometry. *Rapid Commun. Mass Spectrom.* **25**, 25–32 (2011).
[5] Gonda, Z.; Novák, Z. Transition-metal-free *N*-arylation of pyrazoles with diaryliodonium salts *Chem. - Eur. J.* **21**, 16801–16806 (2015).
[6] Li, X.-D.; Xie, L.-J.; Kong, D.-L.; Liu, L.; Cheng, L. Metal-free allylation of electron-rich heteroaryl boronic acids with allylic alcohols. *Tetrahedron* **72**, 1873–1880 (2016).
[7] Lee, B. H.; Choi, Y. L.; Shin, S.; Heo, J.-N. Stereoselective palladium-catalyzed α-arylation of 3-aryl-1-indanones: an asymmetric synthesis of (+)-pauciflorol. F *J. Org. Chem.* **76**, 6611–6618 (2011).
[8] Chan, C.-K.; Tsai, Y.-L.; Chang, M.-Y. $Bi(OTf)_3$ catalyzed disproportionation reaction of cinnamyl alcohols. *Tetrahedron* **73**, 3368–3376 (2017).
[9] Zhuang, M.; Du, H. Chiral Brønsted acid catalyzed enantioselective intermolecular allylic aminations. *Org. Biomol. Chem.* **12**, 4590–4593 (2014).
[10] Johnston, A. J. S.; McLaughlin, M. G.; Reid, J. P.; Cook, M. J. NaH mediated isomerisation–allylation reaction of 1,3-substituted propenols. *Org. Biomol. Chem.* **11**, 7662–7666 (2013).
[11] Kinoshita, N.; Kawabata, T.; Tsubaki, K.; Bando, M.; Fuji, K. Use of zinc enolate, free from other metals, in enantioselective palladium-catalyzed allylic alkylation. *Tetrahedron* **62**, 1756–1763 (2006).
[12] Shibatomi, K.; Muto, T.; Sumikawa, Y.; Narayama, A.; Iwasa, S. Development of a new chiral spiro oxazolinylpyridine ligand (Spymox) for asymmetric catalysis. *Synlett* 241–244 (2009).
[13] Mino, T.; Wakui, K.; Oishi, S.; Hattori, Y.; Sakamoto, M.; Fujita, T. Kinetic resolution of allylic esters in palladium-catalyzed asymmetric allylic alkylations using C–N bond axially chiral aminophosphine ligands. *Tetrahedron Asymmetry* **19**, 2711–2716 (2008).
[14] Caminiti, N. S.; Goodstein, M. B.; Leibler, I. N.-M.; Holtzman, B. S.; Jia, Z. B.; Martini, M. L.; Nelson, N. C.; Bunt, R. C. Reversible nucleophilic addition can lower the observed enantioselectivity in palladium-catalyzed allylic amination reactions with a variety of chiral ligands. *Tetrahedron Lett.* **56**, 5445–5448 (2015).
[15] Discekici, E. H.; Treat, N. J.; Poelma, S. O.; Mattson, K. M.; Hudson, Z. M.; Luo, Y.; Hawker, C. J.; Read de Alaniz, J. A highly reducing metal-free photoredox catalyst: design and application in radical dehalogenations. *Chem. Commun.* **51**, 11705–11708 (2015).
[16] McCarthy, B. G.; Pearson, R. M.; Lim, C.-H.; Sartor, S. M.; Damrauer, N. H.; Miyake, G. M. Structure–property relationships for tailoring phenoxazines as reducing photoredox catalysts *J. Am. Chem. Soc.* **140**, 5088–5101 (2018).
[17] Cao, Z.; Liu, Y.; Feng, X.; Zhuang, M.; Du, H. Pd-Catalyzed asymmetric allylic alkylation of indoles and pyrroles by chiral alkene-phosphine ligands. *Org. Lett.* **13**, 2164–2167 (2011).
[18] Trillo, P.; Baeza, A.; Nájera, C. Fluorinated alcohols as promoters for the metal-free direct substitution reaction of allylic alcohols with nitrogenated, silylated, and carbon nucleophiles. *J. Org. Chem.* **77**, 7344–7354 (2012).
[19] Shirakawa, S.; Kobayashi, S. Surfactant-type Brønsted acid catalyzed dehydrative nucleophilic substitutions of alcohols in water. *Org. Lett.* **9**, 311–314 (2007).
[20] Mino, T.; Nishikawa, K.; Asano, M.; Shima, Y.; Ebisawa, T.; Yoshida, Y.; Sakamoto, M. Chiral *N*-1-adamantyl-*N*-*trans*-cinnamylaniline type ligands: synthesis and application to palladium-catalyzed asymmetric allylic alkylation of indoles. *Org. Biomol. Chem.* **14**, 7509–7519 (2016).
[21] Feng, B.; Pu, X.-Y.; Liu, Z.-C.; Xiao, W.-J.; Chen, J.-R. Highly enantioselective Pd-catalyzed indole allylic alkylation using binaphthyl-based phosphoramidite-thioether ligands. *Org. Chem. Front.* **3**, 1246-1249 (2016).

[22] Cheung, H. Y.; Yu, W.-Y.; Lam, F. L.; Au-Yeung, T. T. L.; Zhou, Z.; Chan, T. H.; Chan. A. S. C. Enantioselective Pd-catalyzed allylic alkylation of indoles by a new class of chiral ferrocenyl P/S ligands. *Org. Lett.* **9**, 4295–4298 (2007).
[23] Liu, Z.; Cao, Z.; Du, H. Highly effective chiral phosphorus amidite–olefin ligands for palladium-catalyzed asymmetric allylic substitutions. *Org. Biomol. Chem.* **9**, 5369–5372 (2011).
[24] Sun, R.; Liu, J.; Yang, S.; Chen, M.; Sun, N.; Chen, H.; Xie, X.; You, X.; Li, S.; Liu, Y. Cp_2TiCl_2-catalyzed *cis*-hydroalumination of propargylic amines with Red-Al: stereoselective synthesis of *Z*-configured allylic amines. *Chem. Commun.* **51**, 6426−6429 (2015).
[25] Ohshima, T.; Miyamoto, Y.; Ipposhi, J.; Nakahara, Y.; Utsunomiya, M.; Mashima, K. Platinum-catalyzed direct amination of allylic alcohols under mild conditions: ligand and microwave effects, substrate scope, and mechanistic study. *J. Am. Chem. Soc.* **131**, 14317-14328 (2009).
[26] Wang, Z.; Mo, H.; Cheng, D.; Bao, W. Metal-free synthesis of allylic amines by cross-dehydrogenative-coupling of 1,3-diarylpropenes with anilines and amides under mild conditions. *Org. Biomol. Chem.* **10**, 4249-4255 (2012).
[27] Ohshima, T.; Nakahara, Y.; Ipposhi, J.; Miyamoto, Y.; Mashima, K. Direct substitution of the hydroxy group with highly functionalized nitrogen nucleophiles catalyzed by Au(III). *Chem. Commun.* **47**, 8322-8324 (2011).
[28] Concellón, J. M.; Suárez, J. R.; del Solar, V. Synthesis of enantiopure allylamines by reductive alkylation of amino epoxides with organolithium reagents. *Org. Lett.* **8**, 349-351 (2006).
[29] Baudoux, J.; Perrigaud, K.; Madec, P.-J.; Gaumont, A.-C.; Dez, I. Development of new SILP catalysts using chitosan as support. *Green Chem.* **9**, 1346−1351 (2007).
[30] Liu, Q.-L.; Chen, W.; Jiang, Q.-Y.; Bai, X.-F.; Li, Z.; Xu, Z.; Xu, L.-W. A D-Camphor-based Schiff base as a highly efficient N,P ligand for enantioselective palladium-catalyzed allylic substitutions. *ChemCatChem* **8**, 1495−1499 (2016).
[31] Thiemann, T. Etherification of (*E*)-1,3-diaryl- and (*E*)-1,3-diheteroaryl- prop-2-en-1-ols with primary and secondary alcohols over platinum on carbon. *J. Chem. Res.* 528−534 (2007).
[32] Miranda, M. A.; Perez-Prieto, J.; Font-Sanchis, E.; Kónya, K.; Scaiano, J. C. Flash photolysis of 1,3-Dichloro-1,3-diphenylpropane in polar solvents: generation of a stabilized γ-chloropropyl cation, subsequent formation of a propenyl cation, and nucleophilic trapping of both cations. *J. Phys. Chem. A* **102***,* 5724-5727 (1998).
[33] Mattay J., Vondenhof M. Contact and solvent-separated radical ion pairs in organic photochemistry. In: Photoinduced Electron Transfer III. Topics in Current Chemistry. Eds: Mattay J.; Springer, Berlin, Heidelberg, **159**, pag. 219-255 (1991).
[34] Silvi, M.; Arceo, E.; Jurberg, I. D.; Cassani, C.; Melchiorre, P. Enantioselective organocatalytic alkylation of aldehydes and enals driven by the direct photoexcitation of enamines. *J. Am. Chem. Soc.* **137**, 6120−6123 (2015).
[35] Kuhn, H. J.; Braslavsky, S. E.; Schmidt, R. Name and symbol of the element with atomic number 111. *Pure Appl. Chem.* **76**, 2105–2146 (2004).
[36] Hamai, S.; Hirayama, F. Actinometric determination of absolute fluorescence quantum yields. *J. Phys. Chem.* **87**, 83-89 (1983).
[37] Zhao Y.; Truhlar, D. G. The M06 suite of density functionals for main group thermochemistry, thermochemical kinetics, noncovalent interactions, excited states, and transition elements: two new functionals and systematic testing of four M06-class functionals and 12 other functionals. *Theor. Chem. Acc.* **120**, 215–241 (2008).
[38] Hehre W. J.; Ditchfield, R.; Pople, J. A. Self-consistent molecular orbital methods. XII. Further extensions of Gaussian-type basis sets for use in molecular orbital studies of organic molecules. *J. Chem. Phys.* **56**, 2257–2261 (1972).
[39] Marenich, A. V.; Cramer, C. J.; Truhlar, D. G. Universal solvation model based on solute electron density and on a continuum model of the solvent defined by the bulk dielectric constant and atomic surface tensions. *J. Phys. Chem. B* **113**, 6378–6396 (2009).
[40] Frisch, M. J.; Trucks, G. W.; Schlegel, H. B.; Scuseria, G. E.; Robb, M. A.; Cheeseman, J. R.; Scalmani, G.; Barone, V.; Mennucci, B.; Petersson, G. A. et al., Gaussian 09 Revision E.01, Wallingford CT: Gaussian, Inc., 2009.
[41] Fawcett, W. R. The ionic work function and its role in estimating absolute electrode potentials. *Langmuir* **24**, 9868-9875 (2008).
